# FAM3C/ILEI protein is elevated in psoriatic lesions and triggers psoriasiform hyperproliferation in mice

**DOI:** 10.15252/emmm.202216758

**Published:** 2023-05-25

**Authors:** Barizah Malik, Iva Vokic, Thomas Mohr, Marle Poppelaars, Martin Holcmann, Philipp Novoszel, Gerald Timelthaler, Thomas Lendl, Dana Krauss, Ulrich Elling, Michael Mildner, Josef M Penninger, Peter Petzelbauer, Maria Sibilia, Agnes Csiszar

**Affiliations:** ^1^ Center for Cancer Research Medical University of Vienna, Comprehensive Cancer Center Vienna Austria; ^2^ Department of Analytical Chemistry, Faculty of Chemistry University of Vienna Vienna Austria; ^3^ Joint Metabolome Facility University of Vienna and Medical University Vienna Vienna Austria; ^4^ Research Institute of Molecular Pathology Vienna Austria; ^5^ Institute of Molecular Biotechnology of the Austrian Academy of Sciences (IMBA) Vienna Austria; ^6^ Department of Dermatology Medical University of Vienna Vienna Austria; ^7^ Department of Medical Genetics, Life Science Institute University of British Columbia Vancouver British Columbia Canada; ^8^ Present address: School of Biochemistry and Biotechnology, Quaid‐e‐Azam Campus University of the Punjab Lahore Pakistan

**Keywords:** ILEI/FAM3C, inflammation, keratinocyte differentiation, psoriasis, uPA/PLAU, Skin

## Abstract

FAM3C/ILEI is an important cytokine for tumor progression and metastasis. However, its involvement in inflammation remains elusive. Here, we show that ILEI protein is highly expressed in psoriatic lesions. Inducible keratinocyte‐specific ILEI overexpression in mice (*K5‐ILEI* ^*ind*^) recapitulates many aspects of psoriasis following TPA challenge, primarily manifested by impaired epidermal differentiation and increased neutrophil recruitment. Mechanistically, ILEI triggers Erk and Akt signaling, which then activates STAT3 via Ser727 phosphorylation. Keratinocyte‐specific ILEI deletion ameliorates TPA‐induced skin inflammation. A transcriptomic ILEI signature obtained from the *K5*‐*ILEI* ^*ind*^ model shows enrichment in several signaling pathways also found in psoriasis and identifies urokinase as a targetable enzyme to counteract ILEI activity. Pharmacological inhibition of urokinase in TPA‐induced *K5‐ILEI* ^*ind*^ mice results in significant improvement of psoriasiform symptoms by reducing ILEI secretion. The ILEI signature distinguishes psoriasis from healthy skin with uPA ranking among the top “separator” genes. Our study identifies ILEI as a key driver in psoriasis, indicates the relevance of ILEI‐regulated genes for disease manifestation, and shows the clinical impact of ILEI and urokinase as novel potential therapeutic targets in psoriasis.

## Graphical abstract

Analyses of human psoriatic skin and studies in genetically engineered mouse models revealed that FAM3C/ILEI contributes to psoriasis by controlling keratinocyte hyperproliferation, differentiation, and neutrophil recruitment and identifies uPA, a regulator of ILEI secretion, as a druggable target.

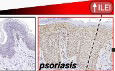

**The paper explained**

**Problem**

Psoriasis is a chronic inflammatory skin disease that affects 2–3% of the worldwide population. Its genetic and environmental triggers are only partially resolved with still unmet need for more therapy options. ILEI is a known regulator of EMT and tumor progression, with an established role in cancer metastasis. Its role in inflammation remains, however elusive, partially due to the strong translational regulation and complex post‐translational control on the activity of the molecule.

**Results**

We find that protein levels of ILEI/FAM3C were increased in the skin of psoriatic patients and correlated with disease severity. In agreement with our clinical observations, *K5‐ILEI* ^*ind*^ mice, our newly generated mouse model with inducible, keratinocyte‐specific ILEI overexpression developed a psoriasis‐like skin phenotype following an inflammatory challenge. A “psoriasis ILEI gene signature” deduced from the transcriptomic profiling of TPA‐treated *K5‐ILEI* ^*ind*^ mice efficiently distinguished psoriasis and healthy skin cohorts. Mechanistically, ILEI shaped an immune response by increasing the expression of inflammatory cytokines and chemokines in keratinocytes in a cell‐autonomous fashion and at the same time inducing a feed‐forward loop on its own post‐translational activation via the plasminogen‐uPA‐uPAR system. Treatment of TPA‐induced *K5‐ILEI* ^*ind*^ mice with a uPA blocker resulted in a significant improvement of psoriasis symptoms by reducing ILEI secretion.

**Impact**

Our study provides a new conceptual insight into the role of ILEI in chronic inflammatory conditions, identifying this cytokine and its secretory regulator, uPA as novel therapeutic targets and thereby offering a new perspective for the treatment of psoriasis. In addition, the paper broadens the current view on ILEI's contribution in cancer, so far linked only to tumor‐intrinsic molecular processes.

## Introduction

Psoriasis is a chronic inflammatory skin disease that affects 2–3% of the people worldwide causing scaly, thickened, erythematous plaques on the skin and nails (Christophers, 2001). The inflammation associated with psoriasis is not confined to skin. Psoriatic arthritis occurs most frequently in patients with psoriasis with a prevalence ranging from 3 to 40%. Other comorbidities, such as cardio‐metabolic disorders and chronic colitis, Crohn's disease, chronic kidney disease affect the patients' quality of life (Gelfand *et al*, 2007; Takeshita *et al*, 2017). Thus, psoriasis can be considered as a systemic inflammatory disorder (Calautti *et al*, 2018). The skin disease is characterized by several histopathological changes, including epidermal thickening resulting from excessive keratinocyte proliferation, abnormal keratinocyte differentiation, and accumulation of neutrophils in parakeratotic stratum corneum (Munro's microabscesses). The dermal infiltrate consists of CD8+ T cells (Tc1), CD4+ T‐helper (Th1) cells, Th17, Th22, and Tc22 cells (Nickoloff *et al*, 2007; Nakajima & Sano, 2018). Mouse models, mimicking the psoriasis‐like phenotype, proved to be a valuable tool in providing in‐depth insight into the disease mechanism, putting keratinocytes and immune cells, especially T cells, as disease initiators, into focus (Gudjonsson *et al*, 2007). At the molecular level, cytokines, chemokines, and growth factors, such as TNFα, IL1‐α, IL6, CXCL1, and keratinocyte growth factor (KGF) produced as a result of a tight cross talk between keratinocytes, fibroblasts, and immune cells are considered to participate in disease manifestation (Wagner *et al*, 2010; Nakajima & Sano, 2018).

The transcription factor STAT3, a member of the family of signal transducers and activators of transcription (STAT), has emerged as one of the key players in the pathogenesis of psoriasis (Calautti *et al*, 2018). *Stat3* has been identified as a genetic susceptibility locus in human psoriasis (Ellinghaus *et al*, 2012; Tsoi *et al*, 2012). Transgenic mice overexpressing a constitutively active form of STAT3 in basal keratinocytes (*K*5*.Stat3C*) develop a mild, psoriasis‐like phenotype that aggravates in response to wounding or 12‐O‐tetradecanoylphorbol‐13‐acetate (TPA) treatment (Sano *et al*, 2005). STAT3 can be activated by several extracellular stimuli (Akira, 1997; Darnell, 1997; Boccaccio *et al*, 1998; Sano *et al*, 1999). Activation induces STAT3 homo‐dimerization and nuclear translocation dependent on tyrosine phosphorylation (Yu *et al*, 2009). Serine phosphorylation of STAT3 modulates its function by maximizing or modifying the spectra of target gene activation and can also induce additional non‐nuclear activities (Wen *et al*, 1995; Andres *et al*, 2013; Balic *et al*, 2020).

ILEI, encoded by the *FAM3C* gene, is a member of the Family with Sequence Similarity 3 (FAM3) cytokine family (Zhu *et al*, 2002). ILEI has been described as an important cytokine for invasion, epithelial‐mesenchymal transition (EMT), and cancer metastasis (Waerner *et al*, 2006; Gao *et al*, 2014; Halberg *et al*, 2016; Schmidt *et al*, 2021). ILEI expression is controlled by TGFβ on the translational level making it difficult to recapitulate its prognostic potential (Chaudhury *et al*, 2010a, 2010b). Additional layers of ILEI activity are controlled by dimerization, with dimers being the active form that induces EMT and metastasis (Jansson *et al*, 2017; Kral *et al*, 2017) and secretion and proteolytic maturation, both regulated via the plasminogen‐uPA‐uPAR system (Csiszar *et al*, 2014). uPA and ILEI have been described to accumulate in the same prometastatic secretory vesicles in lung cancer cells, thus generating an autocrine feed‐forward loop on ILEI secretion and activity (Tan *et al*, 2021).

ILEI signaling still remains elusive. STAT3 has been reported to influence ILEI signaling in several contexts. Binding of ILEI to the LIF receptor (LIFR) activates STAT3 and leads to EMT and stem cell formation in a breast cancer model (Woosley *et al*, 2019). In hepatocellular cancer, ILEI overexpression‐driven EMT acts through mechanisms involving PDGFR/β‐catenin and PDGFR/STAT3 signaling (Lahsnig *et al*, 2009). ILEI has also been linked to other pathways: (i) the heat shock factor 1‐Calmodulin‐Akt signaling axis to regulate glucose and lipid metabolism (Chen *et al*, 2017) and (ii) the Ras‐MAPK pathway for EMT induction in breast cancer cell lines (Waerner *et al*, 2006). Recently, the ILEI/LIFR complex is also reported to induce EMT by activating Akt and Erk pathways in renal interstitial fibrosis (Zhou *et al*, 2022).

Although previous studies have linked ILEI to cancer progression, the involvement of ILEI in inflammation is not understood. Here, we demonstrate the role and clinical relevance of ILEI in skin inflammation with a focus on psoriasis. We found that ILEI is prominently overexpressed in human psoriasis. We used a new, skin‐specific ILEI transgenic mouse model (*K5*‐*ILEI* ^*ind*^) that recapitulated a psoriasiform phenotype after TPA treatment. Mechanistically, we show that ILEI acts cell‐autonomously on keratinocytes via enhanced STAT3 activation through increased Ser727 phosphorylation, which was mediated by elevated Erk and Akt signaling. Genetic deletion of ILEI abrogated TPA‐induced inflammation identifying ILEI as important driver of the psoriasiform phenotype. From the *K5*‐*ILEI* ^*ind*^ mouse model, we identified a transcriptomic ILEI signature that included upregulation of uPA. Pharmacological inhibition of ILEI secretion in overexpressing mice by the urokinase inhibitor UK371804 ameliorated the psoriasiform phenotype. The ILEI signature powerfully separated psoriasis patients from healthy controls, uPA being determined as one of the strongest “separator” genes. These indicate the clinical relevance of ILEI and urokinase as novel potential therapeutic targets in psoriasis.

## Results

### ILEI protein is increased in human psoriatic skin

**Figure 1:**
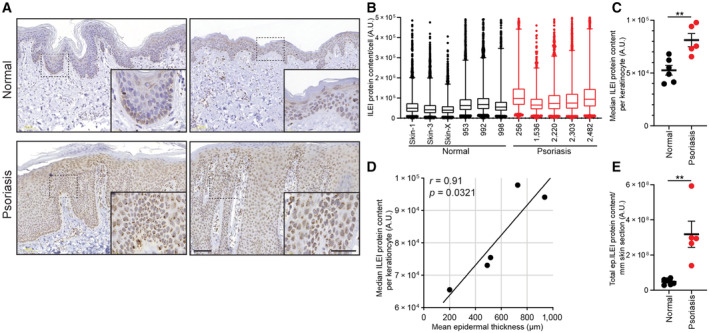
ILEI protein expression is elevated in human psoriasis Representative images of ILEI immunohistochemistry on healthy (upper panels) and psoriatic (lower panels) skin sections, scale bar 100 μm. Inlets show a magnification of the marked regions, scale bar 50 μm.Distribution of ILEI protein content per keratinocyte (*n* = 5,000–15,000 cells per skin sample) in healthy and psoriatic skin. Box‐and‐whiskers plot: Central band shows median, box extends from the 25^th^ to 75^th^ percentiles, and whiskers go from the 1^st^ to the 99^th^ percentiles.Median ILEI content of keratinocytes per person shown as mean ± SEM of healthy (*n* = 6) and psoriasis (*n* = 5) conditions.Pearson correlation plot of median ILEI content per keratinocyte and epidermal thickness of psoriatic skin (*n* = 5).Mean ± SEM of epidermal ILEI load per mm skin section in healthy (*n* = 6) and psoriasis (*n* = 5) condition. A.U., arbitrary unit. Data information: (C, E) Statistical significance was determined by Student's *t*‐test and marked with asterisks (**P < 0.01). Source data are available online for this figure.

**Figure EV1:**
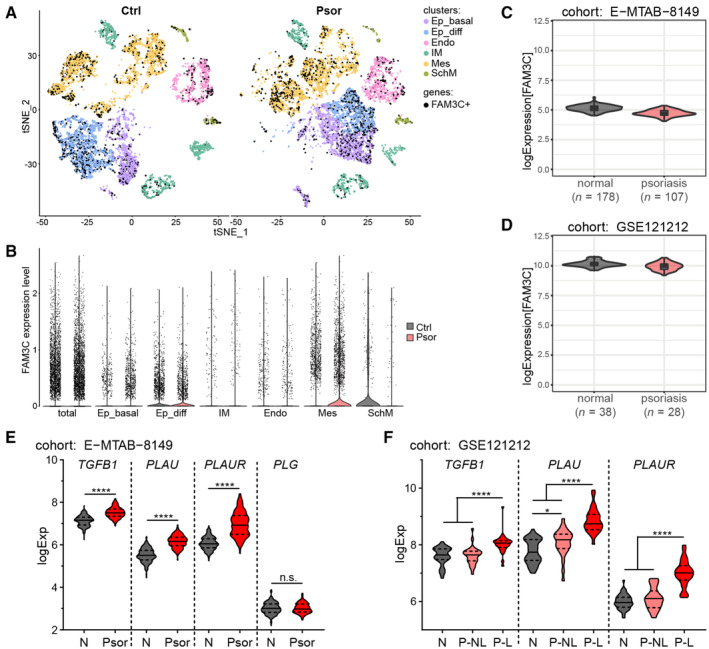
Transcripts of positive regulators of FAM3C/ILEI translation and secretion, but not FAM3C/ILEI mRNA are upregulated in psoriatic patients A, B
scRNA‐Seq human full‐thickness skin dataset GSEE162183 of control (Ctrl) and Psoriasis (Psor) patients presented (A) in t‐SNE overlay visualization overlayed with cells expressing FAM3C and (B) in violin plots showing expression levels of FAM3C in respective cell clusters. Ep_basal, epidermis basal subcluster; Ep_diff, epidermis differentiated subcluster; IM, immune cluster; Endo, endothelial cluster; Mes, mesenchymal cluster; SchM, Schwann/Melanocyte‐like cluster.C, D
log2fold *FAM3C* mRNA expression levels in normal skin and psoriatic lesions of the datasets (C) MTAB‐8149 (*n* = 285) and (D) GSE121212 (*n* = 66). Violin plot overlayed with Box‐and‐whiskers plot: Central band shows median, box extends from the 25^th^ to 75^th^ percentiles and whiskers go from the smallest (min) to the largest (max) value. Density curves of the violin plot correspond to the approximate frequency of data points in each region.E, F
log2fold mRNA expression levels of (E) *TGFB1*, *PLAU*, *PLAUR* and *PLG* in normal skin (N) (*n* = 178) and psoriatic lesions (Psor; *n* = 107) of the RNA sequencing dataset MTAB‐8149 and (F) *TGFB1*, *PLAU* and *PLAUR* in normal skin (N) (*n* = 38) and in nonlesional (P‐NL) (*n* = 27) and lesional (P–L) skin (*n* = 28) of psoriasis patients of the microarray dataset GSE121212. Violin plots: central band shows median, dashed lines label the 25^th^ and 75^th^ percentiles, and density curves correspond to the approximate frequency of data points in each region extending from the smallest (min) to the largest (max) value. Data information: statistical significance was determined by (E) student's *t*‐test and (F) one‐way ANOVA with Tukey multiple comparison test and marked with asterisks (*P < 0.05; ****P < 0.0001).

**Figure EV2:**
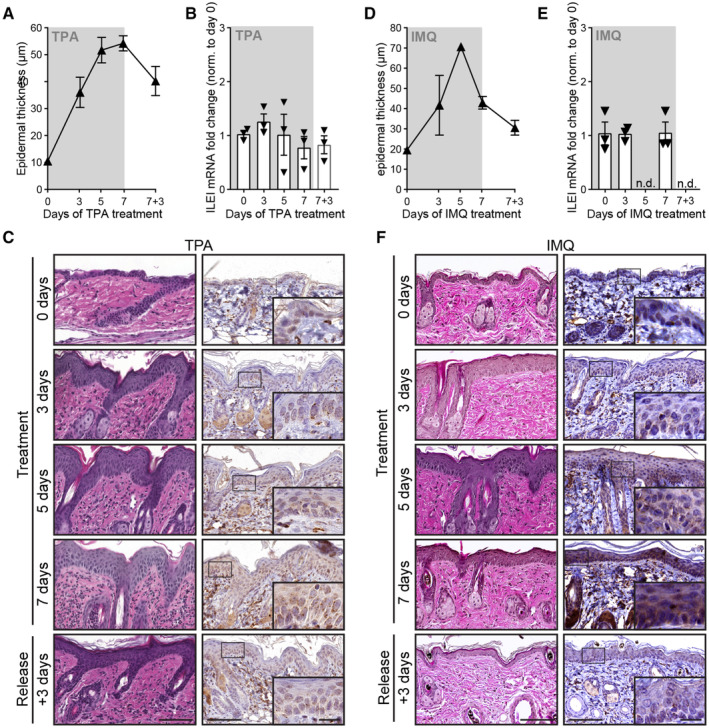
Chemically triggered inflammatory conditions increase ILEI protein, but not mRNA levels in mouse skin A–F
Analysis on back skin of wild‐type mice treated for 0, 3, 5, and 7 days with (A–C) TPA or (D–F) IMQ followed by an additional withdrawal for 3 days (*n* = 3 for each time point and treatment; 2 independent experiments). Timely changes of (A,D) mean epidermal thickness ± SEM and (B, E) mean ILEI mRNA expression fold change ±SEM. Epidermal thickness was quantified from (C, F left panels) hematoxylin–eosin stained thin sections of back skin. mRNA expression was normalized to untreated skin (Day 0). (C, F right panels) Representative images of ILEI immunohistochemistry on thin sections of back skin. Scale bar 100 μm. Inlets show a magnification of the marked regions, scale bar 20 μm. IMQ, imiquimod; nd, not done.

In order to explore a possible role of ILEI in inflammatory skin disease, we tested ILEI expression in psoriasis. We performed ILEI immunohistochemistry (IHC) on skin sections of six healthy donors and five psoriatic patients and determined ILEI protein content in keratinocytes by IHC chromogene intensity quantification after cellular stratification. While in healthy skin ILEI protein was often restricted to basal keratinocytes, in psoriatic skin a more intense and homogenously distributed signal was observed throughout the whole epidermis (Fig 1A). Accordingly, keratinocytes of psoriatic skin showed higher ILEI contents (Fig 1B) and median ILEI protein levels of keratinocytes were significantly increased in psoriatic patients (Fig 1C). Furthermore, ILEI intensity in keratinocytes showed a positive correlation to epidermal thickening (Fig 1D). As a consequence, total ILEI load of the epidermis was also significantly increased in psoriasis patients as compared to healthy individuals (Fig 1E). However, ILEI mRNA levels were unchanged when analyzed both in psoriatic keratinocytes at single‐cell resolution (Fig EV1A and B) and in total skin (Fig EV1C and D) using published human psoriasis scRNA‐Seq (Gao *et al*, 2021) and bulk RNA‐Seq datasets, respectively (Fyhrquist *et al*, 2019; Tsoi *et al*, 2019). Known inducers of ILEI translation and secretory activity, such as TGFβ, uPA, and uPAR, showed at the same time significantly elevated expression in the same datasets (Fig EV1E and F), being in accordance with earlier reports (Han *et al*, 2010; Rubina *et al*, 2017) and indicating an upregulation of the ILEI translational and secretory machinery in psoriasis. Similar observations were made in mouse skin upon short‐term treatment with imiquimod (IMQ) or TPA that cause psoriasis‐like skin inflammation. ILEI protein levels increased over time and decreased upon termination of treatment. ILEI transcript levels remained, however, unchanged (Fig EV2A–F). These data show that ILEI expression is upregulated in psoriasis, exclusively at the protein level, and that upregulation correlates with disease severity.

**Figure 2:**
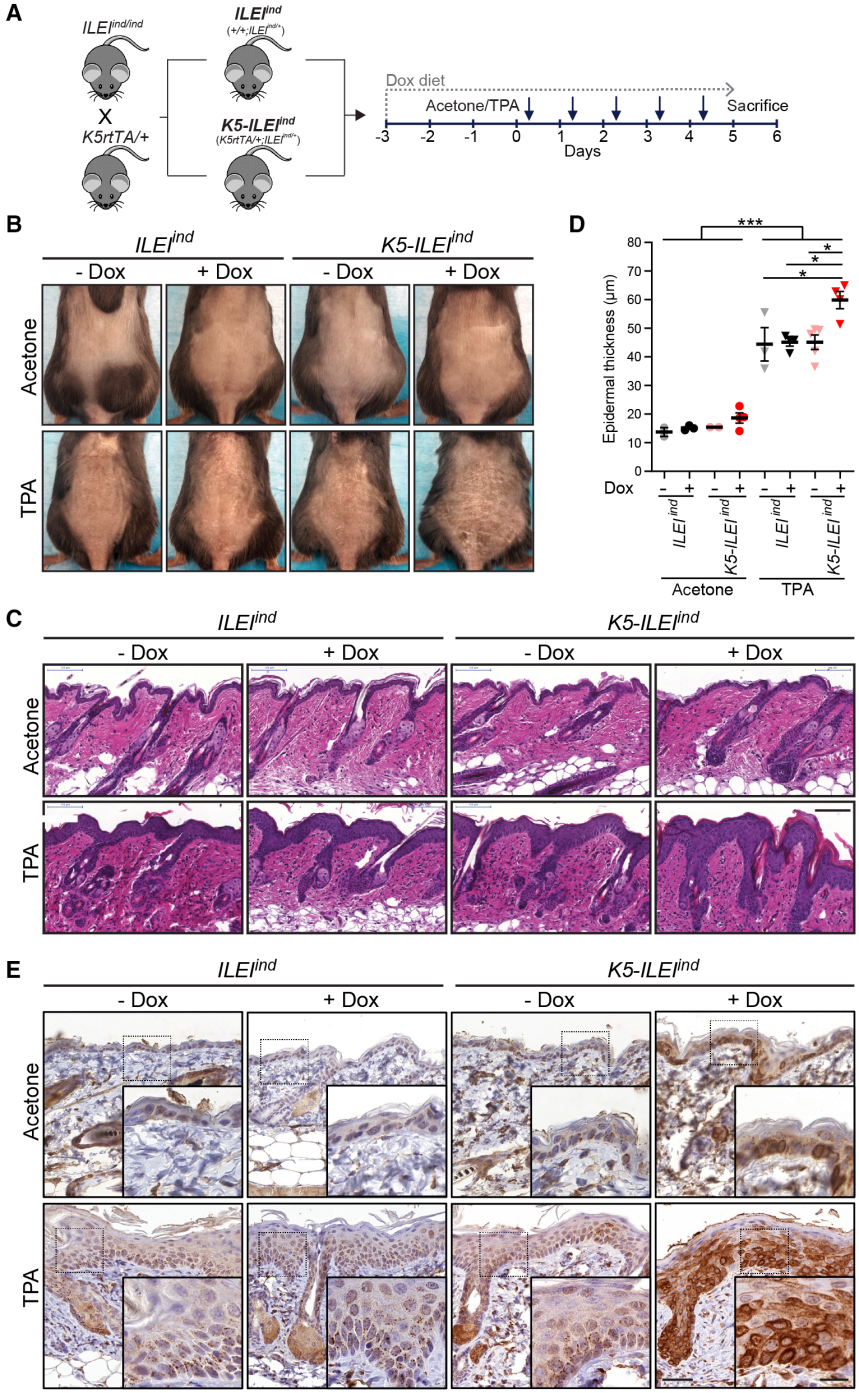
ILEI overexpression in keratinocytes exacerbates inflammation‐triggered epidermal thickening in mice A
Schematic view of the *K5‐ILEI* ^*ind*^ psoriasis mouse model. Tet‐ON inducible ILEI transgenic mice (*ILEI* ^*ind*^) were crossed with mice expressing the reverse tet‐responsive transactivator in epidermal basal keratinocytes under the control of the bovine Keratin 5 promoter (*K5rtTA*). TPA treatment was performed for 5 days. Three days prior treatment start mice were switched to doxycycline diet. Dual transgenic *K5‐ILEI* ^*ind*^ mice with normal diet, *ILEI* ^*ind*^ mice with normal and doxycycline diet and acetone treatment were used as genetic, diet, and treatment controls, respectively.B–E
(B) Macroscopic appearance, (C) hematoxylin–eosin staining with scale bar of 50 μm and (D) mean epidermal thickness ± SEM of the back skin of *ILEI* ^*ind*^ and *K5‐ILEI* ^*ind*^ mice kept on normal or doxycycline diet and treated with acetone or TPA for 5 days (*n* = 2–5; 3 independent experiments). Statistical significance was determined by one‐way ANOVA with Tukey multiple comparison test and marked with asterisks (*P < 0.05; ***P < 0.001). (E) Representative images of ILEI immunohistochemistry on sections of back skin of *ILEI* ^*ind*^ and *K5‐ILEI* ^*ind*^ mice kept on normal or doxycycline diet upon 5 days of treatment with acetone or TPA. Scale bar 50 μm. Inlets show a magnification of the marked regions, scale bar 20 μm. Source data are available online for this figure.

### ILEI overexpression in keratinocytes increases epidermal thickening upon inflammatory trigger in mice

In order to assess whether elevated ILEI expression contributes to psoriasis‐like phenotypes, we generated mice in which overexpression of ILEI can be induced specifically in keratinocytes (*K5‐ILEI* ^*ind*^ *)*. ILEI overexpression alone did not lead to an aberrant skin phenotype or reduce fitness even when mice were kept on doxycycline diet for over 200 days and analyzed for changes in body weight, skin barrier integrity, and epidermal thickness (Appendix Fig S1A–D). Next, we applied TPA treatment after transgene induction (Fig 2A). As expected, all control mice developed scaly skin upon TPA treatment. In ILEI‐overexpressing mice, this phenotype was, however, more pronounced (Fig 2B). Consistently, ILEI overexpression in keratinocytes leads to a significant increase in epidermal thickening upon TPA treatment (Fig 2C and D). No difference was observed in acetone‐treated control mice (Fig 2B–D). Immunohistochemical (IHC) analysis on both TPA‐ and acetone‐treated skin confirmed high ILEI protein expression in transgenic mice on doxycycline diet (Fig 2E). These data demonstrate that high epidermal ILEI levels caused increased epidermal thickening upon an inflammatory trigger.

To test whether the effect of ILEI overexpression was specific to TPA only, we treated mice for 5 days with imiquimod (IMQ). Similar to the TPA‐induced phenotype, we observed an increase in skin thickening in ILEI‐overexpressing mice upon IMQ treatment (Appendix Fig S1E–G) compared with control mice. These results show that ILEI protein levels in the epidermis dictate the outcome of severity upon an inflammatory trigger.

### ILEI overexpression triggers hyperproliferation and impairs differentiation of keratinocytes in inflammatory conditions in a cell‐intrinsic manner

**Figure 3:**
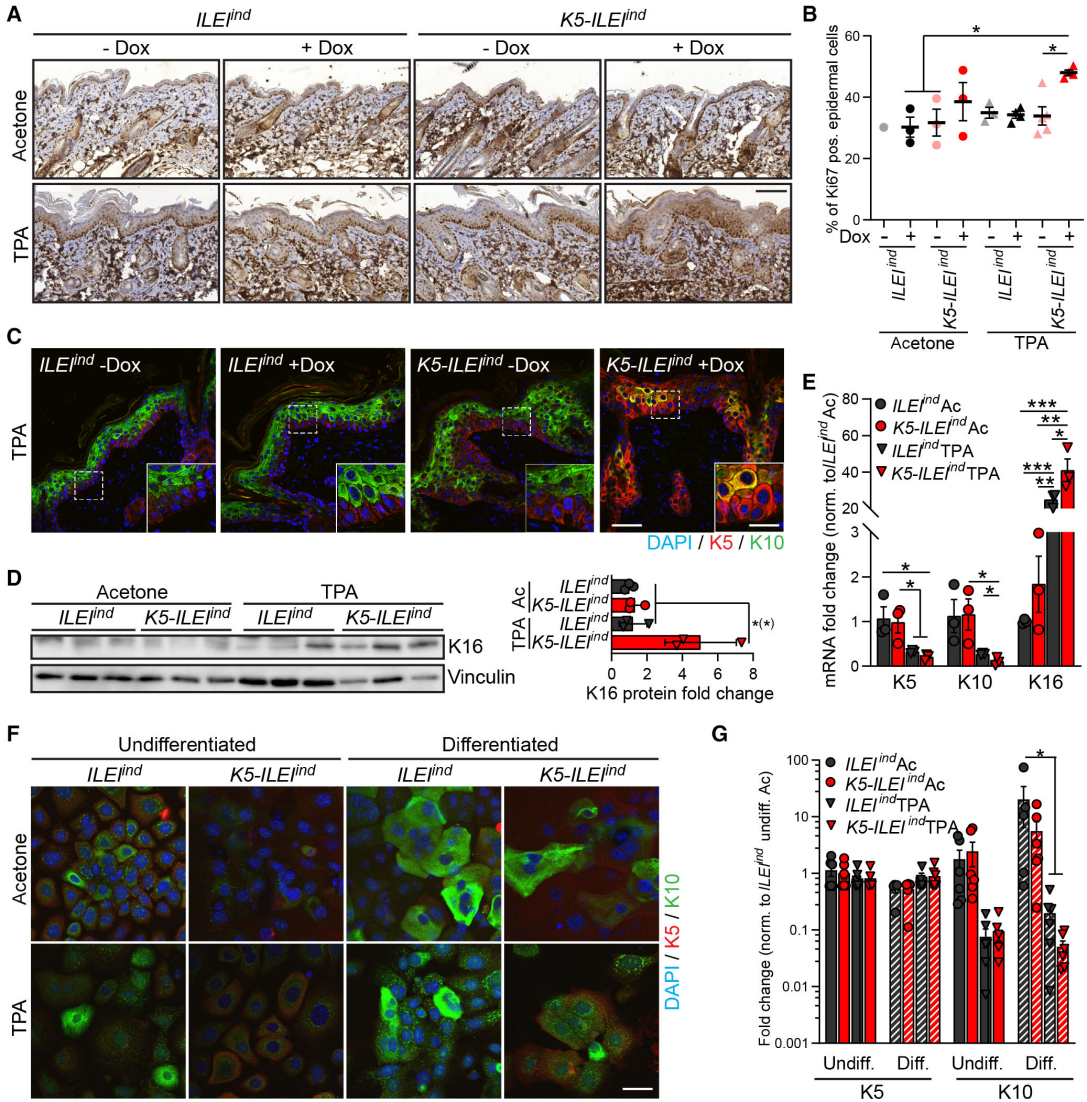
ILEI overexpression leads to increased hyperproliferation and impaired differentiation of mouse epidermis upon TPA treatment Representative images of Ki67 immunohistochemistry on back skin sections of *K5‐ILEI* ^*ind*^ and *ILEI* ^*ind*^ mice kept on normal or doxycycline diet and treated with acetone or TPA for 5 days. Scale bar: 100 μm.Percentage of Ki67 positive cells in the epidermis shown as mean ± SEM and quantified on samples described in panel A (*n* = 1–5; 3 independent experiments).Immunofluorescence for Keratin 5 (K5; red) and Keratin 10 (K10; green) expression on TPA‐treated skin sections of mice described in panel A. Nuclei were counterstained with DAPI (blue). Scale bar: 50 μm. Inlets show a magnification of the marked regions. Scale bar: 20 μm.Keratin 16 (K16) western blot analysis (left) and quantification (right) of proteins extracted from back skin of *ILEI* ^*ind*^ and *K5‐ILEI* ^*ind*^ mice kept on doxycycline and treated with acetone or TPA for 5 days (*n* = 3). Vinculin was used as loading control.Mean mRNA expression ±SEM of *K5*, *K10*, *and K16* in keratinocytes enriched for the interfollicular epithelium freshly sorted from acetone and TPA‐treated back skin of mice described in panel D (*n* = 3).Immunofluorescence for Keratin 5 (K5; red) and Keratin 10 (K10; green) on *in vitro* acetone and TPA‐treated, doxycycline‐induced, primary keratinocytes with or without calcium‐induced differentiation (72 h) isolated from *ILEI* ^*ind*^ and *K5‐ILEI* ^*ind*^ mice. Nuclei are counterstained with DAPI (blue); scale bar, 20 μm.Mean fold change in mRNA expression ±SEM of *K5* and *K10* in primary keratinocytes described in panel F (*n* = 5–6; 2 independent experiments). Data information: Statistical significance was determined by two‐way ANOVA (G), one‐way ANOVA (B, D und K16 in E) and Student's *t*‐test (K5 and K10 in E) at ANOVA with Tukey multiple comparison test and marked with asterisks (*P < 0.05; **P < 0.01; ***P < 0.001). Source data are available online for this figure.

Next, we assessed whether the increase in epidermal thickening caused by ILEI overexpression was a consequence of an increase in inflammation‐induced proliferation rate. *K5‐ILEI* ^*ind*^ mice showed a significant increase in suprabasal expression of Ki67, indicating an elevated proliferative capacity upon induction of ILEI expression (Fig 3A and B). This was confirmed in *ex vivo* cultures of primary keratinocytes isolated from TPA‐treated skin of ILEI‐overexpressing and control mice (Appendix Fig S2A and B). ILEI overexpression also impaired epidermal differentiation, as detected by the incomplete expression of the epidermal differentiation marker Keratin 10 (K10; Blanpain & Fuchs, 2006) in the epidermis of TPA‐treated murine skin sections (Fig 3C). In addition, TPA treatment of ILEI‐overexpressing skin lead to elevated expression of Keratin 16 (K16), a pathological marker, known to be upregulated also in psoriasis (Leigh *et al*, 1995; Zhang *et al*, 2019) as analyzed in total skin protein extracts (Fig 3D) and by qPCR on freshly sorted primary keratinocytes (Fig 3E). Keratin 5 (K5), a marker expressed in the basal layer of keratinocytes, did not show ILEI‐dependent changes in its transcript levels (Fig 3E). Next, we tested whether ILEI acted cell‐autonomously on keratinocyte differentiation. *Ex vivo* cultures of primary keratinocytes isolated from ILEI‐overexpressing mice were impaired in calcium‐induced differentiation in the presence of TPA, as indicated by a reduced K10 expression both at protein and mRNA levels (Fig 3F and G). Impaired differentiation was also shown by reduced loricrin expression, a marker of advanced differentiation, in cultured keratinocytes (Appendix Fig S2C). Together, these data showed that ILEI overexpression results in increased hyperproliferation and counteracts keratinocyte differentiation in a cell‐autonomous manner after an inflammatory stimulus.

### ILEI shapes the inflammatory immune response by increasing the expression of cytokines and chemokines in keratinocytes in a cell‐autonomous fashion

**Figure 4:**
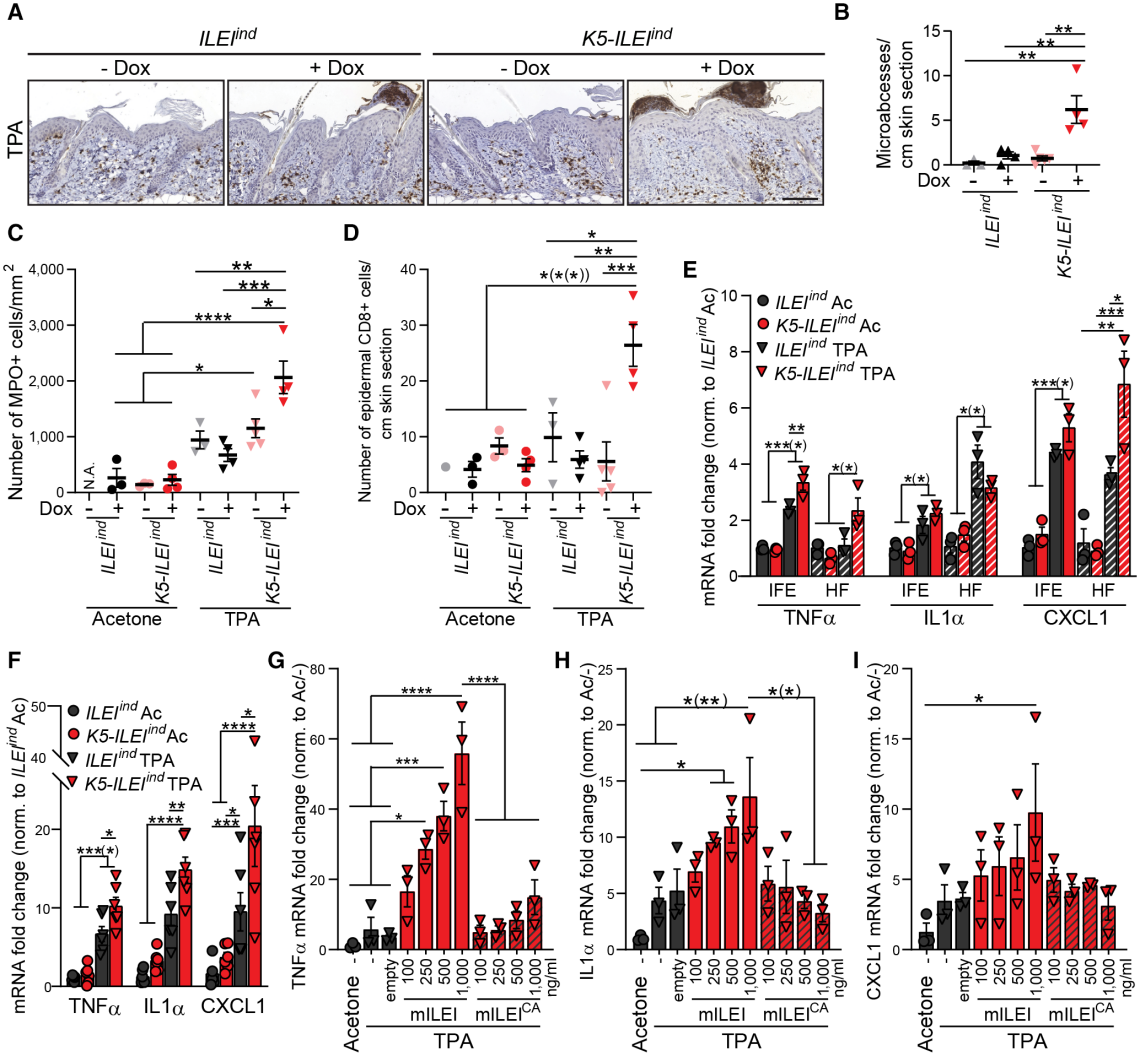
ILEI enhances the inflammatory immune response by cell‐intrinsic upregulation of pro‐inflammatory factors in keratinocytes A
Representative images of MPO‐positive microabscesses stained on sections of acetone and TPA‐treated back skin of *ILEI* ^*ind*^ and *K5‐ILEI* ^*ind*^ mice kept on normal or doxycycline diet. Scale bar: 100 μm.B–D
Mean number ± SEM of (B) neutrophil‐positive microabscesses/cm skin section, (C) neutrophils (MPO+ cells)/mm^2^ epidermal‐dermal area and (D) epidermal CD8+ T cells/cm skin section of mice described in A (*n* = 3–5 (B and C), *n* = 1–5 (D); 3 independent experiments). N.A., not analyzed.E, F
Mean fold change ±SEM in mRNA expression of *Tnfa*, *Il1α*, *and Cxcl1* (E) in freshly sorted keratinocytes enriched for the interfollicular epithelium (IFE) and hair follicles (HF) from acetone and TPA‐treated back skin and (F) in primary keratinocyte cultures isolated from *ILEI* ^*ind*^ and *K5‐ILEI* ^*ind*^ mice supplemented with doxycycline and treated with acetone or TPA for 96 h (*n* = 3 (E), *n* = 6 (F); F, 2 independent experiments).G–I
Mean fold change ±SEM in mRNA expression of (G) *Tnfa*, (H) *Il1α*, *and* (I) *Cxcl1* in primary keratinocytes treated with acetone and TPA and with increasing concentrations (100, 250, 500, 1,000 ng/ml) of murine recombinant ILEI (mILEI) or dimerization‐disabled ILEI (mILEI^CA^) or with empty vector (empty) for 72 h (*n* = 3, standing for independent keratinocyte cultures from three mice). Data information: In (B–I), statistical significance was determined by one‐way ANOVA with Tukey multiple comparison test and marked with asterisks (*P < 0.05; **P < 0.01; ***P < 0.001; ****P < 0.0001). If significance levels were different for the pairwise comparisons with combined marking, asterisks, valid only for a subset of the pairs were put into brackets. Source data are available online for this figure.

Skin inflammation provokes extensive remodeling of the skin immune landscape. Thus, we evaluated whether ILEI had an influence on the composition of inflammatory cells in the skin in TPA‐treated *K5‐ILEI* ^*ind*^ mice. ILEI overexpression resulted in both, a significant increase in the number of neutrophil‐positive microabscesses and in the abundance of neutrophils recruited to the dermis upon TPA trigger (Figs 4A–C; Appendix Fig S5A). Furthermore, the number of CD8+ T cells in the epidermis was significantly higher (Fig 4D; Appendix Fig S5B), and plasmacytoid dendritic cells (pDC) showed also increased accumulation in ILEI‐overexpressing skin after TPA stimulus (Appendix Fig S3C). Other dendritic cell subpopulations (cDC1, cDC2) as well as mast cells, macrophages, CD4+ and γδT cells remained unchanged upon ILEI overexpression (Appendix Figs S3D and E, and S4A–G). In accordance, IL‐17A and CCL2 protein levels, known to be produced primarily by γδT cells via IL23‐driven cDC2 stimulus and involved in monocyte recruitment upon inflammation (Dong, 2006; Cai *et al*, 2011; Wang *et al*, 2019a; Novoszel *et al*, 2021), respectively, were not affected by ILEI expression in TPA‐treated skin (Appendix Fig S4H and I). From this, we concluded that the major ILEI‐regulated inflammatory route was via neutrophils and not via the Th17 axis in our model.

Neutrophil recruitment in psoriasis is regulated by TNFα, IL1α, and CXCL1; factors produced mainly by keratinocytes (Uribe‐Herranz *et al*, 2013; Ogawa *et al*, 2018). Thus, we tested whether ILEI‐overexpressing keratinocytes show elevated levels of these factors by analyzing their expression in freshly sorted keratinocytes enriched for interfollicular (IFE) and hair‐follicle (HF) epidermal cells from vehicle and TPA‐treated *K5‐ILEI* ^*ind*^ mouse skin. As expected, these factors were upregulated upon TPA treatment (Fig 4E). More importantly, TNFα and CXCL1 showed an additional significant upregulation upon ILEI overexpression, with even more pronounced changes in the HF population (Fig 4E). Several growth factors and cytokines, for example, EGFR ligands, IL17C and the IL36 family, have been described to be overexpressed in psoriatic keratinocytes with important roles in disease manifestation, in both human and mouse models, among others by upregulating TNFα production (Johnston *et al*, 2013; Wang *et al*, 2019b; Sachen *et al*, 2022). Thus, we analyzed their expression in the freshly sorted keratinocytes of TPA‐treated ILEI‐overexpressing skin (Appendix Fig S4J). While many of them showed TPA‐driven upregulation, confirming their role in skin inflammation, none of them were increased in an ILEI‐dependent manner, suggesting that ILEI did not act via the upregulation of these factors.

*Ex vivo* TPA stimulation of primary keratinocytes from control and ILEI‐overexpressing murine skin was used to address the cell‐autonomous action of ILEI. We observed a significant increase in the expression of all three factors, in an ILEI‐dependent manner (Fig 4F). To further validate the cell‐intrinsic effect of ILEI on cytokine and chemokine production of keratinocytes, recombinant murine ILEI was used in increasing concentrations to treat primary keratinocytes after TPA stimulus. Both, a dimeric (mILEI) and a dimerization‐mutant, monomeric form (mILEI^CA^) were used (Kral *et al*, 2017). Expression analysis of the neutrophil‐recruiting factors showed that dimeric ILEI was potent to induce a dose‐dependent significant upregulation of TNFα, IL1α, and CXCL1 transcripts (Fig 4G–I) as observed *in vivo* and *ex vivo* upon ILEI overexpression.

These findings demonstrate that a cell‐autonomous action of ILEI dimers in keratinocytes is critical for the production of neutrophil‐recruiting soluble factors (TNFα, IL1α, and CXCL1), which may explain the increased recruitment of neutrophils observed *in vivo*.

### ILEI acts by enhancing STAT3 transcriptional activity via Erk and Akt‐mediated elevated Ser727 phosphorylation

**Figure 5:**
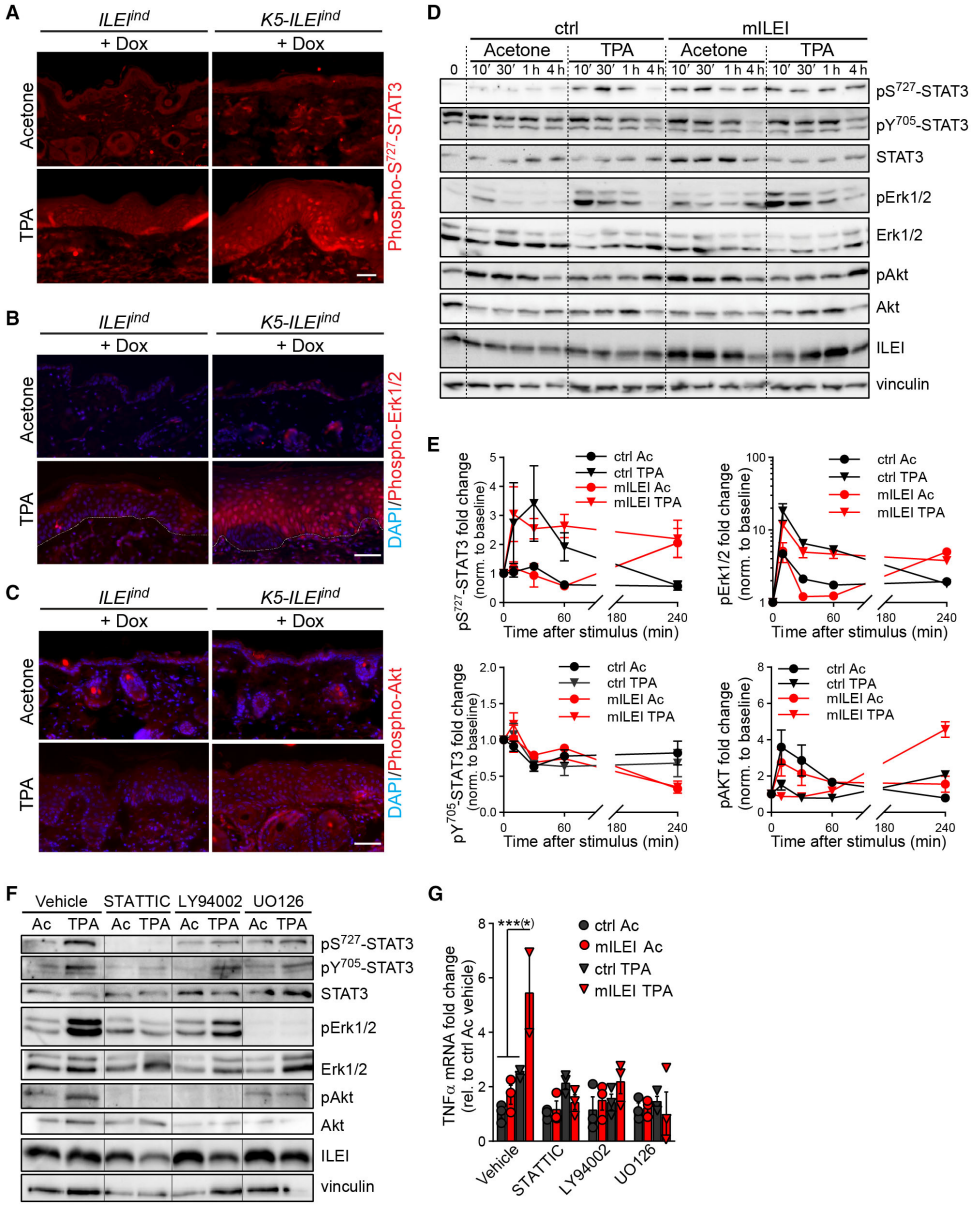
ILEI upregulates Erk and Akt activities that increase STAT3 activation via Ser727 phosphorylation A–C
Representative images of (A) Phospho‐STAT3 (Tyr727), (B) Phospho‐Erk1/2 and (C) Phospho‐Akt immunofluorescence on thin sections of acetone and TPA‐treated back skin of *ILEI* ^*ind*^ and *K5‐ILEI* ^*ind*^ mice kept on doxycycline diet. Scale bar, 50 μm.D
Representative western blot analysis of STAT3, Akt and Erk1/2 phosphorylation levels in primary wild‐type murine keratinocytes harvested after 0, 10, 30 min, 1 and 4 h upon acetone and TPA and simultaneous control (ctrl) and recombinant wild‐type murine ILEI (mILEI) treatments. Vinculin was used as loading control.E
Mean STAT3 (Ser727 and Tyr705), Akt, and Erk1/2 phosphorylation levels ±SEM over time in primary keratinocytes upon TPA and recombinant ILEI treatment as described in panel A (*n* = 3, standing for independent keratinocyte cultures, each a pool of isolates from two mice).F
Western blot analysis of STAT3 (Ser727 and Tyr705), Akt, and Erk1/2 phosphorylation levels in primary Dox‐induced *K5‐ILEI* ^*ind*^ keratinocytes treated with acetone or TPA for 4 h in the presence of the inhibitors STATTIC (10 μM), LY92004 (10 μM) and UO126 (10 μM). DMSO was used as vehicle control. Vinculin was used as loading control. Lanes are from noncontinuous parts of the same gel.G
Mean fold change ±SEM in mRNA expression of TNFα in primary wild‐type murine keratinocytes harvested after 7 h upon acetone and TPA and simultaneous control (ctrl) and recombinant wild‐type murine ILEI (mILEI) treatments in the presence of the inhibitors STATTIC (10 μM), LY92004 (10 μM), and UO126 (10 μM). DMSO was used as vehicle control (*n* = 2–3, each an independent pool of two mice). Statistical significance was determined by two‐way ANOVA with Tukey multiple comparison test and marked with asterisks (*P < 0.05; ***P < 0.001). If significance levels were different for the pairwise comparisons with combined marking, asterisks, valid only for a subset of the pairs were put into brackets. Source data are available online for this figure.

ILEI has been shown to act via the LIFR/ STAT3 axis to mediate EMT in breast cancer stem cells (Woosley *et al*, 2019). Furthermore, several reports link ILEI to Akt and Erk signaling in various contexts (Waerner *et al*, 2006; Yang *et al*, 2019; Zhou *et al*, 2022). Thus, in order to evaluate the signaling mechanism through which ILEI mediated its effects in the epidermis, we analyzed the activation levels of these signaling molecules *in vivo*. ILEI overexpression in the skin led to an elevated phosphorylation of STAT3‐Ser727, Erk1/2, and Akt upon TPA treatment, whereas STAT3 tyrosine phosphorylation remained unchanged, as evaluated by immunofluorescence and immunohistochemistry of control and ILEI‐overexpressing mice (Fig 5A–C; Appendix Fig S5A and B). To evaluate the timely resolution of these changes, the phosphorylation kinetics of STAT3, Erk1/2, and Akt signaling molecules was analyzed in primary keratinocyte cultures upon treatment with recombinant ILEI dimers and acetone or TPA (Fig 5D and E). TPA stimuli had robust effect on STAT3 serine phosphorylation, Erk activation, and downregulation of Akt phosphorylation; the high baseline levels of tyrosine‐phosphorylated STAT3 were not affected. Importantly, co‐treatment with ILEI did not show any specific effects within the first hour after TPA stimuli. (Fig 5D and E). At 4 h, however, ILEI‐dependent differences became detectable. Serine phosphorylation of STAT3 (pSer^727^‐STAT3) showed sustained higher levels in TPA‐ILEI co‐treatment, while levels of tyrosine‐phosphorylated STAT3 (pTyr^705^‐STAT3) decreased more compared with control TPA treatment (Fig 5D and E). Concomitantly, Phospho‐Erk1/2 showed sustained higher levels and Phospho‐Akt proteins reaccumulated to higher levels upon TPA‐ILEI co‐treatment (Fig 5D and E). Of note, recombinant ILEI induced similar changes also in the absence of an inflammatory stimulus. The late effect of ILEI‐specific changes in signaling activities might be indicative for a regulatory feedback loop on these signaling molecules by ILEI rather than a direct activation by ILEI signaling.

Erk and Akt signaling are known to act on serine phosphorylation of STAT3 and thereby enhance/modulate its signaling activity (Wen *et al*, 1995). However, STAT3 signaling can also contribute to elevated Erk and Akt signaling (Gong *et al*, 2015). Thus, we tested the interdependence by selectively inhibiting these pathways in ILEI‐overexpressing keratinocytes upon TPA stimulus (Fig 5F). Treatment with the STAT3 inhibitor STATTIC eliminated both, STAT3 tyrosine and serine phosphorylation, as expected. In addition, it efficiently prevented elevated Akt and Erk phosphorylation that was otherwise observed after TPA trigger (Fig 5F), suggesting that STAT3 activation was a prerequisite of Akt and Erk activation. Both, inhibition of Akt by the PI3K inhibitor LY94002 and Erk by the Mek inhibitor UO126 resulted in a decrease of pSer^727^‐STAT3 to baseline levels without affecting STAT3 tyrosine phosphorylation (Fig 5F), indicating that Akt and Erk might have been responsible for STAT3 serine phosphorylation. The Mek inhibitor, in addition, also blocked Akt activation (Fig 5F), pointing out that Mek might have contributed to STAT3 serine phosphorylation also via PI3K/Akt activation. To verify whether activation of the STAT3, Akt, and Erk/MAPK pathways was important in ILEI function in keratinocytes, the expression of the ILEI‐dependent target gene TNFα was analyzed in primary keratinocytes after treatment with recombinant ILEI and TPA in the presence of the STAT3, PI3K, and Mek inhibitors. Each of the inhibitors prevented ILEI‐dependent upregulation of TNFα expression, PI3K and Mek inhibitors also completely eliminating any TPA‐driven effects (Fig 5G). These data together show that STAT3 activation by tyrosine phosphorylation was essential, but not sufficient to explain ILEI's mechanism of action. STAT3‐dependent activation of PI3K/Akt and Mek/MAPK signaling was additionally required for full ILEI function, most probably by enhancing STAT3 activity via phosphorylation of its Ser727 residue.

### ILEI knockout in keratinocytes ameliorates TPA‐induced skin inflammation

**Figure 6:**
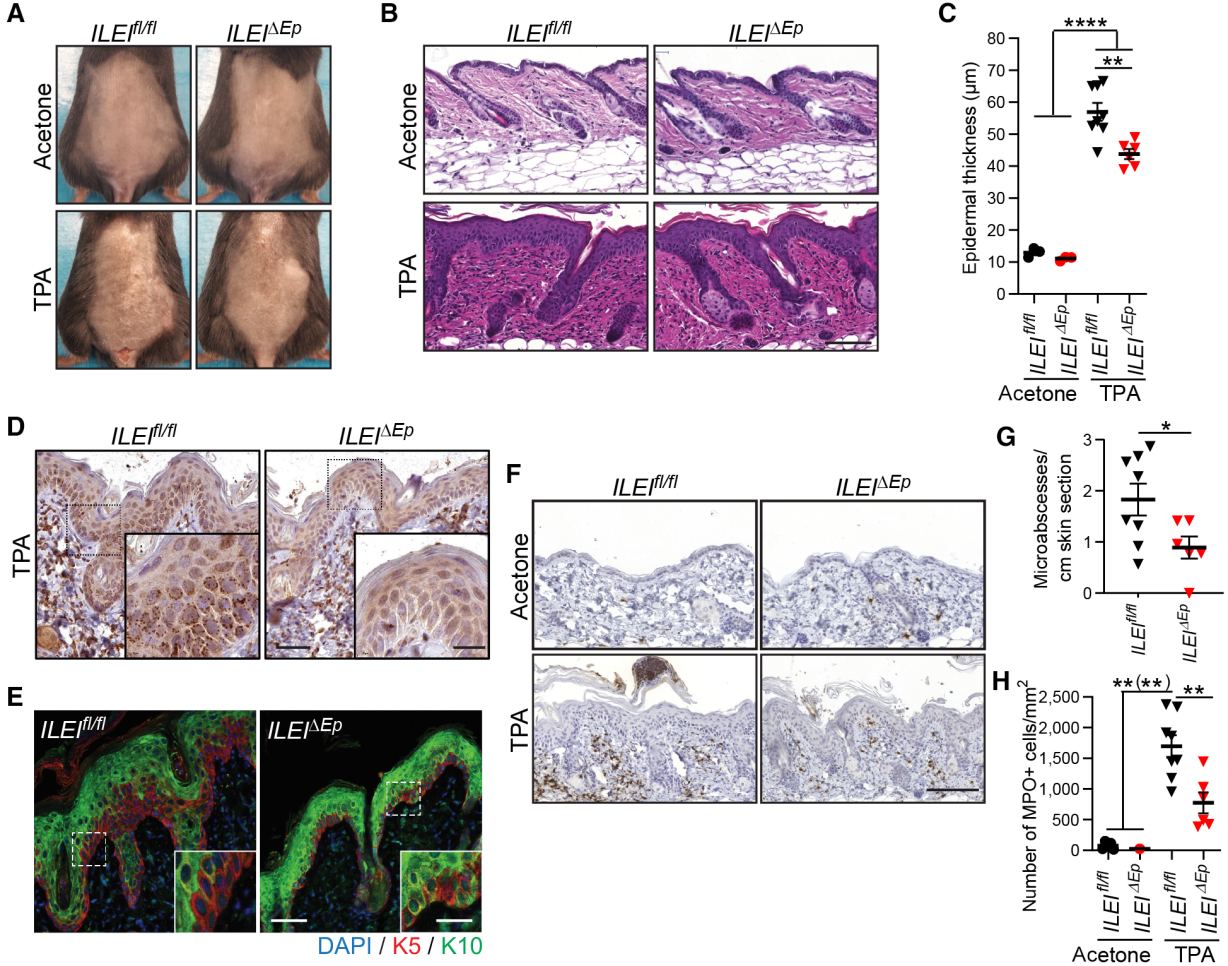
ILEI deletion in the skin ameliorates the psoriasiform phenotype A–C
(A) Macroscopic appearance, (B) hematoxylin–eosin staining with a scale bar of 50 μm and (C) mean epidermal thickness ± SEM of the back skin of *ILEI^fl/fl^* and *ILEI^∆Ep^* mice treated with acetone or TPA for 5 days (*n* = 3–8; 3 independent experiments).D
Representative images of ILEI immunohistochemistry on thin sections of back skin of *ILEI^fl/fl^* and *ILEI^∆Ep^* mice treated with acetone or TPA for 5 days. Scale bar 50 μm. Inlets show a magnification of the marked regions, scale bar 20 μm.E
Immunofluorescence for K5 (red) and K10 (green) expression on TPA‐treated skin sections of *ILEI^fl/fl^* and *ILEI^∆Ep^* mice. Nuclei were counterstained with DAPI (blue). Scale bar, 50 μm. Inlets show a magnification of the marked regions, scale bar 20 μm.F–H
(F) Representative images of MPO+ microabscesses and neutrophils, (G) mean number ± SEM of MPO‐positive microabscesses/cm skin section and (H) mean number of neutrophils (MPO+ cells)/mm^2^ epidermal‐dermal area ± SEM on thin sections of acetone and TPA‐treated back skin of *ILEI* ^*fl/fl*^ and *ILEI* ^*∆Ep*^ mice (*n* = 6–8 (G) and *n* = 2–8 (H); 3 independent experiments). Scale bar, 100 μm. Data information: In (C, G, and H), statistical significance was determined one‐way ANOVA with Tukey multiple comparison test (C, H) or by Student's *t*‐test (G) and marked with asterisks (*P < 0.05; **P < 0.01; ****P < 0.0001). If significance levels were different for the pairwise comparisons with combined marking, asterisks, valid only for a subset of the pairs were put into brackets. Source data are available online for this figure.

ILEI's pro‐inflammatory role in skin inflammation upon overexpression in keratinocytes raised the question, if lack of ILEI would ameliorate inflammation and thus, be of therapeutic value. To test this, mice with keratinocyte‐specific ILEI deletion (*ILEI* ^*ΔEp*^) were generated by crossing *ILEI* ^*fl/fl*^ mice to *K5cre* transgenic mice (Tarutani *et al*, 1997). *ILEI* ^*ΔEp*^ mice were fertile and showed no aberrant skin phenotype at homeostatic conditions (Fig 6A), indicating that the gene had no essential function in skin development and tissue maintenance. Importantly, however, we observed a significant decrease in epidermal thickening in TPA‐treated *ILEI* ^*ΔEp*^ mice (Fig 6A–C) compared with control littermates. Keratinocyte‐specific deletion was confirmed by IHC (Fig 6D). In contrast to our ILEI overexpression mouse model, the keratinocyte differentiation marker K10 showed a mosaic pattern of expression in the basal compartment of ILEI‐deficient epidermis (Fig 6E), and the number of microabscesses and the abundance of neutrophils was significantly lower in *ILEI* ^*ΔEp*^ skin compared with control littermates (Fig 6F–H).

During our study, we observed ILEI expression in several immune cell populations both locally in the skin and systemically. To test whether inhibition of ILEI function in the immune system would have any influence on overall fitness and inflammation, which would potentially also prohibit therapeutic considerations, we generated *ILEI* ^*ΔHem*^ mice bearing ILEI deletion in all hematopoietic cells by crossing *ILEI* ^*fl/fl*^ mice to *Vav1cre* transgenic mice (Georgiades *et al*, 2002). ILEI deletion in *ILEI* ^*ΔHem*^ mice was efficient, as assessed on spleen protein extracts (Appendix Fig S6A) and did not cause alterations in the relative abundance of different immune subpopulations, as analyzed in the spleen by flow cytometry (Appendix Fig S6B). Similarly, hematopoietic ILEI deletion had no effect on skin homeostasis (Appendix Fig S6C–E), nor did it have an effect on the severity of skin inflammation and thickening upon TPA treatment (Appendix Fig S6C–E). Thus, ILEI loss in immune cells was well tolerated both at homeostatic conditions and in a first test of a local inflammatory trigger. These preliminary data make ILEI a potentially feasible therapeutic candidate for psoriasis.

### Transcriptomic profile of TPA‐treated *K5‐ILEI* ^*ind*^ mouse skin is enriched in pathways associated with human psoriasis

**Figure 7:**
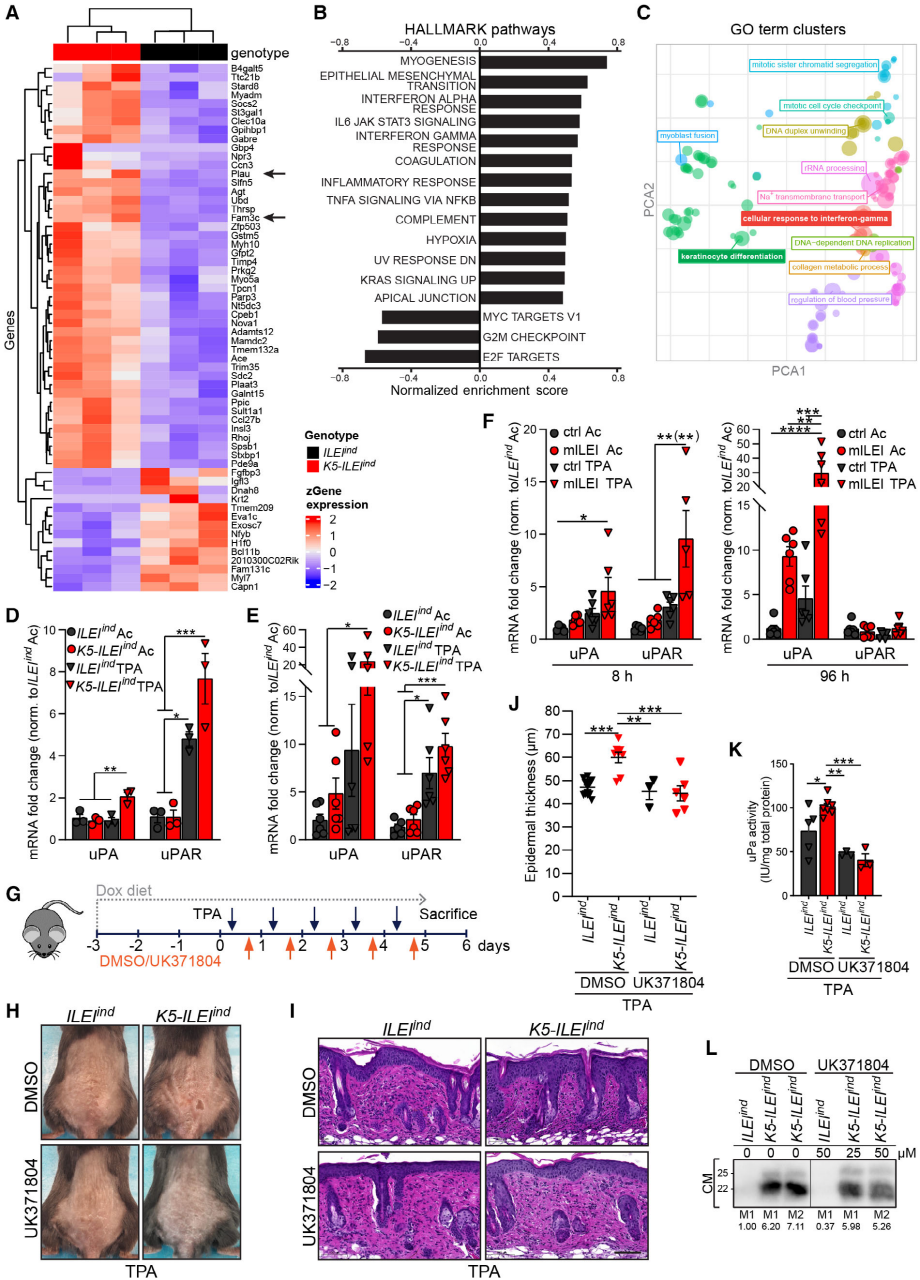
Transcriptomic profiling of the *K5‐ILEI* ^*ind*^ mouse model identifies uPA as therapeutic target in reducing epidermal thickening A
Heatmap of nonsupervised hierarchical clustering of differentially expressed genes (DEGs) in the back skin of *K5‐ILEI* ^*ind*^ vs *ILEI* ^*ind*^ mice kept on doxycycline diet and treated with TPA for 5 days (*n* = 3). Gene list was filtered for an adjusted *P*‐value of < 0.05 and a log fold change of ≥ 1. Arrows mark Fam3c/ILEI and Plau/uPA.B, C
Significantly enriched (B) pathways of the Hallmark database ranked according to their normalized enrichment score and (C) terms of the GO:term database plotted in clusters after dimensionality reduction with indicated lead terms in TPA‐treated *K5‐ILEI* ^*ind*^ mouse skin computed from the gene expression profiling described in panel A.D–F
Mean fold change ±SEM in mRNA expression of *uPA* and *uPAR* (D) in freshly sorted keratinocytes enriched for the interfollicular epithelium from acetone and TPA‐treated back skin, (E) in primary keratinocytes isolated from *ILEI* ^*ind*^ and *K5‐ILEI* ^*ind*^ mice, supplemented with doxycycline and treated with acetone or TPA for 96 h, and (F) in primary wild‐type keratinocytes treated with acetone and TPA and with murine recombinant wild‐type ILEI (mILEI) for 8 and 96 h (*n* = 3 (D), *n* = 6 (E,F); 1–2 independent experiments).G
Schematic drawing of the protocol used for TPA and UK371804 combination treatment. The two compounds were topically applied daily in a 5–6‐h interval for 5 days.H–K
(H) Macroscopic appearance, (I) hematoxylin–eosin staining, (J) mean epidermal thickness and (K) mean uPA activity ±SEM of the back skin of *ILEI* ^*ind*^ and *K5‐ILEI* ^*ind*^ mice kept on doxycycline diet and treated with the protocol shown in (G) (*n* = 3–9 (J), *n* = 3–7 (K); 3 independent experiments). Scale bars, 100 μm.L
ILEI Western blot analysis of conditioned media harvested from primary keratinocytes of *ILEI* ^*ind*^ and *K5‐ILEI* ^*ind*^ mice after 48‐h doxycycline induction and treatment with TPA and DMSO or indicated concentrations of UK371804. Loading was normalized to cell count, numbers indicate relative intensities. Lanes are from noncontinuous parts of the same gel. Data information: In (D, E, F, J, K) Statistical significance was determined by one‐way ANOVA with Tukey multiple comparison test and marked with asterisks (*P < 0.05; **P < 0.01; ***P < 0.001; ****P < 0.0001). Source data are available online for this figure.

**Figure EV3:**
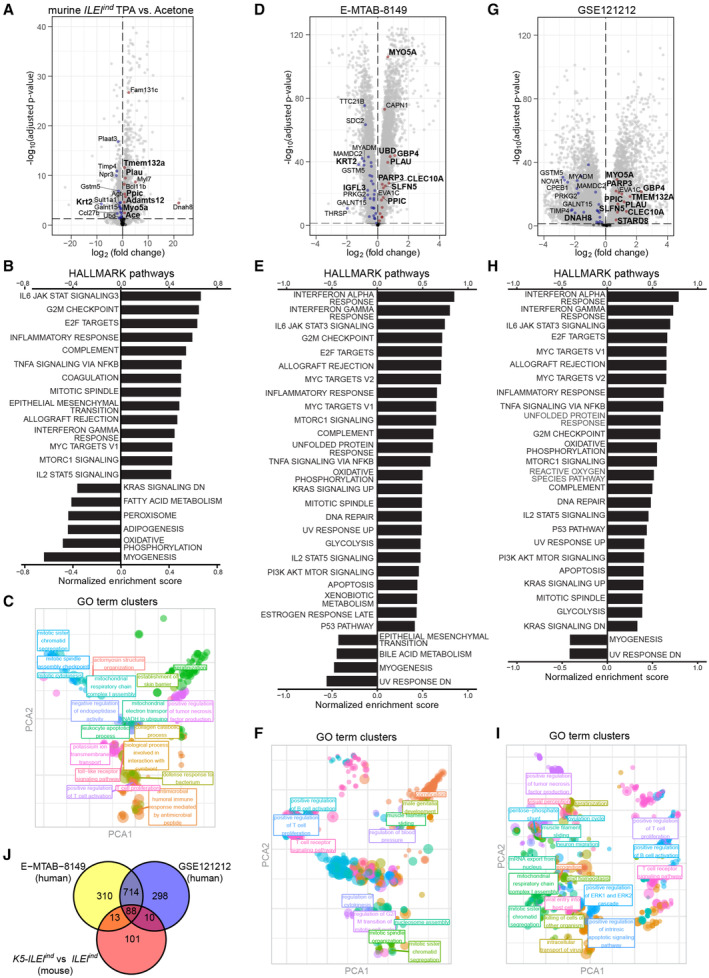
Differentially expressed genes and enriched pathways in murine TPA‐treated skin and in human psoriasis A–I
Transcriptomic analysis showing (A, D, G) Differentially expressed genes (DEGs), (B, E, H) significantly enriched pathways of the Hallmark database ranked according to their normalized enrichment score and (C, F, I) significantly enriched terms of the GO:term database plotted in clusters after dimensionality reduction with indicated lead terms computed from the gene expression profiling of (A–C) the back skin of *ILEI* ^*ind*^ mice treated with acetone or TPA for 5 days (*n* = 3) and the human psoriasis datasets (D–F) E‐MTAB‐8149 and (G–I) GSE121212. For the volcano plot on (A, D, G), cutoff was set for an adjusted *P*‐value of <0.05. Genes of the *K5‐ILEI* ^*ind*^ TPA signature (60 genes) are in blue (downregulated), red (upregulated) or black (nonsignificant), top 10 significant genes of the signature marked by names, if directionality maintained, in bold.J
Venn diagram on the distribution of enriched GO terms in the transcriptome of the back skin of *K5‐ILEI* ^*ind*^ vs *ILEI* ^*ind*^ mice treated with TPA and the two human psoriasis datasets E‐MTAB‐8149 and GSE121212.

To get a deeper understanding of ILEI‐regulated genes and pathways in skin inflammation with potential therapeutical implications, RNA sequencing of TPA‐treated skin from ILEI‐overexpressing and control mice was performed. Transcriptomic profiling of *K5‐ILEI* ^*ind*^ mouse skin revealed a set of 61 differentially expressed genes (DEG) with 14 genes downregulated and 47 genes upregulated (including *Fam3c*, confirming expression of the ILEI transgene; Fig 7A). Among the downregulated genes, *Krt2* and *Bc11b* were found that play an important role in keratinocyte differentiation (Zhang *et al*, 2012). Among the upregulated genes, we found *B4galt5*, linked to TNFα signaling (Parker *et al*, 2016), *Gbp4*, involved in interferone responses (Tretina *et al*, 2019) and interestingly, *Plau*, the protease involved in ILEI maturation and secretion (Csiszar *et al*, 2014). Enrichment analysis with Hallmark pathways (Liberzon *et al*, 2011) showed the highest enrichment for myogenesis, an indication of fibroblast activation, for EMT, a known function of ILEI, but also several immune regulating pathways including TNFα signaling via NFkB, the interferon‐gamma response and IL6‐JAK‐STAT3 signaling (Fig 7B), correlating with our experimental mechanistic findings on elevated TNFα expression, neutrophil recruitment, epithelial CD8+ T cell accumulation, and increased STAT3 signaling activity. Gene set enrichment analysis (GSEA) with GO terms showed 212 terms significantly (*P* < 0.05) enriched for *K5‐ILEI* ^*ind*^ skin including lead terms for keratinocyte differentiation, interferon‐gamma response and terms linked to cell division and proliferation (Fig 7C), further confirming our phenotypic findings on impaired epidermal differentiation and increased hyperproliferation. TPA treatment itself provoked a similar enrichment pattern (Fig EV3A–C), indicating that ILEI overexpression might not induce primarily alternative mechanisms, but rather increase the amplitude of inflammation‐activated pathways. Differential gene expression and GSEA of two independent human psoriasis datasets (E‐MTAB‐8149 and GSE‐121212; Fyhrquist *et al*, 2019; Tsoi *et al*, 2019) further confirmed the relevance of the pathways identified in the murine model (Fig EV3D–I). Of note, the two human psoriasis cohorts showed some variance in GO terms enriched at disease condition with a 71% (802/1,125 or 1,110) overlap (Fig EV3J). Importantly, however, out of the 113 GO terms of the murine data that were shared with at least one of the human datasets (113/212, 52%), over 90% (88/113) overlapped with both human psoriasis cohorts (Fig EV3J), indicating that ILEI‐linked processes hit core pathways commonly appearing in independent human psoriasis studies.

**Figure EV4:**
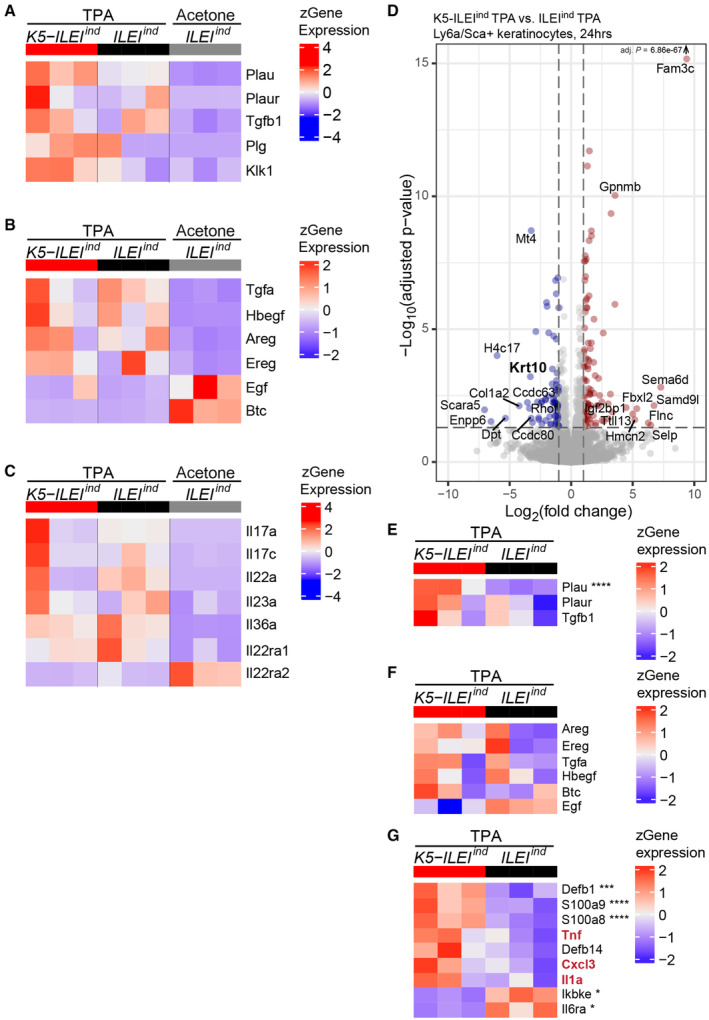
Transcriptomic profiling of total skin and freshly isolated keratinocytes of the *K5*‐*ILEI* ^*ind*^ mouse model identify psoriasis‐linked regulatory genes with ILEI‐ and TPA‐dependent relevance A–C
Heatmap on genes expressed in TPA or acetone‐treated *K5‐ILEI* ^*ind*^ and control back skin showing (A) regulatory genes on ILEI translation, secretion, and proteolytic cleavage, (B) EGFR ligands and (C) subset of psoriasis‐relevant cytokines and cognate receptors (*n* = 3).D
Volcano plot showing differentially expressed genes (DEGs) computed from the gene expression profiling of freshly sorted keratinocytes enriched for IFE isolated 24 h after a single TPA treatment of K5‐*ILEI* ^*ind*^ and *ILEI* ^*ind*^ mice kept on doxycycline diet (*n* = 3). Cutoff was set for an adjusted *P*‐value of <0.05 and a log2FC of ≥ 1. Top 10 up and downregulated genes are marked by names.E–G
Heatmap on genes expressed in freshly sorted keratinocytes enriched for IFE isolated 24 h after a single TPA treatment of *K5‐ILEI* ^*ind*^ and *ILEI* ^*ind*^ mice (*n* = 3) showing (E) regulatory genes on ILEI translation and secretion, (F) EGFR ligands and (G) inflammatory genes. Tnf, Il1a, and Cxcl3 are marked in red. Genes with an adjusted *P*‐value lower than 0.05 are marked with asterisks (*P < 0.05; **P < 0.01; ***P < 0.001; ****P < 0.0001).

Transcriptomic profiling of the *K5‐ILEI* ^*ind*^ model revealed a TPA‐dependent upregulation of TGFβ and uPA (Fig EV4A), providing a possible explanation how topical inflammatory triggers upregulated ILEI only at protein level in murine skin (see Fig EV2A–F). Some regulatory genes of ILEI translation, secretion, and proteolytic processing showed additional increase upon ILEI overexpression (e.g., *Plau*, see also above; Fig EV4A), indicating a feed‐forward regulation of ILEI on its own activity after inflammatory stimulus. Transcriptome data confirmed earlier findings on the strong link between TPA stimulus and the upregulation of EGFR ligands, IL17‐ and IL36‐family cytokines to propagate epidermal hyperproliferation and Th17 inflammatory response. At the same time, they also validated our mechanistic studies that ILEI overexpression did not affect the expression of these factors and the processes they regulate (Fig EV4B and C).

To delineate primary cell‐intrinsic ILEI‐dependent events in skin epidermis after TPA treatment, we also performed transcriptional profiling on freshly sorted keratinocytes enriched for IFE 24 h after TPA treatment of *K5‐ILEI* ^*ind*^ and *ILEI* ^*ind*^ mice. We found a set of 166 differentially expressed genes (DEG) with 66 genes downregulated and 100 genes upregulated (including *Fam3c*, confirming expression of the ILEI transgene; Fig EV4D). Keratin10 (K10) was among the top 10 downregulated genes, further confirming the importance of ILEI in inhibiting keratinocyte differentiation (Fig EV4D). Similarly strikingly, we refound *Plau* among the upregulated genes (Fig EV4E), indicating that ILEI's feed‐forward trigger on its own secretion might occur in an autocrine fashion. While none of the EGFR ligands, IL17 and IL36 family genes showed ILEI dependency (Fig EV4F), Tnfa, Il1a, and Cxcl3 as main neutrophil‐recruiting factors were upregulated upon ILEI overexpression (Fig EV4G), confirming our primary keratinocyte studies and pinpointing once more the early upregulation of these genes. Interesting additional hits, most probably masked in the total skin profiling, were (i) the upregulation of S100A8 and 9, strong inflammatory modulators, described to be the most upregulated proteins in psoriatic epidermis (Schonthaler *et al*, 2013; Wang *et al*, 2018) and (ii) Defb1, an antimicrobial peptide, highly upregulated in psoriasis with a strong genomic association between its increased gene copy number and the risk of psoriasis (de Jongh *et al*, 2005; Hollox *et al*, 2008). These additional hits with high and specific relevance in psoriasis further strengthen the importance of a direct regulatory role of ILEI in psoriatic disease manifestation.

### uPA is upregulated in inflamed skin upon ILEI stimulus and serves as a drugable therapeutic target

In our transcriptomic profiling, we found urokinase plasminogen activator (uPA) to be upregulated in TPA‐treated ILEI‐overexpressing mouse skin and keratinocytes (Figs 7A and EV4E). We also found uPA/PLAU upregulated in psoriasis patients, interestingly also in nonlesional skin, albeit more moderately than in lesions (see Fig EV1E and F), indicating that it might represent an early disease marker or potential risk factor tightly linked to ILEI expression. These data encouraged us to consider uPA inhibition as a method to block ILEI function in a therapeutic setting, as uPA is involved in the regulation of ILEI secretion and proteolytic maturation—both required for ILEI activity (Csiszar *et al*, 2014).

First, we verified whether uPA and its receptor (uPAR/PLAUR) were upregulated in keratinocytes in an ILEI‐dependent manner upon inflammation. Importantly, we found the levels of both uPA and uPAR to be significantly higher upon ILEI overexpression at inflammatory conditions in both freshly sorted and primary cultured keratinocytes and in wild‐type keratinocytes treated with recombinant ILEI (Fig 7D–F). With the recombinant protein, we also observed timely changes in uPA and uPAR regulation. Both were initially upregulated, uPA further showed an increase over time, whereas uPAR decreased to baseline after 96 h, indicating a receptor desensitization (Fig 7F). These data verified our keratinocyte transcriptomic data that ILEI has an autocrine feed‐forward loop on its own activity in keratinocytes by upregulating uPA expression upon inflammation, which makes uPA a promising therapeutic target in ILEI‐linked skin inflammatory diseases.

Next, we tested the effect of uPA inhibition on the disease outcome in our TPA‐induced ILEI‐overexpressing skin inflammation mouse model. As systemic inhibition of uPA has highly pleiotropic effects (Bevan & Mala, 2008; Masucci *et al*, 2022), we used topical application of the small molecule peptide inhibitor UK371804 and followed a treatment protocol depicted in Fig 7G. The augmented epidermal thickening observed in ILEI‐overexpressing mice upon TPA treatment was completely reverted to control levels upon inhibition of uPA (Fig 7H–J). Inhibition of uPA enzymatic activity was confirmed by a fluorometric activity assay on protein extracts of treated skin (Fig 7K). Mechanistically, UK371804 treatment showed a dose‐dependent inhibition of ILEI secretion in TPA‐treated *ex vivo* cultures of wild‐type and ILEI‐overexpressing primary keratinocytes (Fig 7L). These data show that uPA inhibition ameliorates ILEI‐dependent epidermal thickening in inflamed mouse skin by reducing ILEI secretion and indicates uPA inhibition as a potential therapeutic target for ILEI‐linked chronic inflammatory conditions, such as psoriasis.

### ILEI gene signature analysis separates psoriasis from normal condition and predicts uPA as clinically relevant therapeutic target

To evaluate the human relevance of our findings in the murine model, we tested the effect of recombinant human ILEI stimulus combined with TPA treatment in human primary keratinocyte cultures. We confirmed the dose‐dependent effect of recombinant ILEI on TNFa expression (Appendix Fig S7A) and recapitulated rapid ILEI‐dependent upregulation (after 8‐h stimulus) of TNFa, IL1a, CXCL1, and uPA in cultures of three independent donors (Appendix Fig S7B and C). uPAR and a selection of validated nontarget genes, such as Hbegf, IL17C, and IL36A, showed only TPA‐dependent increase, without an ILEI effect (Appendix Fig S7C and D). Of note, differences in the amplitude of TPA and/or ILEI responsiveness of the individual donors were very high, reflecting the individual heterogeneity of human primary cells compared with mouse models. These results show that the identified ILEI functions in the murine system were translatable to human with maintained specificity and selectivity of the responses.

**Figure 8:**
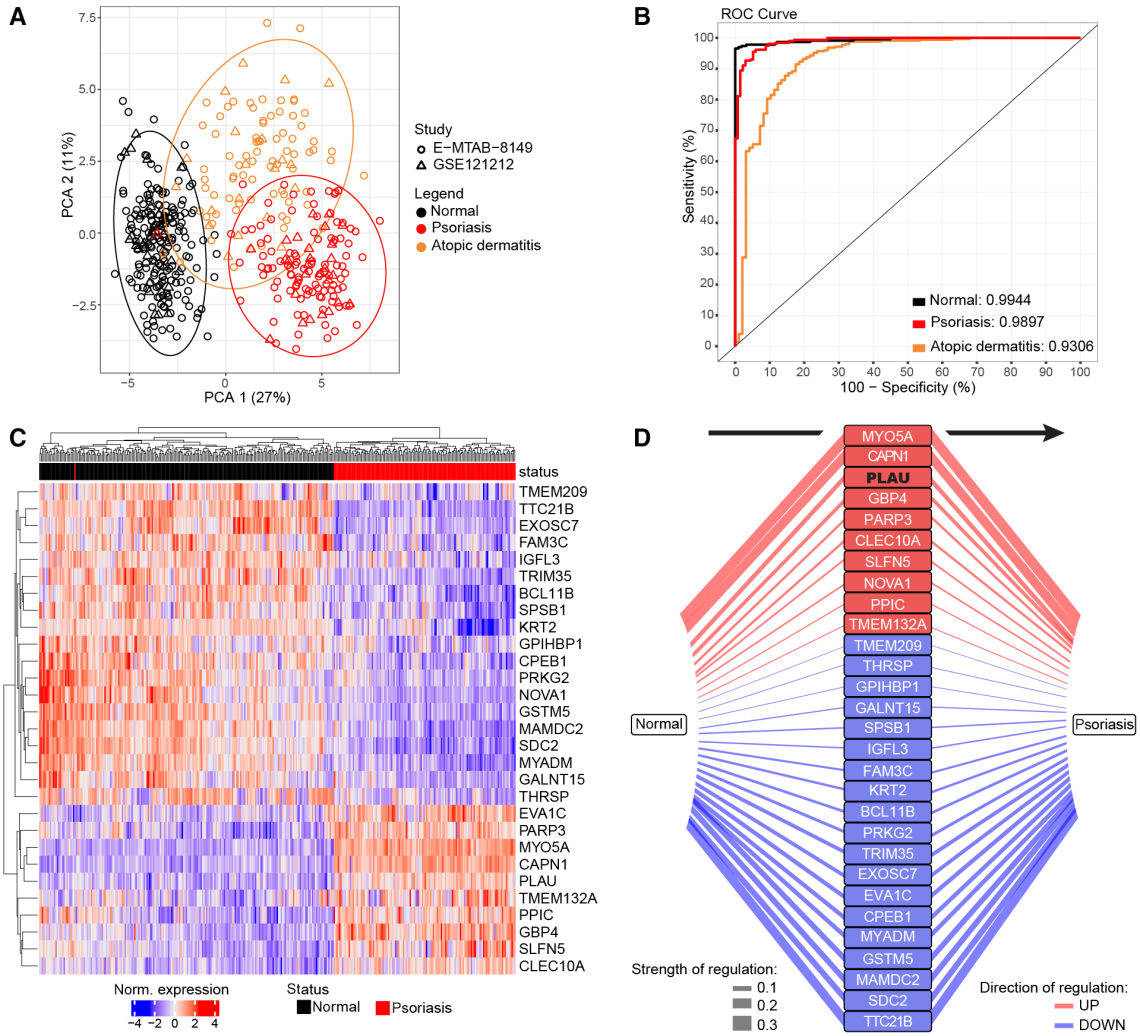
“ILEI signature” of the mouse model separates psoriasis from normal condition with uPA ranking among the top separator genes Principal component analysis of MINT‐integrated data of the two human datasets E‐MTAB‐8149 and GSE121212 consisting of psoriasis, atopic dermatitis (AD) patients and healthy controls based on the “ILEI signature” obtained from the mouse dataset. Circles mark clusters.Performance of the “ILEI signature”‐based classification model on the three disease states (normal, psoriasis and AD) of above two human datasets after MINT‐PLSDA‐based data integration plotted on a ROC curve. Discrimination ratio between given status versus both other states is marked in the legend.Heatmap of nonsupervised hierarchical clustering of the 29 “core separator genes” on MINT transformed expression data of normal and psoriasis conditions of the two human cohorts determined from the “ILEI signature” as number of genes necessary for optimal classification with lowest error rate tested by leave‐one‐out cross‐validation.Relevance network of the “core separator genes” for normal and psoriasis conditions ranked according to the strength of regulation, considering UP as positive, DOWN as negative scores. Width of the edges depicts the strength of regulation, color marks the direction of regulation. PLAU is highlighted in bold.

These findings encouraged us to evaluate the clinical relevance of our ILEI overexpression model. Thus, we tested the power of the 61 ILEI‐linked DEGs of the mouse back skin transcriptome as a psoriasis signature. Strikingly, this ILEI gene signature was able to separate psoriasis from healthy skin in a combined cohort of two human datasets comprising psoriasis and atopic dermatitis (AD) patients and healthy controls (Fig 8A). The discrimination rate for each of the three conditions was high, above 0.9, normal state being almost 100% distinguishable from pathological conditions and psoriasis discrimination being superior to AD with rates of 0.99 and 0.93, respectively (Fig 8B).

To identify the genes with strongest separation power and by this potentially highest clinical impact for psoriasis, we further refined the signature and determined a minimum set of 29 genes that was still able to maintain optimal separation (Fig 8C). uPA/PLAU was among the few targetable genes of this gene list, and relevance network analysis ranked it as top third “separator gene” of the ILEI signature genes upregulated in psoriasis (Fig 8D). These findings show the relevance and predictive power of an ILEI‐driven gene signature in hyperproliferative inflammatory skin conditions, especially psoriasis. In addition, it indicates that the therapeutic effect of uPA demonstrated in our *K5‐ILEI* ^*ind*^ psoriasiform mouse model has a high potential clinical impact in psoriasis therapy.

### Treatment‐induced changes in disease severity are accompanied by changes in uPA mRNA levels

**Figure EV5:**
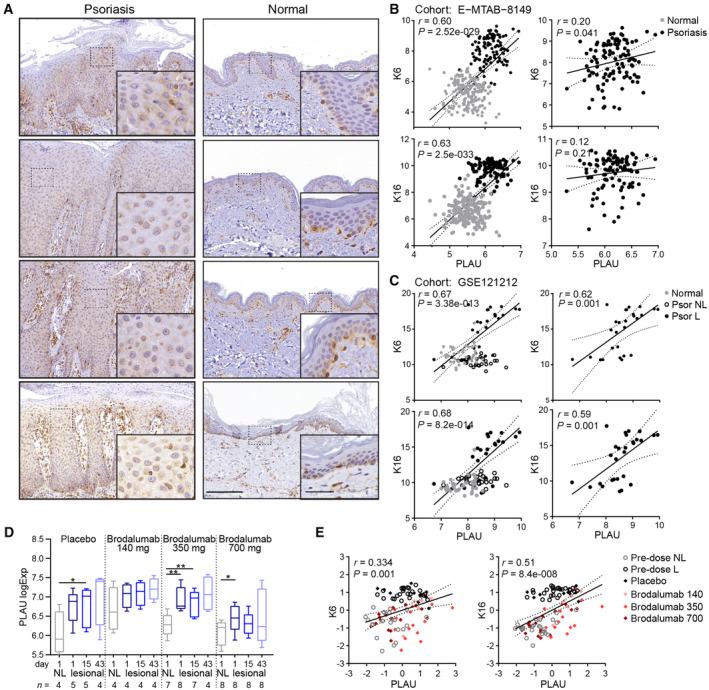
PLAU shows increased protein expression in the epidermis of psoriatic lesions and (therapy‐induced) changes in disease severity are accompanied by changes in uPA mRNA levels A
Representative images of PLAU immunohistochemistry on psoriatic (left panels) and normal (right panels) skin sections, scale bar 200 μm. Inlets show a magnification of the marked regions, scale bar 50 μm.B, C
Pearson correlation plots of K6 (upper panels) and K16 (lower panels) and PLAU gene expression in a combined set of normal and psoriatic skin (left panels) and in psoriatic lesions (right panels) of the datasets (B) MTAB‐8149 and (C) GSE121212.D
log2fold *PLAU* mRNA expression levels of the GSE53552 dataset consisting of lesional and matched nonlesional (NL) psoriatic skin (*n* = 25 patients) obtained at treatment start (day 1) and from the lesions as serial biopsies at 15 and 43 days after placebo (*n* = 5 patients) or Brodalumab therapy with doses of 140 mg (*n* = 4 patients), 350 mg (*n* = 8 patients) and 700 mg (*n* = 8 patients). The number of included patient samples at each condition and time points is marked under the diagram. Box‐and‐whiskers plot: Central band shows median, box extends from the 25^th^ to 75^th^ percentiles, and whiskers go from the smallest (min) to the largest (max) value.E
Pearson correlation plots of K6 (left panel) and K16 (right panel) and PLAU gene expression of the GSE53552 dataset stratified for nonlesional (NL) and lesional (L) psoriatic skin before therapy (predose) and with placebo or 140, 350, and 700 mg doses of Brodalumab therapy.

IHC analysis indicated that ILEI protein levels correlate with psoriasis severity (Fig 1D). The identified transcriptional control of ILEI on uPA prompted us to address whether uPA levels also show a similar correlation. Expression of uPA in psoriasis was assessed by IHC on the panel of healthy and lesional skins as described for ILEI. uPA showed elevated levels in psoriatic epidermis (Fig EV5A), resembling the pattern of ILEI localization. Like ILEI, uPA was mainly restricted to basal keratinocytes in healthy skin, whereas it was homogenously distributed throughout the whole epidermis with a dotted perinuclear subcellular accumulation indicative for secretory structures in psoriatic skin (Fig EV5A, inlets). However, keratinocytes were not the major source of uPA in the skin, and its higher abundance in many other cell types, partially intercalating into the epidermal compartment, did not allow IHC‐based quantification and correlation analysis on severity. To overcome this, we utilized two independent transcriptomics datasets with the limitation that they lacked annotation on clinical severity scores. Therefore, *Keratin 6 (K6)* and *Keratin 16* (*K16)*, described as close molecular correlates of psoriasis severity (Wang & Chang, 2003), were used. *uPA/PLAU* showed strong positive correlation to both of the “psoriasis severity” marker genes in both analyzed cohorts, and the two‐gene combinations separated normal from psoriasis samples, confirming the power of uPA as a psoriasis signature gene (Fig EV5B and C, left panels). In psoriatic lesions alone, the correlation strength of uPA to K6 and K16 was, however, very different in the two analyzed cohorts, strong and significant in GSE121212, but overall weak in E‐MTAB‐8149 (Fig EV5B and C, right panels). To reduce the effect of disease/dataset heterogeneity on severity correlation studies, we utilized a transcriptomic dataset on matched skin biopsies repeatedly taken from the same cohort of psoriasis patients before and after therapy with the anti‐IL17R antibody Brodalumab (Russell *et al*, 2014). This clinical study reported a dose‐dependent improvement in the clinically used PASI severity index (Papp *et al*, 2012), as well as the dose‐dependent conversion of the lesional psoriasis transcriptome to a nonlesional signature, including the validation of a strong positive correlation of K6 and K16 with the PASI score (Papp *et al*, 2012; Russell *et al*, 2014). Using these data, we could confirm elevated uPA/PLAU expression in psoriatic lesions compared with nonlesional skin at treatment start, albeit it showed high variance between the different randomized treatment subsets (Fig EV5D). While placebo, low‐ and mid‐dose treatments did not cause any decrease in the median expression of uPA, high‐dose therapy lead to a gradual decrease of uPA mRNA levels over time (Fig EV5D). This was in line with the reported observation that lower exposures showed transient or incomplete molecular responses (Russell *et al*, 2014). uPA/PLAU expression showed significant positive correlation in this cohort with both “severity markers”, K6 and K16 (Fig EV5E and F), and importantly, samples with decreased uPA levels after high‐dose therapy associated with decreased K6 and K16 expression, specifically mapping to the profile of nonlesional skin samples (Fig EV5E and F). These preliminary data indicate that (therapy‐induced changes in) disease severity is accompanied by concomitant changes in uPA expression. Future studies are needed to delineate the strength of this relationship.

### ILEI protein levels are also elevated in lesions of other skin diseases linked to chronic inflammation.

The ILEI gene signature was able to separate not only psoriasis but also AD from healthy skin with a high discrimination rate. This encouraged us to test whether ILEI protein expression was increased also in other chronic inflammatory skin diseases. Besides AD, mycosis fungoides and lupus erythematosus skin biopsies were analyzed by IHC (Appendix Fig S8). Each of the diseases showed increased ILEI protein levels compared with normal skin both in the epidermis and in dermal infiltrates at variable levels between individual samples (Appendix Fig S8). This indicates that our findings on ILEI's role in psoriasis might have a broader relevance for inflammatory skin diseases.

## Discussion

Inflammatory skin diseases manifest through the involvement of several cells, such as keratinocytes, immune cells, fibroblasts, endothelial cells and the cytokines and chemokines released by these cells in the skin. Psoriasis has been widely studied in the last few decades, and several therapeutic approaches were developed to target specific cells (T cells), chemokines (TNFα), and interleukins (IL‐17 and IL‐23) for treatment. However, there is still more to be explored in the context of the disease mechanism.

ILEI, a pro‐inflammatory cytokine, has been shown to be prognostic in several human cancers (Waerner *et al*, 2006; Gao *et al*, 2014; Yin *et al*, 2018). However, the role of ILEI in inflammatory diseases has not been mechanistically explored so far. In this study, we demonstrate the relevance of ILEI in skin inflammation here exemplified in human psoriasis and in a novel ILEI‐overexpressing murine psoriasiform disease model.

Several genetically engineered mouse models with an epidermal manipulation of gene expression develop psoriasis‐like skin lesions upon or without an inflammatory trigger (Wagner *et al*, 2010; Swindell *et al*, 2011; Nakajima & Sano, 2018). Here, we used transgenic mice with inducible keratinocyte‐specific ILEI overexpression (*K5‐ILEI* ^*ind*^) combined with TPA treatment to investigate the mechanistic involvement of ILEI in psoriasis. *K5‐ILEI* ^*ind*^ mice showed no apparent altered phenotype at homeostatic conditions, an inflammatory agent (TPA and IMQ) was required for the development of the psoriasiform phenotype. This is distinctive to some other murine psoriasis models, for example, induced epidermal deletion of *cJun/JunB*, where deletion of these AP‐1 proteins in keratinocytes is sufficient for spontaneous disease development (Zenz *et al*, 2005). The *K5‐ILEI* ^*ind*^ model shows primarily an enhanced myeloid drive. Most likely, induced by the upregulation of several neutrophil‐recruiting factors, such as TNFα. IL17A‐regulated processes were not affected by ILEI. *cJun* ^*ΔEp*^ */JunB* ^*ΔEp*^ mice also show a myeloid‐psoriasiform phenotype dominated by TNFα signaling and neutrophil accumulation (Guinea‐Viniegra *et al*, 2009). The IMQ‐induced psoriasis model acts, however, primarily via the activation of a Th17 response initiated by DCs (Van der Fits *et al*, 2009). *K5.Stat3C* mice also share some features with the *K5‐ILEI* ^*ind*^ model. Although *K5.Stat3C* mice develop some psoriasis‐like symptoms over time, for adequate disease modeling, an additional trigger is applied, such as TPA treatment or wounding (Sano *et al*, 2005). As injury to the skin (Koebner phenomenon) is considered as a main trigger in psoriasis manifestation (Ji & Liu, 2019), it will be interesting to test the effect of wounding also in the *K5‐ILEI* ^*ind*^ model. Similarly, it will be interesting to investigate, how far other psoriasis models utilize ILEI signaling as an effector axis for disease manifestation. Our studies showed that both, TPA and IMQ, two inflammatory agents with very different mechanisms of action (Stanley *et al*, 1991; Van der Fits *et al*, 2009) upregulated ILEI protein levels and caused a more severe psoriasiform phenotype in *K5‐ILEI* ^*ind*^ mice, indicating that ILEI overexpression could be considered as a psoriasis risk factor model that can be investigated in diverse psoriasis driver backgrounds.

ILEI has a strong translational control via TGFb (Waerner *et al*, 2006; Chaudhury *et al*, 2010a), and its secretion is regulated by the uPA‐uPAR signaling axis (Csiszar *et al*, 2014). Indirectly, through the regulation of proteolytic cascades, plasminogen (Plg) and Kallkrein also play a role in ILEI secretion (Lund *et al*, 2006; Sotiropoulou *et al*, 2009; Smith & Marshall, 2010). Plasmin, in addition, also activates ILEI by proteolytic cleavage, and other serine proteases, for example, neutrophil elastase (NE), show also this capacity (Csiszar *et al*, 2014). As our data demonstrate, ILEI signaling upregulated uPA transcription, indicating that proteolytic activation of ILEI generates a feed‐forward loop on its own secretion. Thus, directly or indirectly, all these factors contribute to increased ILEI protein levels. TGFb1 has an important role in psoriasis: Increased epidermal and serum TGFb1 levels correlate with disease severity, and mouse models with keratinocyte‐specific human TGFb1 overexpression (K5.hTGFb1^wt^) recapitulate the human disease (Han *et al*, 2010). uPA and plasmin are also upregulated in psoriasis (Li *et al*, 2011; Rubina *et al*, 2017), NE levels similarly (Krasavin *et al*, 2019). Latter also contributes to the proteolytic maturation of IL36 family cytokines, prominent factors of psoriasis development (Henry *et al*, 2016). Thus, we can anticipate that NE has a contribution also in ILEI activation in psoriasis. Our mRNA profiling showed that some of these factors were transcriptionally upregulated upon TPA treatment and ILEI overexpression further boosted the expression of a subset of them, most potently of uPA. These data support the observation that elevated ILEI protein levels can be achieved without transcriptional upregulation in psoriasis and also indicate that the necessity of TPA for the manifestation of a psoriasiform phenotype in our ILEI‐overexpressing model can be partially explained by its contribution to the upregulation of the ILEI translational and activating enzymatic machinery.

ILEI exists both, in monomers and dimers, dimers having been described as the ligand for LIFR and responsible in inducing EMT and invasion (Kral *et al*, 2017; Woosley *et al*, 2019). Similarly, we found that in keratinocytes ILEI dimers, not monomers, affected differentiation and target gene expression. Thus, this study also extends the panel of ILEI functions linked to its dimerization.

Several studies have shown the involvement of STAT3 activation in psoriasis (Calautti *et al*, 2018; Nakajima & Sano, 2018). ILEI was also shown to act via LIFR/STAT3 axis in inducing EMT (Woosley *et al*, 2019). Recently, the ILEI/LIFR complex has been reported to induce EMT by activating Akt and Erk pathways in renal interstitial fibrosis (Zhou *et al*, 2022). Here, we demonstrate how ILEI orchestrates the interplay of these signaling pathways upon an inflammatory stimulus in keratinocytes. We propose that ILEI utilizes Ser727 phosphorylation‐driven STAT3 activation to mediate its effects in keratinocytes and that PI3K and Mek are important transducers of this activation. Our data show that tyrosine phosphorylation was a prerequisite of Ser727 phosphorylation, indicating that it was an essential initiating event of further activation of the molecule. Mek and PI3K inhibitors efficiently blocked TPA‐induced elevated ILEI target gene expression affecting, however, only Ser727, but not Tyr705 phosphorylation on STAT3, indicating that Ser727 phosphorylation was responsible for the ILEI‐dependent effects. In addition, ILEI‐dependent elevated phospho‐Serine727 STAT3 levels showed a clear nuclear accumulation in the skin. Considering that many differentially expressed genes were found similarly in ILEI‐overexpressing TPA‐treated skin and upon TPA treatment alone, in latter with a lower amplitude, suggests to us that ILEI‐driven STAT3‐Ser727 phosphorylation might potentiate the activity of the STAT3 transcription factor in keratinocytes. Thus, our study identified STAT3 serine phosphorylation as an important amplifier in ILEI signal transduction that connects STAT3, Akt, and Erk signaling. Future work should focus to uncover the general relevance of our findings in ILEI‐STAT3 signaling, for example, by revisiting LIFR‐STAT3 signaling axis in EMT‐MET switch in cancer (Lin *et al*, 2021).

Some previous studies have indicated a possible role for ILEI in inflammation, but it has so far never been investigated mechanistically (Chaudhury *et al*, 2010a, 2010b). Maybe, due to its strict translational control it was overlooked before. For example, ILEI was identified among the top 20 upregulated proteins in a secretome study of murine macrophages, stimulated with a Stimulator of interferon genes (STING) agonist, without any detectable changes at the transcript level (Motani & Kosako, 2018). In this study, we found ILEI being upregulated at protein level in psoriatic skin and propose ILEI protein as a potential novel biomarker that correlates with disease severity.

Transcriptomic profiling of TPA‐treated *K5‐ILEI* ^*ind*^ murine skin revealed a set of 61 DEGs and GSEA showed enrichment for several immune regulating pathways. Many of these are novel in context of ILEI function. A substantial fraction of gene sets enriched are shared with psoriasis and the “ILEI signature” separated psoriatic from normal skin in two independent patient cohorts. This indicates that, although transcriptionally unchanged, ILEI's elevated protein levels and activity control core mechanisms of the disease and generates a transcriptional fingerprint of high potential clinical value. It will be interesting to explore our “ILEI signature” further to define its predictive power in making therapy decisions.

Psoriasis treatment is now moving toward targeted therapies using specific inhibitors and antibodies. (Campa *et al*, 2015; Wasilewska *et al*, 2016). However, there is no predictive model for treatment response in clinical use (Yiu *et al*, 2021). In addition, current treatments have several side effects in patients and sometimes application must be stopped (Lebwohl *et al*, 2015; Wasilewska *et al*, 2016; Lindhaus *et al*, 2017; Murdaca *et al*, 2019). Hence, it would be clinically relevant to explore the therapeutic potential of ILEI in psoriasis treatment further. Importantly, we show that ILEI deletion in immune cells affects neither gross systemic immune composition nor homeostasis and inflammatory response in the skin. These findings support the idea that ILEI could be utilized as a therapeutic target in the treatment of psoriasis.

Currently, there are no tools available to directly block ILEI function, for example, in the form of neutralizing antibodies. Nevertheless, there are several inhibitors against urokinase (uPA/Plau; Fish *et al*, 2007; Masucci *et al*, 2022). uPA was among the top upregulated genes in our transcriptional profile. We validated its upregulation in ILEI‐overexpressing freshly sorted and *ex vivo* cultured keratinocytes, as well as upon treatment with recombinant ILEI following TPA treatment. These data indicate that ILEI induces a feed‐forward loop on its own secretion and activation in keratinocytes in a cell‐autonomous manner and by this amplifies ILEI signaling‐linked pathological processes. Of note, the urokinase plasminogen activator system was shown in earlier immunohistochemistry studies to be upregulated in psoriasis, and uPA and plasmin having been proposed as potential therapeutic targets (Li *et al*, 2011; Rubina *et al*, 2017). Encouraged by these findings, in a therapeutic intervention, pharmacological inhibition of uPA ameliorated ILEI‐dependent epidermal thickening in inflamed mouse skin and reduced ILEI secretion. Although uPA is involved in wound healing processes, there it acts redundantly with its close relative tissue plasminogen activator (tPA) and only dual deletion of the two genes affects wound repair (Lund *et al*, 2006). The availability of selective inhibitors for these two proteases ensures efficient and specific blockage of uPA without side effects on skin integrity (Fish *et al*, 2007). As only uPA, but not tPA is involved in the regulation of ILEI secretion (Csiszar *et al*, 2014), selective efficient inhibition of ILEI activity is therapeutically possible. Further refinement of the ILEI gene signature identified uPA as top third among the 29 genes minimally required for optimal separation of psoriasis from normal condition. These results indicate uPA inhibition as potential therapeutic target for psoriasis and possibly other ILEI‐linked pathological conditions.

Indeed, preliminary IHC data on other chronic inflammatory disease, such as AD, mycosis fungoides and lupus erythematosus indicate that ILEI protein levels are increased in these diseases as well. Hence, the question arises if ILEI has any psoriasis specificity, or it should be considered as a broad inflammatory marker. Interestingly, the ILEI signature was not only able to distinguish psoriasis from normal condition, but it also separated AD from both, normal and psoriasis conditions, indicating that genes of the signature are important in both diseases, but at the same time, they might have different weighting in the two pathological conditions, and thus, ILEI‐linked pathways might have distinctive regulations in the different diseases. Thus, it will be interesting for future research with high clinical impact, to identify commonalities and specificities of ILEI‐driven regulatory mechanisms in the different inflammatory skin diseases.

In conclusion, we show that ILEI protein levels are upregulated in psoriatic skin and elevated ILEI protein levels contribute to a more severe psoriasiform phenotype in mice. The high therapeutic potential of ILEI and its regulator uPA found in this study will likely open up new opportunities in psoriasis therapy.

## Materials and Methods

### Human material

The IHC study on psoriatic and other skin disease tissue biopsies was approved by the Ethics Committee of the Medical University of Vienna, Austria (EK 1783/2020). Written informed consent was obtained from each individual before inclusion, according to the WMA Declaration of Helsinki. Experiments conformed the Department of Health and Human Services Belmont Report. From formalin‐fixed, paraffin‐embedded 4 mm full‐thickness punch biopsies taken from lesional skin of patients for diagnostic reasons (psoriasis *n* = 5, AD *n* = 4, mycosis fungoides *n* = 4, lupus erythematosus *n* = 2), after finalizing the medical report, 4‐μm‐thick sections were cut. After deparaffinization, sections were subjected to antigen retrieval (citrate buffer pH 6), incubated with primary anti‐ILEI (HPA050548, 1:1,000, Human Protein Atlas; all cases) and anti‐PLAU (HPA070796, 1:700, Human Protein Atlas; psoriasis) antibodies followed by secondary HRP‐coupled antibody (Cell Signaling Technology), DAB reaction and hematoxylin counterstain. Stained slides were scanned using Pannoramic MIDI slide scanner (3DHISTECH).

The study on human primary keratinocyte cultures was approved by the ethics committee of the Medical University of Vienna, Austria (Vote 1969/2021). All donors (*n* = 3) gave their written informed consent.

### ILEI IHC cellular quantification

Quantification was performed by cellular segmentation followed by the calculation of 3,3′‐diaminobenzidine (DAB) chromogen intensities. Briefly, the analysis of the stained human skin sections was performed using a Fiji Macro. As additional plugins we used “Stardist” (https://github.com/stardist/stardist) and “MorpholibJ” (https://imagej.net/MorphoLibJ). Smaller subsets from the tissue scans were exported for the analysis. A manual region of interest was drawn on each image to define the borders of the epidermal tissue. For measuring individual cells, the stained nuclei were detected via the “Stardist” deep learning plugin using the predefined model for bright‐field sections. These segmented nuclei could be manually modified if needed and were then used to define the whole cells via growing into the surrounding tissue using the “Marker controlled watershed”—algorithm of “MorpholibJ.”

For quantifying the ILEI staining, the “Fiji color deconvolution plugin” was used to create separate channels via the predefined setup for DAB and hematoxylin staining. The resulting DAB channel was inverted to measure mean and sum intensities within the previously defined cell borders. In addition to the cellular measurement, intensities were also measured over the whole‐defined region.

### Isolation, cultivation, and treatment of human primary keratinocytes

Fresh human skin specimens from surgery were taken, and the epidermis and dermis were separated by incubating in Dispase II (2.4 U/ml; Roche Diagnostics GmbH, Mannheim, Germany) overnight at 4°C. Single keratinocytes (KCs) were obtained by digesting the epidermis with 0.05% Trypsin–EDTA (Thermo Fisher Scientific, Waltham, MA) and DNAse I (Sigma‐Aldrich, St. Louis, MO) for 7 min at 37°C. Primary KCs were cultured in KC Growth Medium‐2 (PromoCell) at 37°C, in 5% carbon dioxide, and 95% relative humidity, expanded for up to 10 days in the first passage, after which they were frozen and then thawed for the experiments. For stimulation, 1 × 10^5^ cells were seeded in 12‐well plates and cultured for 24 h, followed by media change to KC Basal‐Medium‐2 (PromoCell) for 15 h before treatments. Purified recombinant human ILEI (hILEI; Kral *et al*, 2017) was added for dose–response studies at concentrations of 100, 250, and 500 ng/ml and for other treatments at 500 ng/ml. Co‐treatments with TPA and acetone as vehicle control were performed at a concentration of 20 ng/ml. After 8 h of treatment, cells were harvested and total RNA was isolated.

### Mice

Mice were kept in the animal facility of the Medical University of Vienna in accordance with institutional policies and federal guidelines. Animal experiments were approved by the Animal Experimental Ethics Committee of the Medical University of Vienna and the Austrian Federal Ministry of Science and Research (animal license numbers: GZ BMWFW‐66.009/0319‐V/3b/2019). *K5‐ILEI* ^*ind*^ mice were generated using the tetracycline‐inducible reverse transactivator (rtTA; tet‐ON) approach. For this, *K5rtTA* mice (Vitale‐Cross *et al*, 2004) were crossed to mice expressing a C‐terminally FLAG epitope‐tagged version of an ILEI transgene under the control of the tet‐operon (*ILEI* ^*ind*^; Schmidt *et al*, 2023). To induce ILEI overexpression, doxycycline diet was administered at a dose of 1,000 mg/kg starting 3 days prior to treatments. For conditional deletion of *ILEI*, transgenic mice were generated inserting loxP sites flanking exons 2 and 3 of the *FAM3C/ILEI* gene locus (*ILEI* ^*fl/fl*^). *ILEI* ^*∆Ep*^ mice were generated by crossing *ILEI* ^*fl/fl*^ mice to transgenic mice expressing Cre recombinase under the control of bovine K5 promoter (*K5Cre*; Lichtenberger *et al*, 2010). *ILEI* ^*∆Hem*^ mice were generated by crossing the *ILEI* ^*fl/fl*^ mouse strain to *Vav1‐Cre* transgenic mice (Georgiades *et al*, 2002). Primers for genotyping are listed in Appendix Table S1.

### TEWL measurement

TEWL of back skin was measured with Tewameter^®^ TM 300 probe attached to the MDD4‐display device (Courage + Khazaka) according to the manufacturer's recommendations.

### Treatments of mice

Female *K5‐ILEI* ^*ind*^ mice and control littermates at the age of 7–8 weeks were shaved on the back skin with an electrical animal razor (AESCULAP GT608). 12‐O‐tetradecanoylphorbol‐13‐acetate (TPA) treatment was performed by pipetting 100 μl of a 0.1 mM solution onto the back skin for five consecutive days. Acetone treatment was used as vehicle control. If litter size allowed, both treatments were included for both, control and overexpressor genotypes. For imiquimod (IMQ) treatment, the same schedule was used at a daily dose of 5 mg of a 5% cream formulation (Aldara, Meda Pharma). For the combined treatment of TPA with the uPA inhibitor UK371804 or its respective vehicle control DMSO: UK371804 was applied daily 5–6 h after TPA treatment over a time of 5 days by pipetting 40 μl of a 50 mM solution onto the back skin. Mice were sacrificed and tissues dissected for further analysis on Day 6. Macroscopic changes observed on the back skin were photographically recorded.

### Quantification of epidermal thickness

Tissue biopsies from treated back skin of mice were collected, fixed in 10% formalin, and embedded in paraffin. 4‐μm‐thick skin sections were prepared and stained with hematoxylin and eosin (H&E; Sigma‐Aldrich). Epidermal thickness was determined blindly from H&E stained skin sections by selecting 4 independent microscopic fields on random areas and measuring the thickness at four interfollicular epidermal sections in these fields using Case Viewer (CSV, 3DHISTECH) and calculating the average of the 16 measurements.

### Immunohistochemistry (IHC) and immunofluorescence (IF)

Primary antibodies listed in Appendix Table S2 were used applying standard protocols. Briefly, 4‐μm sections (superadherent glass slides, Dako, K8020) of paraffin‐embedded mouse back skin were deparaffinized and antigen retrieval was performed in a steamer for 1 h with citrate buffer (Target Retrieval S1699, DAKO). Samples were washed with 1X PBS 3 times, treated with 3% H_2_O_2_ (Sigma‐Aldrich) for 10 min, and after repeated washing incubated in Superblock (Scy‐Tek) for 6 min and subsequently for mouse antibodies on mouse tissue in mouse‐on‐mouse block for 1 h (Scy‐Tek). Antibodies were diluted in blocking buffer (2% BSA, 10% Horse serum and 0.1% Tween 20 in PBS) and incubated overnight at 4°C. For IHC, samples were washed and incubated with HRP‐conjugated secondary antibodies (Cell Signaling Technology) for 30 min at room temperature, developed with DAB substrate (Dako) followed by hematoxylin counterstain, rehydration and mounting. Slides were digitized with a Pannoramic SCAN II slide scanner (3DHistech) in extended focus scanning mode using a 20X plan‐apochromat objective (0.8 NA) and a 5Mpxl sCMOS camera. Quantification was performed using an automized histology quantification software (Definiens Tissue studio® 4.3). Giemsa staining was performed to identify mast cells as described previously (Lichtenberger *et al*, 2013) and subsequently processed as IHC slides for imaging. For IF, samples were washed and incubated with fluorochrome‐conjugated secondary antibodies (Guinea Pig Alexa 594, Invitrogen; Rabbit Alexa 488, Invitrogen), counterstained with Hoechst33342 (Life technologies), and mounted (Aquamount, DAKO). IF stainings were captured using confocal (for K5, K10; Zeiss LSM 700 point laser confocal microscope with laser excitation, using either 40X, Plan‐NeoFluar NA 1.3 Oil or 63X Plan‐pochromat NA 1.4 oil immersion objectives and default (72 μm) pinhole for single frames) or epifluorescence (for phosphoSer^727^‐STAT3, phosphoErk1/2, phospho‐Akt; Nikon 80i (Nikon, Japan) widefield epifluorescence microscope using a 40X Plan Fluor NA 0.75 DIC objective, an EXFO white light source in combination with UV‐2A, FITC (465–495 nm) DM505 and G‐2A (510–560 nm) DM575 fluorescence filters as well as a Nikon DS Qi2 Monochrome CMOS camera for image acquisition) microscopy.

### Isolation of murine primary keratinocytes, *ex vivo* TPA treatments and differentiation

Keratinocytes were isolated from treated back skin or tails and ears of the mice as mentioned previously (Lichtenberger *et al*, 2010) with modifications. Briefly, back skin was cut and fat tissue was scraped off. Tissues were kept in ice‐cold PBS till the preparation of all the tissues. PBS was exchanged with fresh PBS containing 0.8% trypsin and incubated for 1 h at 37°C, and digestion was stopped by replacing the solution with PBS supplemented with 10%FCS. Epidermal layer was separated from the dermis. Epidermal tissue was incubated in keratinocyte growth medium (Minimal Essential Medium [MEM; Gibco] supplemented with 0.03 mg/ml bovine pituitary extract [Promocell], 0.125 ng/ml murine EGF [Peprotech], 5 μg/ml insulin [Sigma], 0.39 μg/ml epinephrine [Sigma], 10 μg/ml transferrin [Sigma], 0.33 μg/ml hydrocortison [Sigma], 10 μM ethanolamine, 10 μM phosphoethanolamine [Sigma], 0.02 mM CaCl_2_ [Promocell], and 1× Glutamine, 1× Pen/Strep [Sigma]) supplemented with DNaseI (250 μg/ml, Sigma) for 30 min at 37°C with shaking at 250 rpm. Suspension was filtered through a 70 μm cell strainer and centrifuged at 300 *g* for 5 min at 4°C. Cell pellet was dissolved in KGM medium and plated at a density of 3 × 10^5^ cells/ml on plates precoated with KC coating medium (MEM [Gibco] supplemented with 1% PureCol [Advanced Biometrix], 10 μg/ml human Fibronectin [Gibco] and 1 mg/ml BSA fraction V, 20 mM HEPES, 1 mM CaCl_2_ [Sigma]). Keratinocytes were cultured at 32°C and 5% CO_2_. *Ex vivo* TPA treatments with acetone as vehicle control were performed at a concentration of 20 ng/ml for 4–96 h earliest starting next day after seeding. To induce ILEI transgene overexpression, cells were treated with 5 mg/ml doxycycline (Sigma) at seeding or at indicated time points. Keratinocyte differentiation was induced by the addition of 0.3 mM CaCl_2_ for 72 h before harvest. Treatment for dose–response studies with recombinant murine ILEI (mILEI) and its mutant monomeric form (mILEI^CA^) was performed at concentrations of 125, 250, 500 and 1,000 ng/ml for 96 h, other mILEI treatments with 250 ng/ml for 4–96 h.

### Inhibitor treatment of murine keratinocyte cultures

Keratinocytes were pretreated with STAT3 inhibitor (STATTIC, 10 μM, Selleckchem), PI3K inhibitor (LY94002, 10 μM, Selleckchem), and Mek inhibitor (UO126, 10 μM, Sigma) for 30 min followed by the simultaneous addition of mILEI (250 ng/ml) and TPA/Acetone (20 ng/ml) in the continued presence of the inhibitors. After 4 or 6 h of TPA induction, cells were harvested and whole cell lysate was prepared or RNA was isolated, respectively.

### Immunocytochemistry of murine keratinocytes

Isolated primary keratinocytes of treated or nontreated *ILEI* ^*ind*^ and *K5‐ILEI* ^*ind*^ mice were cultured in chamber slides (Nunc Lab‐Tek, Thermo Scientific). At treatment endpoint, cells were fixed in 4% PFA and permeabilized with 0.1% TritonX‐100 in 1×PBS for 5 min. After several washes in 1×PBS and blocking, cells were incubated with primary antibodies diluted in blocking buffer (1×PBS with 0.1% Tween‐20, 2% BSA, 5% horse serum) for 1 h followed by washing, incubation with fluorochrome‐conjugated secondary antibodies and Hoechst contrastain for 30 min and embedding with Vectashield (Vector Ltd.). Details on primary antibodies are listed in Appendix Table S2. Images were captured using confocal microscopy (Zeiss LSM 700 point laser confocal microscope with laser excitation, using either 40X, Plan‐NeoFluar NA 1.3 Oil or 63X Plan‐pochromat NA 1.4 Oil immersion objectives and default (72 μm) pinhole for single frames).

### Western blot analysis

Keratinocyte cultures were lysed in RIPA buffer (150 mM NaCl, 50 mM Tris pH 7.4, 1% Nonidet P40, 1% Na‐deoxycholate, 1 mM EDTA, 1 mM Na_3_VO_4_, 25 mM NaF) supplemented with Complete protease inhibitor cocktail (Roche). Skin tissues were homogenized with a Precellys 24 homogenizer (Bertin) for protein extraction. Total protein quantification in skin lysates was done by Bradford protein assay according to the manufacturer's protocol (Bio‐Rad). After SDS–PAGE proteins were transferred onto PVDF membrane (Immobilon®‐P). Western blot analysis was performed using primary antibodies listed in Appendix Table S2 and HRP‐coupled secondary antibodies (Jackson ImmunoResearch). Blots were developed using Clarity ECL Substrate and Chemidoc Touch device (Bio‐Rad) and quantified using ImageLab software (Bio‐Rad).

### FACS sorting of primary keratinocytes

Single‐cell suspensions of freshly isolated keratinocytes were prepared from epidermal sheet as described previously and subsequently blocked with anti‐CD16/32 antibody (BioLegend). Cells were stained with APC‐conjugated CD45 and FITC‐conjugated Ly6a/Sca1 antibodies (Biolegend) for 30 min at 4°C. After incubation, cells were washed, filtered, and stained with Zombie UV™ viability dye (BioLegend) according to the manufacturer's recommendation to exclude dead cells. Keratinocytes were gated as CD45 negative live cell population and sorted to be enriched for cells of the interfollicular epithelium (IFE; Ly6a/Sca1 positive) and of hair follicles (HF; Ly6a/Sca1 negative) according to (Sakamoto *et al*, 2022) by a *Moflo Astrios EQ cell sorter* (Beckman Coulter) at cooled conditions. Sorted cells were directly pelleted and propagated for RNA isolation.

### ELISA

100 μg of total skin protein extracts prepared in RIPA lysis buffer was diluted in ELISA assay diluent (BioLegend) with Complete protease inhibitor cocktail (Roche), and ELISA was performed for CCL2 and IL‐17A (BD Biosciences) according to the manufacturer's instructions. Absorbance was measured with a Tecan plate reader (Tecan Infinite 200 Pro fluorometer).

### Fluorometric uPA activity assay

uPA activity was determined using the Urokinase Activity Fluorometric Assay kit (Sigma) according to the manufacturer's instructions. Briefly, 200 μg of total skin protein extracts was prepared using the lysis buffer provided in the kit and added to the reaction. Kinetics of absorbance was measured with a Tecan plate reader (Tecan Infinite 200 Pro fluorometer).

### Flow cytometry of total skin and spleen

Single‐cell suspension of freshly isolated splenocytes and total skin was prepared as described earlier (Novoszel *et al*, 2021) and subsequently blocked with anti‐CD16/32 antibody (BioLegend). Samples were stained with following fluorophore‐conjugated primary antibodies (Biolegend) for 30 min at 4°C: Ly6G, CD11b, CD11c, CD19, F4/80, MHC class II, Ly6C, and CD3ε (spleen) and B220, BST‐2, CD3ε, CD11c, CD11b, CD19, F4/80, Ly6C, Ly6G, MHC class II, TCR γδ, and XCR1 (skin, two panels; see Appendix Table S2). After incubation, cells were washed, filtered, and stained with SYTOXblue viability dye (Thermo Fisher) according to the manufacturer's recommendation to exclude dead cells. Flow cytometry was performed on a *Fortessa* (BD Bioscience) and analyzed by FlowJo v10 software.

### Bone marrow isolation

Bone marrow cells were isolated as described previously (Novoszel *et al*, 2021). Pelleted bone marrow cells were directly lysed in RIPA buffer, and western blot analysis was performed.

### RNA extraction and quantitative real‐time PCR

RNA was extracted from murine back skin and from freshly isolated or cultured primary keratinocytes by using TRIzol reagent (Invitrogen). For tissue homogenization, back skin was mechanically disrupted with a Precellys 24 homogenizer (Bertin). cDNA was synthesized by using the ProtoScript^®^ II reverse transcriptase (New England Biolabs) or SuperScript^®^ IV reverse transcriptase (Fischer Scientific) at limiting RNA concentrations. Quantitative real‐time reverse transcription‐PCR was performed with the Power SYBR Green Master Mix (Applied Biosystems). Each step followed the manufacturers' instructions. All primers are listed in Appendix Table S3. Relative quantification of RNA was calculated according to the *ddCt* method using GAPDH as reference gene and acetone‐treated *ILEI* ^*ind*^ skin or keratinocytes as control condition.

### mRNA sequencing

Total RNA isolated from back skin tissue biopsies of treated *ILEI* ^*ind*^ and *K5‐ILEI* ^*ind*^ mice was used for library preparation. The directional library was prepared using NEBNext^®^ UltraTM Directional RNA Library Prep Kit for Illumina^®^ (NEB, USA) following the manufacturer's protocol. Indices were included to multiplex multiple samples. Briefly, mRNA was purified from total RNA using poly‐T oligo‐attached magnetic beads. After fragmentation, the first strand cDNA was synthesized using random hexamer primers followed by the second strand cDNA synthesis. The strand‐specific library was ready after end repair, A‐tailing, adapter ligation, size selection, and USER enzyme digestion. After amplification and purification, insert size of the library was validated on an Agilent 2100 and quantified using quantitative PCR (qPCR). Libraries were then sequenced on Illumina NovaSeq 6000 S4 flowcell with PE150 according to results from library quality control and expected data volume. Library preparation and sequencing was performed by Novogene UK. Fastq files were aligned using kallisto (Bray *et al*, 2016) to the GRCm38 mouse genome.

### Transcriptomic analysis

Human patient data were downloaded from Gene Expression Omnibus (GSE121212) and Array Express (E‐MTAB‐8149). Briefly, GSE121212 and E‐MTAB‐8149 contain biopsies from psoriatic and atopic dermatitis patients, and healthy controls (Fyhrquist *et al*, 2019; Tsoi *et al*, 2019). The count matrices of the GSE121212 human dataset and the in‐house generated mouse dataset were read into GNU R and analyzed for differentially expressed genes using the DESeq2 (Love *et al*, 2014). The list of differentially expressed genes of the mouse dataset was defined as “ILEI signature.” The CEL files of E‐MTAB‐8149 were loaded into R using the oligo package (Carvalho & Irizarry, 2010), rma normalized and subjected to quality control using arrayQualityMetrics (Kauffmann *et al*, 2009) as described earlier (Mohr *et al*, 2021). Differentially expressed genes were determined using LIMMA (Ritchie *et al*, 2015). Biological context of differentially expressed genes was determined using clusterProfiler (Yu *et al*, 2012). Datasets of the Molecular Signature Database (H—hallmark genes, and C5—GO Terms biologic process) were used as input (Subramanian *et al*, 2005; Liberzon *et al*, 2011). Dimensionality reduction and lead term determination was performed as described earlier (Mohr *et al*, 2021).

The ILEI signature was checked for performance using the mixOmics package of R (Rohart *et al*, 2017b). Data were integrated using MINT (Rohart *et al*, 2017a) in combination with partial least square discriminant analysis (MINT‐PLSDA; Rohart *et al*, 2017b) with default parameters. Model optimization resulted in a model using the first three principal components and 32 genes. ROC curves and relevance networks were constructed using the mixOmics package (Rohart *et al*, 2017b). Heatmaps and relevance networks were calculated from the integrated data using 29 genes based on the first component of the model. Where appropriate, P‐values were corrected for multiple testing according to Benjamini–Hochberg (Hochberg & Benjamini, 1990).

### scRNA‐seq analysis

Processed sequencing data reported in (Gao *et al*, 2021) were downloaded from the Gene Expression Omnibus (GEO) database with accession GSE162183. scRNA‐Seq analysis was carried out using most recent version of Seurat functions (Seurat V4.0+; Hao *et al*, 2021; Stuart *et al*, 2019). According to parameters and known markers used in (Gao *et al*, 2021), cells harboring < 200 transcripts, < 1,000 unique molecules and > 5% of mitochondrial gene ratio were removed and the remaining cells were clustered into five main clusters. Identified Epidermis (Ep) cluster was further clustered into Ep_basal & Ep_diff based on the markers KRT14, KRT5, ITGA6 and KRT1, KRT10, FLG, respectively. Dimensionality was further reduced for visualization using t‐Distributed Stochastic Neighbor Embedding (t‐SNE). The MAST packages integrated in the Seurat “FindMarkers” were utilized to run differential expression testing (Luecken & Theis, 2019).

### Statistical analysis

Statistical analysis was done with GraphPad Prism 8.0. Sample size estimate was not statistically performed. To compare one parameter between two groups unpaired and paired two‐tailed Student's *t*‐test, across multiple groups one‐way ANOVA with Tukey multiple comparison test was applied to determine statistical significance. Welch's correction was performed on Student's *t*‐test, if variance between groups was significantly different. When possible, normality of data was assessed with D'Agostino and Pearson test, outliers were identified by Grubbs´ test or ROUT Method. P‐values of lower than 0.05 were considered statistically significant (*P < 0.05, **P < 0.01, ***P < 0.001). Error bars are represented as standard error of mean (SEM). The strength of relationship between two variables was calculated by Pearson correlation analysis, linear regression curves calculated and presented with 99% confidence intervals.

## Author contributions

**Barizah Malik:** Investigation; writing – original draft. **Iva Vokic:** Investigation. **Thomas Mohr:** Data curation; formal analysis; writing – review and editing. **Marle Poppelaars:** Investigation. **Martin Holcmann:** Formal analysis; investigation; writing – review and editing. **Philipp Novoszel:** Resources; investigation; writing – review and editing. **Gerald Timelthaler:** Formal analysis. **Thomas Lendl:** Software; methodology. **Dana Krauss:** Formal analysis. **Ulrich Elling:** Methodology. **Michael Mildner:** Resources. **Josef M Penninger:** Resources. **Peter Petzelbauer:** Resources. **Maria Sibilia:** Resources; supervision. **Agnes Csiszar:** Conceptualization; supervision; funding acquisition; writing – original draft.

## Disclosure and competing interests statement

The authors declare that they have no conflict of interest.

## Supporting information



Appendix

Expanded View Figures PDF

Source Data for Appendix

PDF+

Source Data for Figure 1

Source Data for Figure 2

Source Data for Figure 3

Source Data for Figure 4

Source Data for Figure 5

Source Data for Figure 6

Source Data for Figure 7

## Data Availability

The datasets produced in this study are available in the following database: RNAseq data: ArrayExpress/Biostudies E‐MTAB‐12849 (https://www.ebi.ac.uk/biostudies/arrayexpress/studies/E‐MTAB‐12849).

## References

[emmm202216758-bib-0001] Akira S (1997) IL‐6‐regulated transcription factors. Int J Biochem Cell Biol 29: 1401–1418 9570135 10.1016/s1357-2725(97)00063-0

[emmm202216758-bib-0002] Andres RM , Hald A , Johansen C , Kragballe K , Iversen L (2013) Studies of Jak/STAT3 expression and signalling in psoriasis identifies STAT3‐Ser727 phosphorylation as a modulator of transcriptional activity. Exp Dermatol 22: 323–328 23614738 10.1111/exd.12128

[emmm202216758-bib-0003] Balic JJ , Albargy H , Luu K , Kirby FJ , Jayasekara WSN , Mansell F , Garama DJ , De Nardo D , Baschuk N , Louis C *et al* (2020) STAT3 serine phosphorylation is required for TLR4 metabolic reprogramming and IL‐1beta expression. Nat Commun 11: 3816 32732870 10.1038/s41467-020-17669-5 PMC7393113

[emmm202216758-bib-0004] Bevan P , Mala C (2008) The role of uPA and uPA inhibitors in breast cancer. Breast Care 3: 1–2 10.1159/000151735 PMC2930994 20824006

[emmm202216758-bib-0005] Blanpain C , Fuchs E (2006) Epidermal stem cells of the skin. Annu Rev Cell Dev Biol 22: 339–373 16824012 10.1146/annurev.cellbio.22.010305.104357 PMC2405915

[emmm202216758-bib-0006] Boccaccio C , Andò M , Tamagnone L , Bardelli A , Michieli P , Battistini C , Comoglio PM (1998) Induction of epithelial tubules by growth factor HGF depends on the STAT pathway. Nature 391: 285–288 9440692 10.1038/34657

[emmm202216758-bib-0007] Bray NL , Pimentel H , Melsted P , Pachter L (2016) Near‐optimal probabilistic RNA‐seq quantification. Nat Biotechnol 34: 525–527 27043002 10.1038/nbt.3519

[emmm202216758-bib-0008] Cai Y , Shen X , Ding C , Qi C , Li K , Li X , Jala VR , Zhang HG , Wang T , Zheng J *et al* (2011) Pivotal role of dermal IL‐17‐producing gammadelta T cells in skin inflammation. Immunity 35: 596–610 21982596 10.1016/j.immuni.2011.08.001 PMC3205267

[emmm202216758-bib-0009] Calautti E , Avalle L , Poli V (2018) Psoriasis: a STAT3‐centric view. Int J Mol Sci 19: 171 29316631 10.3390/ijms19010171 PMC5796120

[emmm202216758-bib-0010] Campa M , Ryan C , Menter A (2015) An overview of developing TNF‐α targeted therapy for the treatment of psoriasis. Expert Opin Investig Drugs 24: 1343–1354 10.1517/13543784.2015.1076793 26289788

[emmm202216758-bib-0011] Carvalho BS , Irizarry RA (2010) A framework for oligonucleotide microarray preprocessing. Bioinformatics 26: 2363–2367 20688976 10.1093/bioinformatics/btq431 PMC2944196

[emmm202216758-bib-0012] Chaudhury A , Hussey GS , Ray PS , Jin G , Fox PL , Howe PH (2010a) TGF‐beta‐mediated phosphorylation of hnRNP E1 induces EMT via transcript‐selective translational induction of Dab2 and ILEI. Nat Cell Biol 12: 286–293 20154680 10.1038/ncb2029 PMC2830561

[emmm202216758-bib-0013] Chaudhury A , Hussey GS , Ray PS , Jin G , Fox PL , Howe PH (2010b) TGF‐β‐mediated phosphorylation of hnRNP E1 induces EMT via transcript‐selective translational induction of Dab2 and ILEI. Nat Cell Biol 12: 286–293 20154680 10.1038/ncb2029 PMC2830561

[emmm202216758-bib-0014] Chen Z , Ding L , Yang W , Wang J , Chen L , Chang Y , Geng B , Cui Q , Guan Y , Yang J (2017) Hepatic activation of the FAM3C‐HSF1‐CaM pathway attenuates hyperglycemia of obese diabetic mice. Diabetes 66: 1185–1197 28246289 10.2337/db16-0993

[emmm202216758-bib-0015] Christophers E (2001) Psoriasis − epidemiology and clinical spectrum. Clin Exp Dermatol 26: 314–320 11422182 10.1046/j.1365-2230.2001.00832.x

[emmm202216758-bib-0016] Csiszar A , Kutay B , Wirth S , Schmidt U , Macho‐Maschler S , Schreiber M , Alacakaptan M , Vogel GF , Aumayr K , Huber LA (2014) Interleukin‐like epithelial‐to‐mesenchymal transition inducer activity is controlled by proteolytic processing and plasminogen–urokinase plasminogen activator receptor system–regulated secretion during breast cancer progression. Breast Cancer Res 16: 1–18 10.1186/s13058-014-0433-7 PMC4303039 25212966

[emmm202216758-bib-0017] Darnell JE (1997) STATs and gene regulation. Science 277: 1630–1635 9287210 10.1126/science.277.5332.1630

[emmm202216758-bib-0018] Dong C (2006) Diversification of T‐helper‐cell lineages: finding the family root of IL‐17‐producing cells. Nat Rev Immunol 6: 329–334 16557264 10.1038/nri1807

[emmm202216758-bib-0019] Ellinghaus D , Ellinghaus E , Nair RP , Stuart PE , Esko T , Metspalu A , Debrus S , Raelson JV , Tejasvi T , Belouchi M *et al* (2012) Combined analysis of genome‐wide association studies for Crohn disease and psoriasis identifies seven shared susceptibility loci. Am J Hum Genet 90: 636–647 22482804 10.1016/j.ajhg.2012.02.020 PMC3322238

[emmm202216758-bib-0020] Fish PV , Barber CG , Brown DG , Butt R , Collis MG , Dickinson RP , Henry BT , Horne VA , Huggins JP , King E *et al* (2007) Selective urokinase‐type plasminogen activator inhibitors. 4. 1‐(7‐sulfonamidoisoquinolinyl)guanidines. J Med Chem 50: 2341–2351 17447747 10.1021/jm061066t

[emmm202216758-bib-0021] Fyhrquist N , Muirhead G , Prast‐Nielsen S , Jeanmougin M , Olah P , Skoog T , Jules‐Clement G , Feld M , Barrientos‐Somarribas M , Sinkko H (2019) Microbe‐host interplay in atopic dermatitis and psoriasis. Nat Commun 10: 1–15 31619666 10.1038/s41467-019-12253-y PMC6795799

[emmm202216758-bib-0022] Gao ZH , Lu C , Wang ZN , Song YX , Zhu JL , Gao P , Sun JX , Chen XW , Wang MX , Dong YL (2014) ILEI: a novel marker for epithelial–mesenchymal transition and poor prognosis in colorectal cancer. Histopathology 65: 527–538 24738665 10.1111/his.12435

[emmm202216758-bib-0023] Gao Y , Yao X , Zhai Y , Li L , Li H , Sun X , Yu P , Xue T , Li Y , Hu Y (2021) Single cell transcriptional zonation of human psoriasis skin identifies an alternative immunoregulatory axis conducted by skin resident cells. Cell Death Dis 12: 450 33958582 10.1038/s41419-021-03724-6 PMC8102483

[emmm202216758-bib-0024] Gelfand JM , Troxel AB , Lewis JD , Kurd SK , Shin DB , Wang X , Margolis DJ , Strom BL (2007) The risk of mortality in patients with psoriasis: results from a population‐based study. Arch Dermatol 143: 1493–1499 18086997 10.1001/archderm.143.12.1493

[emmm202216758-bib-0025] Georgiades P , Ogilvy S , Duval H , Licence DR , Charnock‐Jones DS , Smith SK , Print CG (2002) VavCre transgenic mice: a tool for mutagenesis in hematopoietic and endothelial lineages. Genesis 34: 251–256 12434335 10.1002/gene.10161

[emmm202216758-bib-0026] Gong C , Zhang Y , Shankaran H , Resat H (2015) Integrated analysis reveals that STAT3 is central to the crosstalk between HER/ErbB receptor signaling pathways in human mammary epithelial cells. Mol Biosyst 11: 146–158 25315124 10.1039/c4mb00471j PMC4540226

[emmm202216758-bib-0027] Gudjonsson JE , Johnston A , Dyson M , Valdimarsson H , Elder JT (2007) Mouse models of psoriasis. J Invest Dermatol 127: 1292–1308 17429444 10.1038/sj.jid.5700807

[emmm202216758-bib-0028] Guinea‐Viniegra J , Zenz R , Scheuch H , Hnisz D , Holcmann M , Bakiri L , Schonthaler HB , Sibilia M , Wagner EF (2009) TNFalpha shedding and epidermal inflammation are controlled by Jun proteins. Genes Dev 23: 2663–2674 19933155 10.1101/gad.543109 PMC2779759

[emmm202216758-bib-0029] Halberg N , Sengelaub CA , Navrazhina K , Molina H , Uryu K , Tavazoie SF (2016) PITPNC1 recruits RAB1B to the Golgi network to drive malignant secretion. Cancer Cell 29: 339–353 26977884 10.1016/j.ccell.2016.02.013 PMC5300038

[emmm202216758-bib-0030] Han G , Williams CA , Salter K , Garl PJ , Li AG , Wang XJ (2010) A role for TGFbeta signaling in the pathogenesis of psoriasis. J Invest Dermatol 130: 371–377 19710682 10.1038/jid.2009.252 PMC2898194

[emmm202216758-bib-0031] Hao Y , Hao S , Andersen-Nissen E , Mauck 3rd WM , Zheng S , Butler A , Lee MJ , Wilk AJ , Darby C , Zager M *et al* (2021) Integrated analysis of multimodal single-cell data. Cell 184: 3573–3587.e29 34062119 10.1016/j.cell.2021.04.048 PMC8238499

[emmm202216758-bib-0032] Henry CM , Sullivan GP , Clancy DM , Afonina IS , Kulms D , Martin SJ (2016) Neutrophil‐derived proteases escalate inflammation through activation of IL‐36 family cytokines. Cell Rep 14: 708–722 26776523 10.1016/j.celrep.2015.12.072

[emmm202216758-bib-0033] Hochberg Y , Benjamini Y (1990) More powerful procedures for multiple significance testing. Stat Med 9: 811–818 2218183 10.1002/sim.4780090710

[emmm202216758-bib-0034] Hollox EJ , Huffmeier U , Zeeuwen PL , Palla R , Lascorz J , Rodijk‐Olthuis D , van de Kerkhof PC , Traupe H , de Jongh G , den Heijer M *et al* (2008) Psoriasis is associated with increased beta‐defensin genomic copy number. Nat Genet 40: 23–25 18059266 10.1038/ng.2007.48 PMC2447885

[emmm202216758-bib-0035] Jansson AM , Csiszar A , Maier J , Nyström A‐C , Ax E , Johansson P , Schiavone LH (2017) The interleukin‐like epithelial‐mesenchymal transition inducer ILEI exhibits a non‐interleukin‐like fold and is active as a domain‐swapped dimer. J Biol Chem 292: 15501–15511 28751379 10.1074/jbc.M117.782904 PMC5602407

[emmm202216758-bib-0036] Ji YZ , Liu SR (2019) Koebner phenomenon leading to the formation of new psoriatic lesions: evidences and mechanisms. Biosci Rep 39: BSR20193266 31710084 10.1042/BSR20193266 PMC6893164

[emmm202216758-bib-0037] Johnston A , Fritz Y , Dawes SM , Diaconu D , Al‐Attar PM , Guzman AM , Chen CS , Fu W , Gudjonsson JE , McCormick TS *et al* (2013) Keratinocyte overexpression of IL‐17C promotes psoriasiform skin inflammation. J Immunol 190: 2252–2262 23359500 10.4049/jimmunol.1201505 PMC3577967

[emmm202216758-bib-0038] de Jongh GJ , Zeeuwen PL , Kucharekova M , Pfundt R , van der Valk PG , Blokx W , Dogan A , Hiemstra PS , van de Kerkhof PC , Schalkwijk J (2005) High expression levels of keratinocyte antimicrobial proteins in psoriasis compared with atopic dermatitis. J Invest Dermatol 125: 1163–1173 16354186 10.1111/j.0022-202X.2005.23935.x

[emmm202216758-bib-0039] Kauffmann A , Gentleman R , Huber W (2009) arrayQualityMetrics—a bioconductor package for quality assessment of microarray data. Bioinformatics 25: 415–416 19106121 10.1093/bioinformatics/btn647 PMC2639074

[emmm202216758-bib-0040] Kral M , Klimek C , Kutay B , Timelthaler G , Lendl T , Neuditschko B , Gerner C , Sibilia M , Csiszar A (2017) Covalent dimerization of interleukin‐like epithelial‐to‐mesenchymal transition (EMT) inducer (ILEI) facilitates EMT, invasion, and late aspects of metastasis. FEBS J 284: 3484–3505 28837266 10.1111/febs.14207

[emmm202216758-bib-0041] Krasavin MY , Gureev MA , Garabadzhiu AV , Pashkin AY , Zhukov AS , Khairutdinov VR , Samtsov AV , Shvets VI (2019) Inhibition of neutrophil elastase and cathepsin G As a new approach to the treatment of psoriasis: from fundamental biology to development of new target‐specific drugs. Dokl Biochem Biophys 487: 272–276 31559596 10.1134/S1607672919040082

[emmm202216758-bib-0042] Lahsnig C , Mikula M , Petz M , Zulehner G , Schneller D , Van Zijl F , Huber H , Csiszar A , Beug H , Mikulits W (2009) ILEI requires oncogenic Ras for the epithelial to mesenchymal transition of hepatocytes and liver carcinoma progression. Oncogene 28: 638–650 19015638 10.1038/onc.2008.418 PMC2900603

[emmm202216758-bib-0043] Lebwohl M , Strober B , Menter A , Gordon K , Weglowska J , Puig L , Papp K , Spelman L , Toth D , Kerdel F (2015) Phase 3 studies comparing brodalumab with ustekinumab in psoriasis. N Engl J Med 373: 1318–1328 26422722 10.1056/NEJMoa1503824

[emmm202216758-bib-0044] Leigh I , Navsaria H , Purkis P , McKay I , Bowden P , Riddle P (1995) Keratins (Kl6 and Kl7) as markers of keratinocyte hyperproliferation in psoriasis *in vivo* and *in vitro* . Br J Dermatol 133: 501–511 7577575 10.1111/j.1365-2133.1995.tb02696.x

[emmm202216758-bib-0045] Li Q , Ke F , Zhang W , Shen X , Xu Q , Wang H , Yu XZ , Leng Q , Wang H (2011) Plasmin plays an essential role in amplification of psoriasiform skin inflammation in mice. PloS one 6: e16483 21311769 10.1371/journal.pone.0016483 PMC3032787

[emmm202216758-bib-0046] Liberzon A , Subramanian A , Pinchback R , Thorvaldsdóttir H , Tamayo P , Mesirov JP (2011) Molecular signatures database (MSigDB) 3.0. Bioinformatics 27: 1739–1740 21546393 10.1093/bioinformatics/btr260 PMC3106198

[emmm202216758-bib-0047] Lichtenberger BM , Tan PK , Niederleithner H , Ferrara N , Petzelbauer P , Sibilia M (2010) Autocrine VEGF signaling synergizes with EGFR in tumor cells to promote epithelial cancer development. Cell 140: 268–279 20141840 10.1016/j.cell.2009.12.046

[emmm202216758-bib-0048] Lichtenberger BM , Gerber PA , Holcmann M , Buhren BA , Amberg N , Smolle V , Schrumpf H , Boelke E , Ansari P , Mackenzie C (2013) Epidermal EGFR controls cutaneous host defense and prevents inflammation. Sci Transl Med 5: 199ra111 10.1126/scitranslmed.3005886 23966300

[emmm202216758-bib-0049] Lin WH , Chang YW , Hong MX , Hsu TC , Lee KC , Lin C , Lee JL (2021) STAT3 phosphorylation at Ser727 and Tyr705 differentially regulates the EMT‐MET switch and cancer metastasis. Oncogene 40: 791–805 33262462 10.1038/s41388-020-01566-8 PMC7843420

[emmm202216758-bib-0050] Lindhaus C , Tittelbach J , Elsner P (2017) Cutaneous side effects of TNF‐alpha inhibitors. JDDG. J Dtsch Dermatol Ges 15: 281–288 10.1111/ddg.13200 28252861

[emmm202216758-bib-0051] Love MI , Huber W , Anders S (2014) Moderated estimation of fold change and dispersion for RNA-seq data with DESeq2. Genome Biol 15: 550 25516281 10.1186/s13059-014-0550-8 PMC4302049

[emmm202216758-bib-0052] Luecken MD , Theis FJ (2019) Current best practices in single-cell RNA-seq analysis: a tutorial. Mol Syst Biol 15: e8746 31217225 10.15252/msb.20188746 PMC6582955

[emmm202216758-bib-0053] Lund LR , Green KA , Stoop AA , Ploug M , Almholt K , Lilla J , Nielsen BS , Christensen IJ , Craik CS , Werb Z *et al* (2006) Plasminogen activation independent of uPA and tPA maintains wound healing in gene‐deficient mice. EMBO J 25: 2686–2697 16763560 10.1038/sj.emboj.7601173 PMC1500865

[emmm202216758-bib-0054] Masucci MT , Minopoli M , Di Carluccio G , Motti ML , Carriero MV (2022) Therapeutic strategies targeting urokinase and its receptor in cancer. Cancer 14: 498 10.3390/cancers14030498 PMC8833673 35158766

[emmm202216758-bib-0055] Mohr T , Katz S , Paulitschke V , Aizarani N , Tolios A (2021) Systematic analysis of the transcriptome profiles and co‐expression networks of tumour endothelial cells identifies several tumour‐associated modules and potential therapeutic targets in hepatocellular carcinoma. Cancer 13: 1768 10.3390/cancers13081768 PMC8067977 33917186

[emmm202216758-bib-0056] Motani K , Kosako H (2018) Activation of stimulator of interferon genes (STING) induces ADAM17‐mediated shedding of the immune semaphorin SEMA4D. J Biol Chem 293: 7717–7726 29618514 10.1074/jbc.RA118.002175 PMC5961039

[emmm202216758-bib-0057] Murdaca G , Negrini S , Pellecchio M , Greco M , Schiavi C , Giusti F , Puppo F (2019) Update upon the infection risk in patients receiving TNF alpha inhibitors. Expert Opin Drug Saf 18: 219–229 30704314 10.1080/14740338.2019.1577817

[emmm202216758-bib-0058] Nakajima K , Sano S (2018) Mouse models of psoriasis and their relevance. J Dermatol 45: 252–263 29226571 10.1111/1346-8138.14112

[emmm202216758-bib-0059] Nickoloff BJ , Qin J‐Z , Nestle FO (2007) Immunopathogenesis of psoriasis. Clin Rev Allergy Immunol 33: 45–56 18094946 10.1007/s12016-007-0039-2

[emmm202216758-bib-0060] Novoszel P , Holcmann M , Stulnig G , De Sa FC , Zyulina V , Borek I , Linder M , Bogusch A , Drobits B , Bauer T *et al* (2021) Psoriatic skin inflammation is promoted by c‐Jun/AP‐1‐dependent CCL2 and IL‐23 expression in dendritic cells. EMBO Mol Med 13: e12409 33724710 10.15252/emmm.202012409 PMC8033525

[emmm202216758-bib-0061] Ogawa E , Sato Y , Minagawa A , Okuyama R (2018) Pathogenesis of psoriasis and development of treatment. J Dermatol 45: 264–272 29226422 10.1111/1346-8138.14139

[emmm202216758-bib-0062] Papp KA , Reid C , Foley P , Sinclair R , Salinger DH , Williams G , Dong H , Krueger JG , Russell CB , Martin DA (2012) Anti‐IL‐17 receptor antibody AMG 827 leads to rapid clinical response in subjects with moderate to severe psoriasis: results from a phase I, randomized, placebo‐controlled trial. J Invest Dermatol 132: 2466–2469 22622425 10.1038/jid.2012.163

[emmm202216758-bib-0063] Parker BL , Thaysen‐Andersen M , Fazakerley DJ , Holliday M , Packer NH , James DE (2016) Terminal galactosylation and sialylation switching on membrane glycoproteins upon TNF‐alpha‐induced insulin resistance in adipocytes. Mol Cell Proteomics 15: 141–153 26537798 10.1074/mcp.M115.054221 PMC4762517

[emmm202216758-bib-0064] Ritchie ME , Phipson B , Wu D , Hu Y , Law CW , Shi W , Smyth GK (2015) Limma powers differential expression analyses for RNA‐sequencing and microarray studies. Nucleic Acids Res 43: e47 25605792 10.1093/nar/gkv007 PMC4402510

[emmm202216758-bib-0065] Rohart F , Eslami A , Matigian N , Bougeard S , Le Cao KA (2017a) MINT: a multivariate integrative method to identify reproducible molecular signatures across independent experiments and platforms. BMC Bioinformatics 18: 128 28241739 10.1186/s12859-017-1553-8 PMC5327533

[emmm202216758-bib-0066] Rohart F , Gautier B , Singh A , Le Cao KA (2017b) mixOmics: an R package for 'omics feature selection and multiple data integration. PLoS Comput Biol 13: e1005752 29099853 10.1371/journal.pcbi.1005752 PMC5687754

[emmm202216758-bib-0067] Rubina K , Sysoeva VY , Zagorujko E , Tsokolaeva Z , Kurdina M , Parfyonova YV , Tkachuk V (2017) Increased expression of uPA, uPAR, and PAI‐1 in psoriatic skin and in basal cell carcinomas. Arch Dermatol Res 309: 433–442 28429105 10.1007/s00403-017-1738-z

[emmm202216758-bib-0068] Russell CB , Rand H , Bigler J , Kerkof K , Timour M , Bautista E , Krueger JG , Salinger DH , Welcher AA , Martin DA (2014) Gene expression profiles normalized in psoriatic skin by treatment with brodalumab, a human anti‐IL‐17 receptor monoclonal antibody. J Immunol 192: 3828–3836 24646743 10.4049/jimmunol.1301737

[emmm202216758-bib-0069] Sachen KL , Arnold Greving CN , Towne JE (2022) Role of IL‐36 cytokines in psoriasis and other inflammatory skin conditions. Cytokine 156: 155897 35679693 10.1016/j.cyto.2022.155897

[emmm202216758-bib-0070] Sakamoto K , Goel S , Funakoshi A , Honda T , Nagao K (2022) Flow cytometry analysis of the subpopulations of mouse keratinocytes and skin immune cells. STAR Protoc 3: 101052 34977690 10.1016/j.xpro.2021.101052 PMC8689364

[emmm202216758-bib-0071] Sano S , Itami S , Takeda K , Tarutani M , Yamaguchi Y , Miura H , Yoshikawa K , Akira S , Takeda J (1999) Keratinocyte‐specific ablation of Stat3 exhibits impaired skin remodeling, but does not affect skin morphogenesis. EMBO J 18: 4657–4668 10469645 10.1093/emboj/18.17.4657 PMC1171539

[emmm202216758-bib-0072] Sano S , Chan KS , Carbajal S , Clifford J , Peavey M , Kiguchi K , Itami S , Nickoloff BJ , DiGiovanni J (2005) Stat3 links activated keratinocytes and immunocytes required for development of psoriasis in a novel transgenic mouse model. Nat Med 11: 43–49 15592573 10.1038/nm1162

[emmm202216758-bib-0073] Schmidt U , Heller G , Timelthaler G , Heffeter P , Somodi Z , Schweifer N , Sibilia M , Berger W , Csiszar A (2021) The FAM3C locus that encodes interleukin‐like EMT inducer (ILEI) is frequently co‐amplified in MET‐amplified cancers and contributes to invasiveness. J Exp Clin Cancer Res 40: 69 33596971 10.1186/s13046-021-01862-5 PMC7890988

[emmm202216758-bib-0074] Schmidt U , Uluca B , Vokic Malik B , Kolbe T , Lassnig C , Holcmann M , Moreno-Viedma V , Robl B , Mühlberger C *et al* (2023) Inducible overexpression of a FAM3C/ILEI transgene has pleiotropic effects with shortened life span, liver fibrosis and anemia in mice. PlosONE 10.1371/journal.pone.0286256 PMC10503705 37713409

[emmm202216758-bib-0075] Schonthaler HB , Guinea‐Viniegra J , Wculek SK , Ruppen I , Ximenez‐Embun P , Guio‐Carrion A , Navarro R , Hogg N , Ashman K , Wagner EF (2013) S100A8‐S100A9 protein complex mediates psoriasis by regulating the expression of complement factor C3. Immunity 39: 1171–1181 24332034 10.1016/j.immuni.2013.11.011

[emmm202216758-bib-0076] Smith HW , Marshall CJ (2010) Regulation of cell signalling by uPAR. Nat Rev Mol Cell Biol 11: 23–36 20027185 10.1038/nrm2821

[emmm202216758-bib-0077] Sotiropoulou G , Pampalakis G , Diamandis EP (2009) Functional roles of human kallikrein‐related peptidases. J Biol Chem 284: 32989–32994 19819870 10.1074/jbc.R109.027946 PMC2785139

[emmm202216758-bib-0078] Stanley PL , Steiner S , Havens M , Tramposch KM (1991) Mouse skin inflammation induced by multiple topical applications of 12‐O‐tetradecanoylphorbol‐13‐acetate. Skin Pharmacol 4: 262–271 1789987 10.1159/000210960

[emmm202216758-bib-0079] Stuart T , Butler A , Hoffman P , Hafemeister C , Papalexi E , Mauck 3rd WM , Hao Y , Stoeckius M , Smibert P , Satija R (2019) Comprehensive integration of single-cell data. Cell 177: 1888–1902.e21 31178118 10.1016/j.cell.2019.05.031 PMC6687398

[emmm202216758-bib-0080] Subramanian A , Tamayo P , Mootha VK , Mukherjee S , Ebert BL , Gillette MA , Paulovich A , Pomeroy SL , Golub TR , Lander ES (2005) Gene set enrichment analysis: a knowledge‐based approach for interpreting genome‐wide expression profiles. Proc Natl Acad Sci USA 102: 15545–15550 16199517 10.1073/pnas.0506580102 PMC1239896

[emmm202216758-bib-0081] Swindell WR , Johnston A , Carbajal S , Han G , Wohn C , Lu J , Xing X , Nair RP , Voorhees JJ , Elder JT *et al* (2011) Genome‐wide expression profiling of five mouse models identifies similarities and differences with human psoriasis. PloS one 6: e18266 21483750 10.1371/journal.pone.0018266 PMC3070727

[emmm202216758-bib-0082] Takeshita J , Grewal S , Langan SM , Mehta NN , Ogdie A , Van Voorhees AS , Gelfand JM (2017) Psoriasis and comorbid diseases: epidemiology. J Am Acad Dermatol 76: 377–390 28212759 10.1016/j.jaad.2016.07.064 PMC5731650

[emmm202216758-bib-0083] Tan X , Banerjee P , Shi L , Xiao GY , Rodriguez BL , Grzeskowiak CL , Liu X , Yu J , Gibbons DL , Russell WK *et al* (2021) p53 loss activates prometastatic secretory vesicle biogenesis in the Golgi. Sci Adv 7: eabf4885 34144984 10.1126/sciadv.abf4885 PMC8213221

[emmm202216758-bib-0084] Tarutani M , Itami S , Okabe M , Ikawa M , Tezuka T , Yoshikawa K , Kinoshita T , Takeda J (1997) Tissue‐specific knockout of the mouse pig‐a gene reveals important roles for GPI‐anchored proteins in skin development. Proc Natl Acad Sci USA 94: 7400–7405 9207103 10.1073/pnas.94.14.7400 PMC23833

[emmm202216758-bib-0085] Tretina K , Park ES , Maminska A , MacMicking JD (2019) Interferon‐induced guanylate‐binding proteins: guardians of host defense in health and disease. J Exp Med 216: 482–500 30755454 10.1084/jem.20182031 PMC6400534

[emmm202216758-bib-0086] Tsoi LC , Spain SL , Knight J , Ellinghaus E , Stuart PE , Capon F , Ding J , Li Y , Tejasvi T , Gudjonsson JE *et al* (2012) Identification of 15 new psoriasis susceptibility loci highlights the role of innate immunity. Nat Genet 44: 1341–1348 23143594 10.1038/ng.2467 PMC3510312

[emmm202216758-bib-0087] Tsoi LC , Rodriguez E , Degenhardt F , Baurecht H , Wehkamp U , Volks N , Szymczak S , Swindell WR , Sarkar MK , Raja K (2019) Atopic dermatitis is an IL‐13–dominant disease with greater molecular heterogeneity compared to psoriasis. J Invest Dermatol 139: 1480–1489 30641038 10.1016/j.jid.2018.12.018 PMC6711380

[emmm202216758-bib-0088] Uribe‐Herranz M , Lian L‐H , Hooper KM , Milora KA , Jensen LE (2013) IL‐1R1 signaling facilitates Munro's microabscess formation in psoriasiform imiquimod‐induced skin inflammation. J Invest Dermatol 133: 1541–1549 23407395 10.1038/jid.2012.512 PMC3656131

[emmm202216758-bib-0089] Van der Fits L , Mourits S , Voerman JS , Kant M , Boon L , Laman JD , Cornelissen F , Mus A‐M , Florencia E , Prens EP (2009) Imiquimod‐induced psoriasis‐like skin inflammation in mice is mediated via the IL‐23/IL‐17 axis. J Immunol 182: 5836–5845 19380832 10.4049/jimmunol.0802999

[emmm202216758-bib-0090] Vitale‐Cross L , Amornphimoltham P , Fisher G , Molinolo AA , Gutkind JS (2004) Conditional expression of K‐ras in an epithelial compartment that includes the stem cells is sufficient to promote squamous cell carcinogenesis. Cancer Res 64: 8804–8807 15604235 10.1158/0008-5472.CAN-04-2623

[emmm202216758-bib-0091] Waerner T , Alacakaptan M , Tamir I , Oberauer R , Gal A , Brabletz T , Schreiber M , Jechlinger M , Beug H (2006) ILEI: a cytokine essential for EMT, tumor formation, and late events in metastasis in epithelial cells. Cancer Cell 10: 227–239 16959614 10.1016/j.ccr.2006.07.020

[emmm202216758-bib-0092] Wagner EF , Schonthaler HB , Guinea‐Viniegra J , Tschachler E (2010) Psoriasis: what we have learned from mouse models. Nat Rev Rheumatol 6: 704–714 20877306 10.1038/nrrheum.2010.157

[emmm202216758-bib-0093] Wang YN , Chang WC (2003) Induction of disease‐associated keratin 16 gene expression by epidermal growth factor is regulated through cooperation of transcription factors Sp1 and c‐Jun. J Biol Chem 278: 45848–45857 12954631 10.1074/jbc.M302630200

[emmm202216758-bib-0094] Wang S , Song R , Wang Z , Jing Z , Wang S , Ma J (2018) S100A8/A9 in inflammation. Front Immunol 9: 1298 29942307 10.3389/fimmu.2018.01298 PMC6004386

[emmm202216758-bib-0095] Wang LX , Zhang SX , Wu HJ , Rong XL , Guo J (2019a) M2b macrophage polarization and its roles in diseases. J Leukoc Biol 106: 345–358 30576000 10.1002/JLB.3RU1018-378RR PMC7379745

[emmm202216758-bib-0096] Wang S , Zhang Z , Peng H , Zeng K (2019b) Recent advances on the roles of epidermal growth factor receptor in psoriasis. Am J Transl Res 11: 520–528 30899359 PMC6413281

[emmm202216758-bib-0097] Wasilewska A , Winiarska M , Olszewska M , Rudnicka L (2016) Interleukin‐17 inhibitors. A new era in treatment of psoriasis and other skin diseases. Postepy Dermatol Alergol 33: 247–252 27605893 10.5114/ada.2016.61599 PMC5004212

[emmm202216758-bib-0098] Wen Z , Zhong Z , Darnell JE Jr (1995) Maximal activation of transcription by Statl and Stat3 requires both tyrosine and serine phosphorylation. Cell 82: 241–250 7543024 10.1016/0092-8674(95)90311-9

[emmm202216758-bib-0099] Woosley AN , Dalton AC , Hussey GS , Howley BV , Mohanty BK , Grelet S , Dincman T , Bloos S , Olsen SK , Howe PH (2019) TGFβ promotes breast cancer stem cell self‐renewal through an ILEI/LIFR signaling axis. Oncogene 38: 3794–3811 30692635 10.1038/s41388-019-0703-z PMC6525020

[emmm202216758-bib-0100] Yang W , Feng B , Meng Y , Wang J , Geng B , Cui Q , Zhang H , Yang Y , Yang J (2019) FAM3C‐YY1 axis is essential for TGFbeta‐promoted proliferation and migration of human breast cancer MDA‐MB‐231 cells via the activation of HSF1. J Cell Mol Med 23: 3464–3475 30887707 10.1111/jcmm.14243 PMC6484506

[emmm202216758-bib-0101] Yin S , Chen F , Ye P , Yang G (2018) Overexpression of FAM3C protein as a novel biomarker for epithelial‐mesenchymal transition and poor outcome in gastric cancer. Int J Clin Exp Pathol 11: 4247 31949820 PMC6962811

[emmm202216758-bib-0102] Yiu ZZN , Barker J , Barnes MR , Di Meglio P , Emsley R , Reynolds NJ , Smith CH , Warren RB , Griffiths CEM , PSORT Consortium (2021) Meeting report: psoriasis stratification to optimize relevant therapy showcase. J Invest Dermatol 141: 1872–1878 33771529 10.1016/j.jid.2021.02.746

[emmm202216758-bib-0103] Yu H , Pardoll D , Jove R (2009) STATs in cancer inflammation and immunity: a leading role for STAT3. Nat Rev Cancer 9: 798–809 19851315 10.1038/nrc2734 PMC4856025

[emmm202216758-bib-0104] Yu G , Wang L‐G , Han Y , He Q‐Y (2012) clusterProfiler: an R package for comparing biological themes among gene clusters. Omics 16: 284–287 22455463 10.1089/omi.2011.0118 PMC3339379

[emmm202216758-bib-0105] Zenz R , Eferl R , Kenner L , Florin L , Hummerich L , Mehic D , Scheuch H , Angel P , Tschachler E , Wagner EF (2005) Psoriasis‐like skin disease and arthritis caused by inducible epidermal deletion of Jun proteins. Nature 437: 369–375 16163348 10.1038/nature03963

[emmm202216758-bib-0106] Zhang LJ , Bhattacharya S , Leid M , Ganguli‐Indra G , Indra AK (2012) Ctip2 is a dynamic regulator of epidermal proliferation and differentiation by integrating EGFR and notch signaling. J Cell Sci 125: 5733–5744 23015591 10.1242/jcs.108969 PMC3575708

[emmm202216758-bib-0107] Zhang X , Yin M , Zhang L‐j (2019) Keratin 6, 16 and 17—critical barrier alarmin molecules in skin wounds and psoriasis. Cell 8: 807 10.3390/cells8080807 PMC6721482 31374826

[emmm202216758-bib-0108] Zhou J , Jiang H , Jiang H , Fan Y , Zhang J , Ma X , Yang X , Sun Y , Zhao X (2022) The ILEI/LIFR complex induces EMT via the Akt and ERK pathways in renal interstitial fibrosis. J Transl Med 20: 1–11 35093095 10.1186/s12967-022-03265-2 PMC8800269

[emmm202216758-bib-0109] Zhu Y , Xu G , Patel A , McLaughlin MM , Silverman C , Knecht KA , Sweitzer S , Li X , McDonnell P , Mirabile R (2002) Cloning, expression, and initial characterization of a novel cytokine‐like gene family. Genomics 80: 144–150 12160727 10.1006/geno.2002.6816

