# Supplementary material for: FAM3C/ILEI protein is elevated in psoriatic lesions and triggers psoriasiform hyperproliferation in mice
Source: EMBO Mol Med. 2023 May 25;15(7):e16758. doi: 10.15252/emmm.202216758 (PMC10331587; doi:10.15252/emmm.202216758)
Supplement: Supplementary file 1 — Appendix [file EMMM-15-e16758-s007.pdf]

# FAM3C/ILEI protein is elevated in psoriatic lesions and triggers psoriasiform hyperproliferation in mice

Barizah Malik[1,§], Iva Vokic[1], Thomas Mohr[1,2,3], Marle Poppelaars[1], Martin Holcmann[1], Philipp Novoszel[1], Gerald Timelthaler[1], Thomas Lendl[4], Dana Krauss[1], Ulrich Elling[5], Michael Mildner[6], Josef M. Penninger[5,7], Peter Petzelbauer[6], Maria Sibilia[1], Agnes Csiszar[1,*]

## Appendix

### Table of Content

Appendix Figure S1 .......... 2
Appendix Figure S2 .......... 4
Appendix Figure S3 .......... 6
Appendix Figure S4 .......... 8
Appendix Figure S5 .......... 10
Appendix Figure S6 .......... 11
Appendix Figure S7 .......... 12
Appendix Figure S8 .......... 14
Appendix Table S1 .......... 15
Appendix Table S2 .......... 16
Appendix Table S3 .......... 17

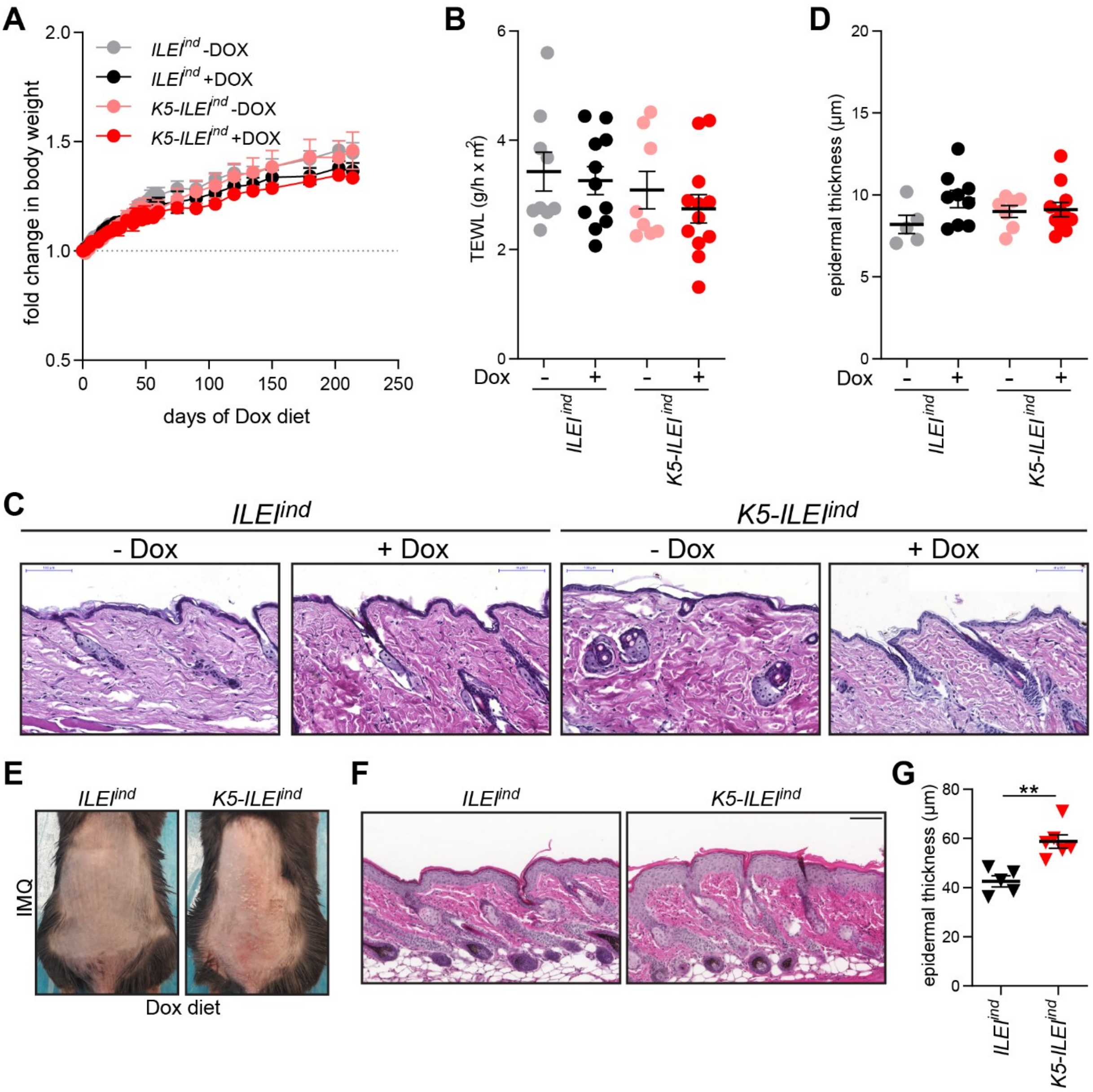


**Appendix Figure S1. Malik et al.**

**Appendix Figure S1. Long-term ILEI overexpression in keratinocytes does not alter overall fitness and homeostatic skin.**

(A) Relative changes of body weight of $ILEI^{ind}$ and $K5\text{-}ILEI^{ind}$ mice kept on normal or doxycycline diet and monitored for 230 days (n=8-12; 4 independent experiments).

(B) Mean trans-epidermal water loss (TEWL) ±SEM of $ILEI^{ind}$ and $K5\text{-}ILEI^{ind}$ mice kept on normal or doxycycline diet for 230 days (n=8-12; 4 independent experiments).

(C) Representative images of hematoxylin-eosin stained thin sections of back skin of $ILEI^{ind}$ and $K5\text{-}ILEI^{ind}$ mice kept on normal or doxycycline diet for 230 days. Scale bar 100 µm.

(D) Mean epidermal thickness ±SEM quantified from hematoxylin-eosin stained thin sections of back skin of $ILEI^{ind}$ and $K5\text{-}ILEI^{ind}$ mice kept on normal or doxycycline diet for 230 days (n=5-10; 4 independent experiments).

(E-G) (E) Macroscopic appearance, (F) hematoxylin-eosin staining and (G) mean epidermal thickness ±SEM of the back skin of $ILEI^{ind}$ and $K5\text{-}ILEI^{ind}$ mice kept on doxycycline diet and treated with imiquimod (IMQ) for 5 days (n=5-6; 2 independent experiments). In G, statistical significance was determined by Student’s t-test and marked with asterisks (**$p<0.01$).

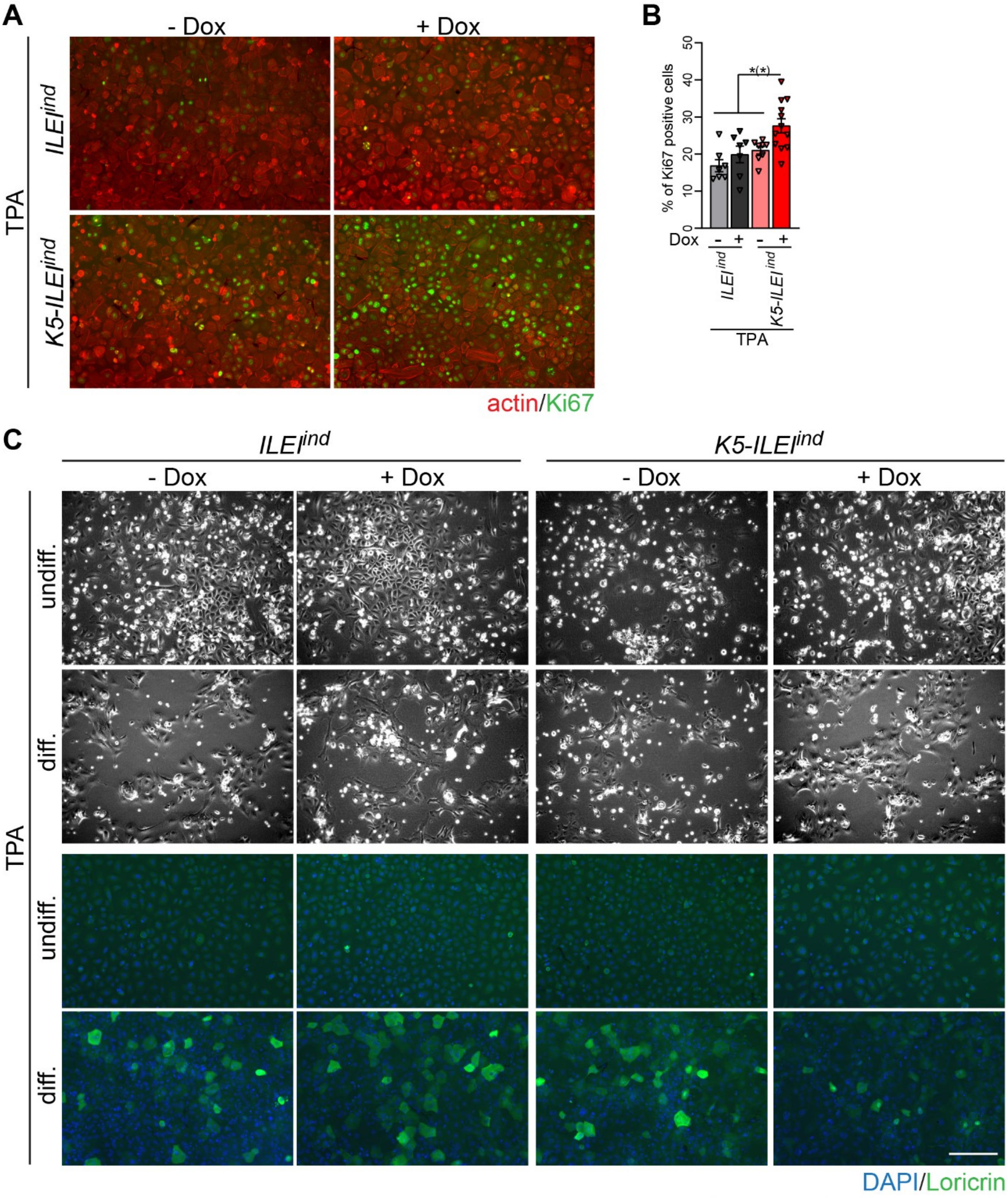


**Appendix Figure S2. Malik et al.**

**Appendix Figure S2. ILEI overexpression contributes to increased proliferation and inhibits differentiation of ex vivo primary keratinocyte cultures after TPA treatment**

(A-B) (A) Representative immunofluorescent microscopic images (Ki67, green; F-actin, red) and (B) mean percentage ±SEM of Ki67 positive cells in primary keratinocyte cultures maintained under TPA treatment with or without doxycycline supplementation for 72 hours after isolation from $ILEI^{ind}$ and $K5\text{-}ILEI^{ind}$ mice kept on normal or doxycycline diet and treated with TPA for 5 days (n=7-12; 4 independent experiments).

(C) Bright field microscopic images (upper panel) and immunofluorescence (lower panel) for Loricrin protein expression on TPA treated, control or doxycycline-supplemented primary keratinocyte cultures with or without calcium-induced differentiation for 72 hours isolated from $ILEI^{ind}$ and $K5\text{-}ILEI^{ind}$ mice. Nuclei are counterstained with DAPI (blue); scale bar, 100 µm. In B, statistical significance was determined by one-way ANOVA with Tukey multiple comparison test and marked with asterisks (*$p<0.05$; **$p<0.01$).

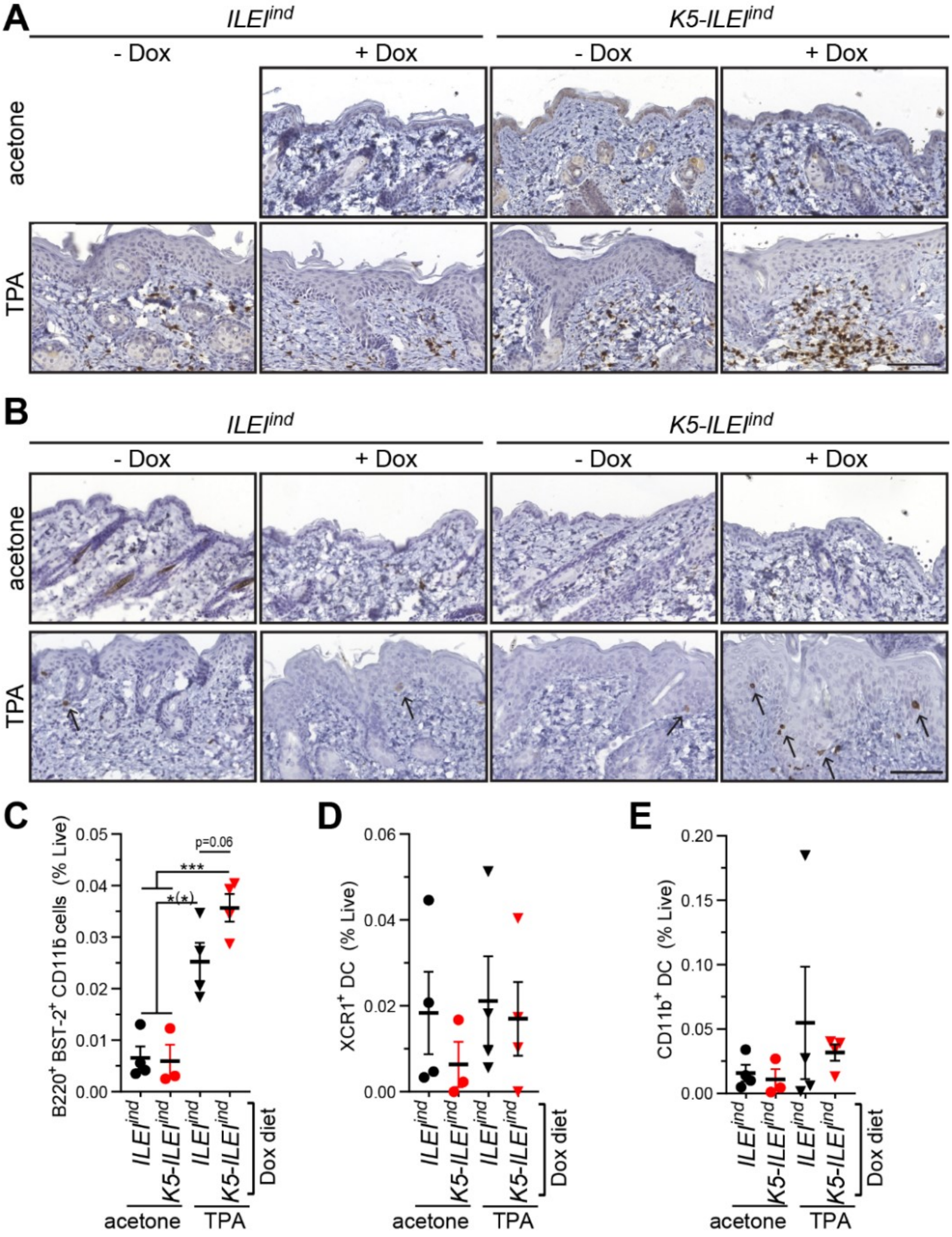


**Appendix Figure S3. Malik et al.**

**Appendix Figure S3. ILEI overexpression in keratinocytes increases the recruitment of neutrophils, epidermal CD8+ T-cells and pDCs.**

(A-B) Representative images of (A) MPO and (B) CD8 immunohistochemistry on thin sections of back skin of *ILEI*$^{ind}$ and *K5-ILEI*$^{ind}$ mice kept on normal or doxycycline diet upon 5 days treatment with acetone or TPA. Arrows mark positive cells in the epidermis.

(C-E) Flow cytometry of back skin after 5 days of TPA or acetone treatment of *ILEI*$^{ind}$ and *K5-ILEI*$^{ind}$ mice kept on doxycycline diet. Analyzed were (C) plasmacytoid dendritic cells (pDC) defined as B220$^{+}$ BST-2$^{+}$ CD11b$^{-}$ dendritic cells (DC), (D) Type I DCs (cDC1) defined as XCR1$^{+}$ DCs and (E) Type II DCs (cDC2) defined as CD11b$^{+}$ (CD11c$^{+}$ MHC-II$^{+}$) DCs. Graphs show immune cells as % of live, single cells (n=3-4; 2 independent experiments). In C, statistical significance was determined by Student's t-test and marked with asterisks (*$p<0.05$; **$p<0.01$; ***$p<0.001$).

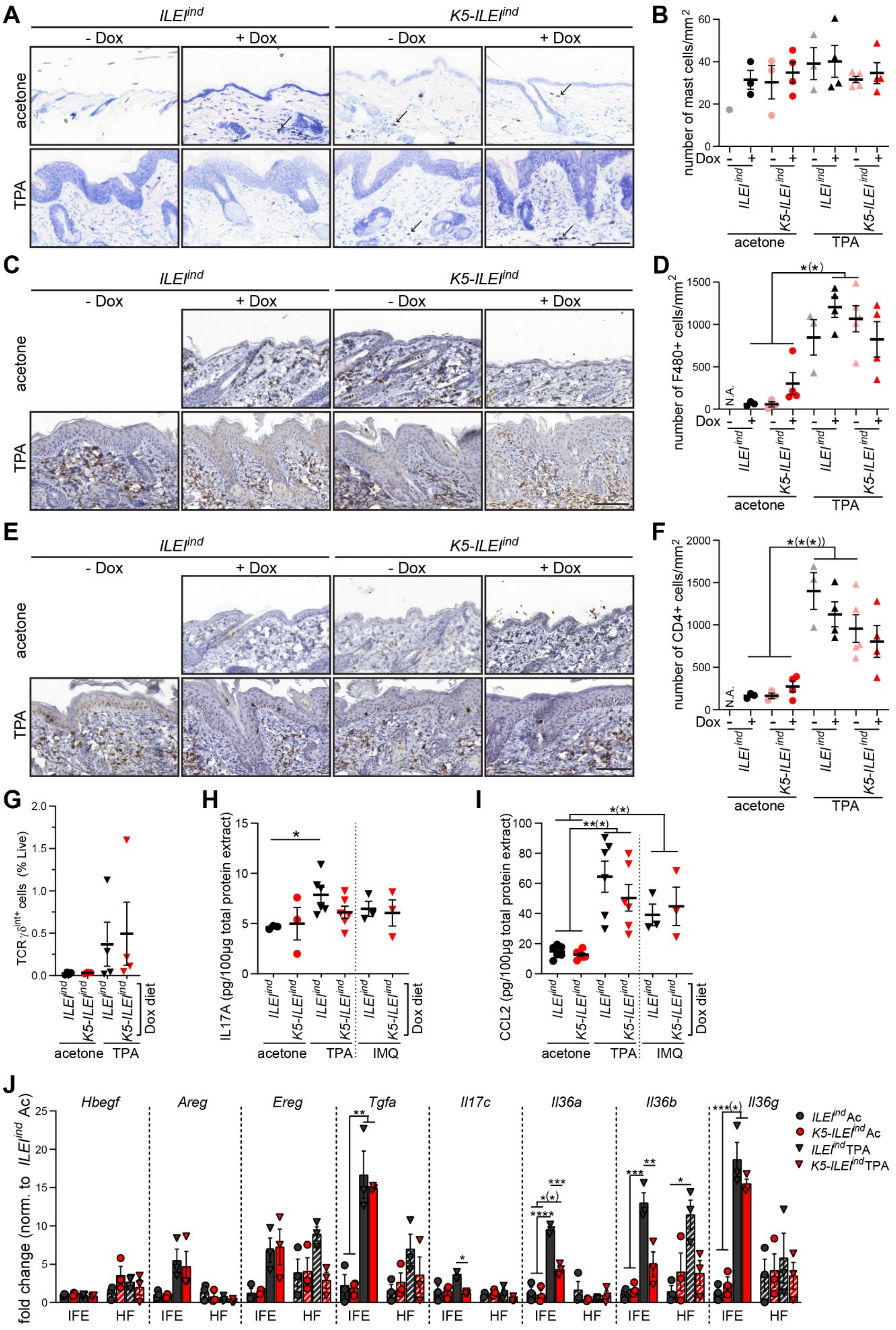


**Appendix Figure S4. Malik et al.**

**Appendix Figure S4. ILEI overexpression in keratinocytes does not influence the recruitment of mast cells, macrophages, CD4+ and gdT-cells and the expression of EGFR ligands and IL17- and IL36-family cytokines after TPA treatment.**

(A-B) Giemsa staining with (A) representative images and (B) mean number of mast cells/$mm^2$ skin area ±SEM on thin sections of back skin of $ILEI^{ind}$ and $K5\text{-}ILEI^{ind}$ mice kept on normal or doxycycline diet upon 5 days treatment with acetone or TPA (n=1-4; 2 independent experiments). Arrows mark purple colored counted cells.

(C-D) F4/80 immunohistochemistry with (C) representative images and (D) mean number of F4/80 positive cells/$mm^2$ skin area ±SEM on thin sections of back skin of $ILEI^{ind}$ and $K5\text{-}ILEI^{ind}$ mice kept on normal or doxycycline diet upon 5 days treatment with acetone or TPA (n=3-4; 2 independent experiments). N.A., not analyzed.

(E-F) CD4 immunohistochemistry with (E) representative images and (F) mean number of CD4 positive cells/$mm^2$ skin area ±SEM on thin sections of back skin of $ILEI^{ind}$ and $K5\text{-}ILEI^{ind}$ mice kept on normal or doxycycline diet upon 5 days treatment with acetone or TPA (n=3-4; 2 independent experiments). N.A., not analyzed.

(G) Flow cytometry of back skin after 5 days of TPA or acetone treatment of $ILEI^{ind}$ and $K5\text{-}ILEI^{ind}$ mice kept on doxycycline diet. Analyzed were dermal γδ T cells (TCR $\gamma\delta^{int\,+}$). Immune cells are shown as % of live, single cells (n=3-4; 2 independent experiments).

(H-I) ELISA for (H) IL17A and (I) CCL2 on whole protein extracts of total back skin after 5 days of TPA, acetone or imiquimod (IMQ) treatment of $ILEI^{ind}$ and $K5\text{-}ILEI^{ind}$ mice kept on doxycycline diet (n=3-6; 3 independent experiments). TPA and IMQ cohorts were separately compared to acetone treatment.

(J) Mean fold change ±SEM in mRNA expression of *Hbegf, Areg, Ereg, Tgfa, Il17c, Il36a, Il36b and IL36g* in freshly sorted keratinocytes enriched for the inter-follicular epithelium (IFE) and hair follicles (HF) from acetone and TPA treated back skin of $ILEI^{ind}$ and $K5\text{-}ILEI^{ind}$ mice kept on doxycycline diet (n=3).

In, statistical significance was determined by one-way ANOVA (B,D,F,I and J) with Tukey multiple comparison test or with Student'S t-test (H) and marked with asterisks (*$p<0.05$; **$p<0.01$; ***$p<0.001$; ***$p<0.0001$).

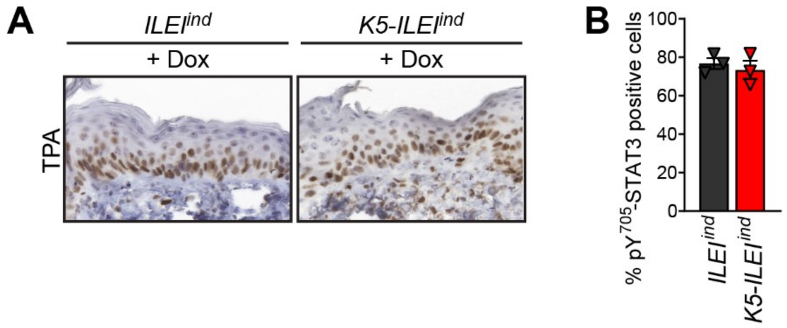


Appendix Figure S5. Malik et al.

**Appendix Figure S5. STAT3-Tyr705 phosphorylation levels are not significantly altered in ILEI overexpressing skin upon inflammation.**

(A-B) pY$^{705}$-STAT3 immunohistochemistry with (A) representative images and (B) mean number of pY$^{705}$-STAT3 positive cells/mm$^2$ skin area ±SEM on thin sections of back skin of *ILEI$^{ind}$* and *K5-ILEI$^{ind}$* mice kept on doxycycline diet upon 5 days treatment with acetone or TPA (n=3).

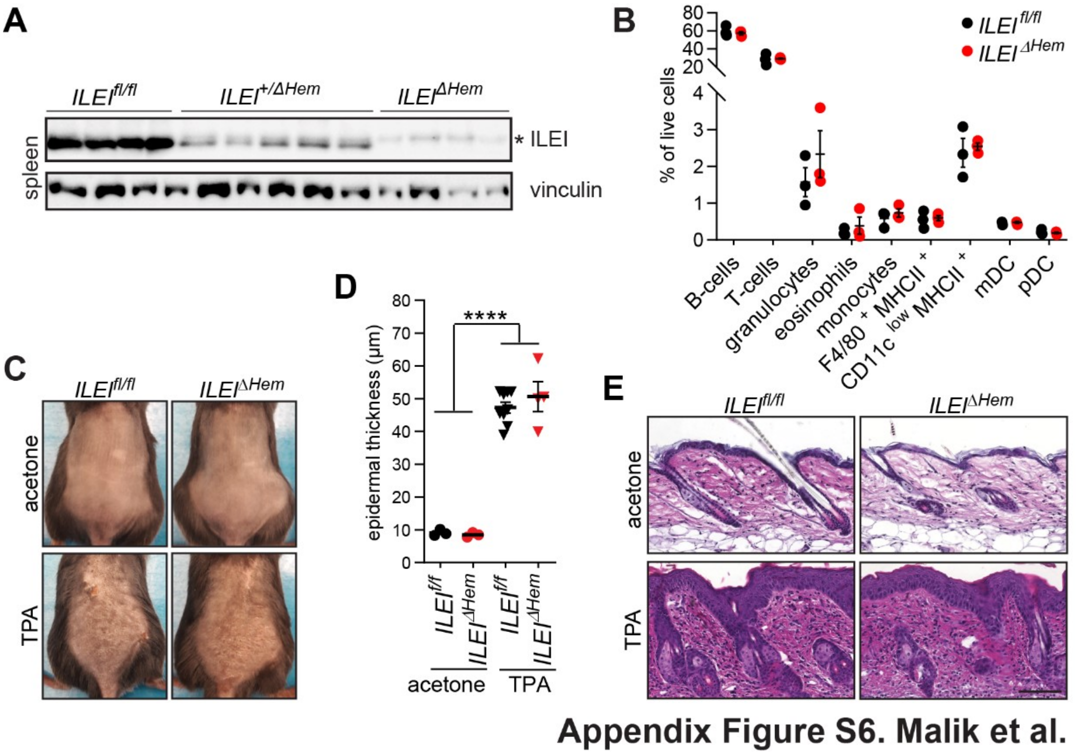


**Appendix Figure S6. ILEI deletion in hematopoietic cells does not influence systemic immune composition, homeostatic skin and TPA-triggered epidermal thickening.**

(A) ILEI Western blot analysis of spleen extracts of *ILEI*$^{fl/fl}$, *ILEI*$^{+/\Delta Hem}$ and *ILEI*$^{\Delta Hem}$ mice (n=4-5). Vinculin was used as loading control, asterisk marks cross-reacting protein with the ILEI antibody.

(B) Relative distribution of marked immune cell populations shown as mean percentage of live cells ±SEM of spleen single cell suspensions of *ILEI*$^{fl/fl}$ and *ILEI*$^{\Delta Hem}$ mice (n=3).

(C) Macroscopic appearance, (D) mean epidermal thickness ±SEM and (E) hematoxylin-eosin staining of the back skin of *ILEI*$^{fl/fl}$ and *ILEI*$^{\Delta Hem}$ mice upon 5 days treatment with acetone or TPA (n=3-9; 2 independent experiments). Scale bar 100 µm. In D, statistical significance was determined by one-way ANOVA with Tukey multiple comparison test and marked with asterisks (****$p<0.0001$).

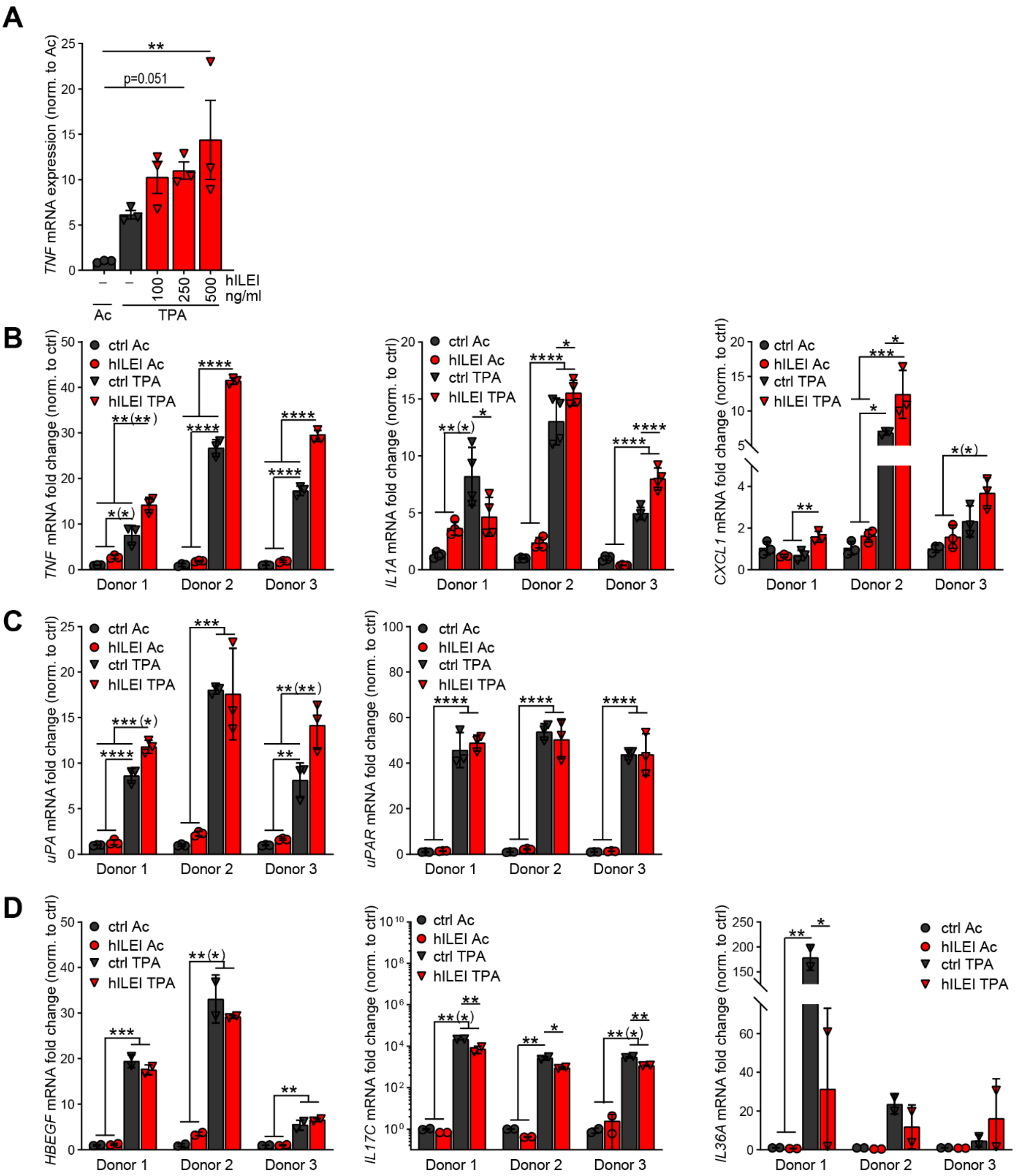


**Appendix Figure S7. Malik et al.**

**Appendix Figure S7. Recombinant ILEI in human keratinocytes recapitulates the changes in cytokine expression found in the *K5-ILEI*$^{ind}$ mouse model.**

(A) Mean fold change ±SEM in mRNA expression of *TNFa* in human primary keratinocytes treated with acetone and TPA and with increasing concentrations (100ng/ml, 250ng/ml, 500ng/ml) of human recombinant ILEI (hILEI) for 8 hours (n=3).

(B-D) Mean fold change ±SD in mRNA expression of (B) *TNFa* (left panel), *IL1A* (mid panel), *CXCL1* (right panel), (C) *uPA* (left panel), *uPAR* (mid panel) and (D) *HBEGF* (left panel), *IL17C* (mid panel) and *IL36A* (right panel) in human primary keratinocytes treated with acetone or TPA and hILEI (500ng/ml) for 8 hours (n=2-4). (A) consists of biological, (B-D) technical replicates, plots representative example of two independent experiments. Statistical significance was determined by one-way ANOVA and marked with asterisks (*$p<0.05$; **$p<0.01$; ***$p<0.001$; ****$p<0.0001$).

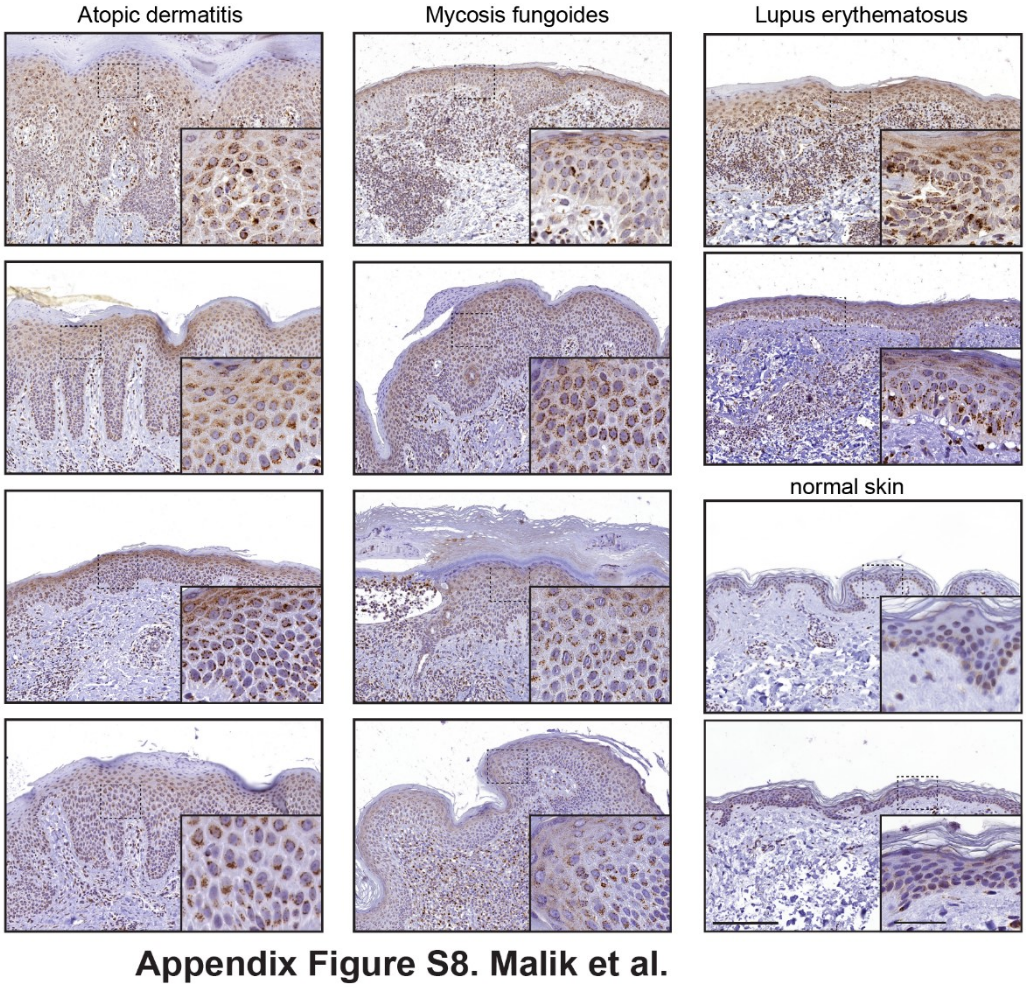


**Appendix Figure S8. ILEI protein levels are elevated in skin lesions of Atopic dermatitis and other skin diseases linked to chronic inflammation.**

Representative images of ILEI immunohistochemistry on Atopic dermatitis (left panels, n=4), Mycosis fungoides (mid panels, n=4), Lupus erythematosus (upper right panels, n=2) and normal (right panels, n=2) skin sections, scale bar 200 µm. Inlets show a magnification of the marked regions, scale bar 50 µm.

**Appendix Table S1. List of genotyping primers**

| Primer name | Sequence 5'-3' |
|---|---|
| K5rtTA_fwd | CGCTGTGGGGCATTTTACTTTAG |
| K5rtTA_rev | CATGTCCAGATCGAAATCGTC |
| ILEIind_fwd | CCCTCCATGTGTGACCAAGG |
| ILEIind_Rwt | GCACAGCATTGCGGACATGC |
| ILEIind_Rmut | GCAGAAGCGCGGCCGTCTGG |
| ILEIloxP3_F3 | CCTTACATCCTAGGCCAGATGCTTGG |
| ILEIloxP3_R | CATGAGGCCAATCCTTTCATAGTTCAC |
| K5cre1 | CATACCTGGAAAATGCTTCTGTCC |
| K5cre2 | CATCGCTCGACCAGTTTAGTTACC |
| Jun1 | CCGCTAGCACTCACGTTGGTAGGC |
| Jun2 | CTCATACCAGTTCGCACAGGCGGC |
| Vav iCre for | CCG AGG GGC CAA GTG AGA GG |
| Vav iCre rev | GGA GGG CAG GCA GGT TTT GGT G |

**Appendix Table S2. List of primary antibodies**

| Antibody | Company | Catalogue# | Species | Application |
|---|---|---|---|---|
| Fam3C | Proteintech | 60282-1-Ig | mouse | IHC: 1:700<br>W: 1:1000 |
| Ki67 | Abcam | ab15580 | Rabbit | IHC: 1:1500 |
| MPO | Abcam | ab208670 | Rabbit | IHC: 1:1000 |
| CD4 | Abcam | ab183685 | Rabbit | IHC: 1:1000 |
| CD8 | Abcam | ab217344 | Rabbit | IHC: 1:1000 |
| F4/80 | Biorad | MCA497GA | Rat | IHC: 1:100 |
| K5 | Progen | GP-CK5-WBC | Guinea Pig | IHC: 1:200<br>ICC: 1:200 |
| K10 | Biolegend | 905401 | Rabbit | IHC: 1:200<br>ICC:1:200 |
| AKTpospho (Ser437) | CST | 4060 | Rabbit | IHC: 1:100<br>W: 1:1000 |
| p44/42MAPKphospho (T202/Y204) | CST | 4370 | Rabbit | IHC: 1:200<br>W: 1:1000 |
| pSTAT3(Ser727) | CST | 9134 | Rabbit | IHC: 1:100<br>W: 1:1000 |
| pSTAT3(Tyr705) | CST | 9145 | rabbit | IHC: 1:100<br>W: 1:1000 |
| AKT | CST | 9272 | rabbit | W: 1:1000 |
| p44/42MAPK | CST | 4696 | mouse | W: 1:1000 |
| STAT3 | CST | 9139 | mouse | W: 1:1000 |
| vinculin | Sigma | V9131 | mouse | W 1:1000 |
| Antibodies used for flow cytometry | | | | |
| BST-2 | BioLegend | 127023 | Rat | FC: 1:200 |
| B220 | BioLegend | 103258 | Rat | FC: 1:200 |
| CD11b | BioLegend | 101216 | Rat | FC: 1:500 |
| CD11c | BioLegend | 117338 | Armenian Hamster | FC: 1:500 |
| CD45 | BioLegend | 103149 | Rat | FC: 1:200 |
| CD64 | BioLegend | 139316 | Mouse | FC: 1:200 |
| MHC-II | BioLegend | 107622 | Rat | FC: 1:500 |
| TCR $\gamma/\delta$ | BioLegend | 118116 | Armenian Hamster | FC: 1:200 |
| XCR1 | BioLegend | 148220 | Mouse | FC 1:200 |

**Appendix Table S3. List of primer sequences for qPCR**

| Target | Mouse (sequence 5'-3') | Human (sequence 5'-3') |
|---|---|---|
| GAPDH_fwd | ACCCAGAAGACTGTGGATGG | - |
| GAPDH_rev | CACATTGGGGGTAGGAACAC | - |
| TBP_fwd | - | TGTATCCACAGTGAATCTTGGTTG |
| TBP_rev | - | GGTTCGTGGCTCTCTTATCCTC |
| K10_fwd | CCCCGGGACTACAGCAAATA | - |
| K10_rev | CAGGGTCACCTCATTCTCGT | - |
| K5_fwd | TACAGGAAGCTGCTGGAGGG | - |
| K5_rev | TCCGTAGCCAGAAGAGACAC | - |
| K16_fwd | CCACTCCTCCTCACAGCACTC | - |
| K16_rev | CCTGGAACTCTGACTTTGGCTCT | - |
| TNFa_fwd | GAACTGGCAGAAGAGGCACT | CTCTTCTGCCTGCTGCACTTT |
| TNFa_rev | AGGGTCTGGGCCATAGAACT | ATGGGCTACAGGCTTGTCACT |
| IL1a_fwd | CACCTTACACCTACCAGAGTGATTTG | TGTATGTGACTGCCCAAGATG AAG |
| IL1a_rev | TGTTGCAGGTCATTTAACCAAGTG | AGAGGAGGTTGGTCTCACTACC |
| CXCL1_fwd | GCCAATGAGCTGCGCTGT | AGCTTGCCTCAATCCTGCATCC |
| CXCL1_rev | CCTTCAAGCTCTGGATGTTCTTG | TCCTTCAGGAACAGCCACCAG |
| uPA_fwd | TGTGAGATCACTGGCTTTGG | GTCACCACCAAAATGCTGTG |
| uPA_rev | CCATAGTAGTGGGGCTGCAT | CCAGCTCACAATTCCAGTCA |
| uPAR_fwd | GCCTGGTAGAGGAGTGTGCT | AGCTATCGGACTGGCTTGAA |
| uPAR_rev | CCTGTTGGTCTTTTCGCTGT | ATGTCTGATGAGCCACAG |
| IL17C_fwd | AGGTGCTGGAAGCTGACACTC | GAGGTGTTGGAGGCAGACA |
| IL17C_rev | TCCACGACACAAGCATCCTGC | CATCGATACAGCCTCTGCAC |
| IL36a_fwd | GCTGTGTTGAGATGGAGGGCA | CTTCAGGACCAGACGCTCATAG |
| IL36a_rev | GACAGAAGTGGAGCCCTCTAT | GGCAGAGATTGAGTCCATTCAGG |
| Hbegf_fwd | GAGTCCCGTACTCCTTCTTGC | TGTATCCACGGACCAGCTGCTA |
| Hbegf_rev | CAGCCAAGACTGTAGTGTGGT | TGCTCCTCCTTGTTTGGTGTGG |
