# Supplementary material for: FAM3C/ILEI protein is elevated in psoriatic lesions and triggers psoriasiform hyperproliferation in mice
Source: EMBO Mol Med. 2023 May 25;15(7):e16758. doi: 10.15252/emmm.202216758 (PMC10331587; doi:10.15252/emmm.202216758)
Supplement: Supplementary file 2 — Expanded View Figures PDF [file EMMM-15-e16758-s010.pdf]

Expanded View Figures

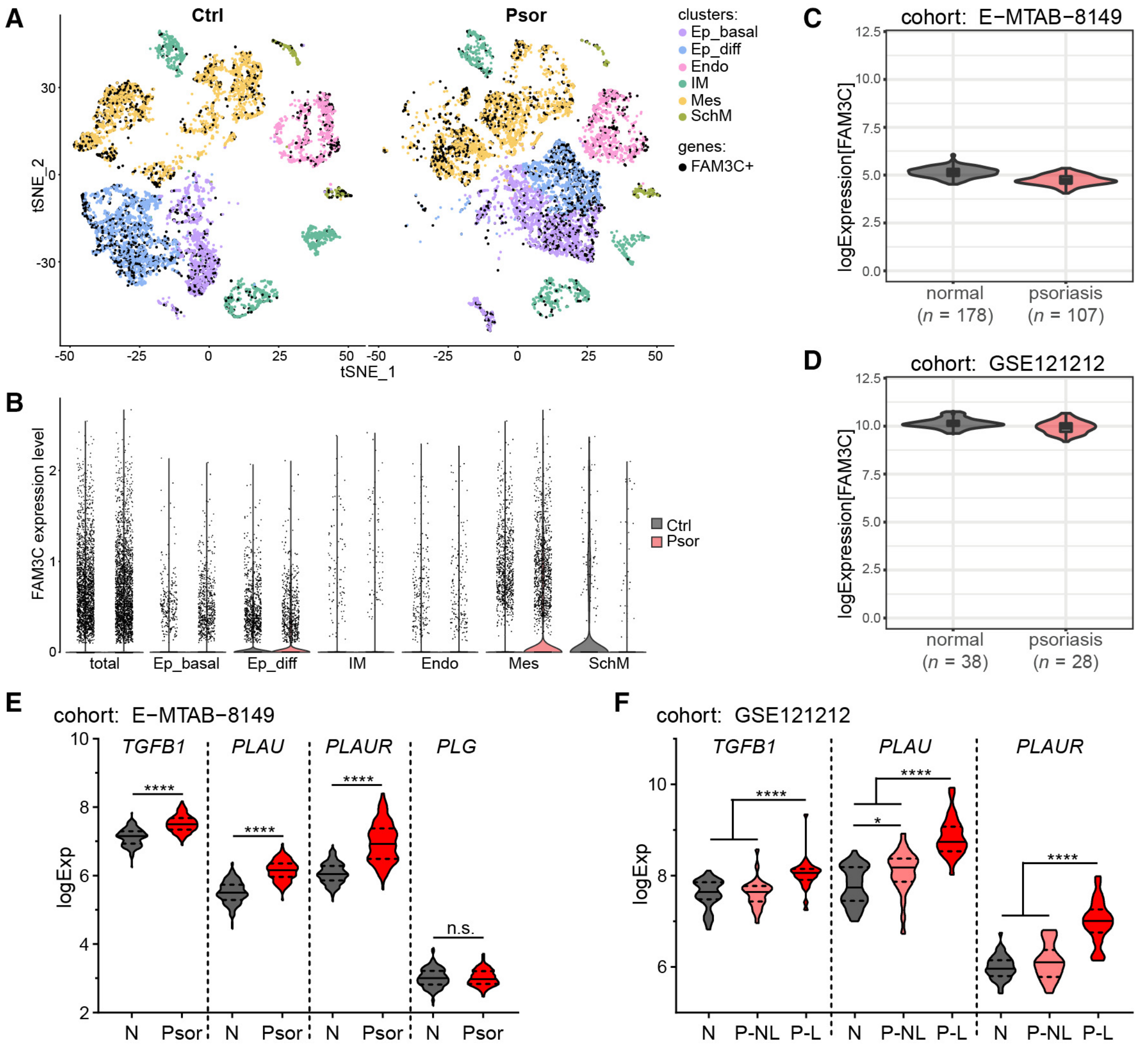

Figure EV1. Transcripts of positive regulators of FAM3C/ILEI translation and secretion, but not FAM3C/ILEI mRNA are upregulated in psoriatic patients.

A, B scRNA-Seq human full-thickness skin dataset GSEE162183 of control (Ctrl) and Psoriasis (Psor) patients presented (A) in t-SNE overlay visualization overlayed with cells expressing FAM3C and (B) in violin plots showing expression levels of FAM3C in respective cell clusters. Ep_basal, epidermis basal subcluster; Ep_diff, epidermis differentiated subcluster; IM, immune cluster; Endo, endothelial cluster; Mes, mesenchymal cluster; SchM, Schwann/Melanocyte-like cluster.

C, D log2fold *FAM3C* mRNA expression levels in normal skin and psoriatic lesions of the datasets (C) MTAB-8149 (n = 285) and (D) GSE121212 (n = 66). Violin plot overlayed with Box-and-whiskers plot: Central band shows median, box extends from the 25th to 75th percentiles and whiskers go from the smallest (min) to the largest (max) value. Density curves of the violin plot correspond to the approximate frequency of data points in each region.

E, F log2fold mRNA expression levels of (E) *TGFB1*, *PLAU*, *PLAUR* and *PLG* in normal skin (N) (n = 178) and psoriatic lesions (Psor; n = 107) of the RNA sequencing dataset MTAB-8149 and (F) *TGFB1*, *PLAU* and *PLAUR* in normal skin (N) (n = 38) and in nonlesional (P-NL) (n = 27) and lesional (P–L) skin (n = 28) of psoriasis patients of the microarray dataset GSE121212. Violin plots: central band shows median, dashed lines label the 25th and 75th percentiles, and density curves correspond to the approximate frequency of data points in each region extending from the smallest (min) to the largest (max) value.

Data information: statistical significance was determined by (E) student's t-test and (F) one-way ANOVA with Tukey multiple comparison test and marked with asterisks (*$P < 0.05$; ****$P < 0.0001$).

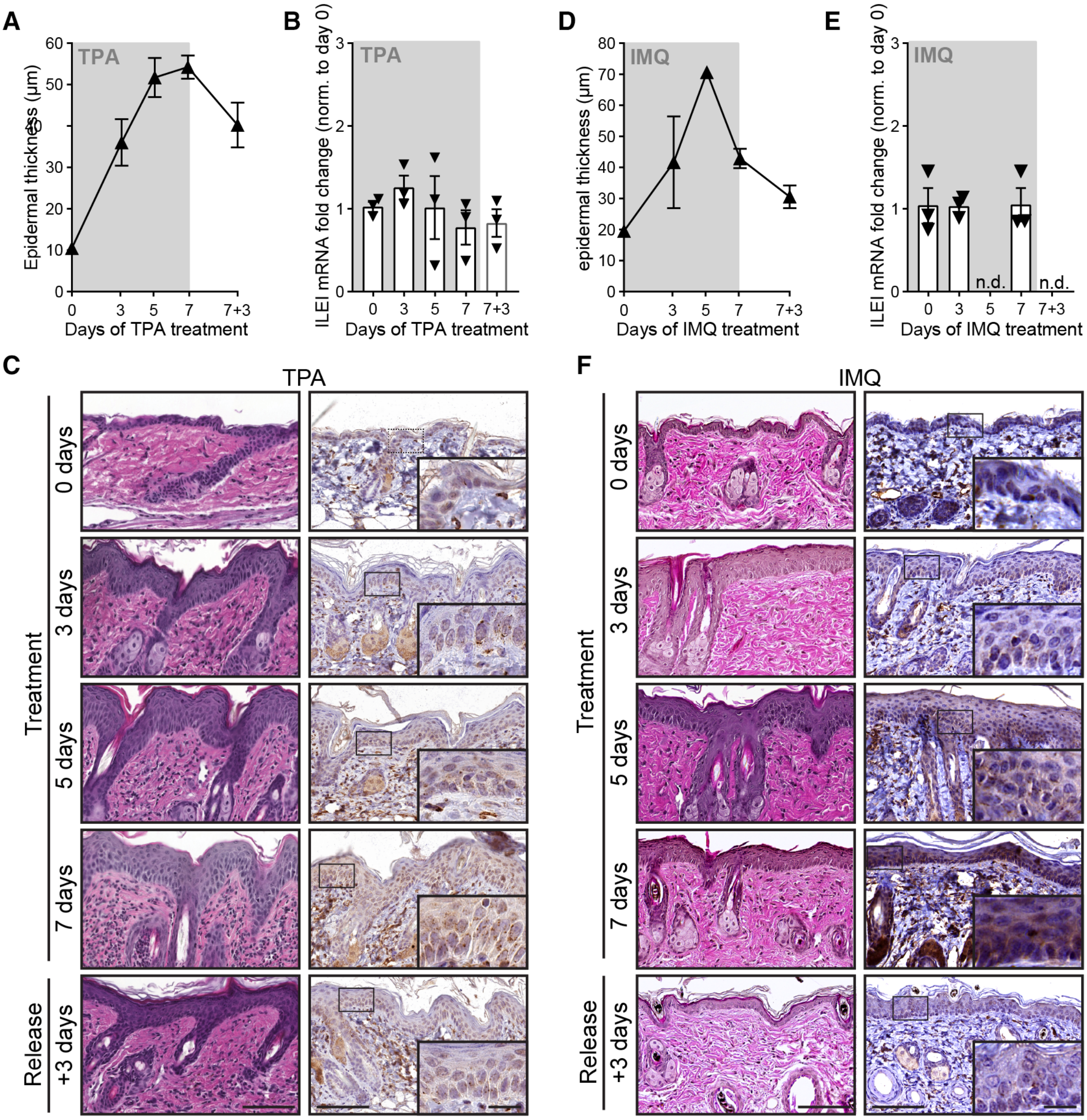

Figure EV2. Chemically triggered inflammatory conditions increase ILEI protein, but not mRNA levels in mouse skin.

A–F Analysis on back skin of wild-type mice treated for 0, 3, 5, and 7 days with (A–C) TPA or (D–F) IMQ followed by an additional withdrawal for 3 days (n = 3 for each time point and treatment; 2 independent experiments). Timely changes of (A,D) mean epidermal thickness ± SEM and (B, E) mean ILEI mRNA expression fold change ±SEM. Epidermal thickness was quantified from (C, F left panels) hematoxylin–eosin stained thin sections of back skin. mRNA expression was normalized to untreated skin (Day 0). (C, F right panels) Representative images of ILEI immunohistochemistry on thin sections of back skin. Scale bar 100 µm. Inlets show a magnification of the marked regions, scale bar 20 µm. IMQ, imiquimod; nd, not done.

Figure EV3. Differentially expressed genes and enriched pathways in murine TPA-treated skin and in human psoriasis.

A–I Transcriptomic analysis showing (A, D, G) Differentially expressed genes (DEGs), (B, E, H) significantly enriched pathways of the Hallmark database ranked according to their normalized enrichment score and (C, F, I) significantly enriched terms of the GO:term database plotted in clusters after dimensionality reduction with indicated lead terms computed from the gene expression profiling of (A–C) the back skin of *ILEI*^{ind} mice treated with acetone or TPA for 5 days (n = 3) and the human psoriasis datasets (D–F) E-MTAB-8149 and (G–I) GSE121212. For the volcano plot on (A, D, G), cutoff was set for an adjusted P-value of <0.05. Genes of the *K5-ILEI*^{ind} TPA signature (60 genes) are in blue (downregulated), red (upregulated) or black (nonsignificant), top 10 significant genes of the signature marked by names, if directionality maintained, in bold.

J Venn diagram on the distribution of enriched GO terms in the transcriptome of the back skin of *K5-ILEI*^{ind} vs *ILEI*^{ind} mice treated with TPA and the two human psoriasis datasets E-MTAB-8149 and GSE121212.

▶

 © 2023 The Authors

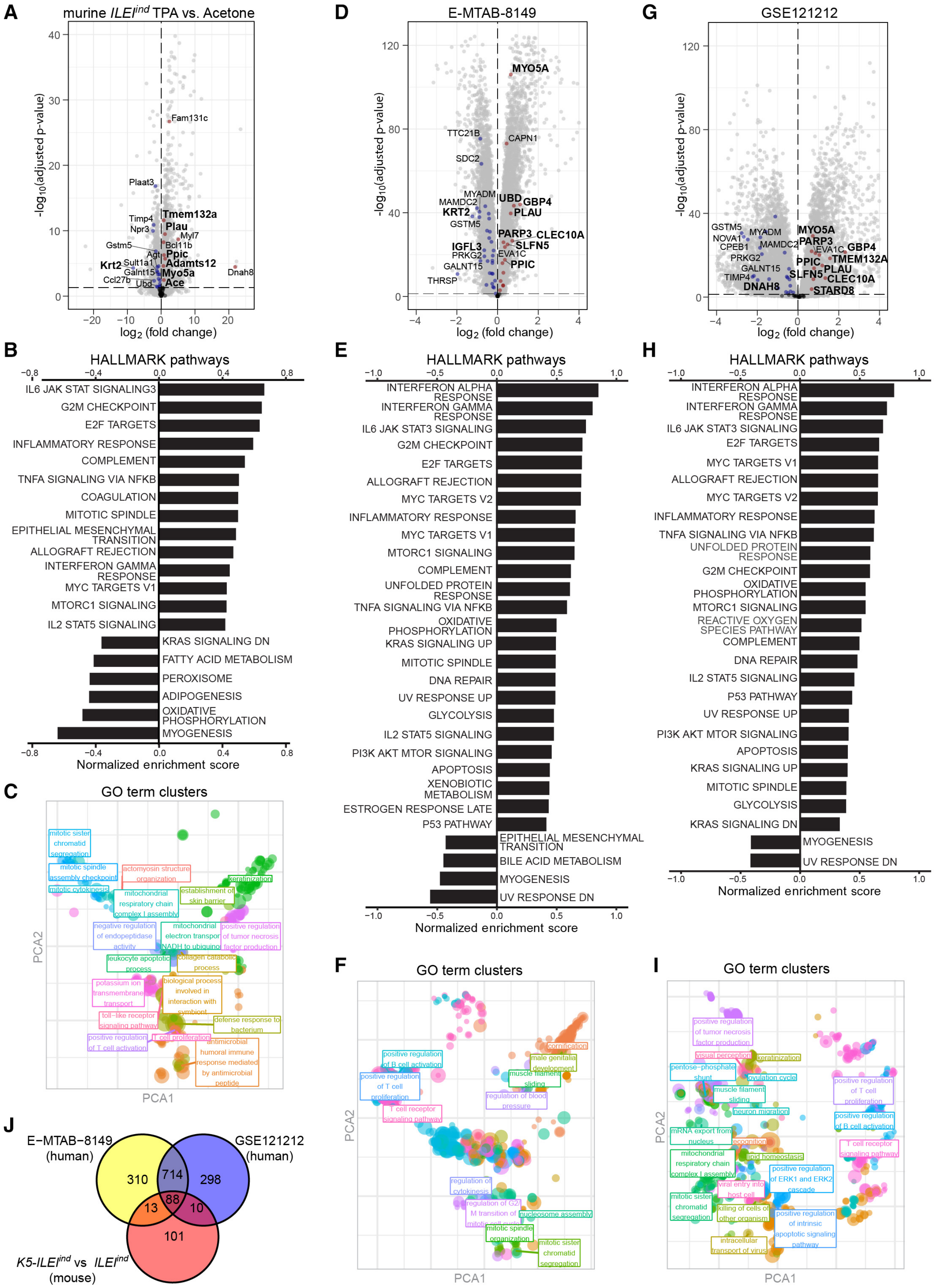
A
murine ILEI^ind TPA vs. Acetone
D
E-MTAB-8149
G
GSE121212
B
HALLMARK pathways
E
HALLMARK pathways
H
HALLMARK pathways
Normalized enrichment score
C
GO term clusters
F
GO term clusters
I
GO term clusters
PCA1
PCA2
J
E-MTAB-8149 (human)
GSE121212 (human)
K5-ILEI^ind vs ILEI^ind (mouse)
310
714
298
13
88
10
101

Figure EV3.

Figure EV4. Transcriptomic profiling of total skin and freshly isolated keratinocytes of the *K5*-ILEI^{ind} mouse model identify psoriasis-linked regulatory genes with ILEI- and TPA-dependent relevance.

A–C Heatmap on genes expressed in TPA or acetone-treated *K5-ILEI^{ind}* and control back skin showing (A) regulatory genes on ILEI translation, secretion, and proteolytic cleavage, (B) EGFR ligands and (C) subset of psoriasis-relevant cytokines and cognate receptors (n = 3).

D Volcano plot showing differentially expressed genes (DEGs) computed from the gene expression profiling of freshly sorted keratinocytes enriched for IFE isolated 24 h after a single TPA treatment of K5-*ILEI^{ind}* and *ILEI^{ind}* mice kept on doxycycline diet (n = 3). Cutoff was set for an adjusted P-value of <0.05 and a log2FC of ≥ 1. Top 10 up and downregulated genes are marked by names.

E–G Heatmap on genes expressed in freshly sorted keratinocytes enriched for IFE isolated 24 h after a single TPA treatment of *K5-ILEI^{ind}* and *ILEI^{ind}* mice (n = 3) showing (E) regulatory genes on ILEI translation and secretion, (F) EGFR ligands and (G) inflammatory genes. Tnf, Il1a, and Cxcl3 are marked in red. Genes with an adjusted P-value lower than 0.05 are marked with asterisks (*$P < 0.05$; **$P < 0.01$; ***$P < 0.001$; ****$P < 0.0001$).

 © 2023 The Authors

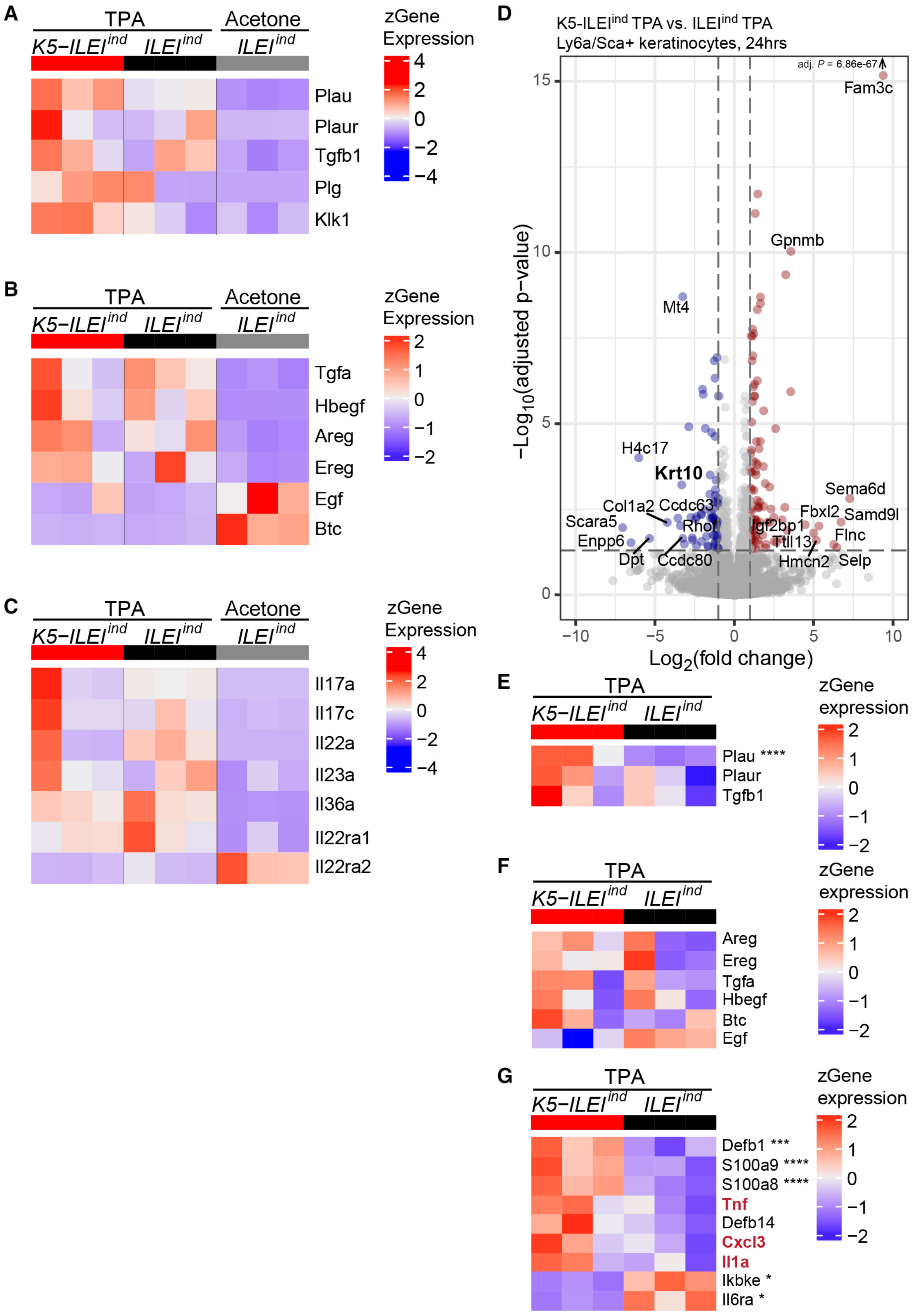
A
TPA
Acetone
K5−ILEI^ind ILEI^ind ILEI^ind
zGene Expression
Plau
Plaur
Tgfb1
Plg
Klk1
B
Tgfa
Hbegf
Areg
Ereg
Egf
Btc
C
Il17a
Il17c
Il22a
Il23a
Il36a
Il22ra1
Il22ra2
D
K5-ILEI^ind TPA vs. ILEI^ind TPA
Ly6a/Sca+ keratinocytes, 24hrs
adj. P = 6.86e-67
Fam3c
Gpnmb
Mt4
H4c17
Krt10
Sema6d
Col1a2 Ccdc63
Fbxl2
Samd9l
Scara5
Rhof
Igf2bp1
Flnc
Enpp6
Ttll13
Dpt
Ccdc80
Hmcn2
Selp
−Log10(adjusted p−value)
Log2(fold change)
E
zGene expression
Plau ****
Plaur
Tgfb1
F
Areg
Ereg
Tgfa
Hbegf
Btc
Egf
G
Defb1 ***
S100a9 ****
S100a8 ****
Tnf
Defb14
Cxcl3
Il1a
Ikbke *
Il6ra *

Figure EV4.

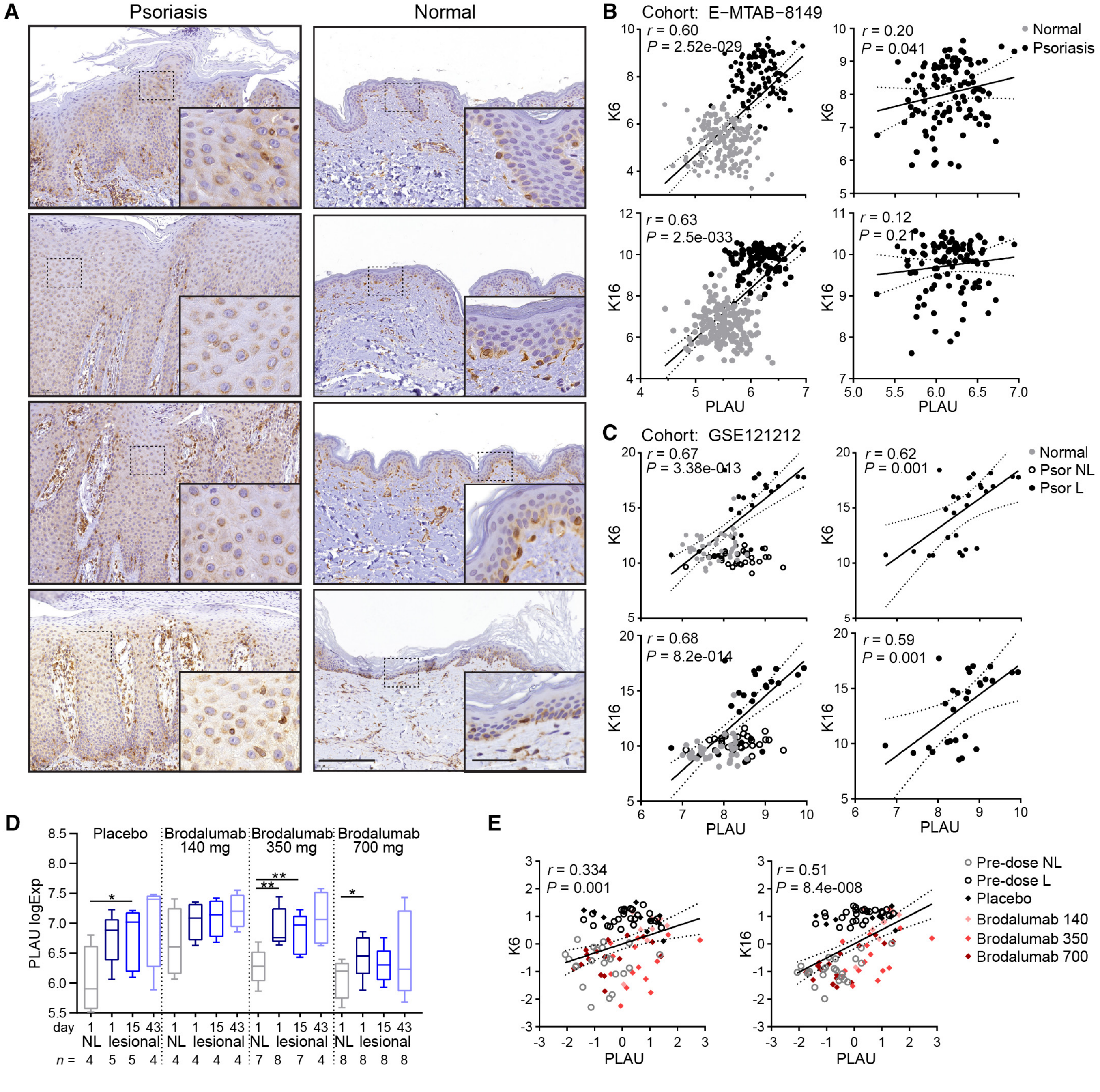

Figure EV5. PLAU shows increased protein expression in the epidermis of psoriatic lesions and (therapy-induced) changes in disease severity are accompanied by changes in uPA mRNA levels.

A Representative images of PLAU immunohistochemistry on psoriatic (left panels) and normal (right panels) skin sections, scale bar 200 μm. Inlets show a magnification of the marked regions, scale bar 50 μm.

B, C Pearson correlation plots of K6 (upper panels) and K16 (lower panels) and PLAU gene expression in a combined set of normal and psoriatic skin (left panels) and in psoriatic lesions (right panels) of the datasets (B) MTAB-8149 and (C) GSE121212.

D log2fold *PLAU* mRNA expression levels of the GSE53552 dataset consisting of lesional and matched nonlesional (NL) psoriatic skin (n = 25 patients) obtained at treatment start (day 1) and from the lesions as serial biopsies at 15 and 43 days after placebo (n = 5 patients) or Brodalumab therapy with doses of 140 mg (n = 4 patients), 350 mg (n = 8 patients) and 700 mg (n = 8 patients). The number of included patient samples at each condition and time points is marked under the diagram. Box-and-whiskers plot: Central band shows median, box extends from the 25th to 75th percentiles, and whiskers go from the smallest (min) to the largest (max) value.

E Pearson correlation plots of K6 (left panel) and K16 (right panel) and PLAU gene expression of the GSE53552 dataset stratified for nonlesional (NL) and lesional (L) psoriatic skin before therapy (predose) and with placebo or 140, 350, and 700 mg doses of Brodalumab therapy.

 © 2023 The Authors
