# Supplementary figures and images for: FAM3C/ILEI protein is elevated in psoriatic lesions and triggers psoriasiform hyperproliferation in mice
Source: EMBO Mol Med. 2023 May 25;15(7):e16758. doi: 10.15252/emmm.202216758 (PMC10331587; doi:10.15252/emmm.202216758)

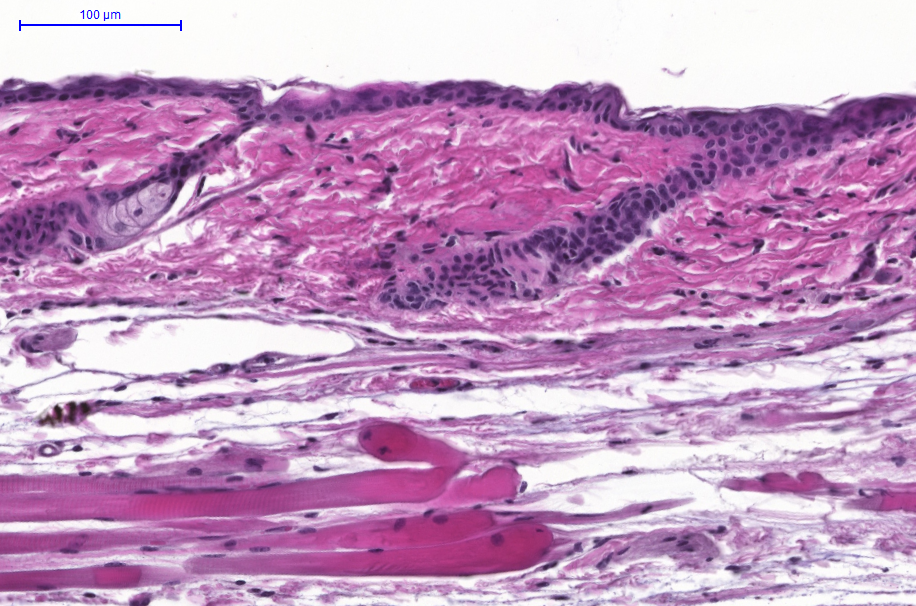

Supplement: Supplementary file 3 — Source Data for Appendix [file EMMM-15-e16758-s005.zip › Figure S1/S1F/0d HE_BL6_014_20x_2.bmp]

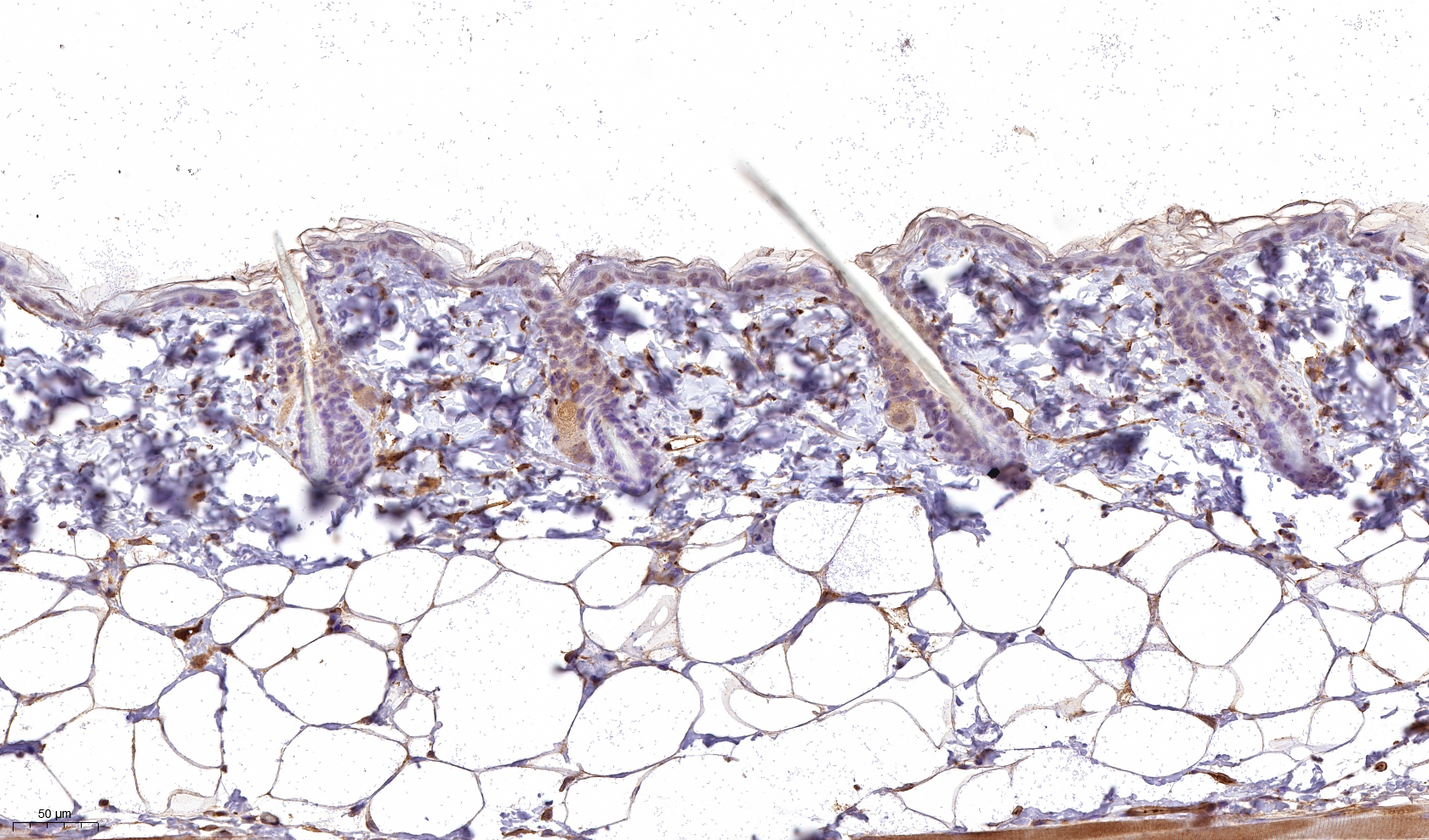

Supplement: Supplementary file 3 — Source Data for Appendix [file EMMM-15-e16758-s005.zip › Figure S1/S1F/0d_TPA ILEI_20.0x.tif]

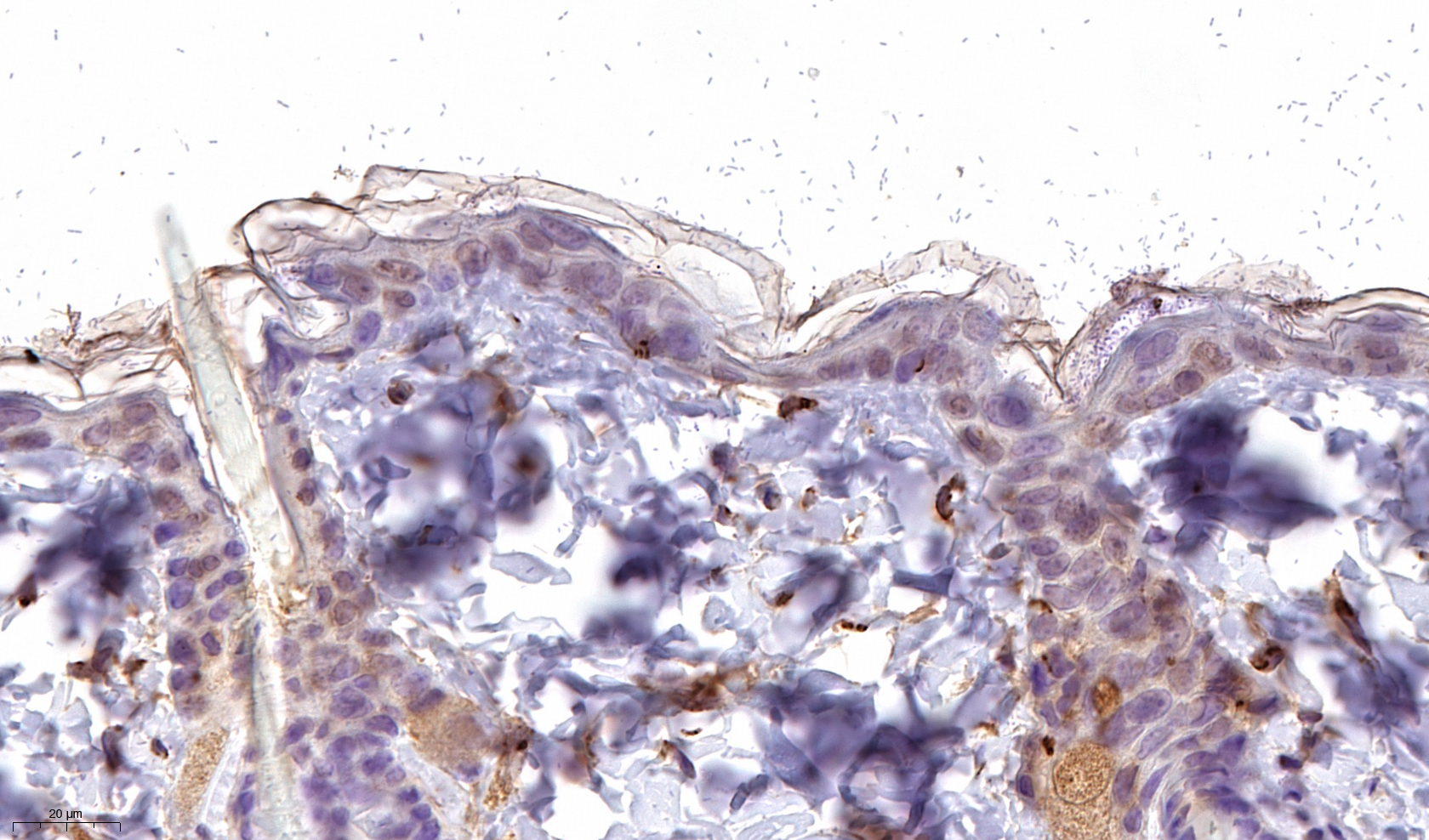

Supplement: Supplementary file 3 — Source Data for Appendix [file EMMM-15-e16758-s005.zip › Figure S1/S1F/0d_TPA ILEI_63.0x.tif]

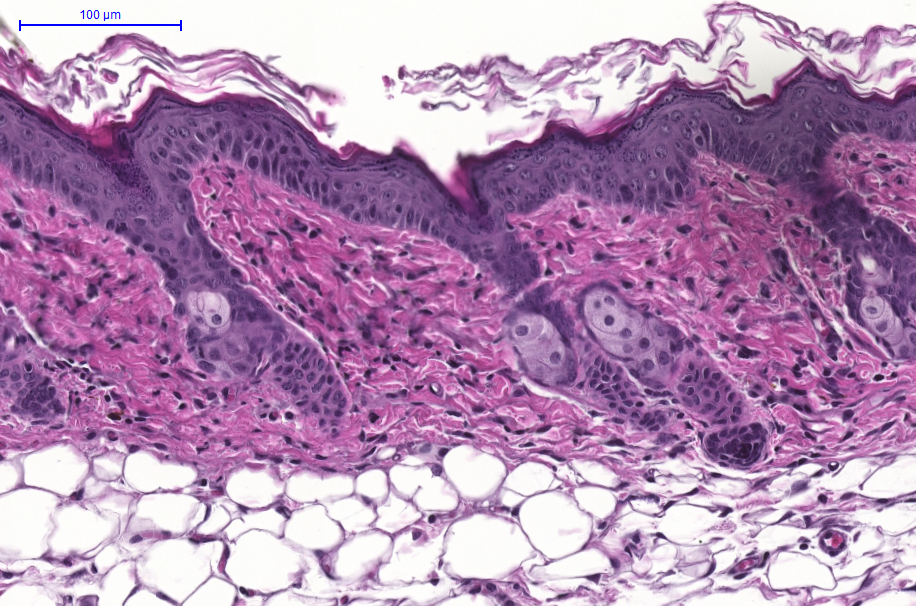

Supplement: Supplementary file 3 — Source Data for Appendix [file EMMM-15-e16758-s005.zip › Figure S1/S1F/3d HE_BL6_013_20x_1.bmp]

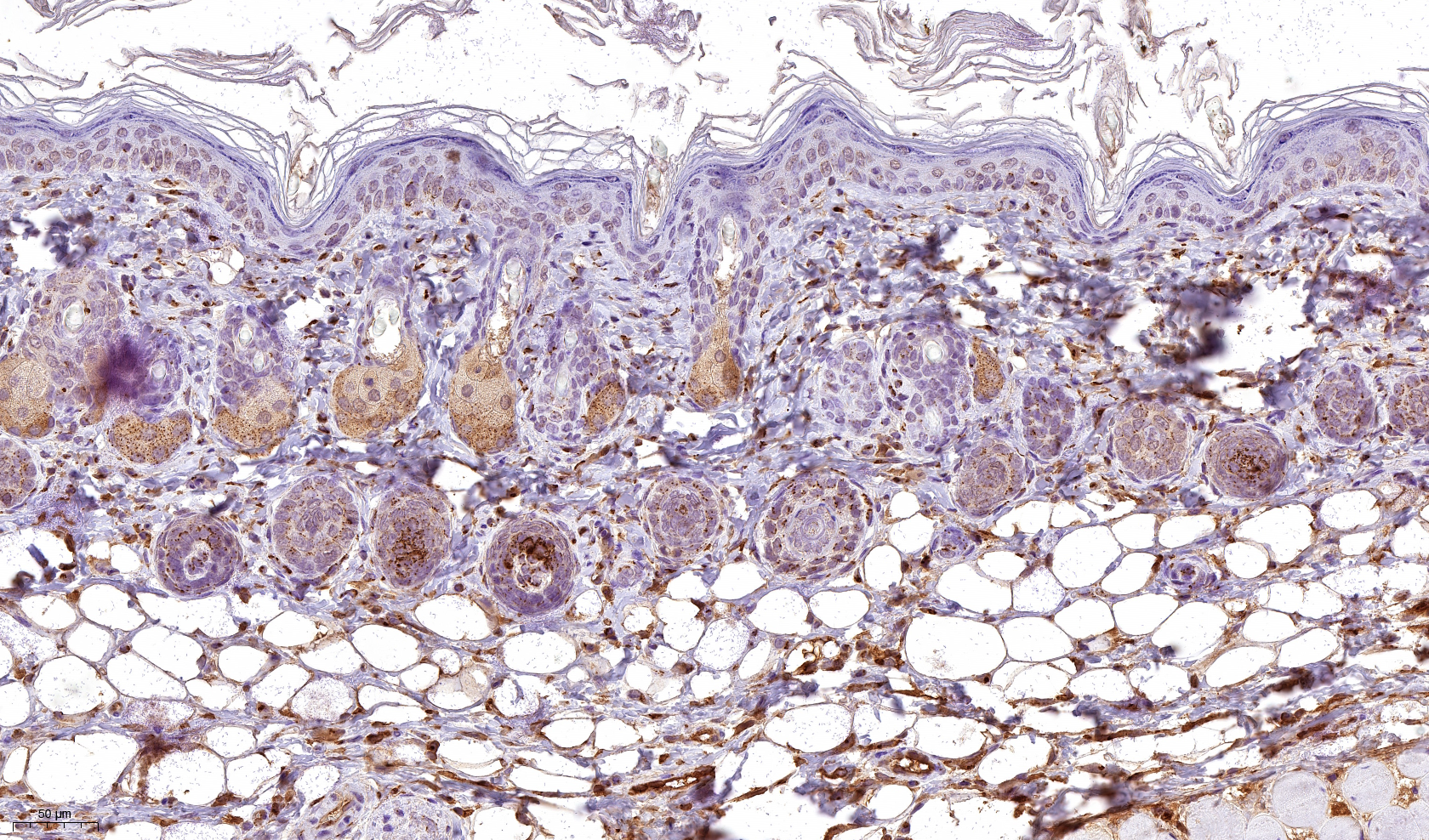

Supplement: Supplementary file 3 — Source Data for Appendix [file EMMM-15-e16758-s005.zip › Figure S1/S1F/3d_TPA ILEI_20.0x.tif]

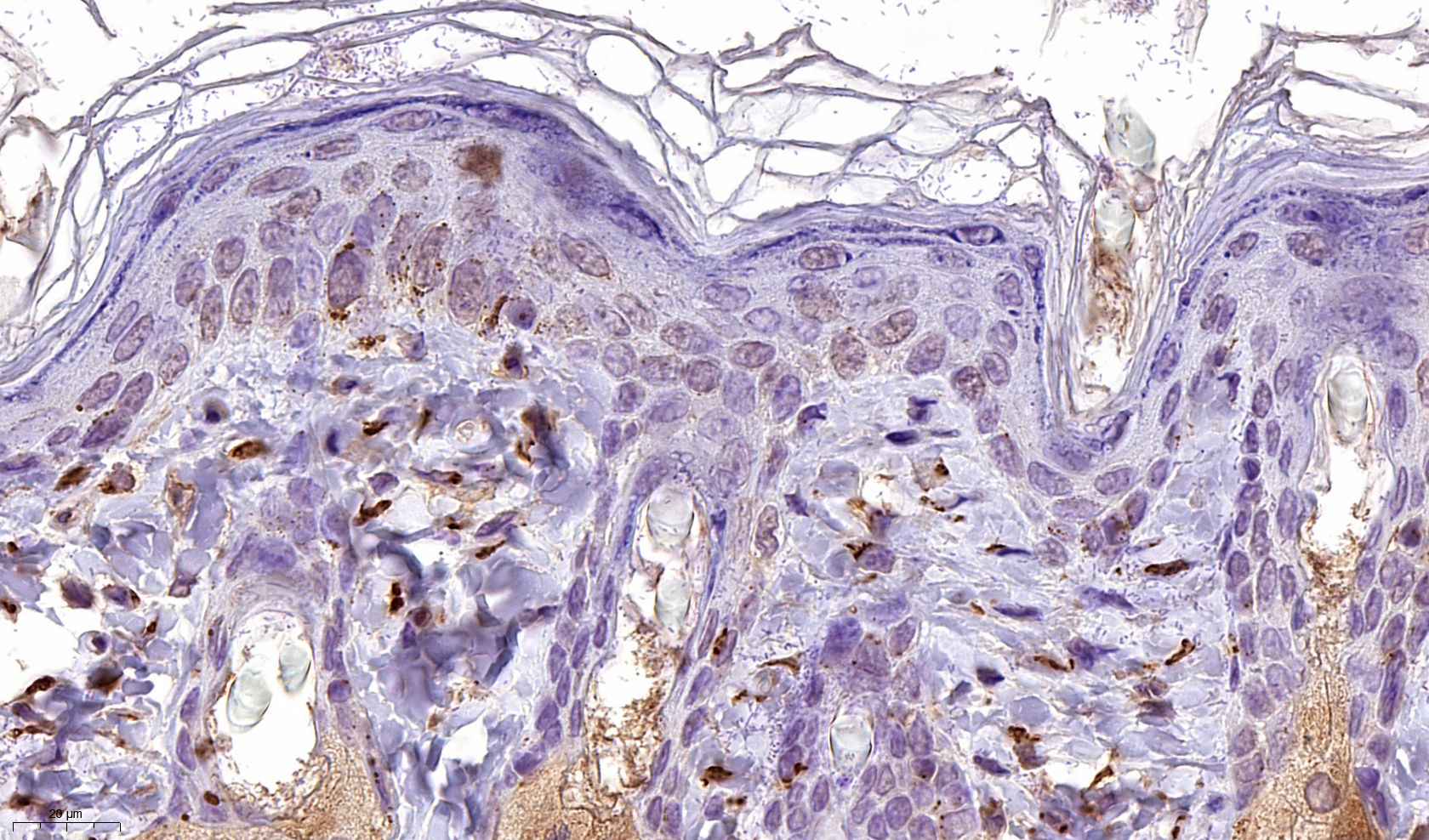

Supplement: Supplementary file 3 — Source Data for Appendix [file EMMM-15-e16758-s005.zip › Figure S1/S1F/3d_TPA ILEI_63.0x.tif]

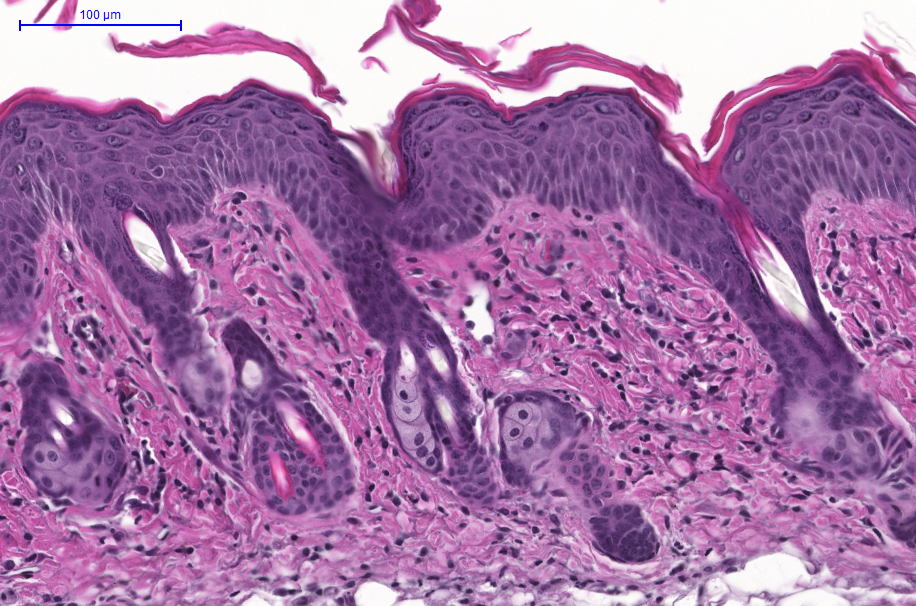

Supplement: Supplementary file 3 — Source Data for Appendix [file EMMM-15-e16758-s005.zip › Figure S1/S1F/5d HE_BL6_011_20x_1.bmp]

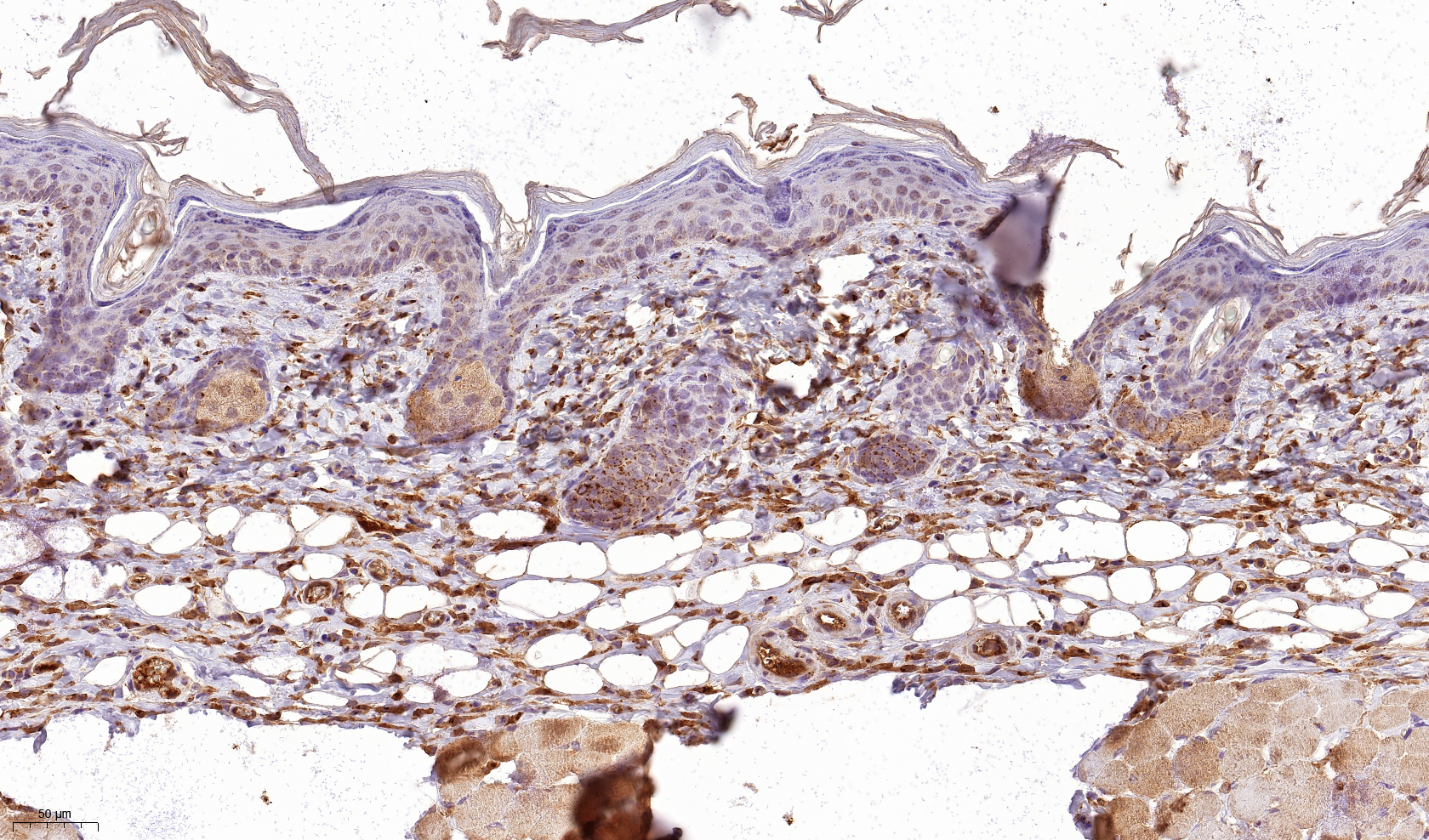

Supplement: Supplementary file 3 — Source Data for Appendix [file EMMM-15-e16758-s005.zip › Figure S1/S1F/5d_TPA ILEI_20.0x.tif]

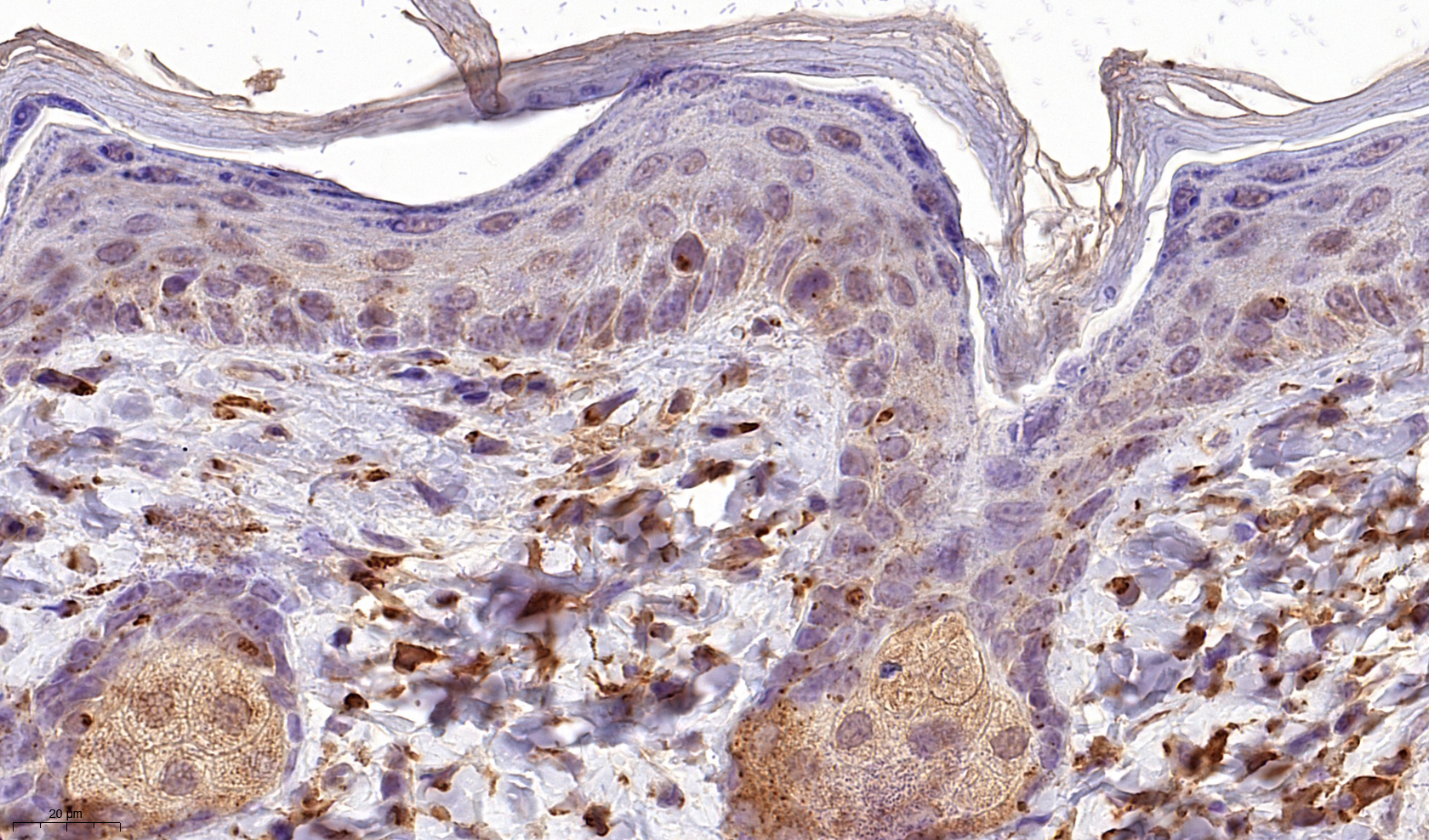

Supplement: Supplementary file 3 — Source Data for Appendix [file EMMM-15-e16758-s005.zip › Figure S1/S1F/5d_TPA ILEI_63.0x.tif]

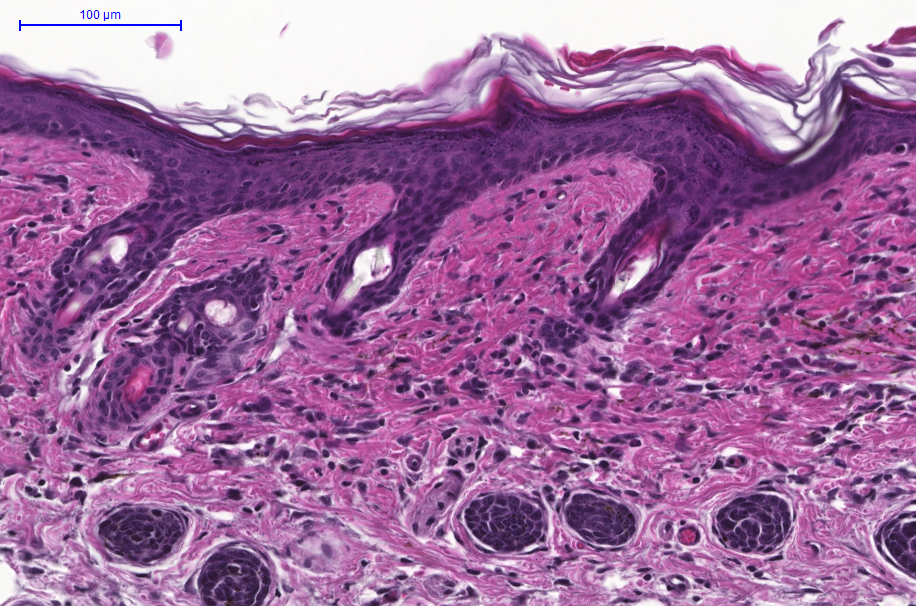

Supplement: Supplementary file 3 — Source Data for Appendix [file EMMM-15-e16758-s005.zip › Figure S1/S1F/7+3 HE_BL6_006_20x_4.bmp]

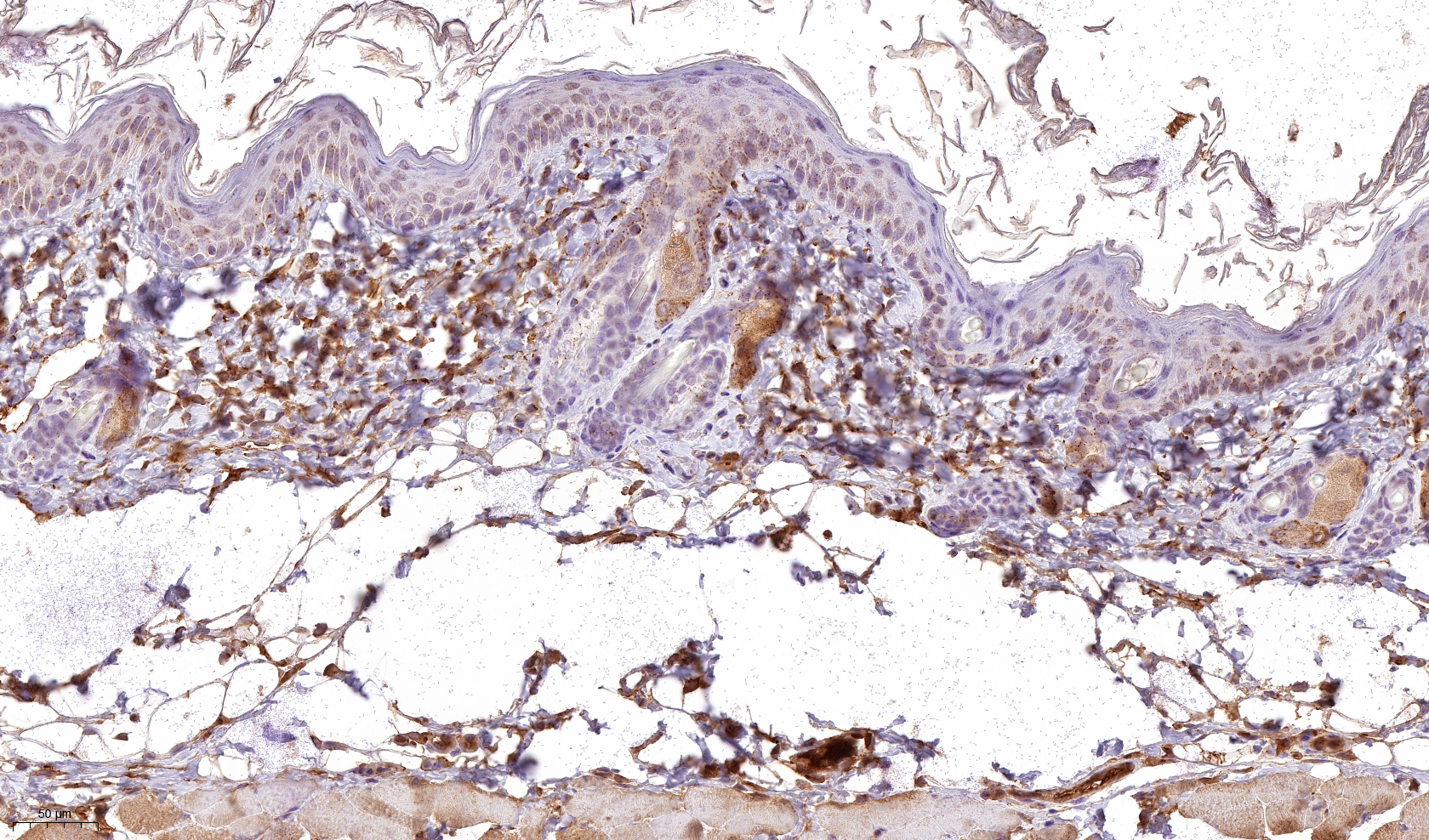

Supplement: Supplementary file 3 — Source Data for Appendix [file EMMM-15-e16758-s005.zip › Figure S1/S1F/7+3_TPA ILEI_20.0x.tif]

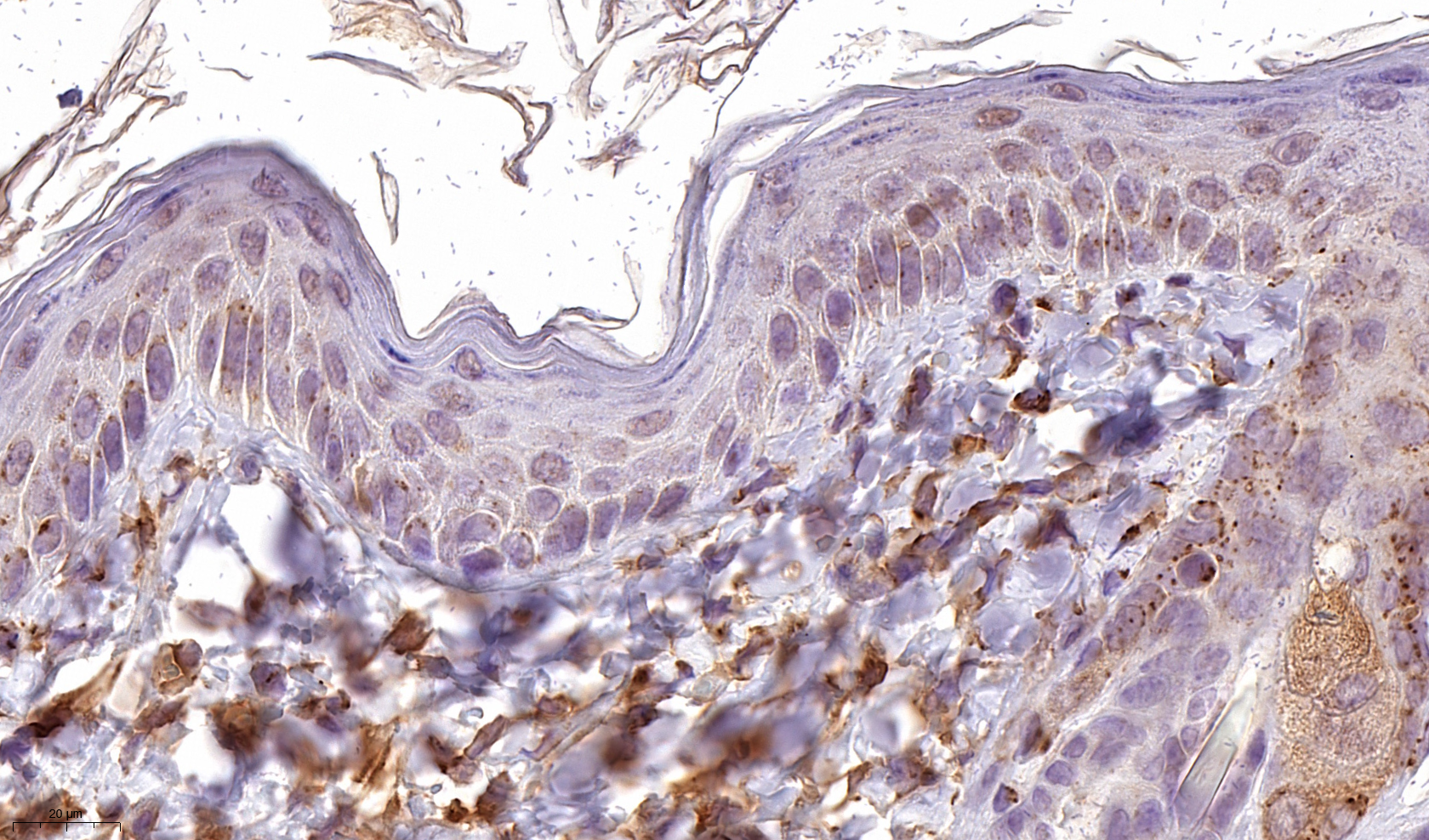

Supplement: Supplementary file 3 — Source Data for Appendix [file EMMM-15-e16758-s005.zip › Figure S1/S1F/7+3_TPA ILEI_63.0x.tif]

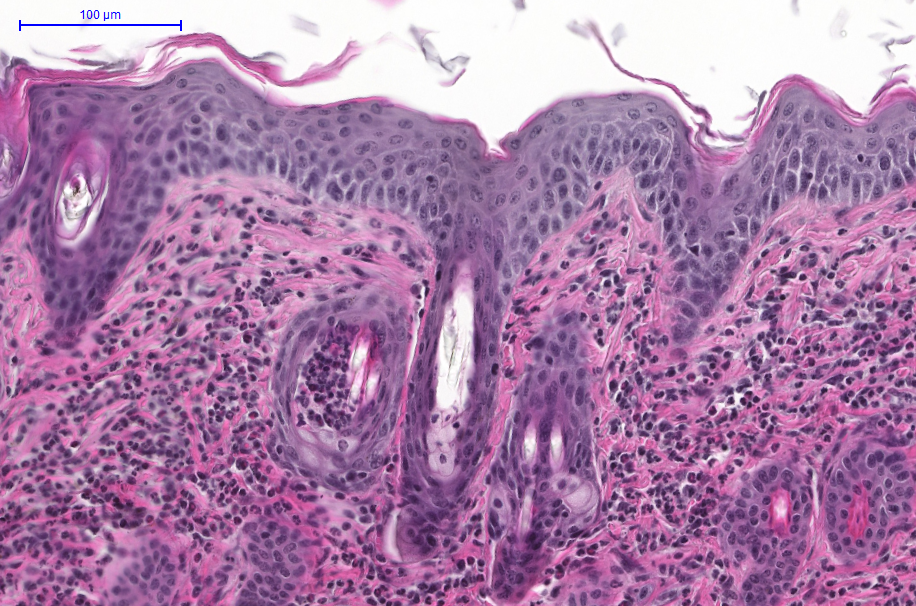

Supplement: Supplementary file 3 — Source Data for Appendix [file EMMM-15-e16758-s005.zip › Figure S1/S1F/7d HE_BL6_002_20x_1.bmp]

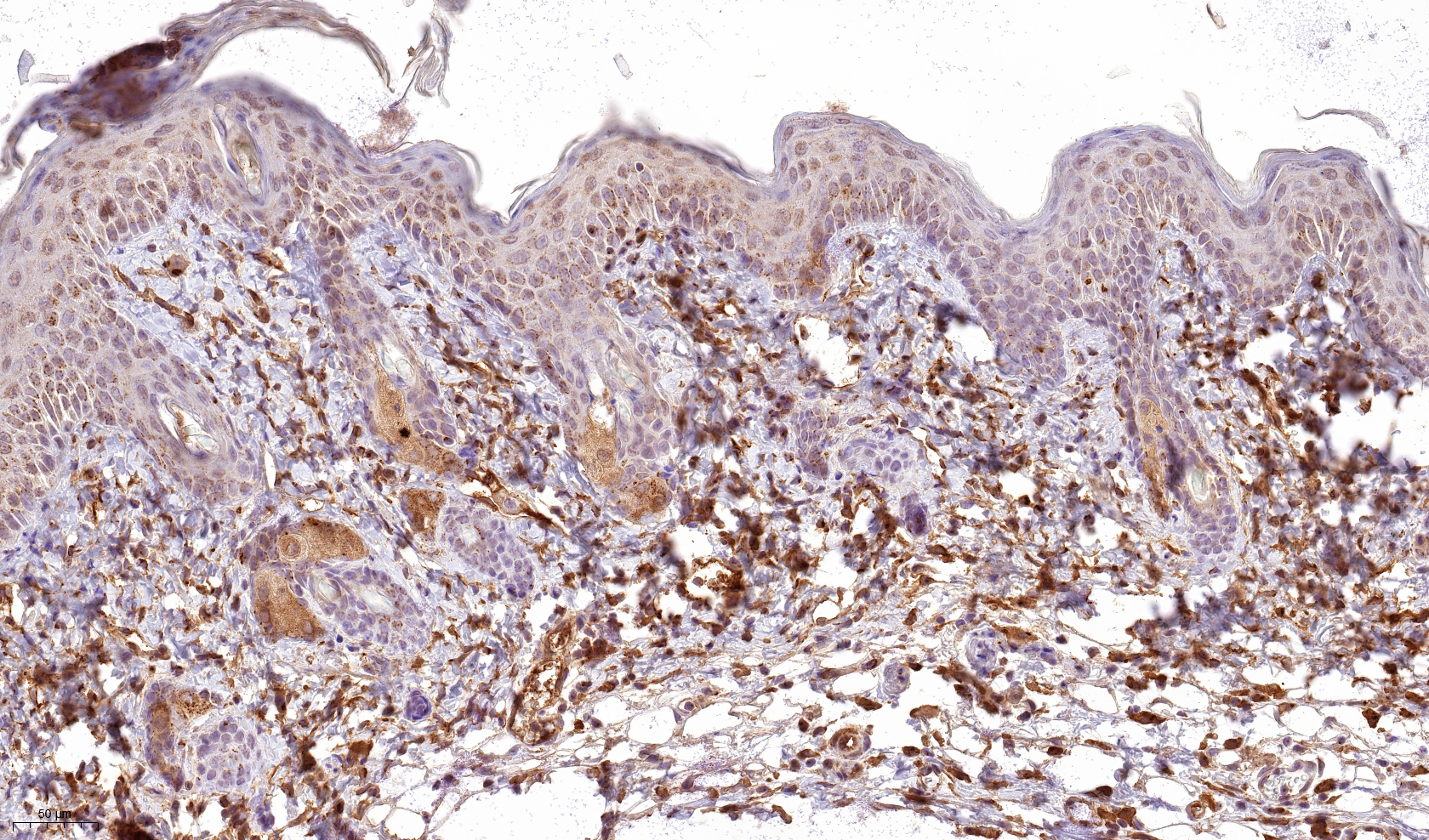

Supplement: Supplementary file 3 — Source Data for Appendix [file EMMM-15-e16758-s005.zip › Figure S1/S1F/7d_TPA ILEI_20.0x.tif]

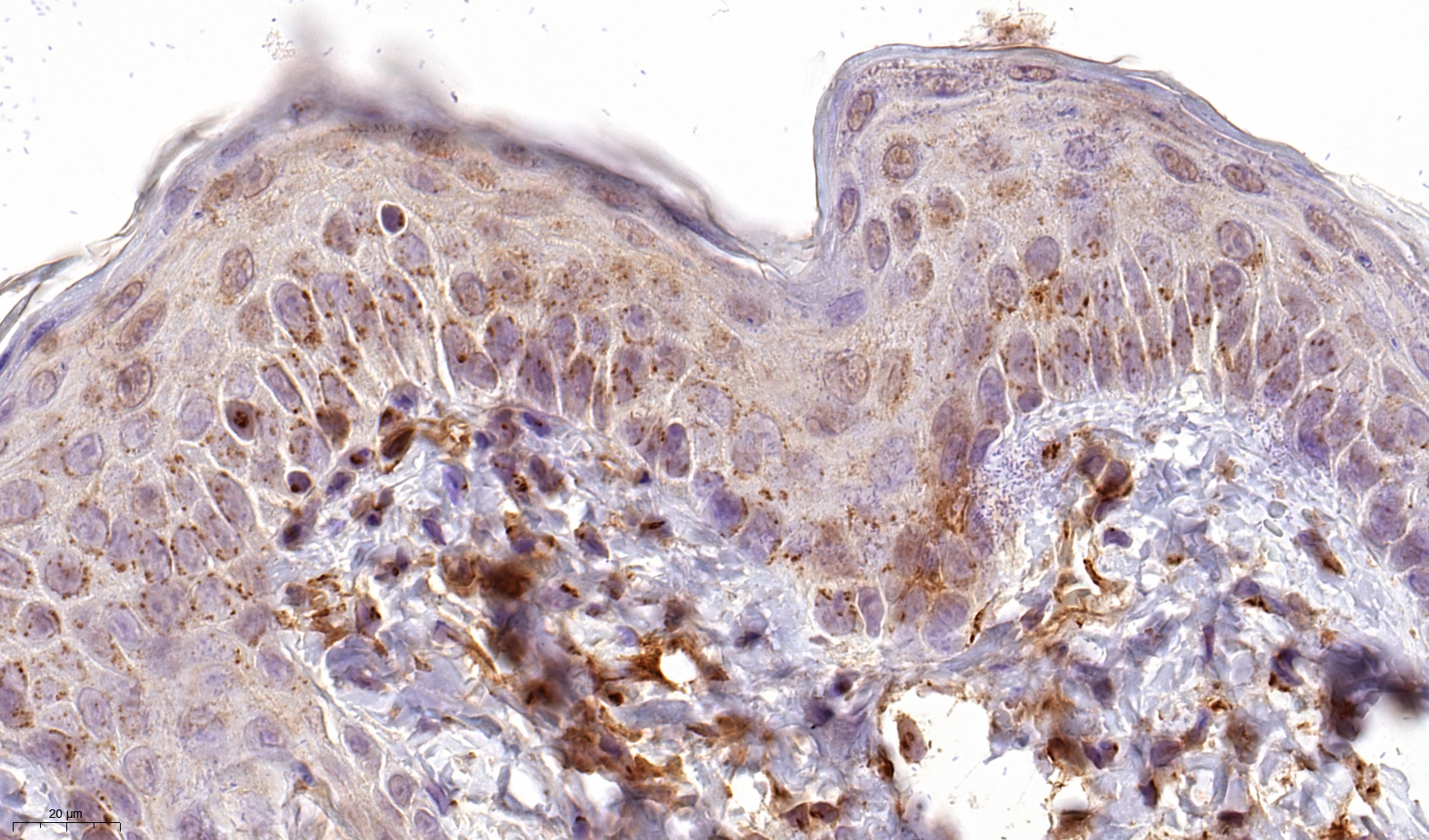

Supplement: Supplementary file 3 — Source Data for Appendix [file EMMM-15-e16758-s005.zip › Figure S1/S1F/7d_TPA ILEI_63.0x.tif]

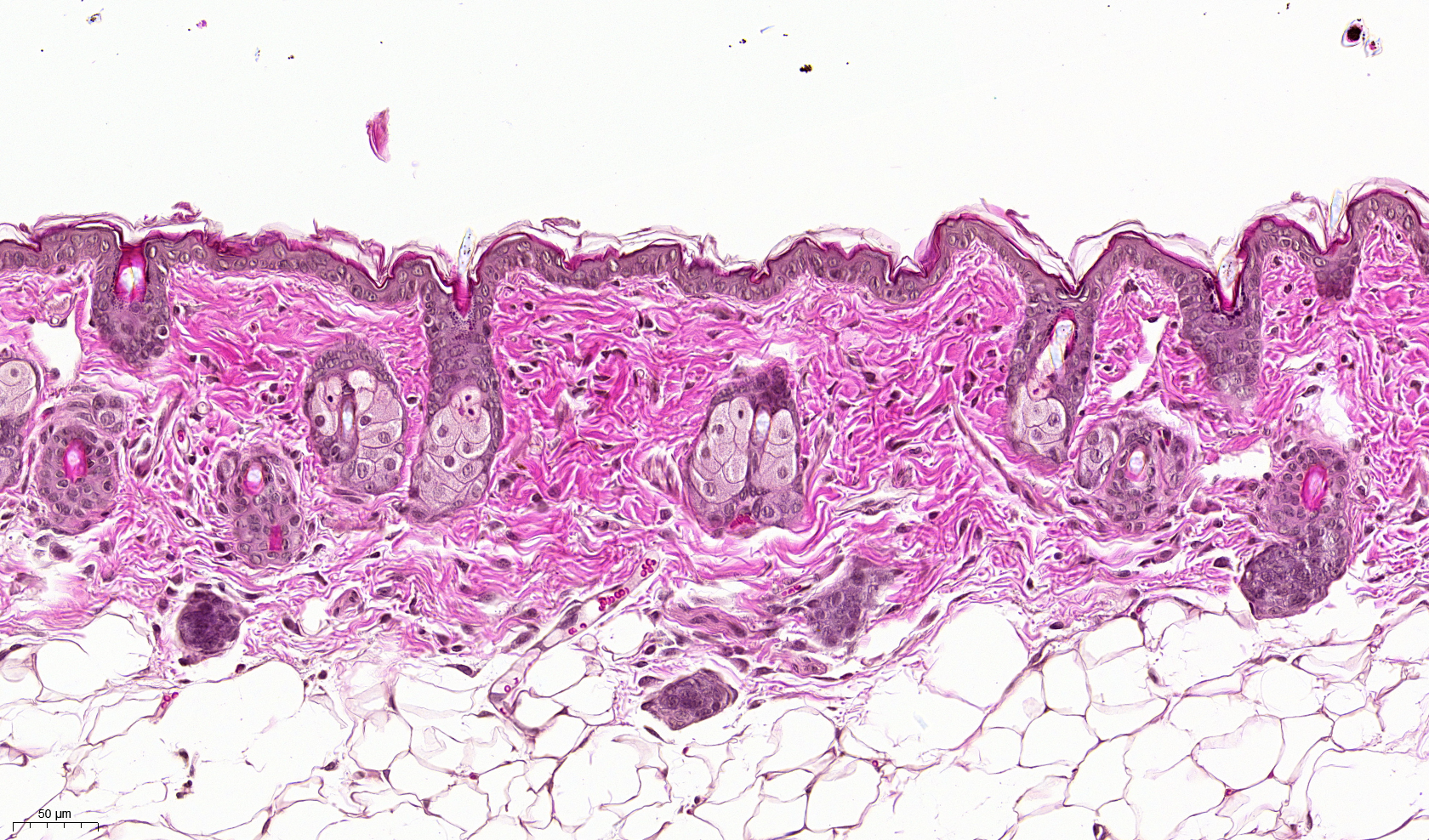

Supplement: Supplementary file 3 — Source Data for Appendix [file EMMM-15-e16758-s005.zip › Figure S1/S1I/0d HE 9-23 d0_20.0x.tif]

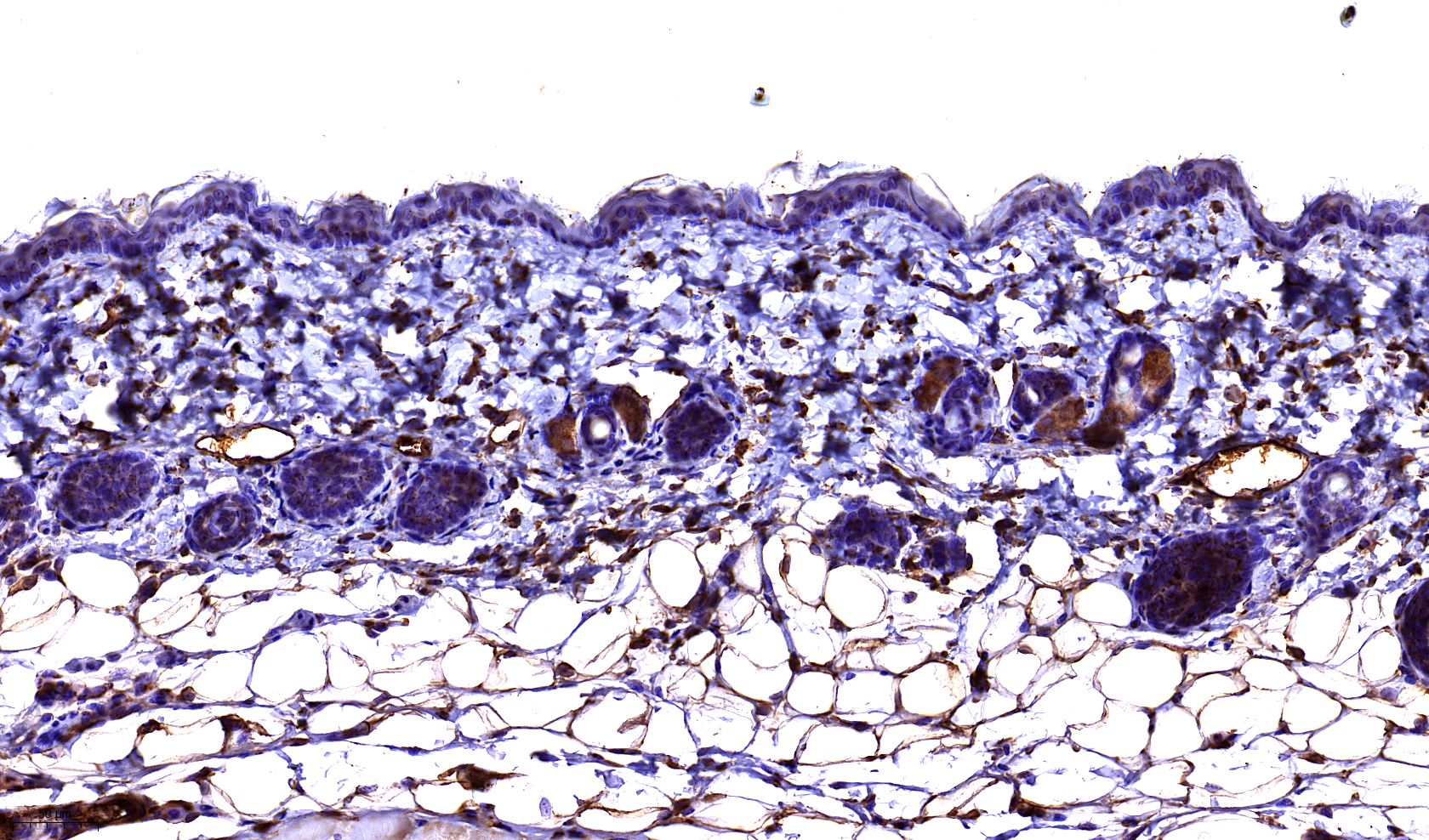

Supplement: Supplementary file 3 — Source Data for Appendix [file EMMM-15-e16758-s005.zip › Figure S1/S1I/0d_IMQ ILEI_20.0x.tif]

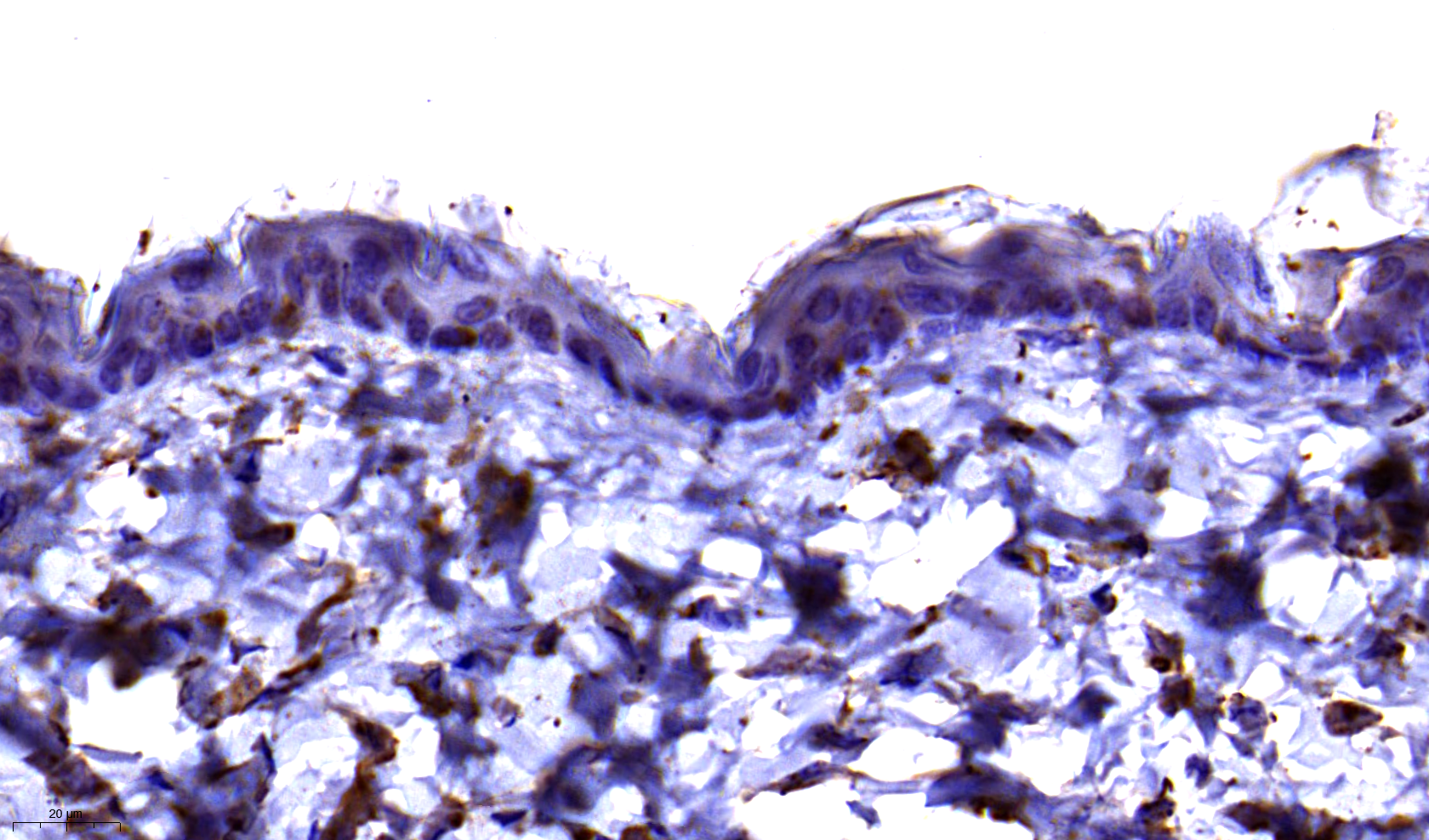

Supplement: Supplementary file 3 — Source Data for Appendix [file EMMM-15-e16758-s005.zip › Figure S1/S1I/0d_IMQ ILEI_63.0x.tif]

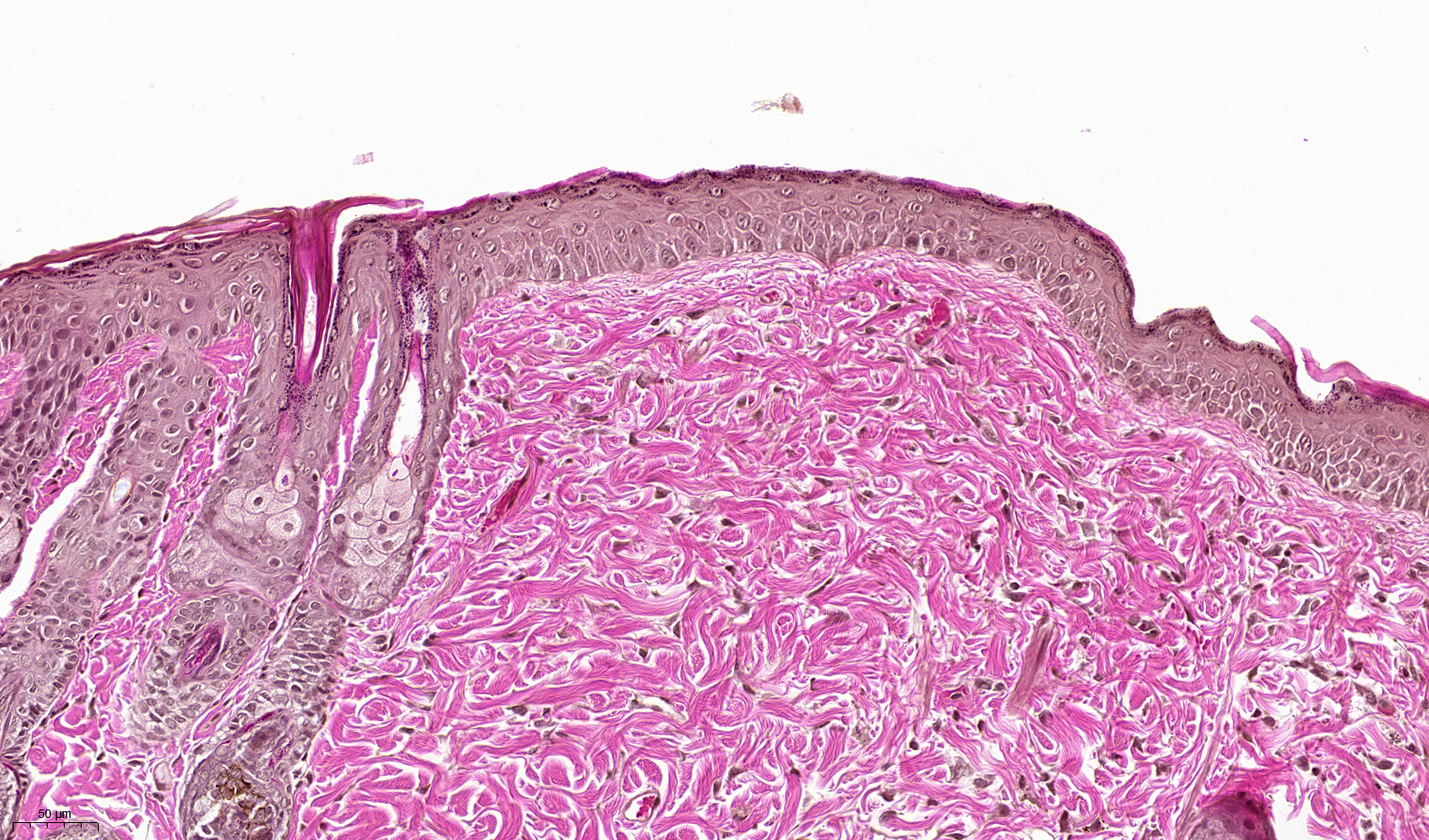

Supplement: Supplementary file 3 — Source Data for Appendix [file EMMM-15-e16758-s005.zip › Figure S1/S1I/3d HE 9-74 d3_20.0x.tif]

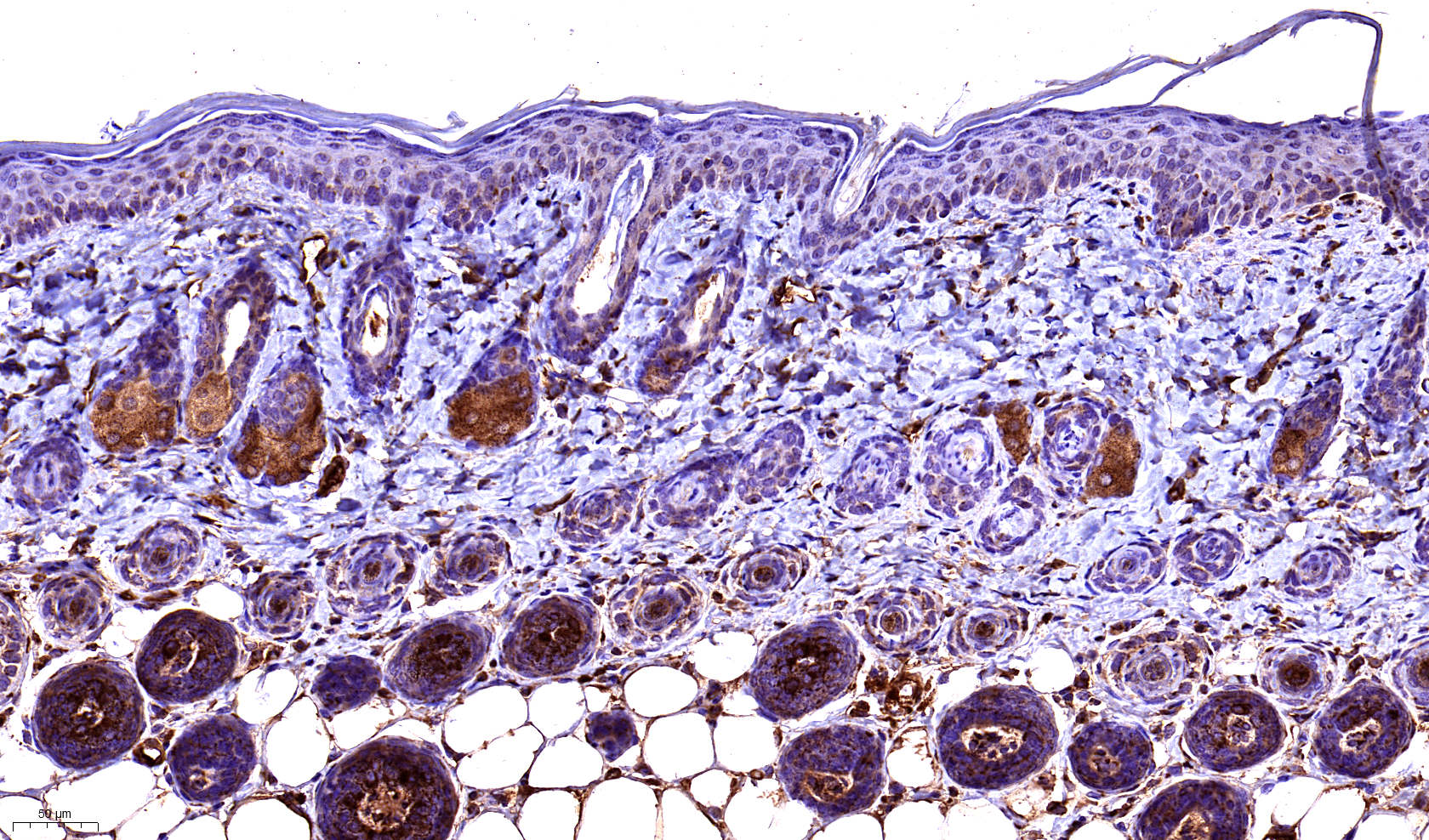

Supplement: Supplementary file 3 — Source Data for Appendix [file EMMM-15-e16758-s005.zip › Figure S1/S1I/3d_IMQ ILEI_20.0x.tif]

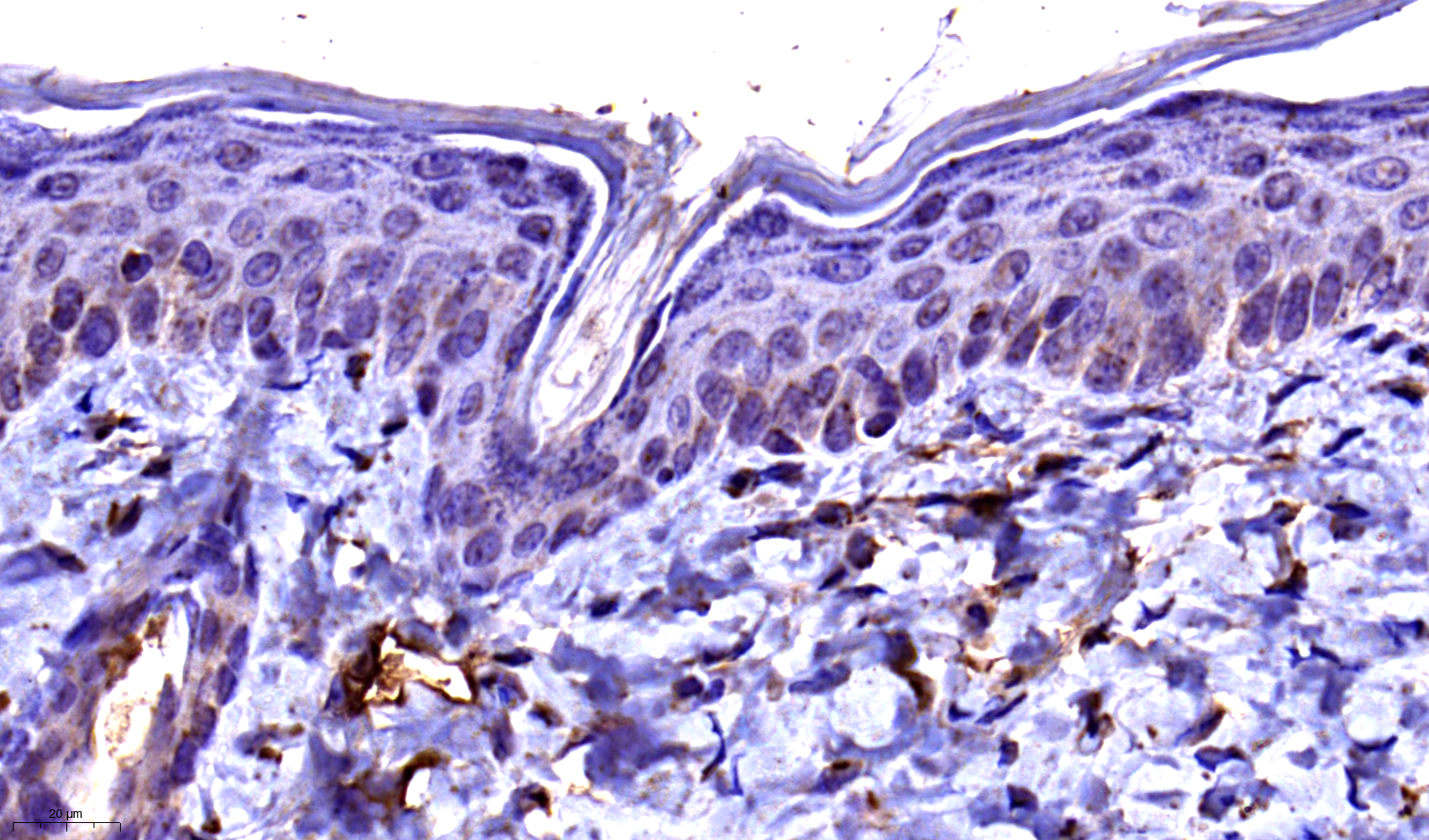

Supplement: Supplementary file 3 — Source Data for Appendix [file EMMM-15-e16758-s005.zip › Figure S1/S1I/3d_IMQ ILEI_63.0x.tif]

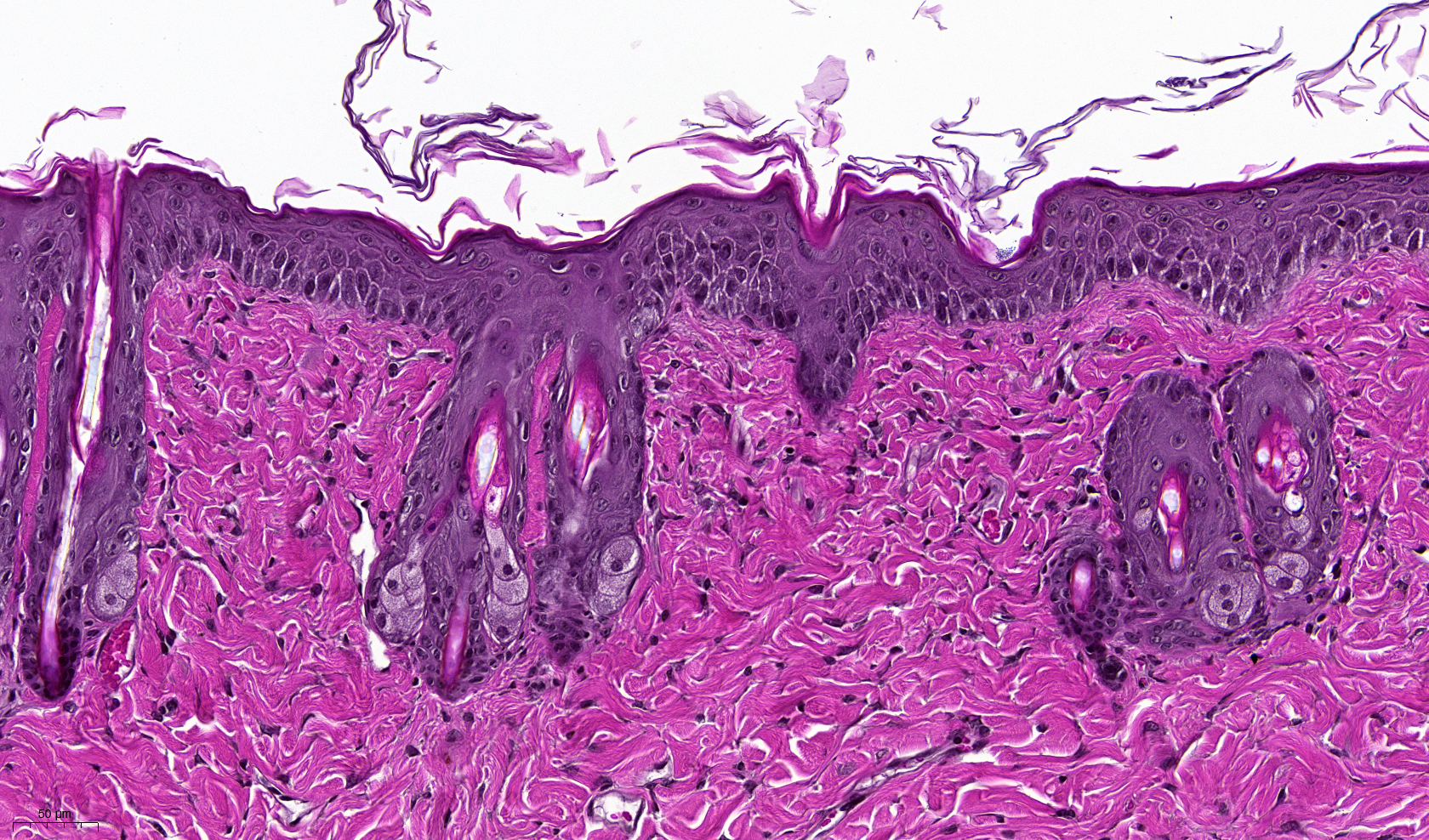

Supplement: Supplementary file 3 — Source Data for Appendix [file EMMM-15-e16758-s005.zip › Figure S1/S1I/5d HE 9-721 d5_20.0x_2.tif]

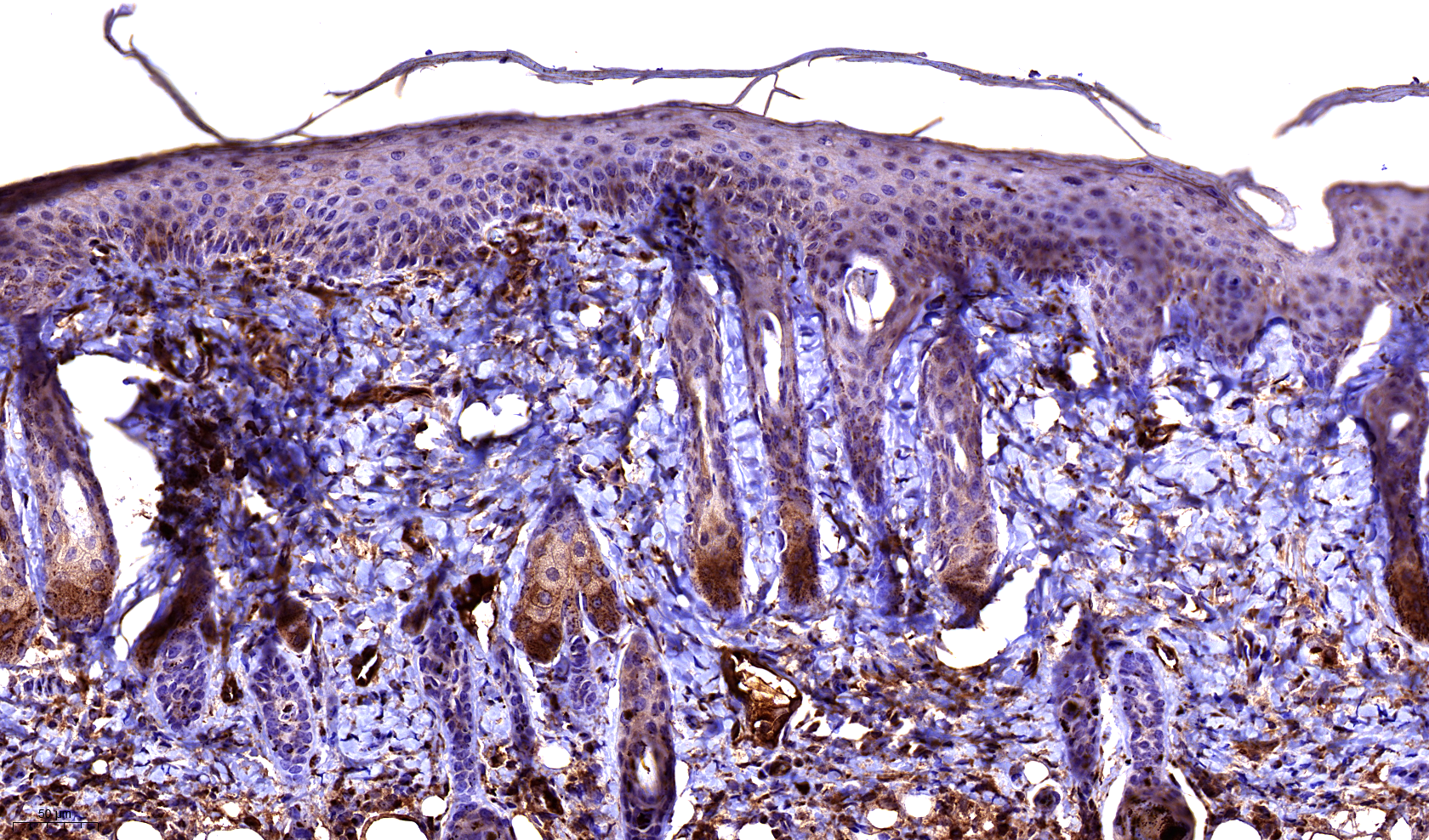

Supplement: Supplementary file 3 — Source Data for Appendix [file EMMM-15-e16758-s005.zip › Figure S1/S1I/5d_IMQ ILEI_20.0x.tif]

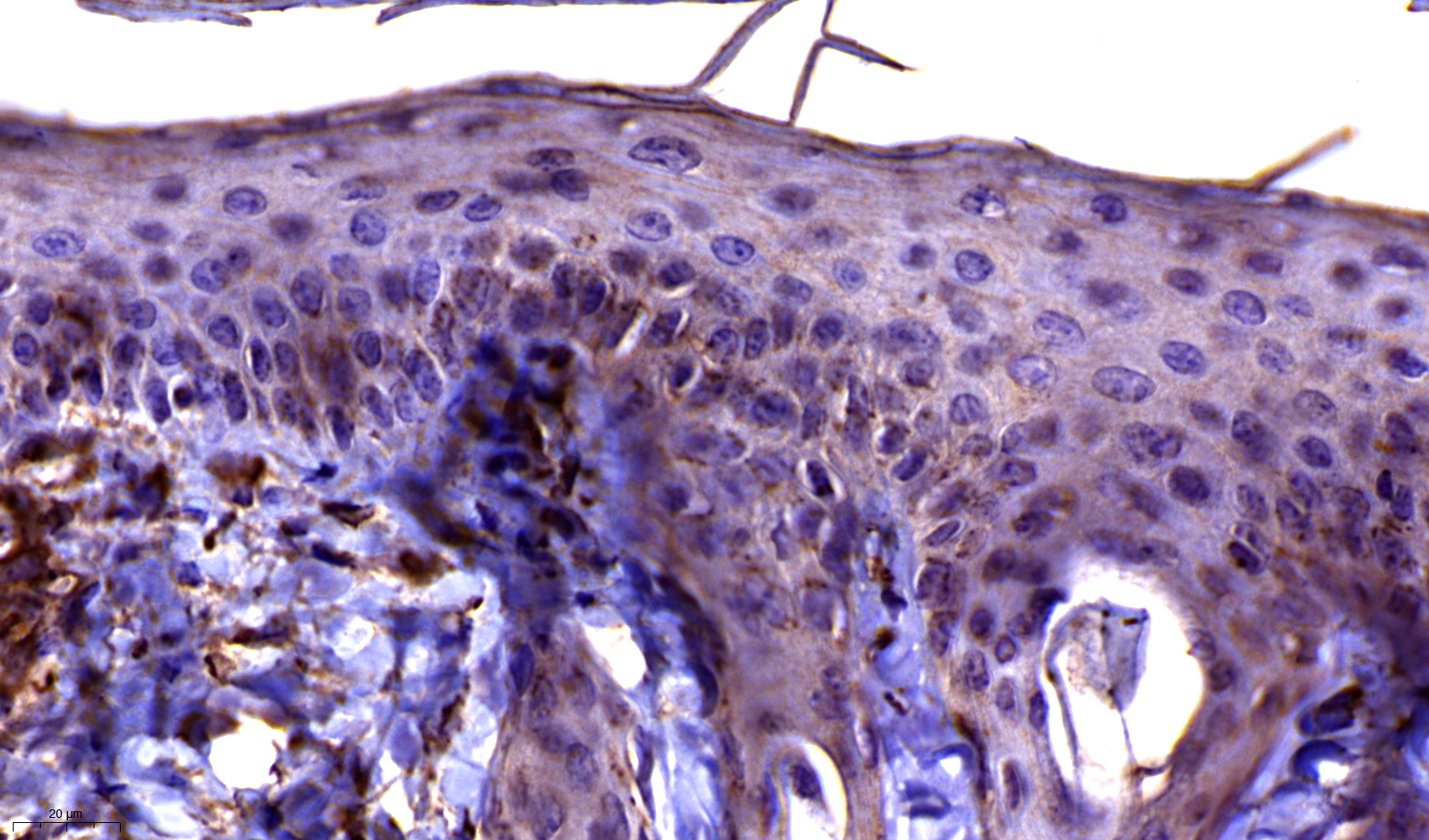

Supplement: Supplementary file 3 — Source Data for Appendix [file EMMM-15-e16758-s005.zip › Figure S1/S1I/5d_IMQ ILEI_63.0x.tif]

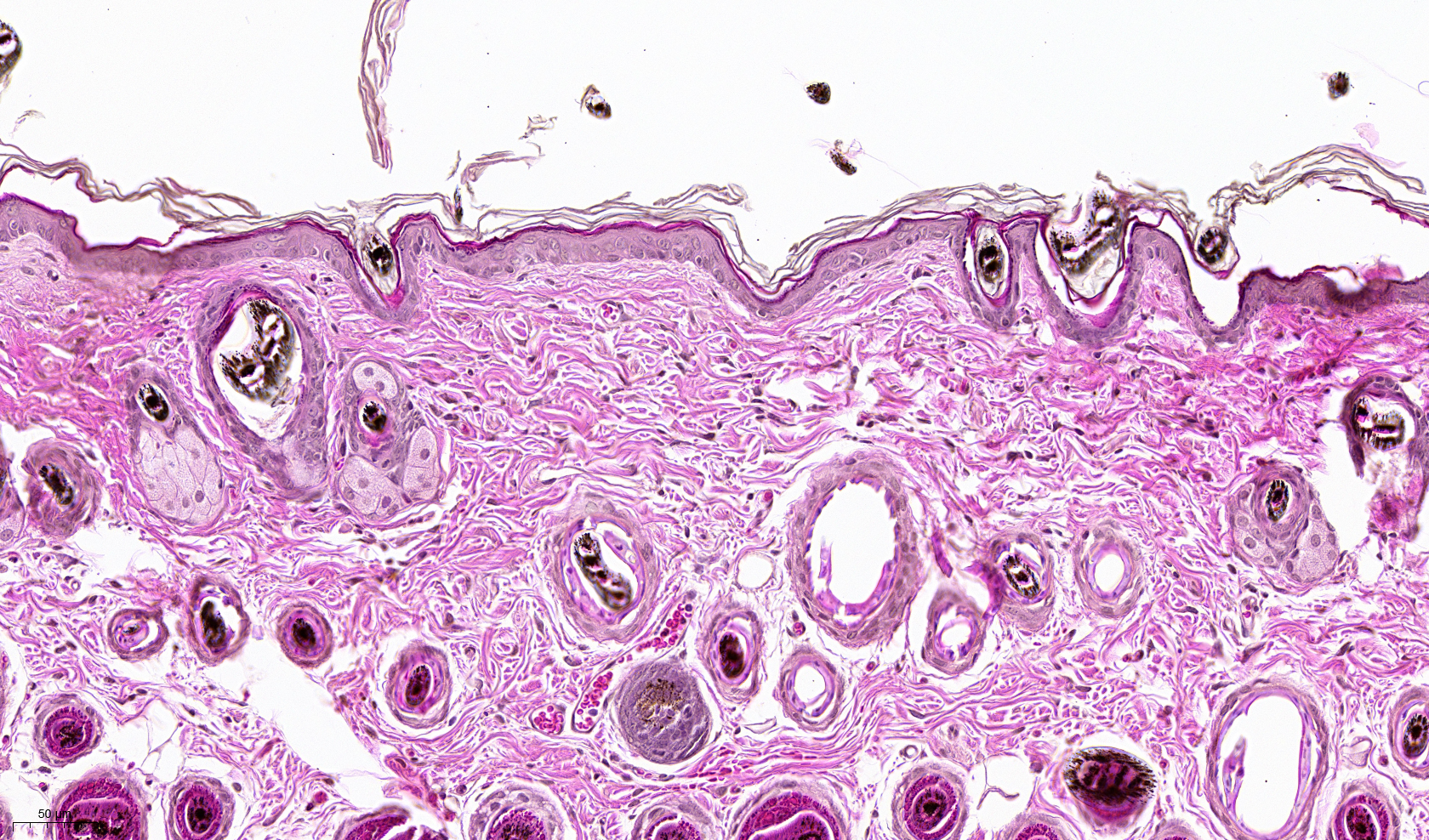

Supplement: Supplementary file 3 — Source Data for Appendix [file EMMM-15-e16758-s005.zip › Figure S1/S1I/7+3d HE 9-206 d7+3_20.0x.tif]

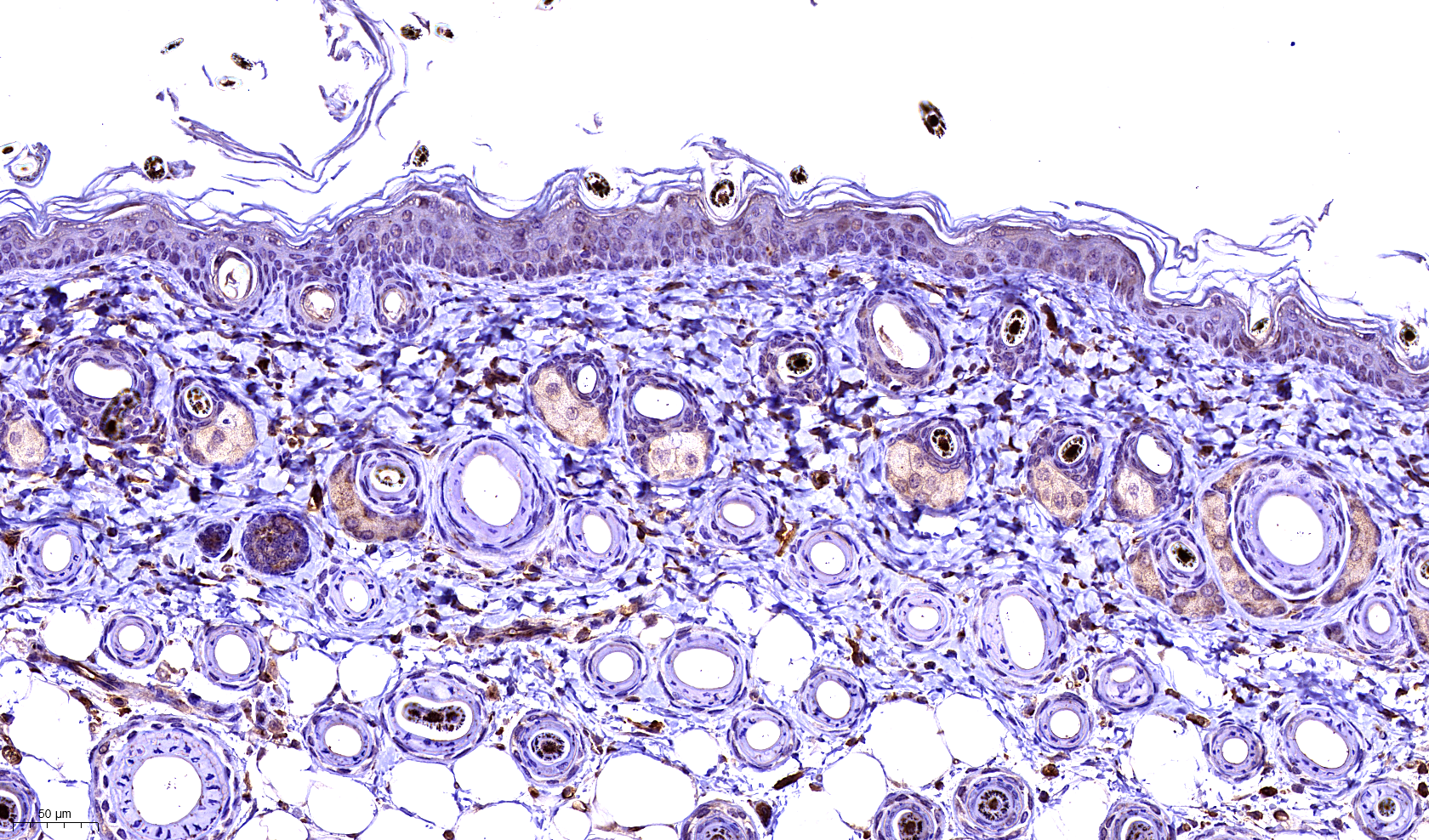

Supplement: Supplementary file 3 — Source Data for Appendix [file EMMM-15-e16758-s005.zip › Figure S1/S1I/7+3d_IMQ ILEI_20.0x.tif]

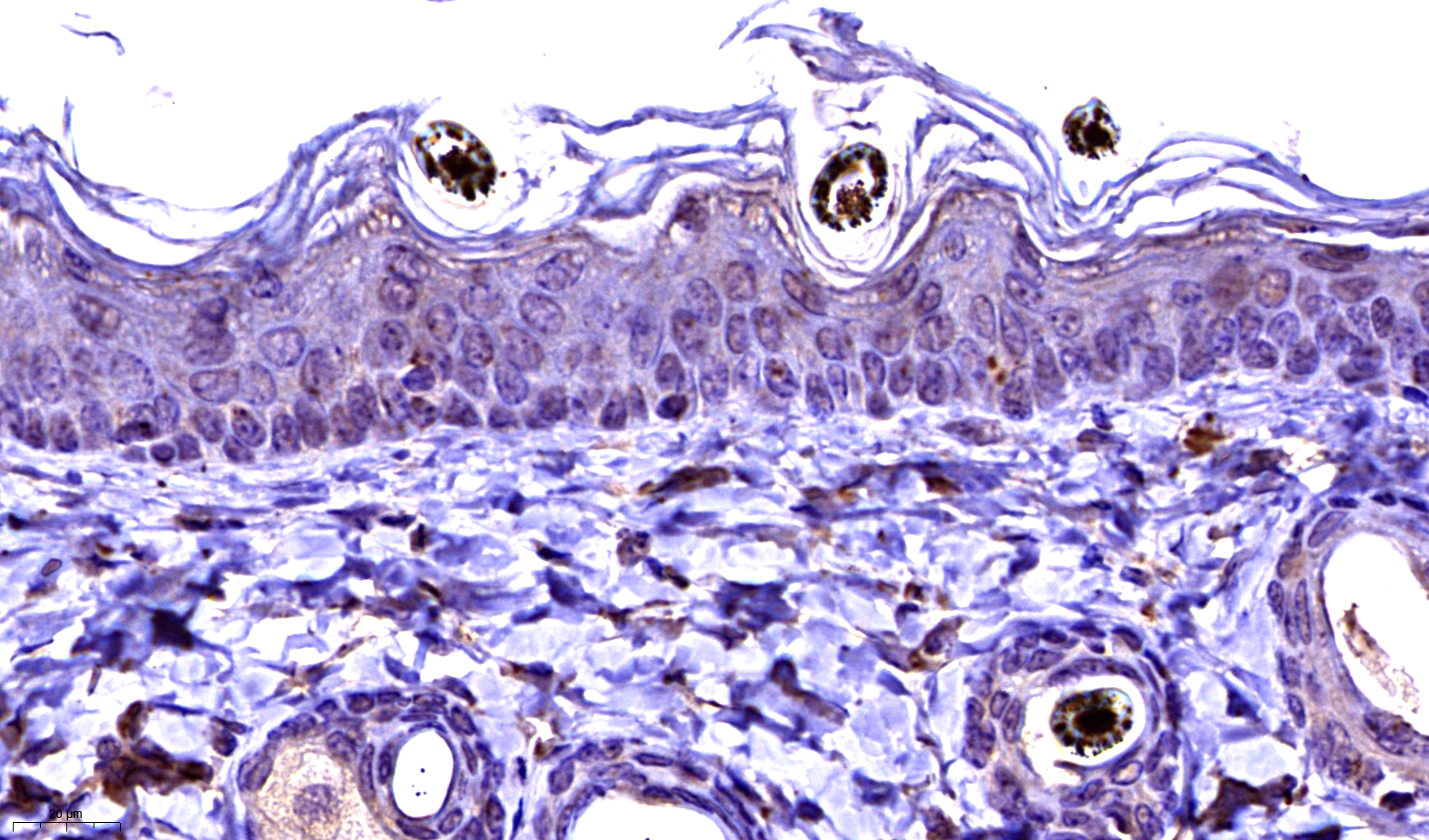

Supplement: Supplementary file 3 — Source Data for Appendix [file EMMM-15-e16758-s005.zip › Figure S1/S1I/7+3d_IMQ ILEI_63.0x.tif]

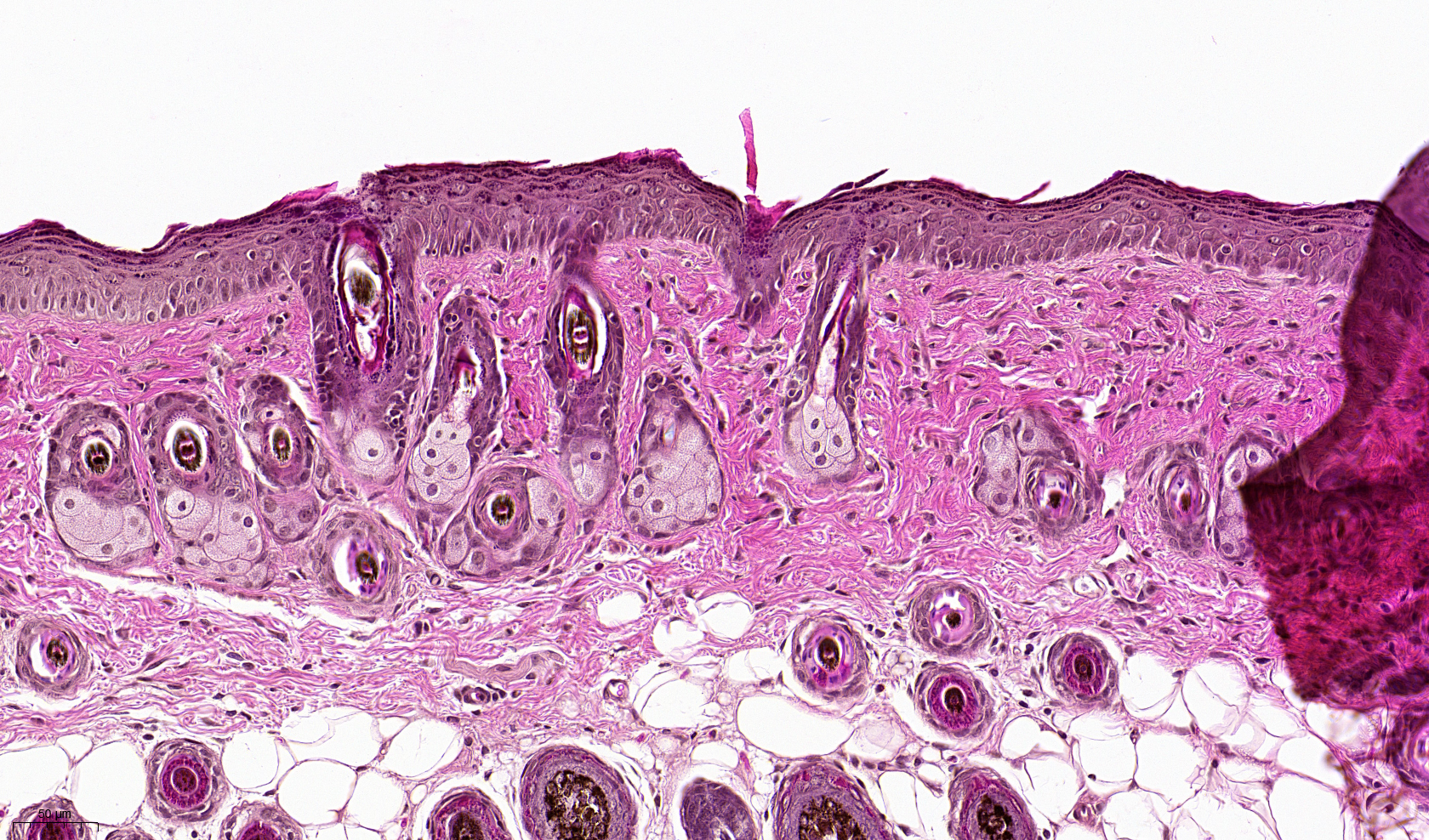

Supplement: Supplementary file 3 — Source Data for Appendix [file EMMM-15-e16758-s005.zip › Figure S1/S1I/7d HE 9-110 d7_20.0x.tif]

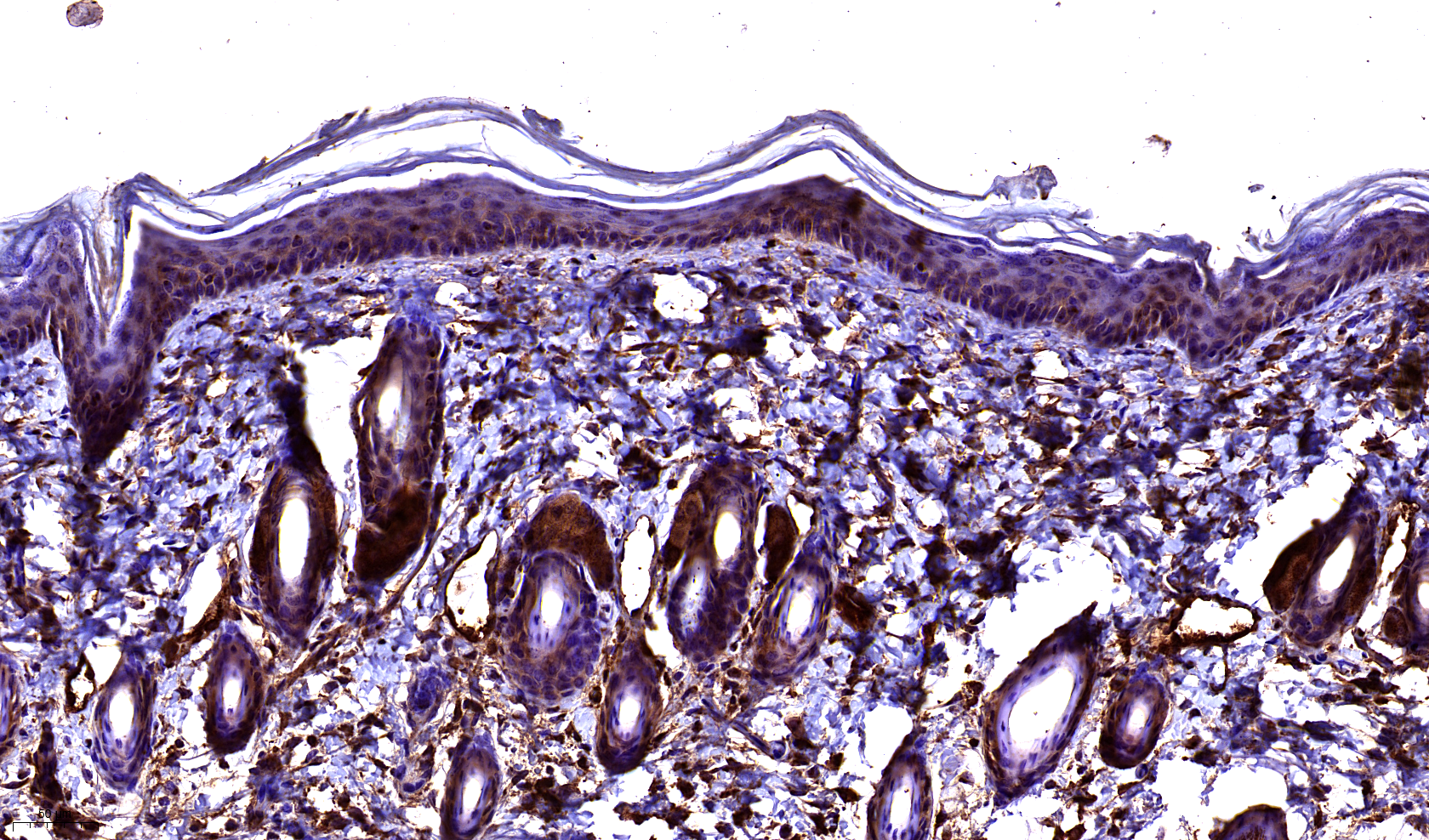

Supplement: Supplementary file 3 — Source Data for Appendix [file EMMM-15-e16758-s005.zip › Figure S1/S1I/7d_IMQ ILEI_20.0x.tif]

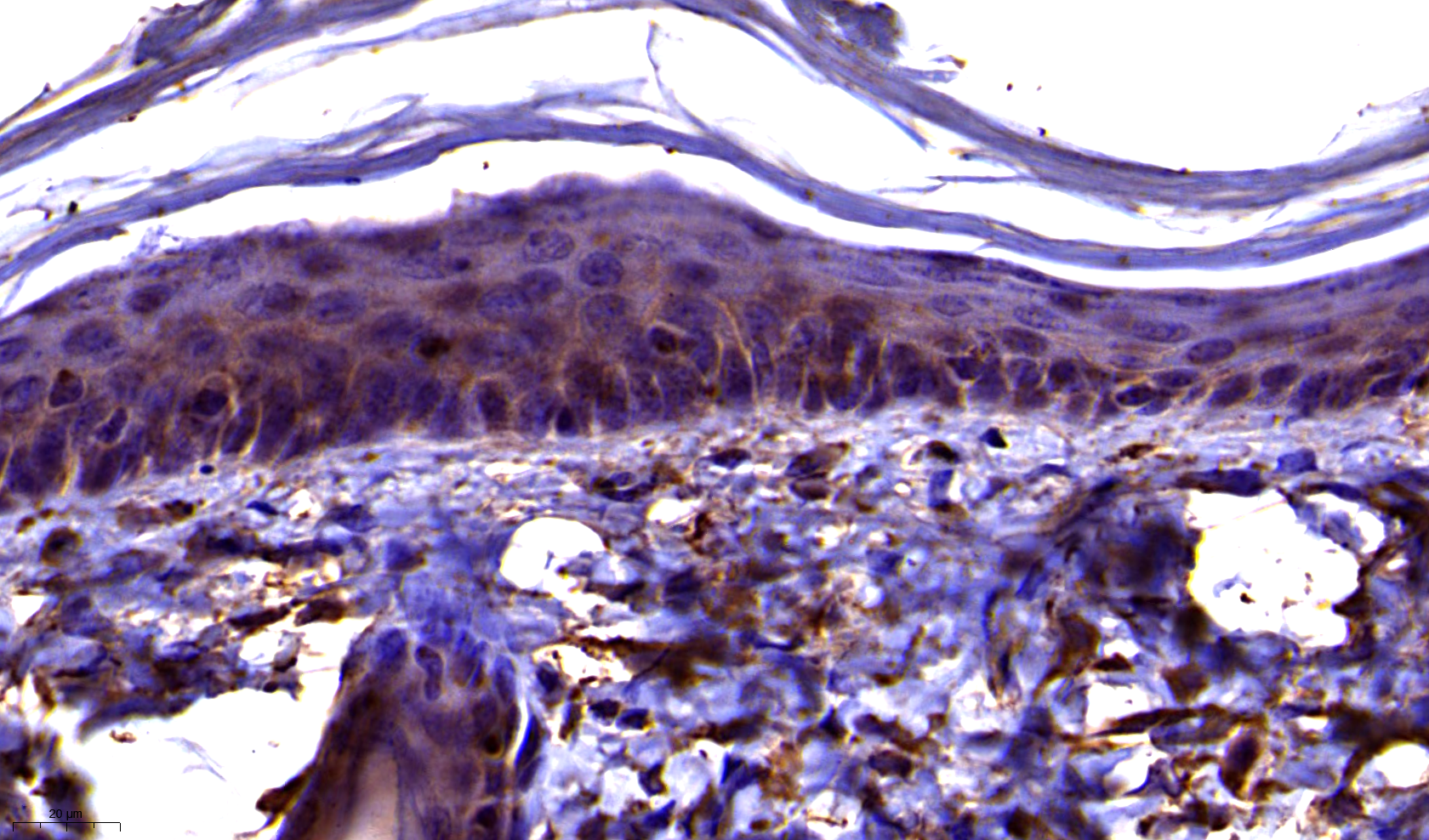

Supplement: Supplementary file 3 — Source Data for Appendix [file EMMM-15-e16758-s005.zip › Figure S1/S1I/7d_IMQ ILEI_63.0x.tif]

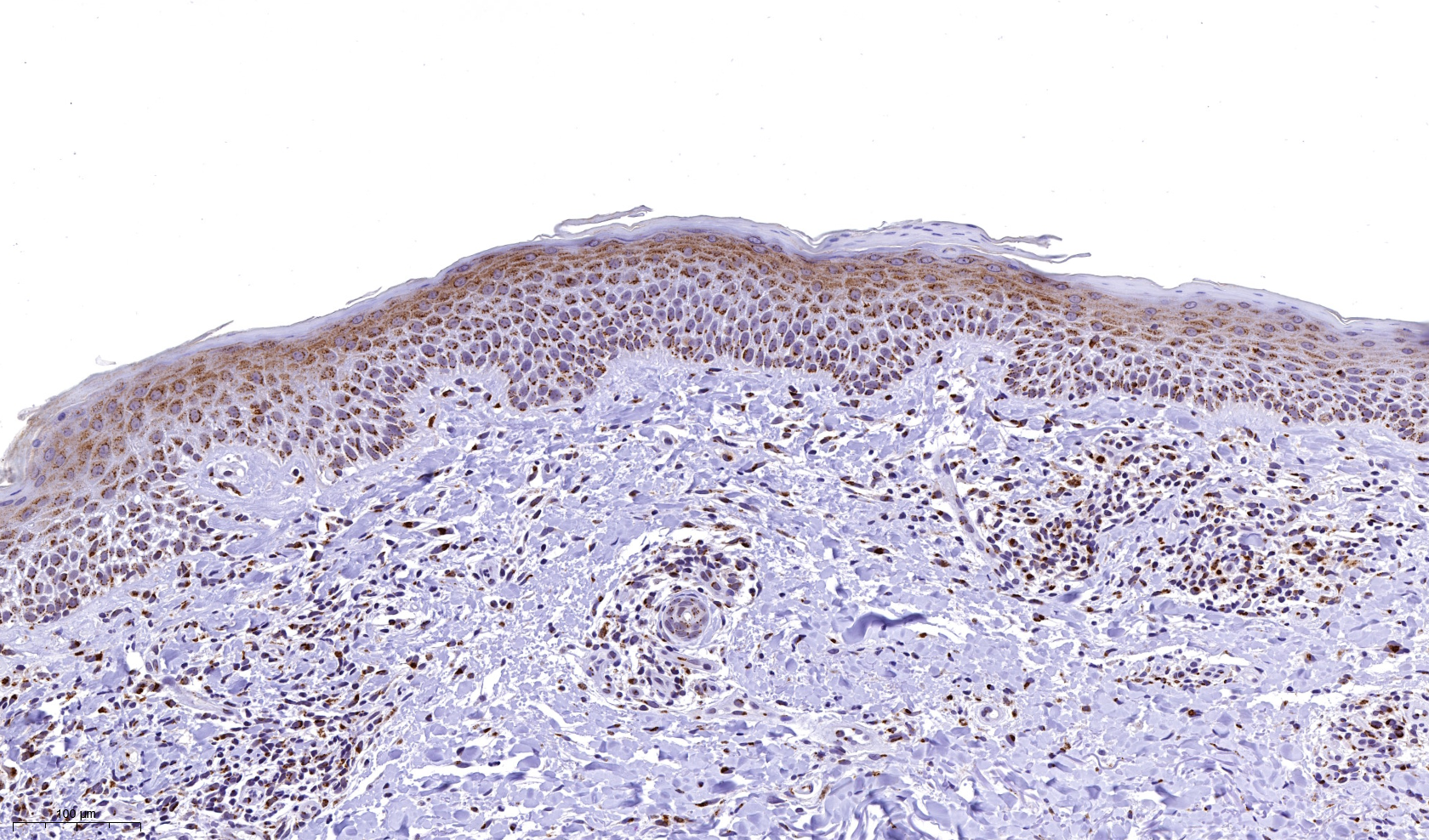

Supplement: Supplementary file 3 — Source Data for Appendix [file EMMM-15-e16758-s005.zip › Figure S8/AD 1541_15_15.0x 20230221.tif]

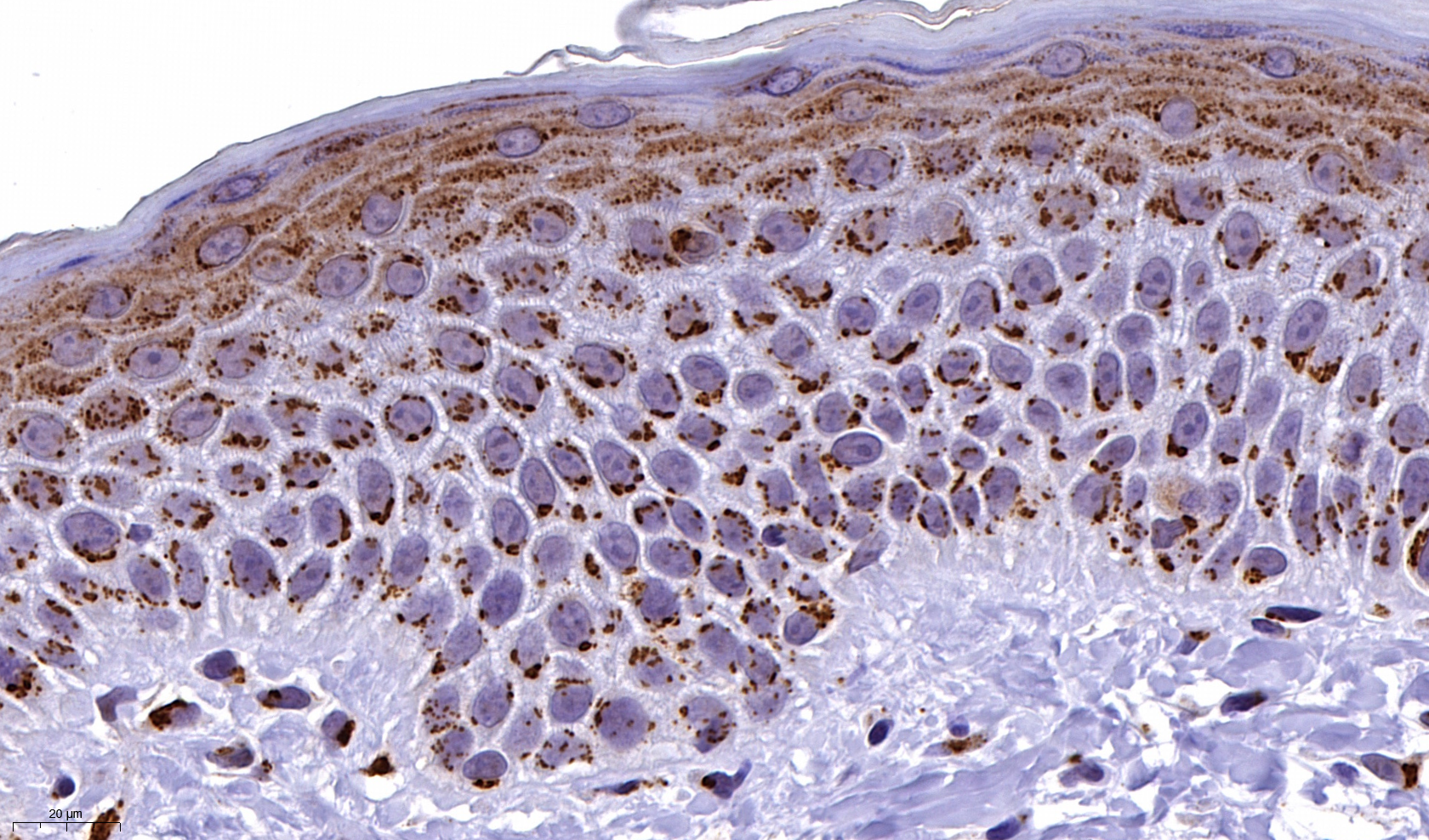

Supplement: Supplementary file 3 — Source Data for Appendix [file EMMM-15-e16758-s005.zip › Figure S8/AD 1541_15_63.0x.tif]

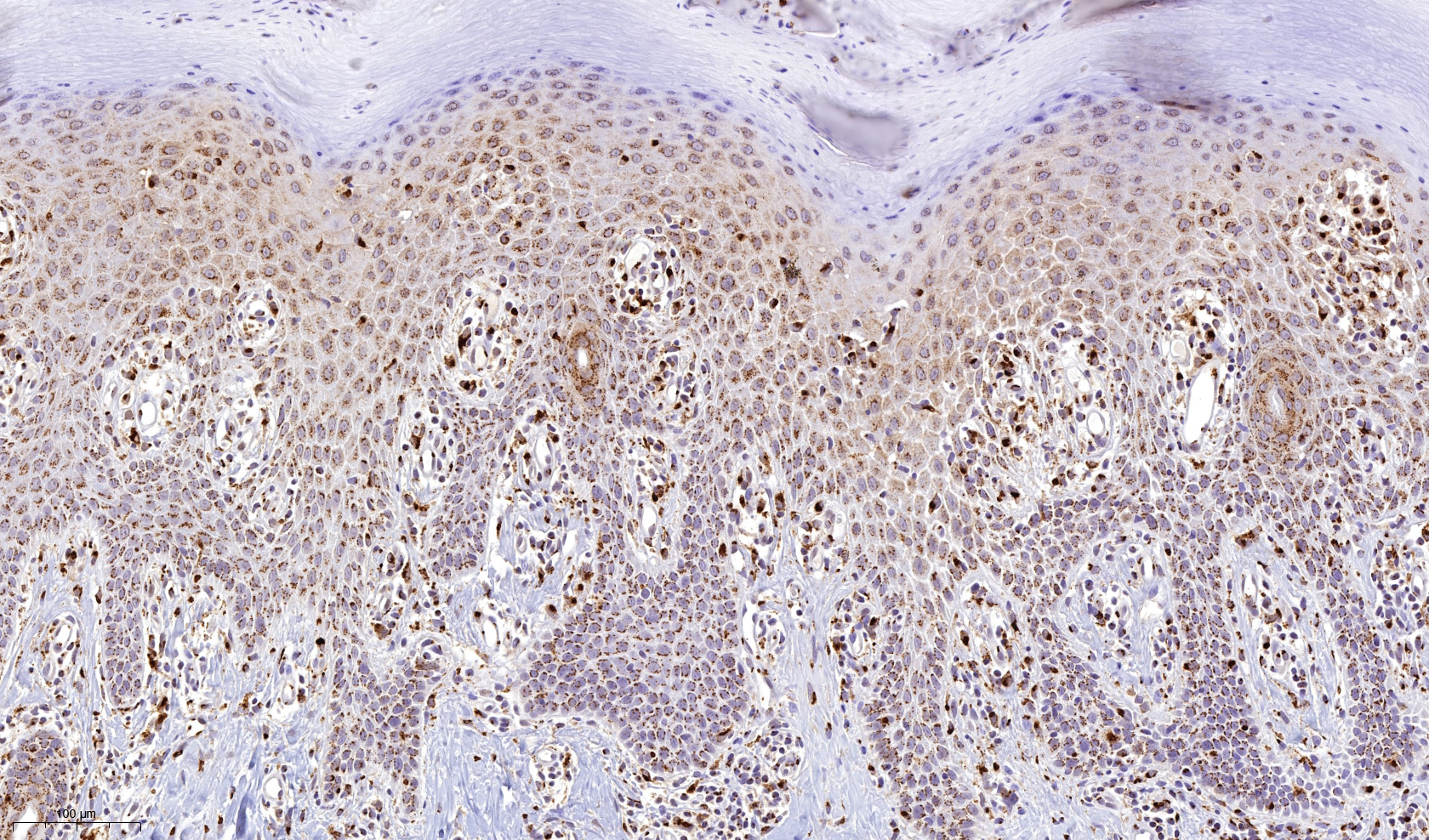

Supplement: Supplementary file 3 — Source Data for Appendix [file EMMM-15-e16758-s005.zip › Figure S8/AD 2631_12_15.0x 20230221.tif]

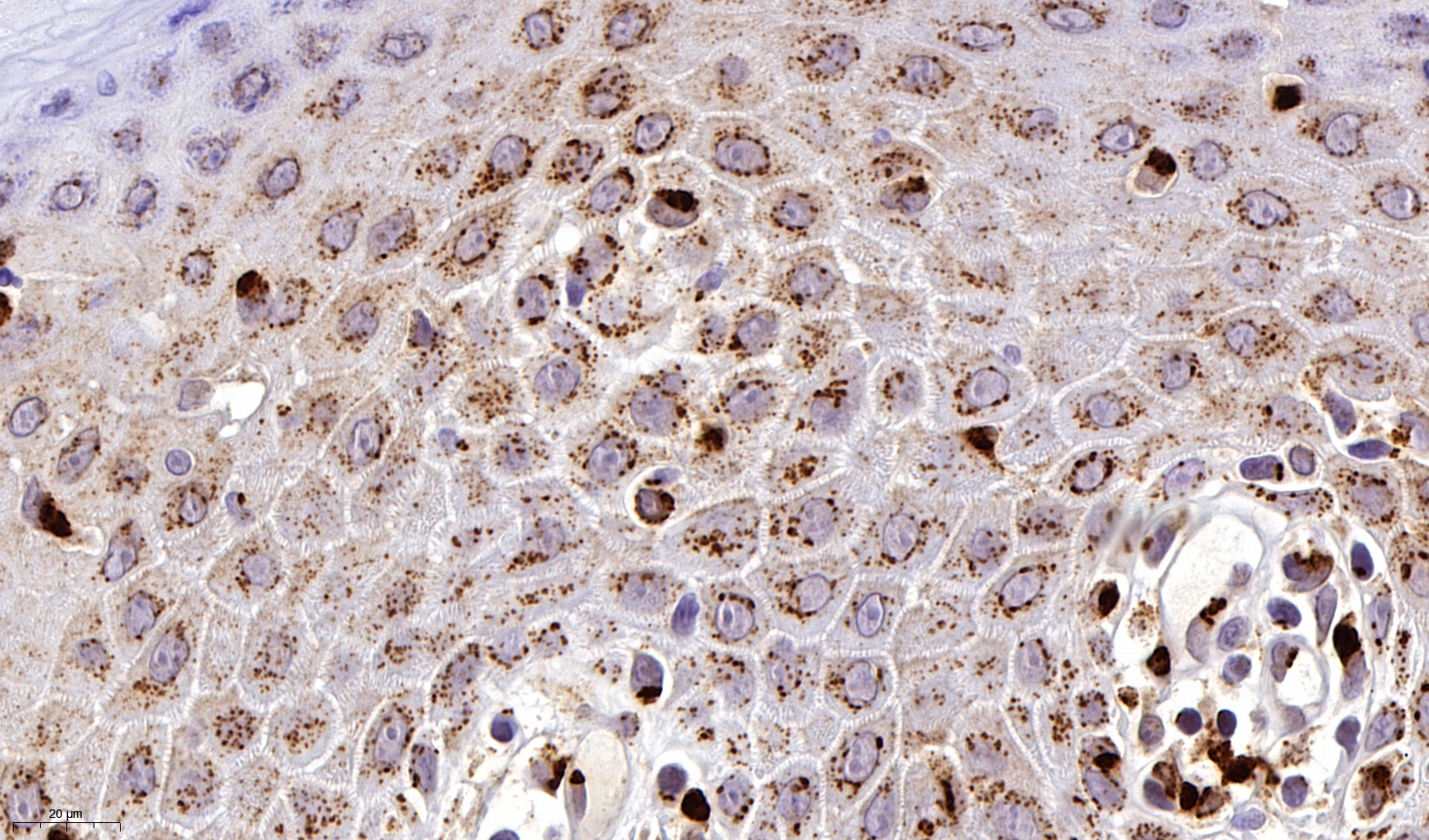

Supplement: Supplementary file 3 — Source Data for Appendix [file EMMM-15-e16758-s005.zip › Figure S8/AD 2631_12_63.0x.tif]

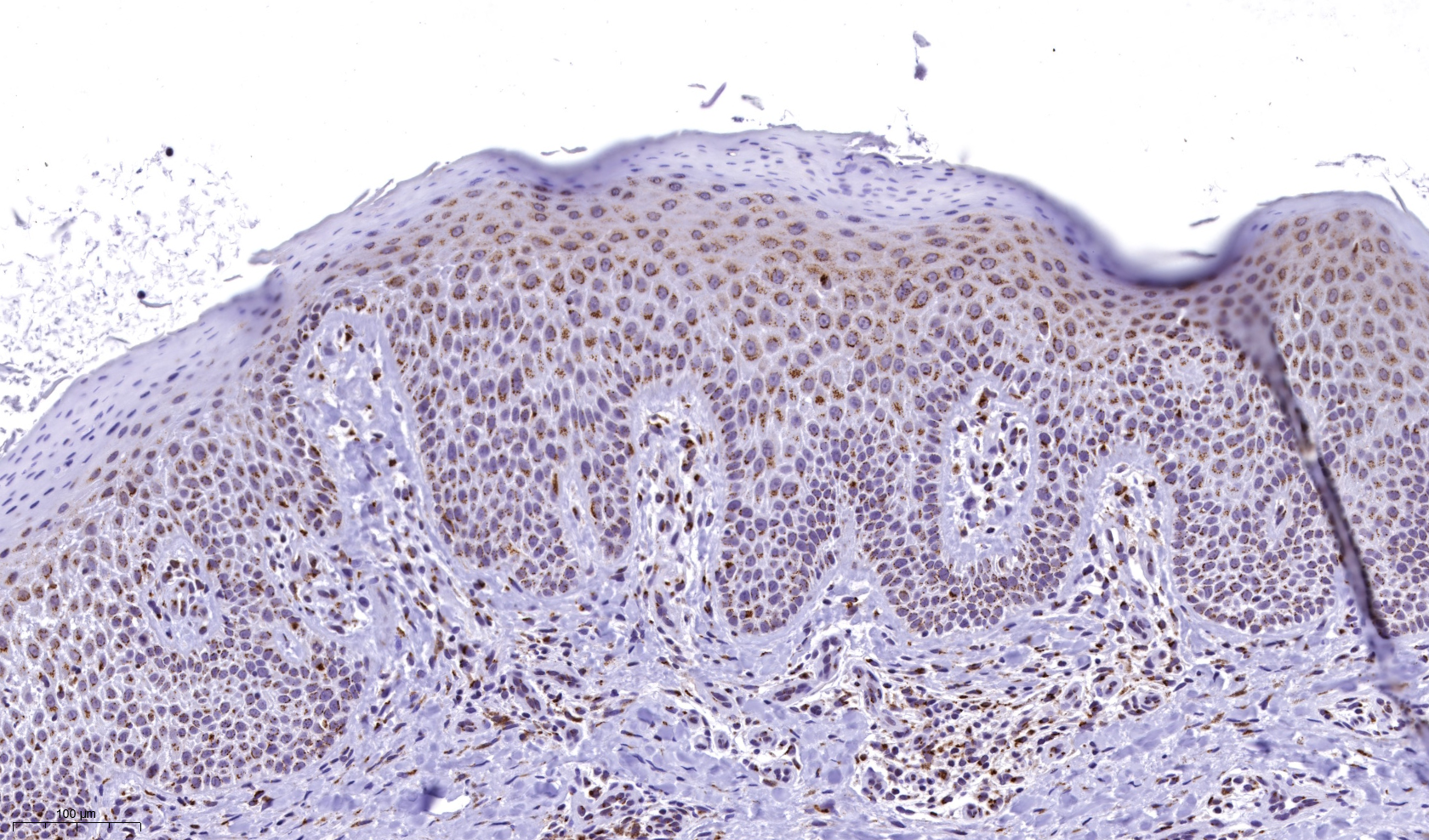

Supplement: Supplementary file 3 — Source Data for Appendix [file EMMM-15-e16758-s005.zip › Figure S8/AD 3357_14_15.0x 20230221.tif]

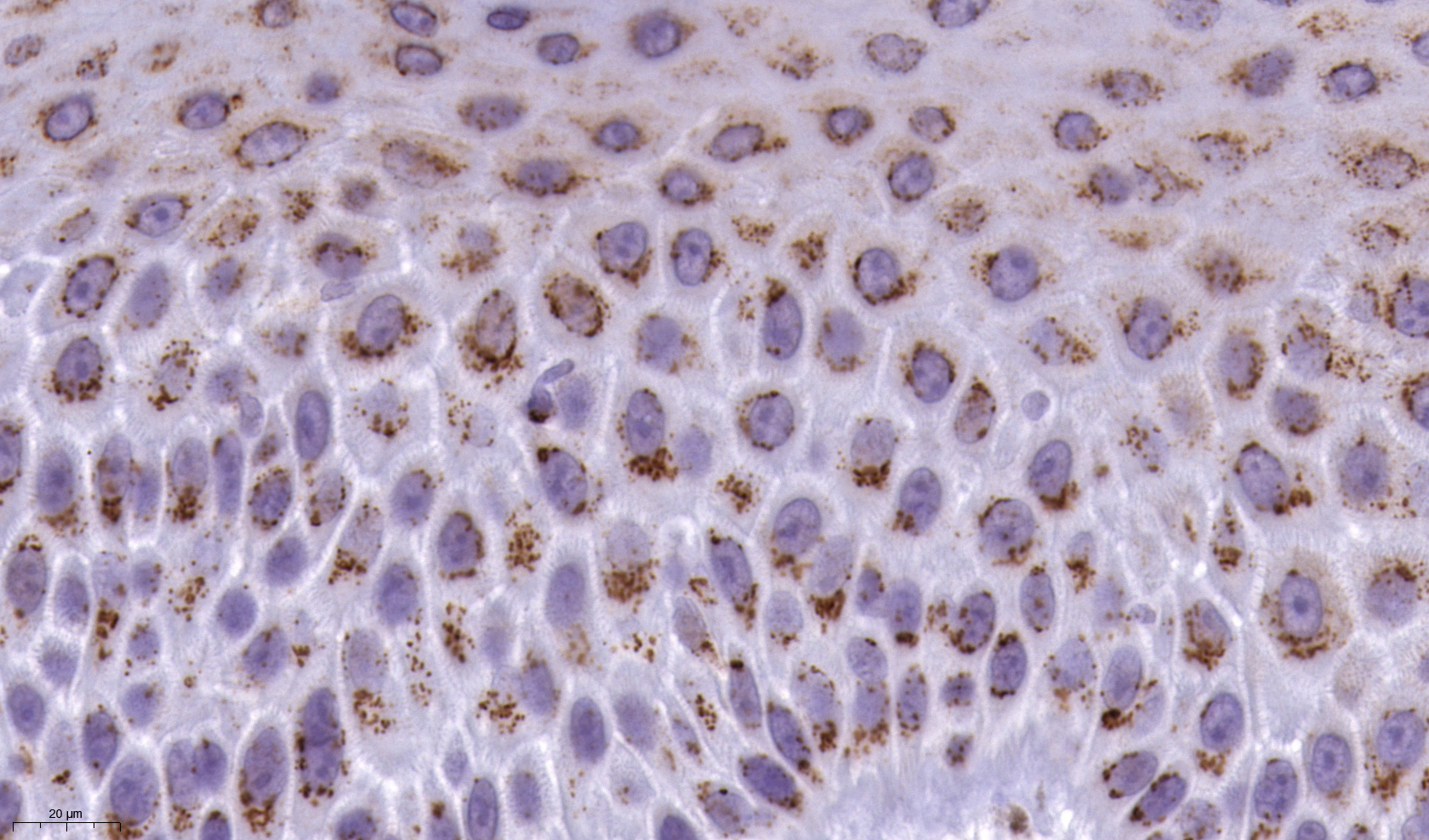

Supplement: Supplementary file 3 — Source Data for Appendix [file EMMM-15-e16758-s005.zip › Figure S8/AD 3357_14_63.0x.tif]

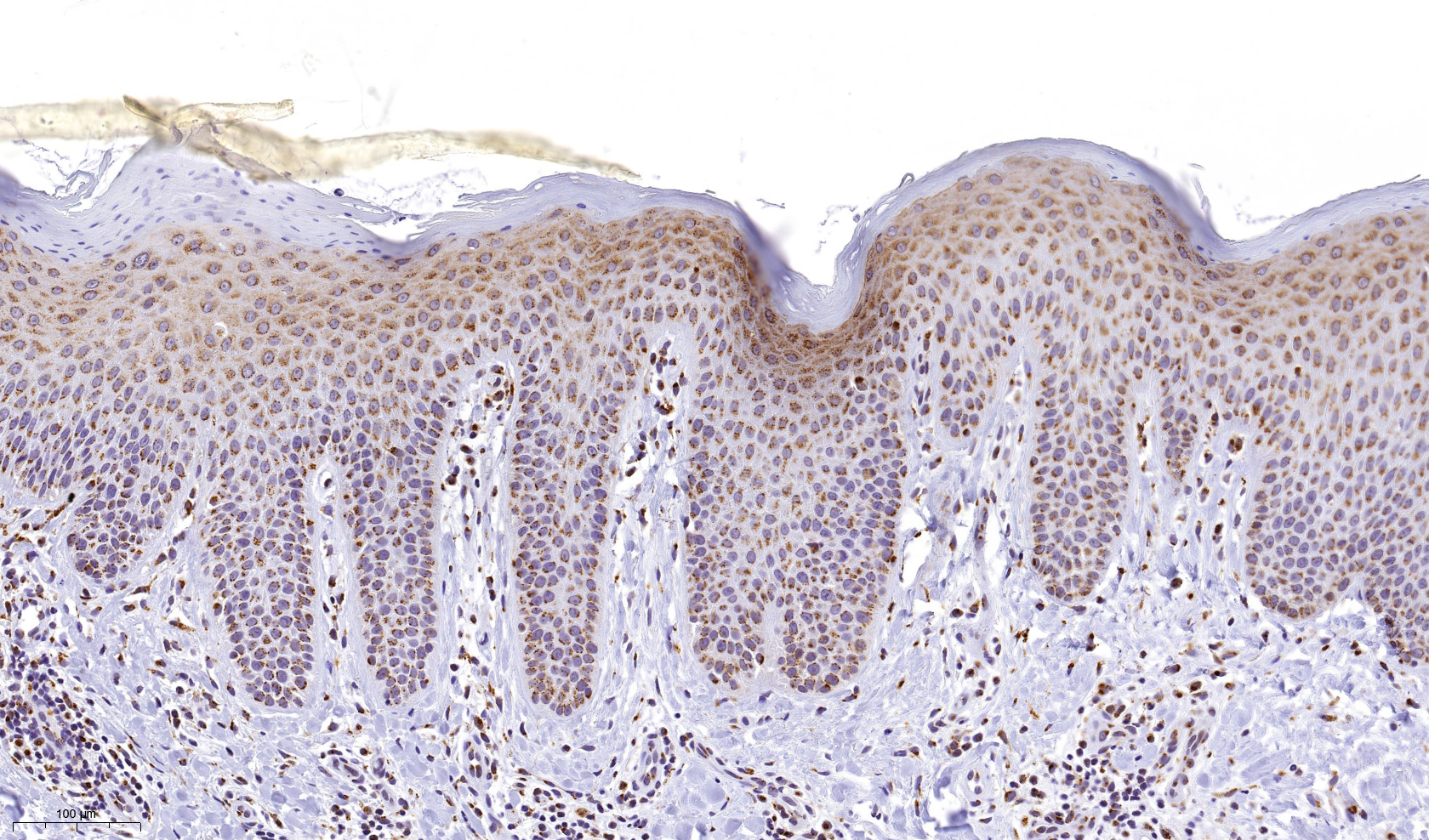

Supplement: Supplementary file 3 — Source Data for Appendix [file EMMM-15-e16758-s005.zip › Figure S8/AD 956_15_15.0x 20230221.tif]

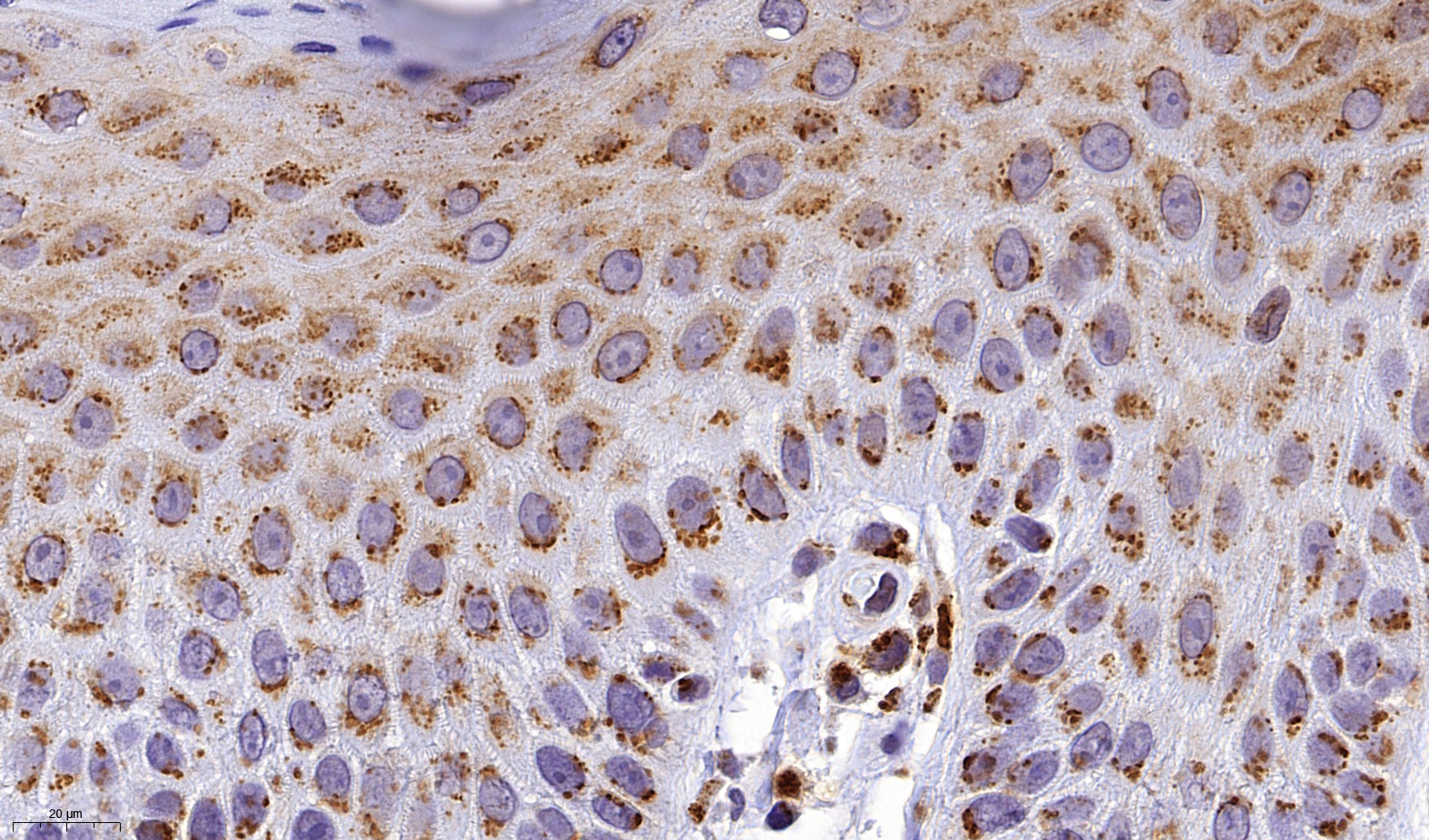

Supplement: Supplementary file 3 — Source Data for Appendix [file EMMM-15-e16758-s005.zip › Figure S8/AD 956_15_63.0x.tif]

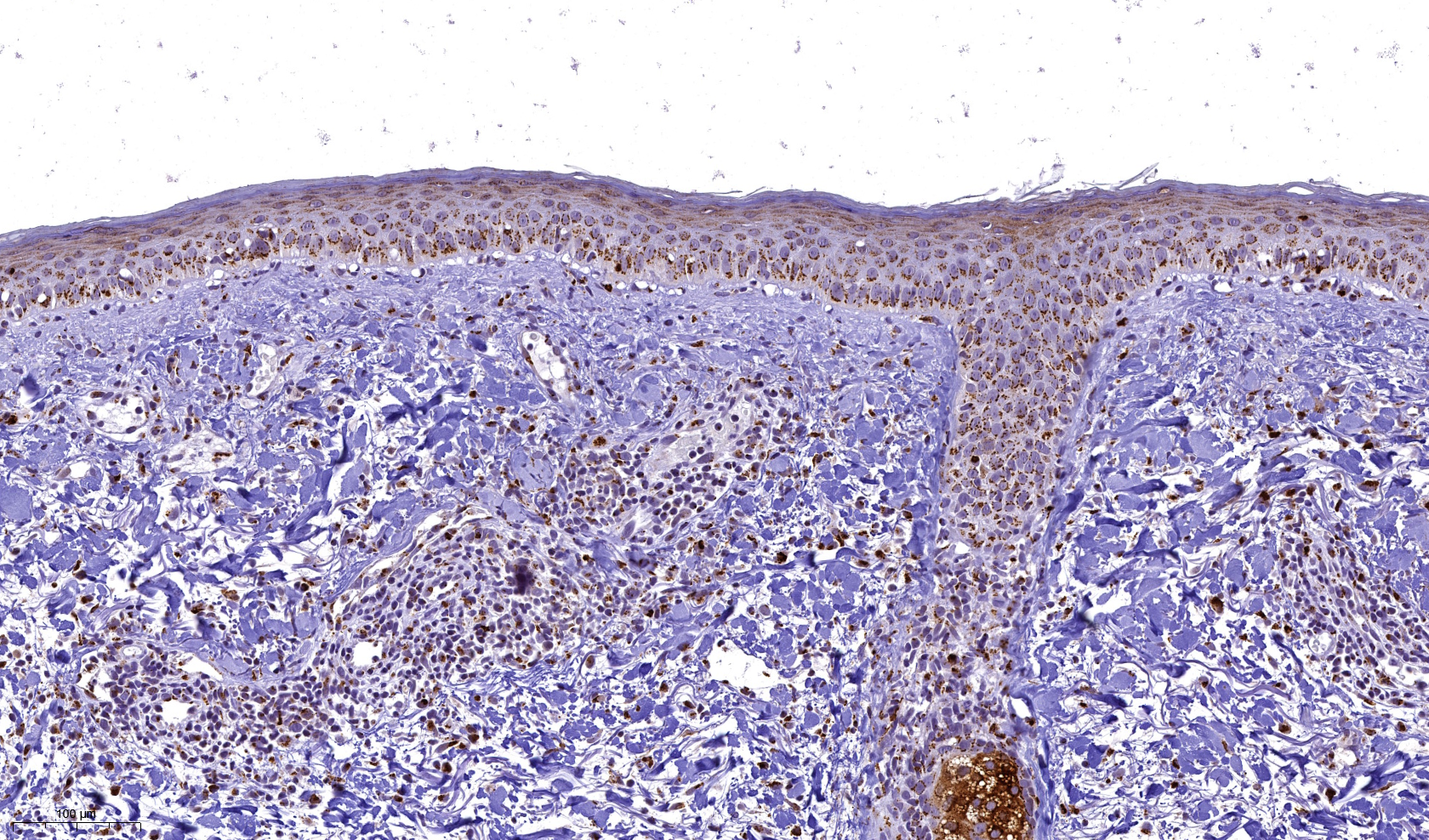

Supplement: Supplementary file 3 — Source Data for Appendix [file EMMM-15-e16758-s005.zip › Figure S8/Lupus 2809_12_15.0x 20230221.tif]

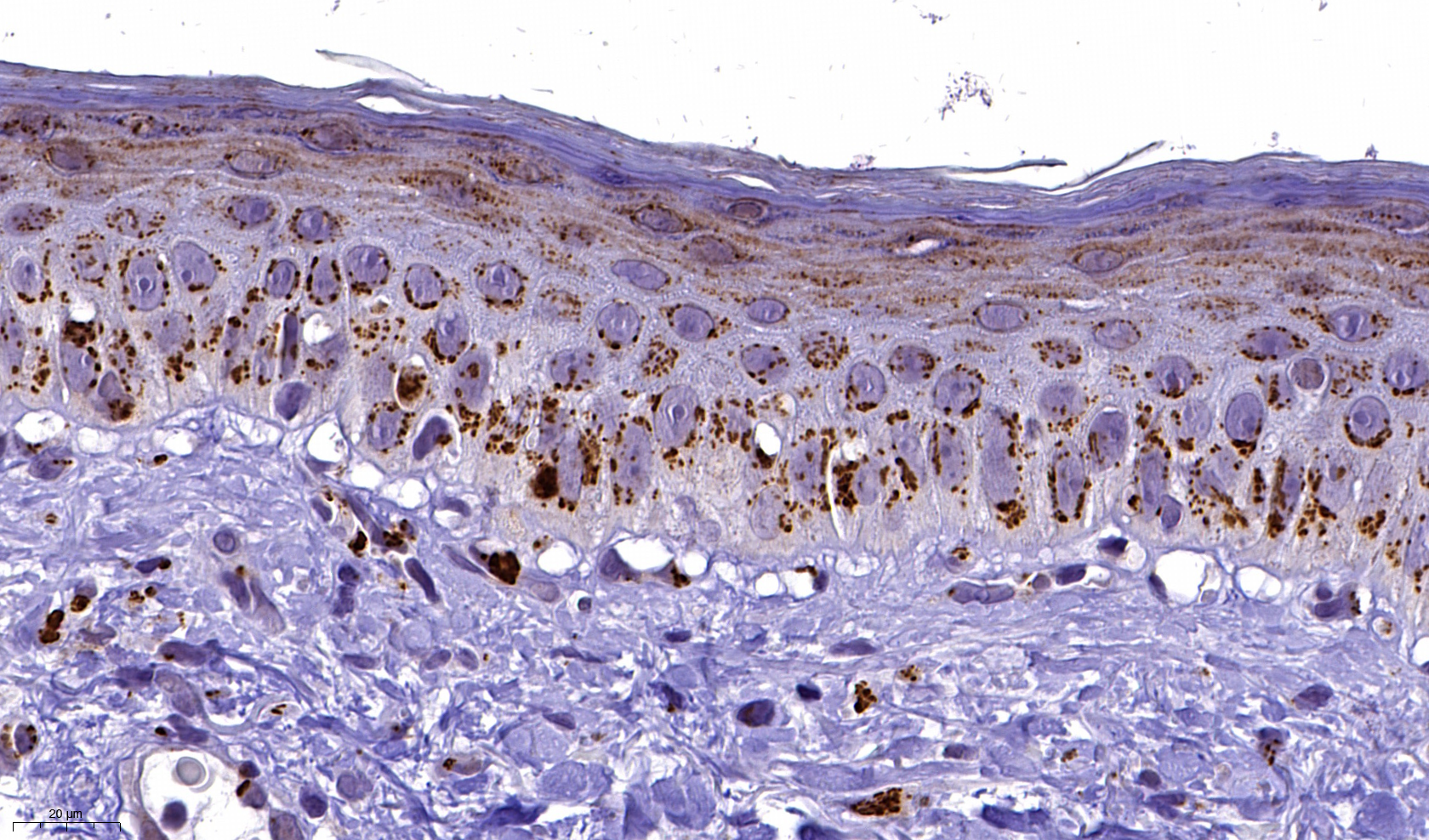

Supplement: Supplementary file 3 — Source Data for Appendix [file EMMM-15-e16758-s005.zip › Figure S8/Lupus 2809_12_63.0x.tif]

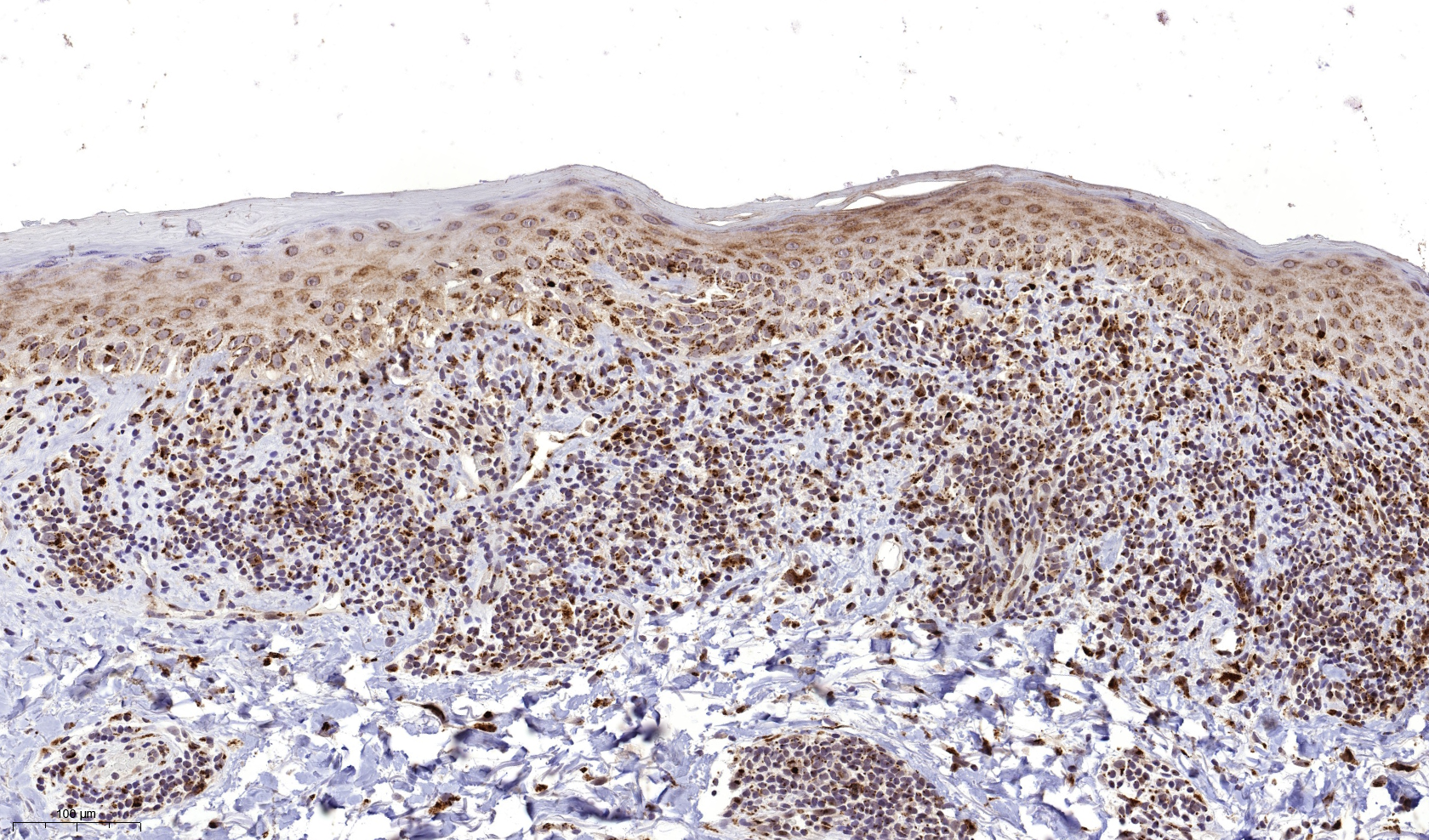

Supplement: Supplementary file 3 — Source Data for Appendix [file EMMM-15-e16758-s005.zip › Figure S8/Lupus 3392_12_15.0x 20230221.tif]

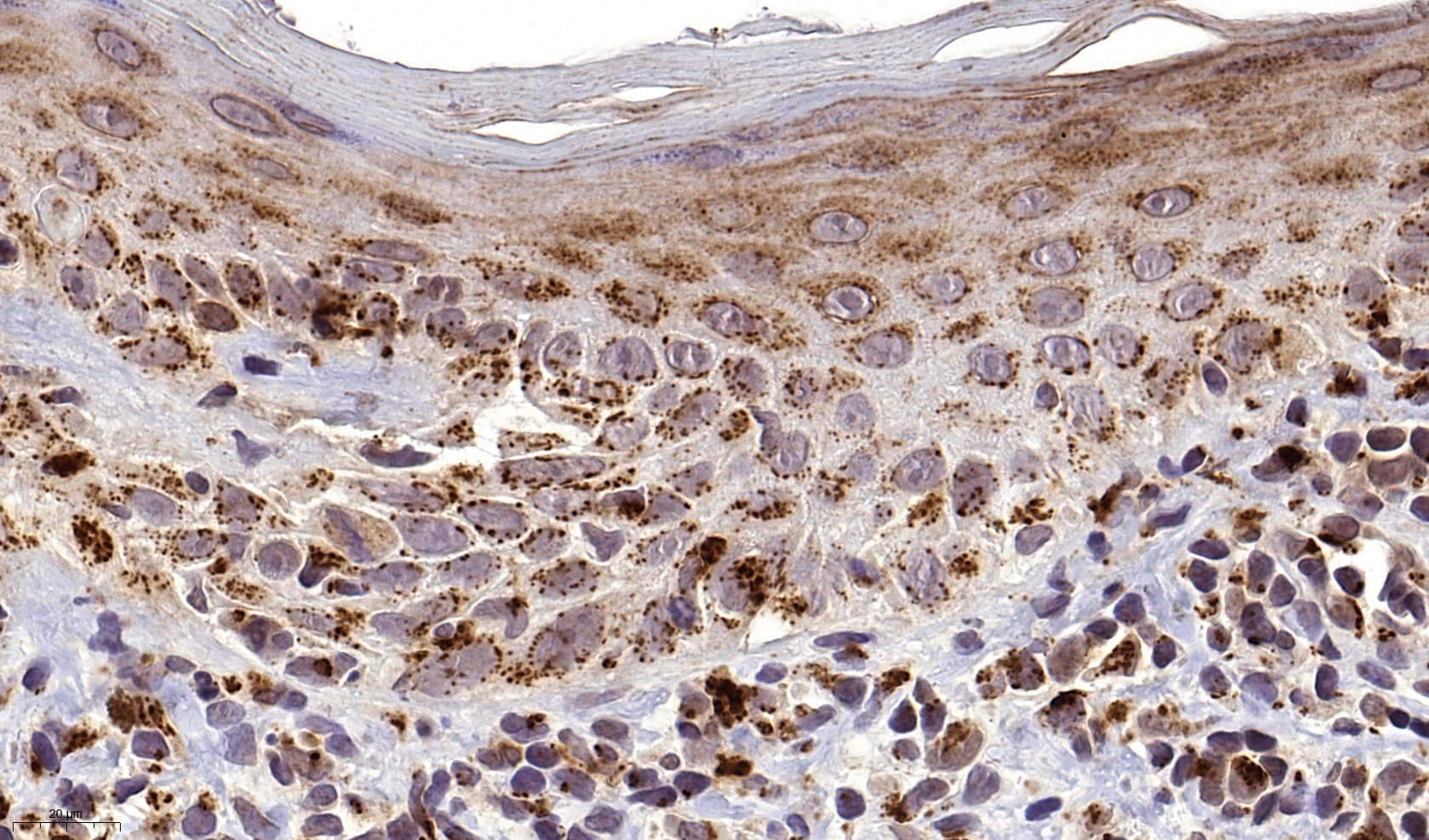

Supplement: Supplementary file 3 — Source Data for Appendix [file EMMM-15-e16758-s005.zip › Figure S8/Lupus 3392_12_63.0x.tif]

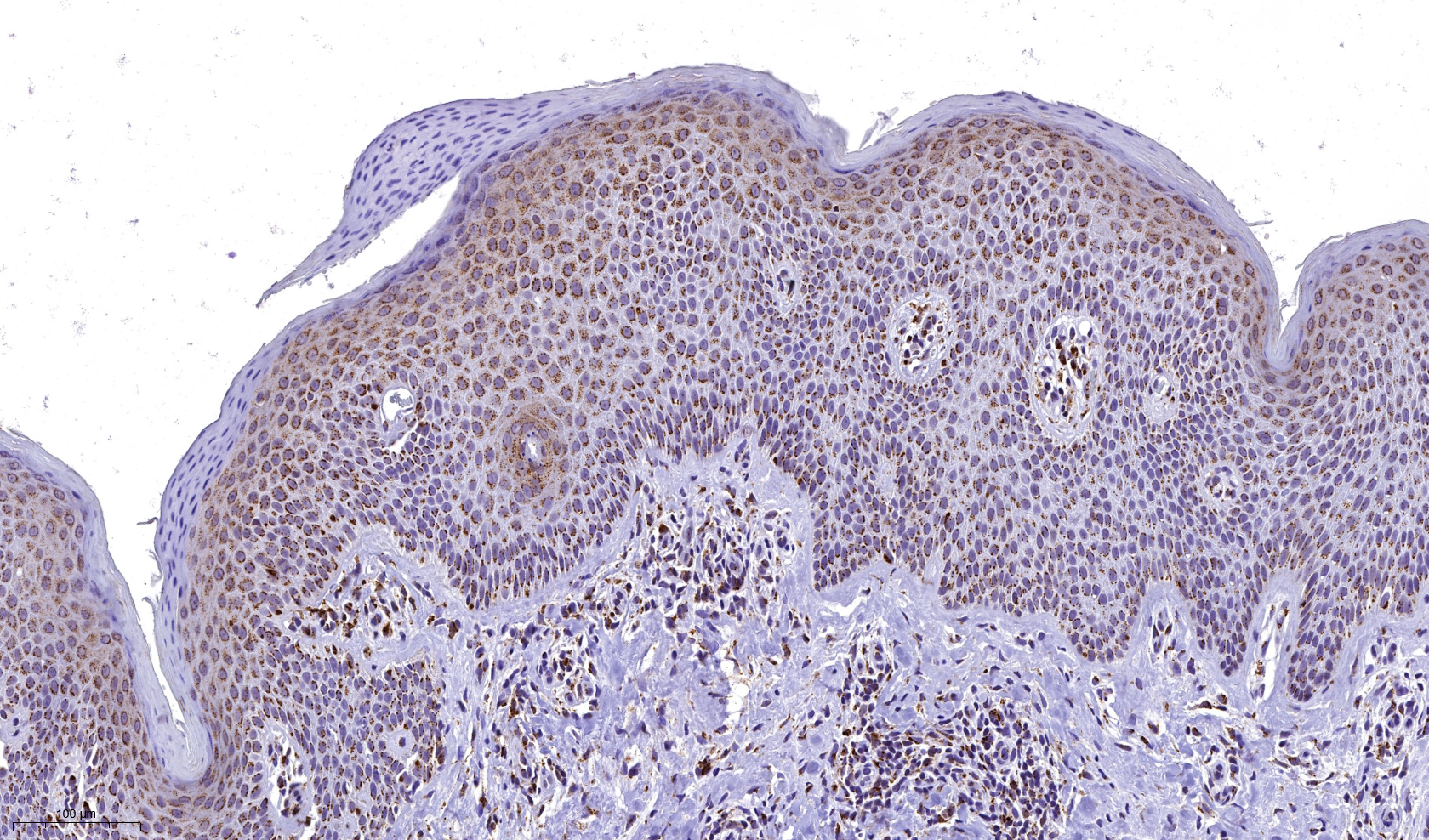

Supplement: Supplementary file 3 — Source Data for Appendix [file EMMM-15-e16758-s005.zip › Figure S8/Mykosis 2798_11_15.0x 20230220.tif]

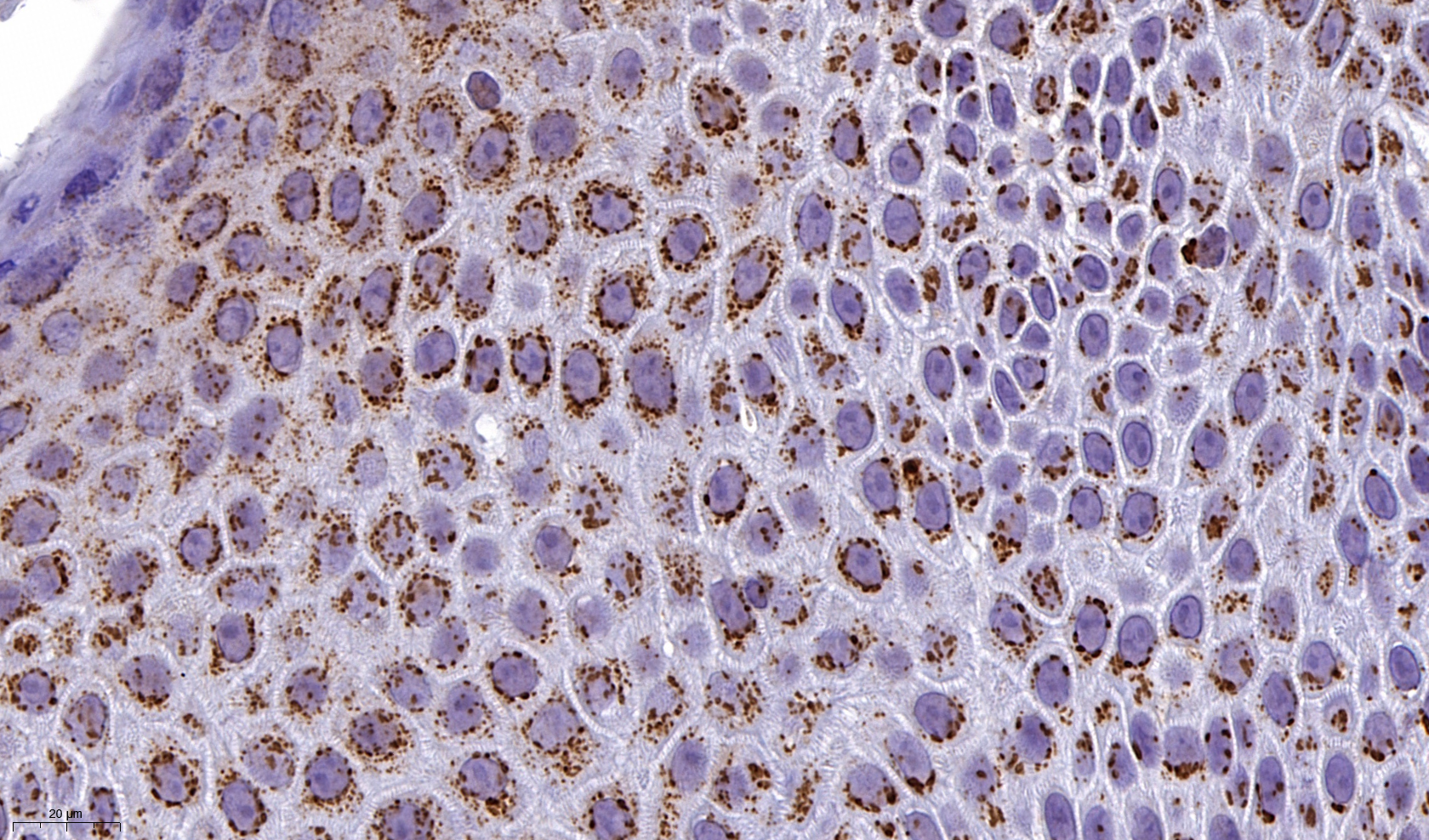

Supplement: Supplementary file 3 — Source Data for Appendix [file EMMM-15-e16758-s005.zip › Figure S8/Mykosis 2798_11_63.0x.tif]

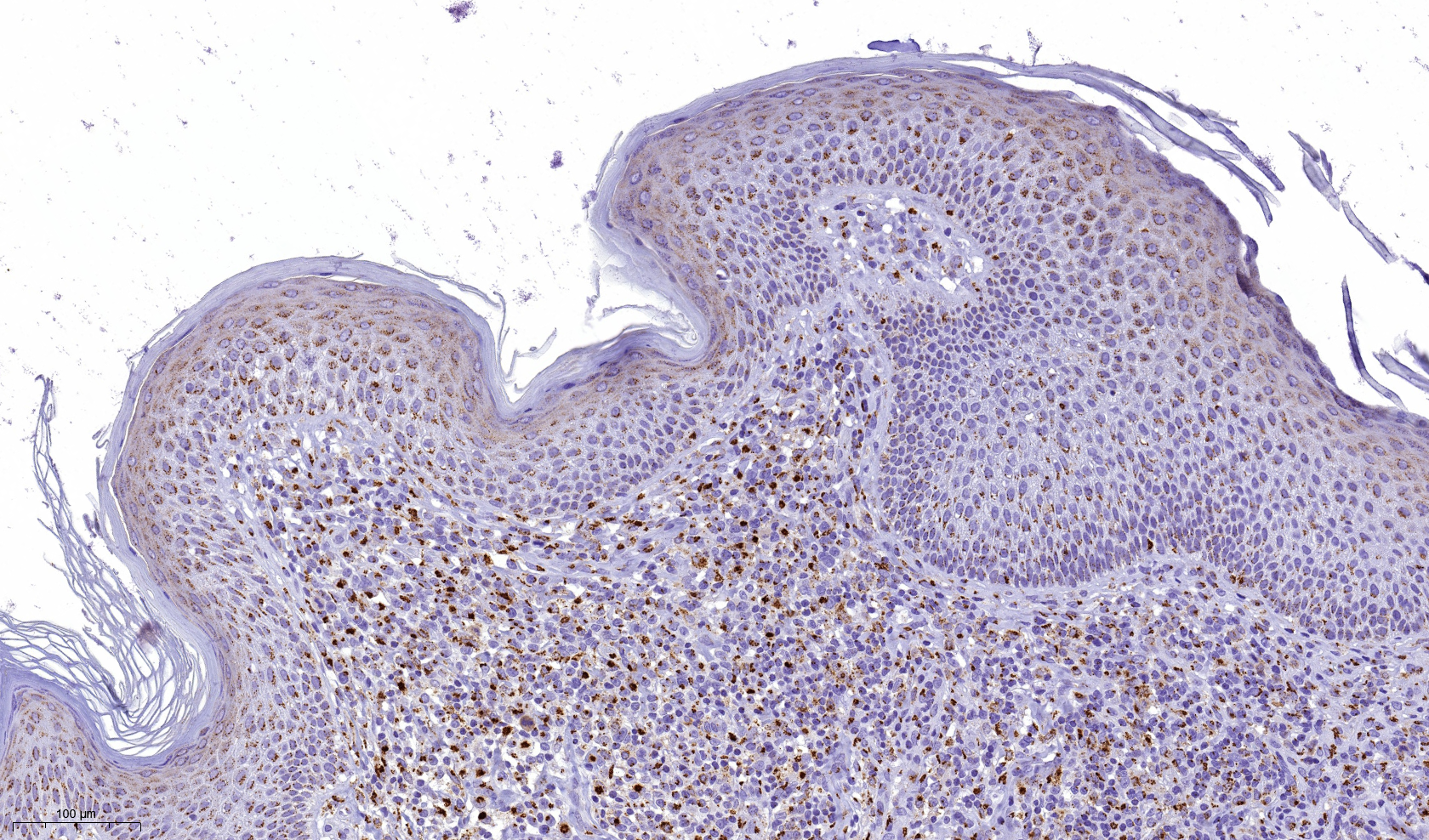

Supplement: Supplementary file 3 — Source Data for Appendix [file EMMM-15-e16758-s005.zip › Figure S8/Mykosis 3009_12_15.0x 20230220.tif]

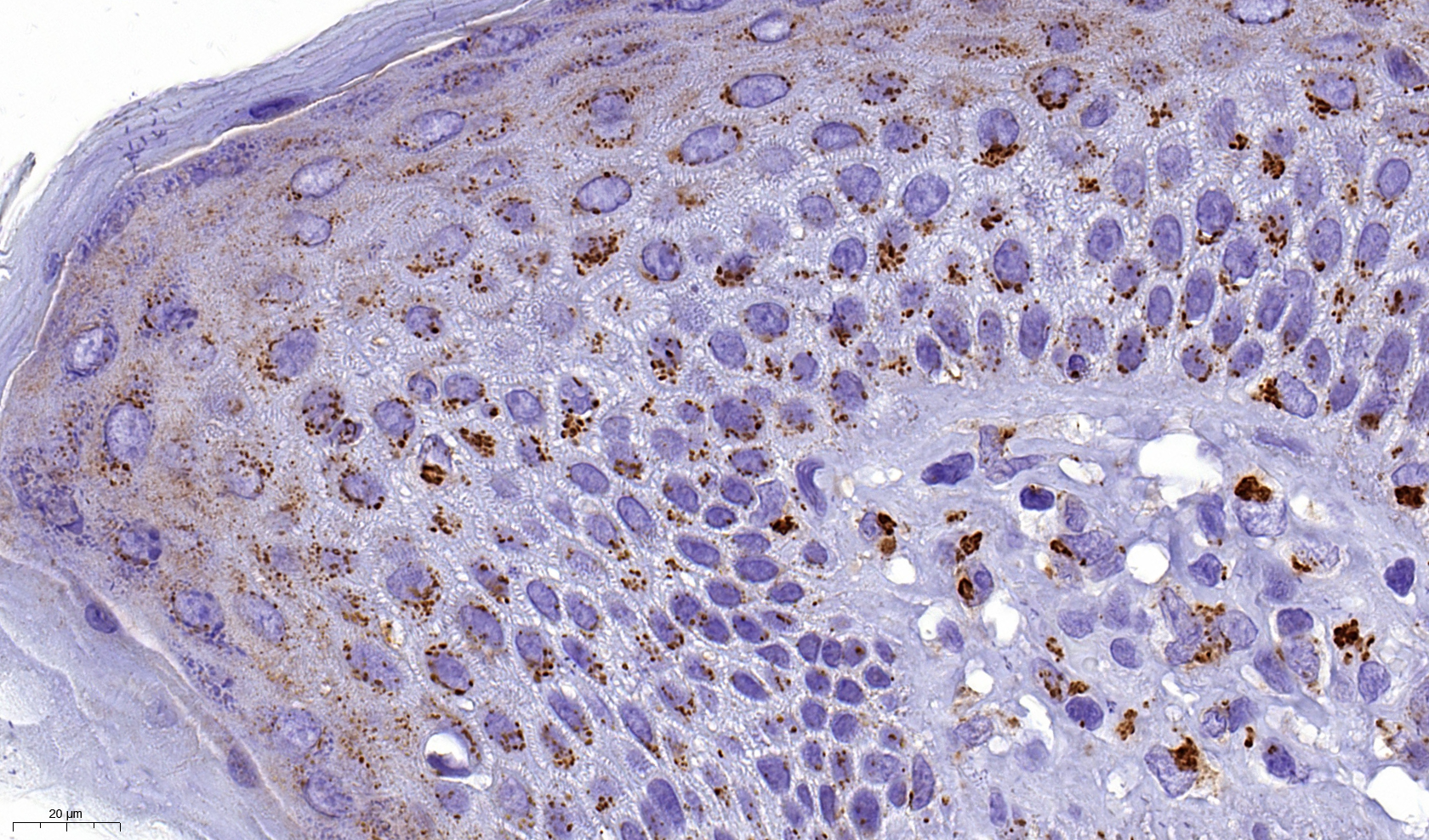

Supplement: Supplementary file 3 — Source Data for Appendix [file EMMM-15-e16758-s005.zip › Figure S8/Mykosis 3009_12_63.0x.tif]

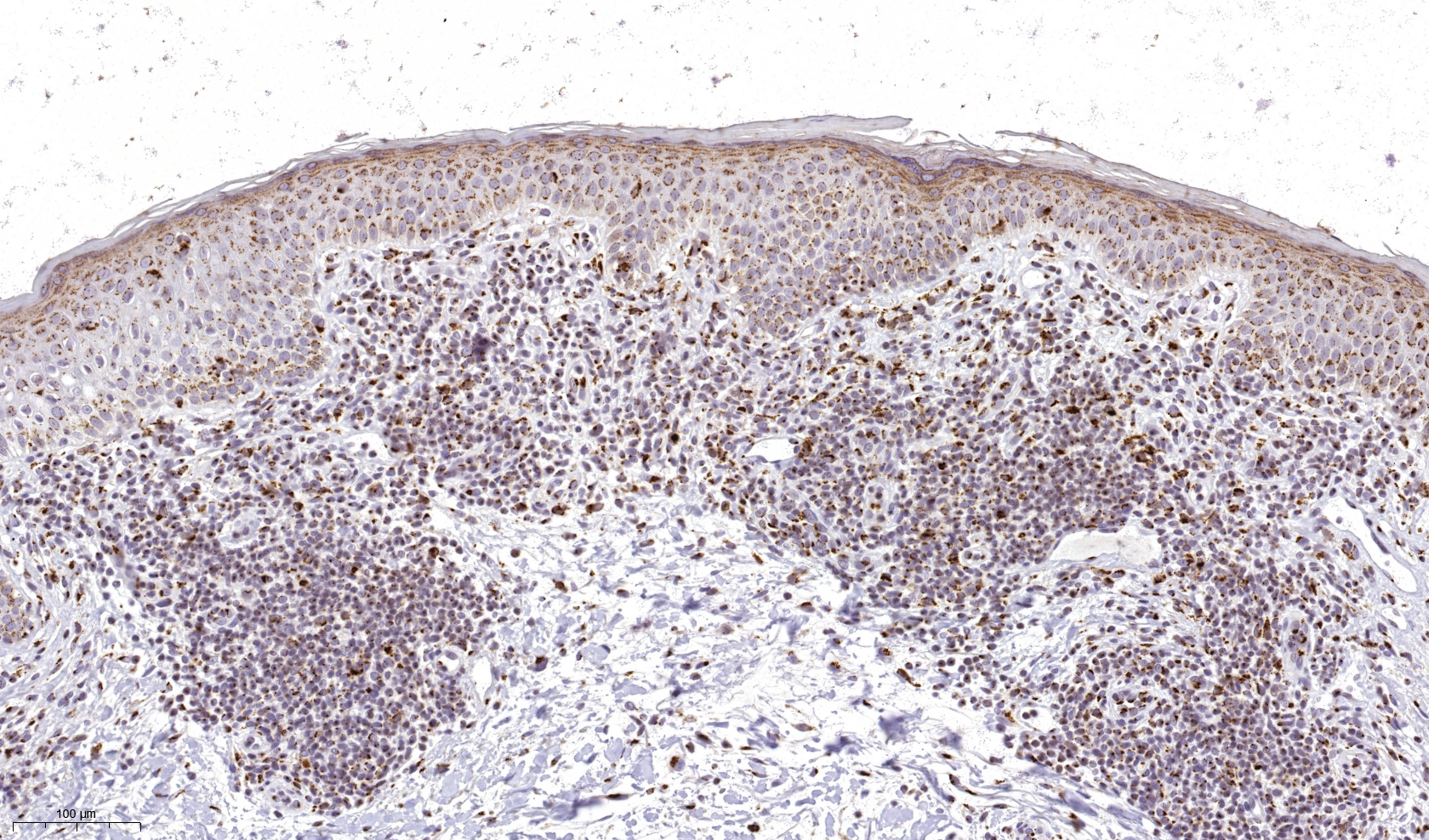

Supplement: Supplementary file 3 — Source Data for Appendix [file EMMM-15-e16758-s005.zip › Figure S8/Mykosis 3461_19_15.0x 20230220.tif]

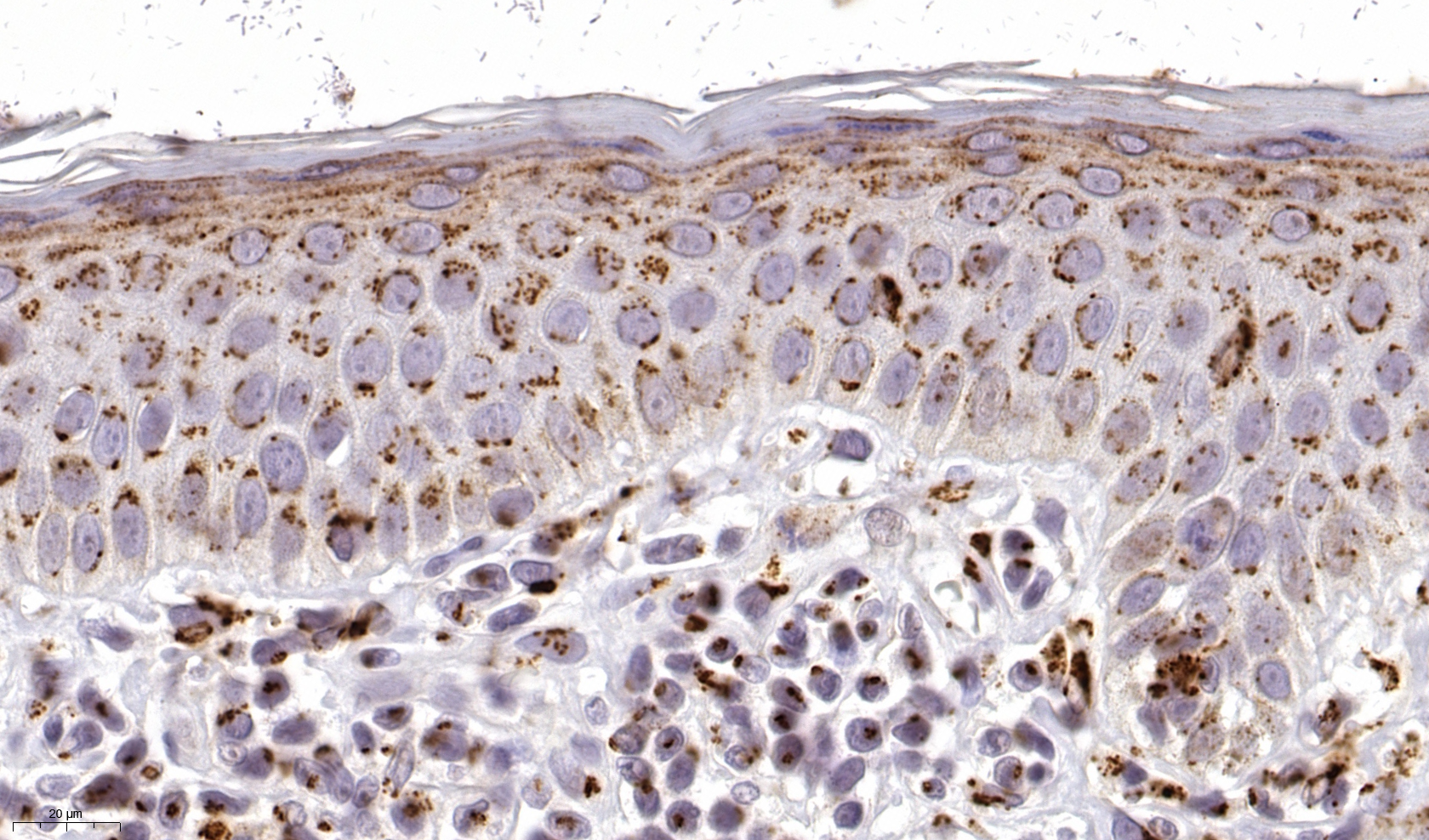

Supplement: Supplementary file 3 — Source Data for Appendix [file EMMM-15-e16758-s005.zip › Figure S8/Mykosis 3461_19_63.0x.tif]

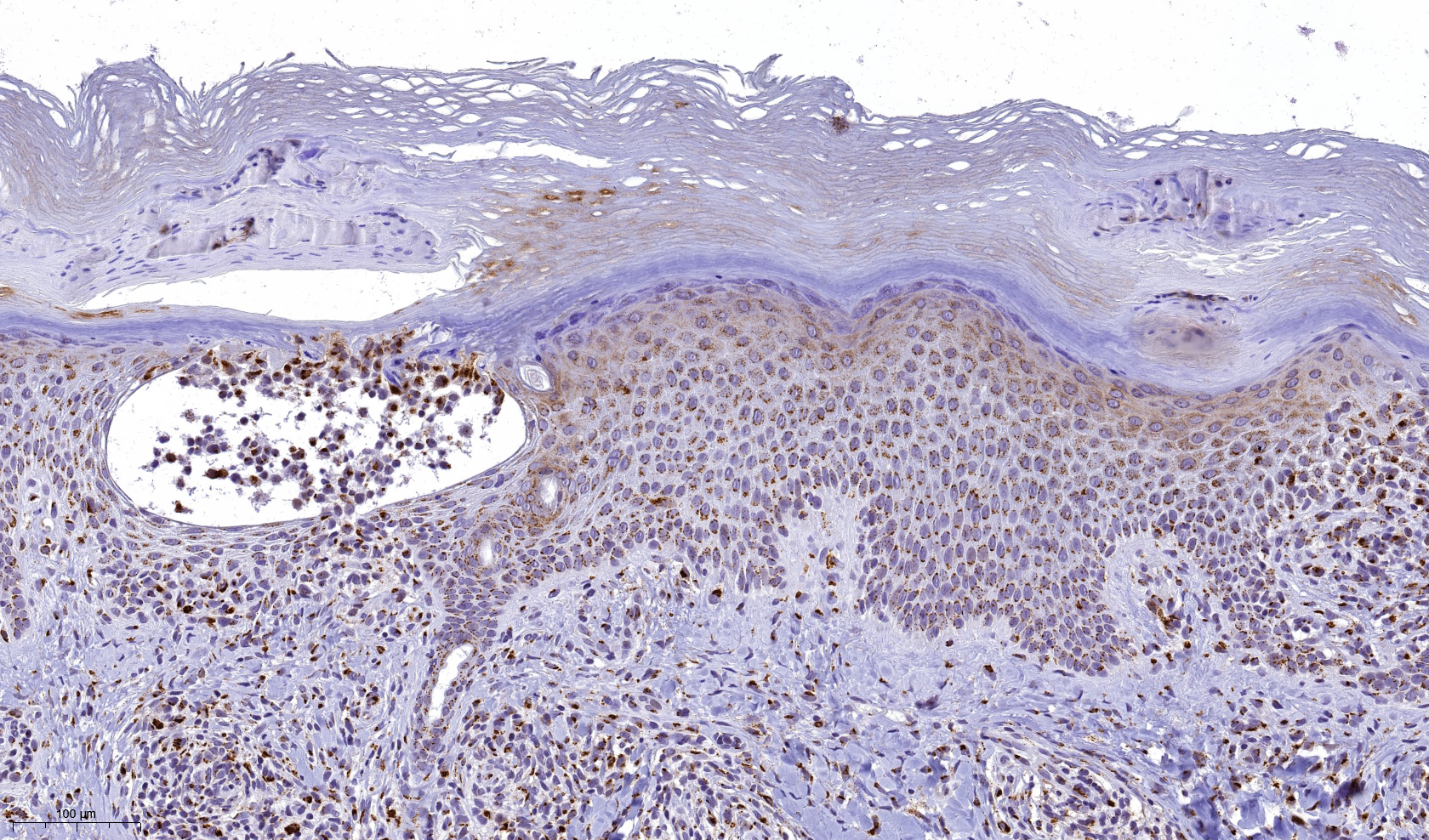

Supplement: Supplementary file 3 — Source Data for Appendix [file EMMM-15-e16758-s005.zip › Figure S8/Mykosis 609_12_15.0x 20230221.tif]

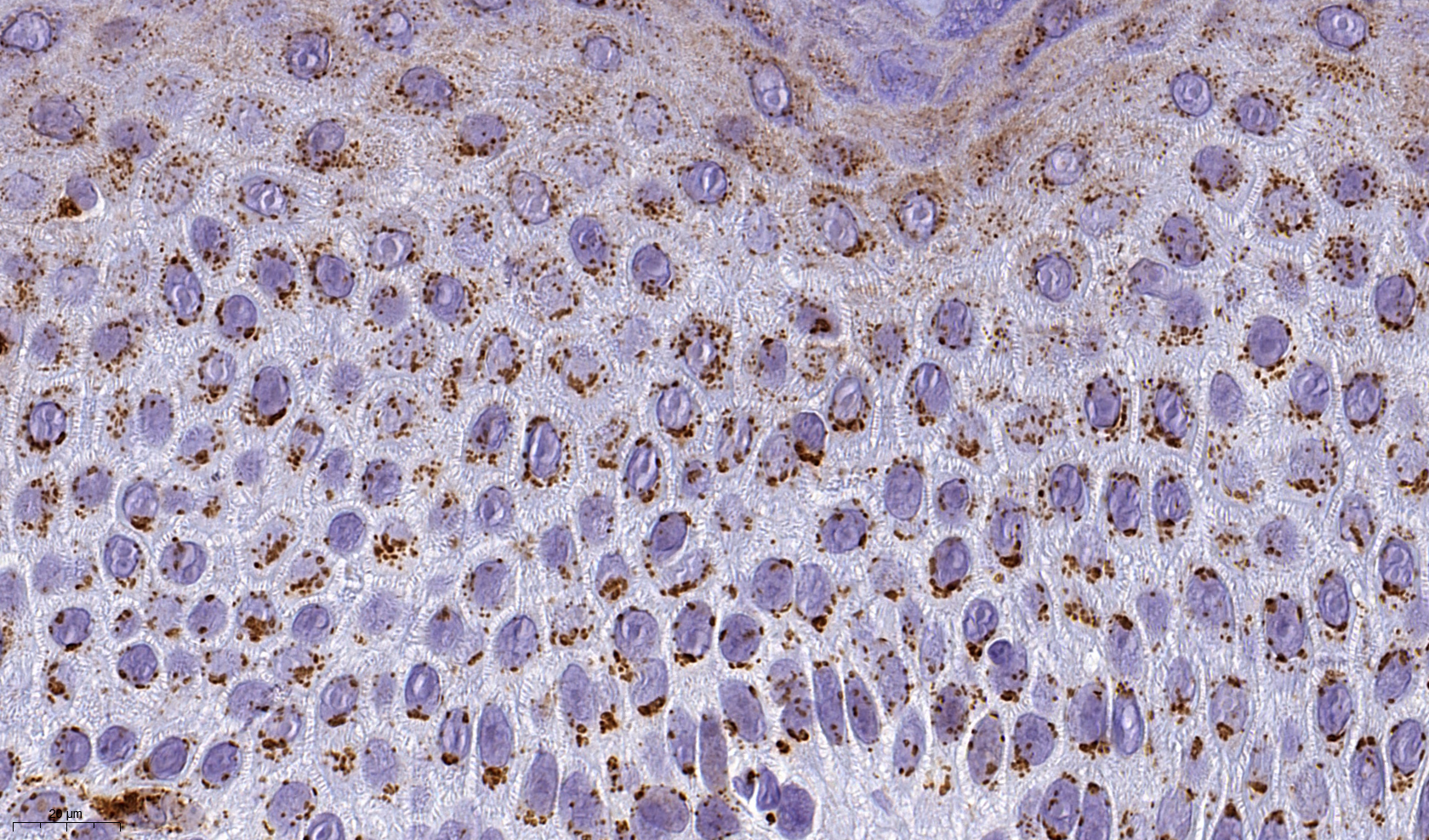

Supplement: Supplementary file 3 — Source Data for Appendix [file EMMM-15-e16758-s005.zip › Figure S8/Mykosis 609_12_63.0x.tif]

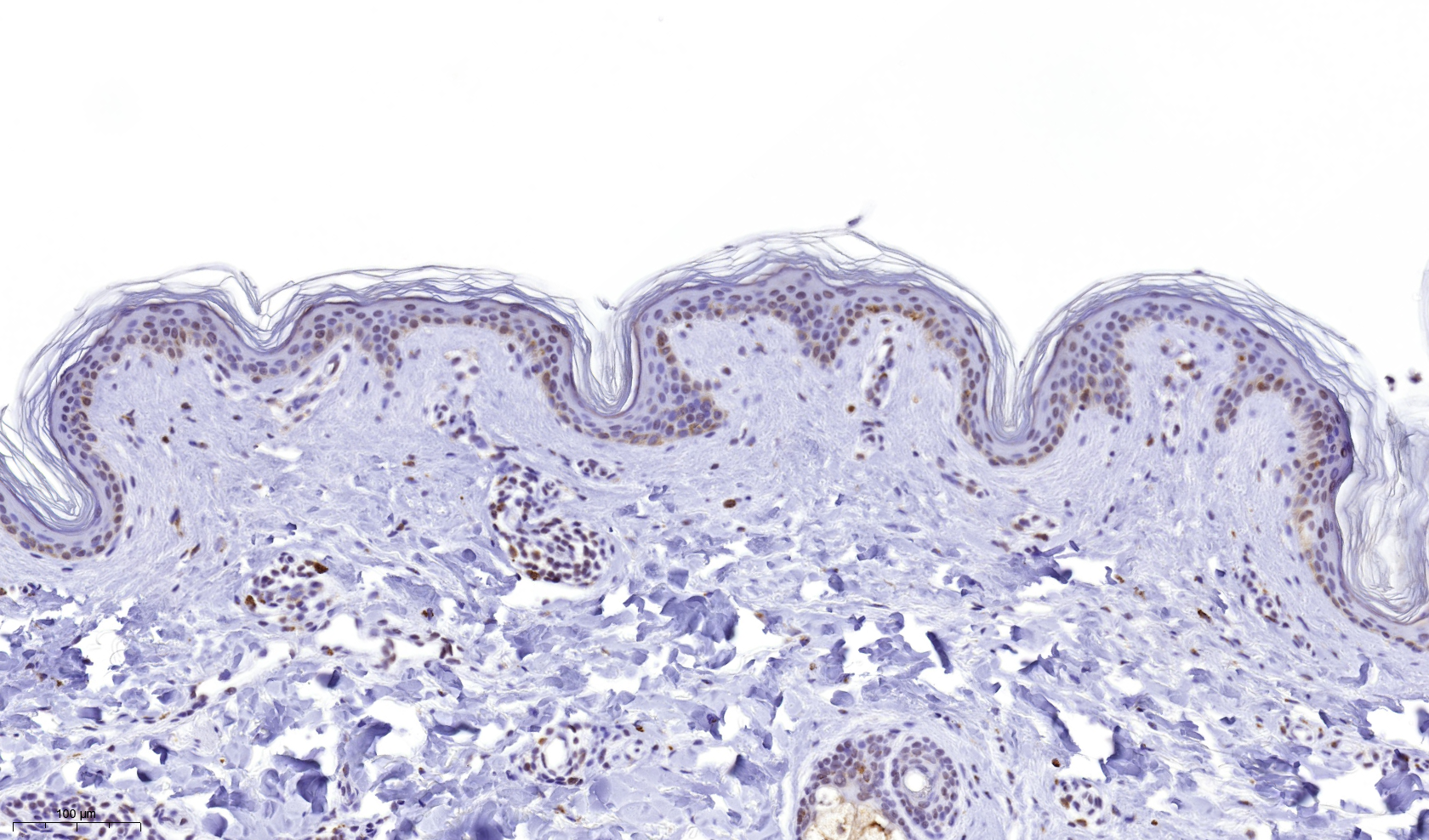

Supplement: Supplementary file 3 — Source Data for Appendix [file EMMM-15-e16758-s005.zip › Figure S8/norm BML960 u_arm_15.0x 20230221.tif]

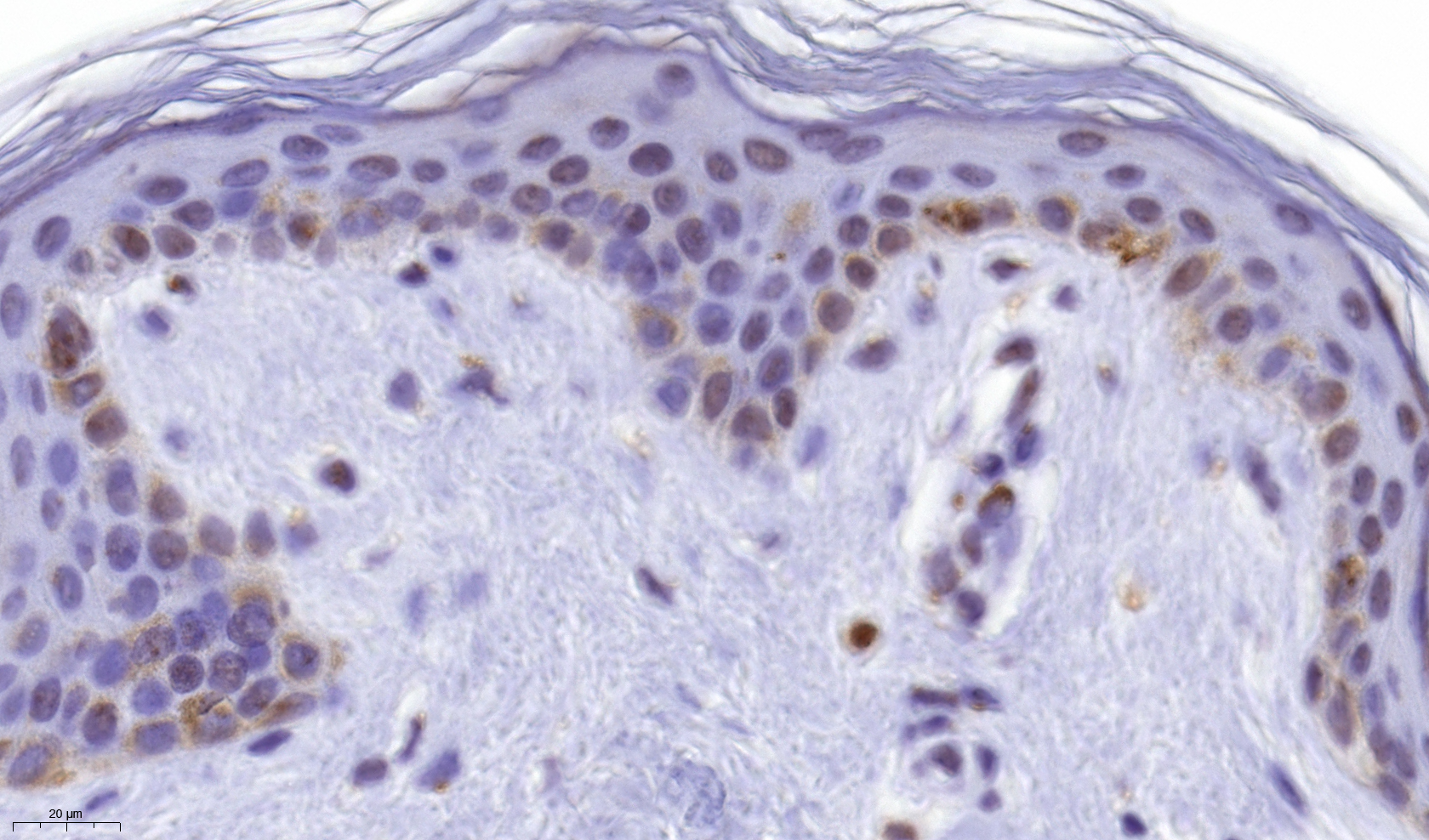

Supplement: Supplementary file 3 — Source Data for Appendix [file EMMM-15-e16758-s005.zip › Figure S8/norm BML960 u_arm_63.0x.tif]

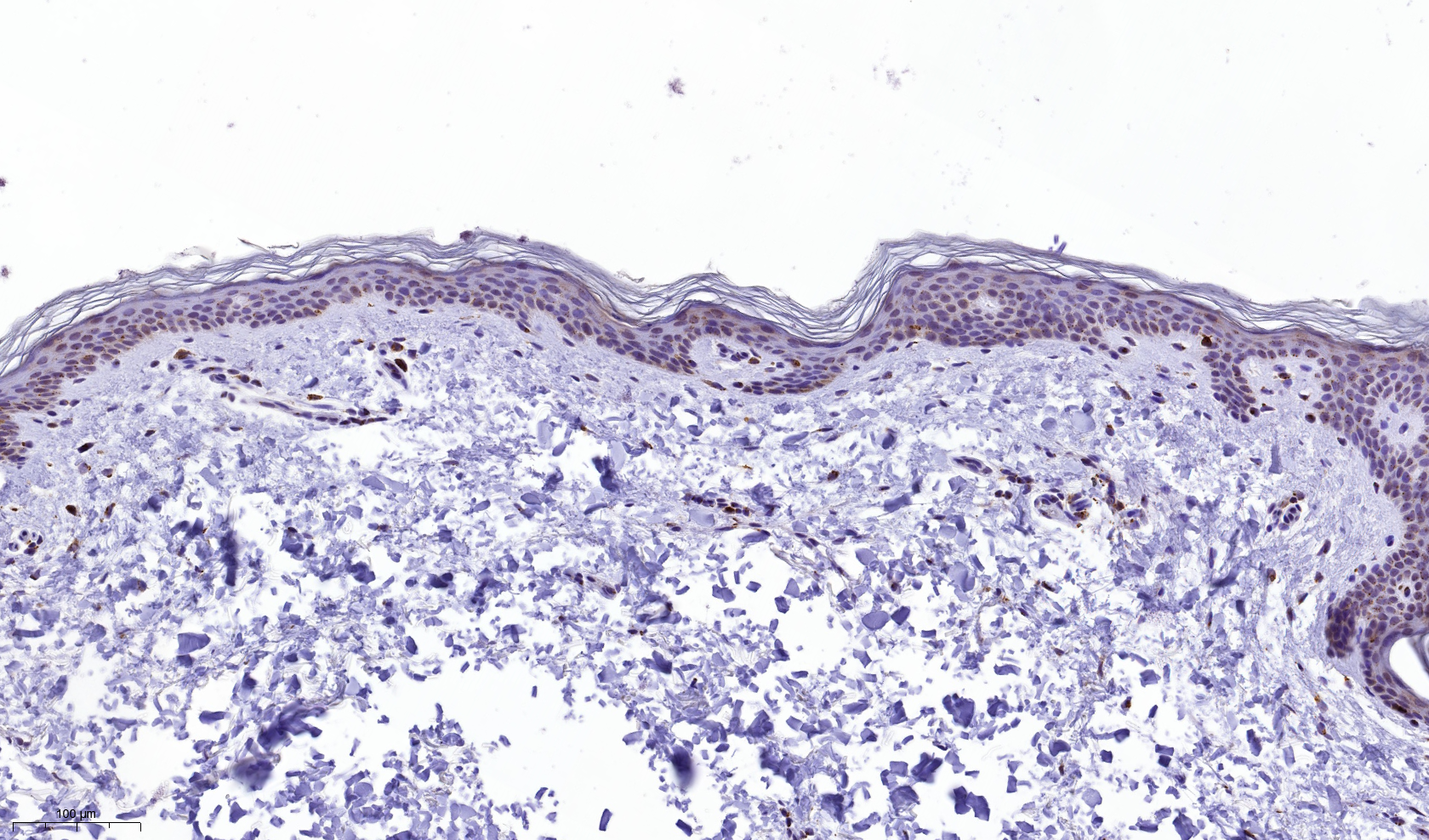

Supplement: Supplementary file 3 — Source Data for Appendix [file EMMM-15-e16758-s005.zip › Figure S8/norm_Haut_15.0x 20230221.tif]

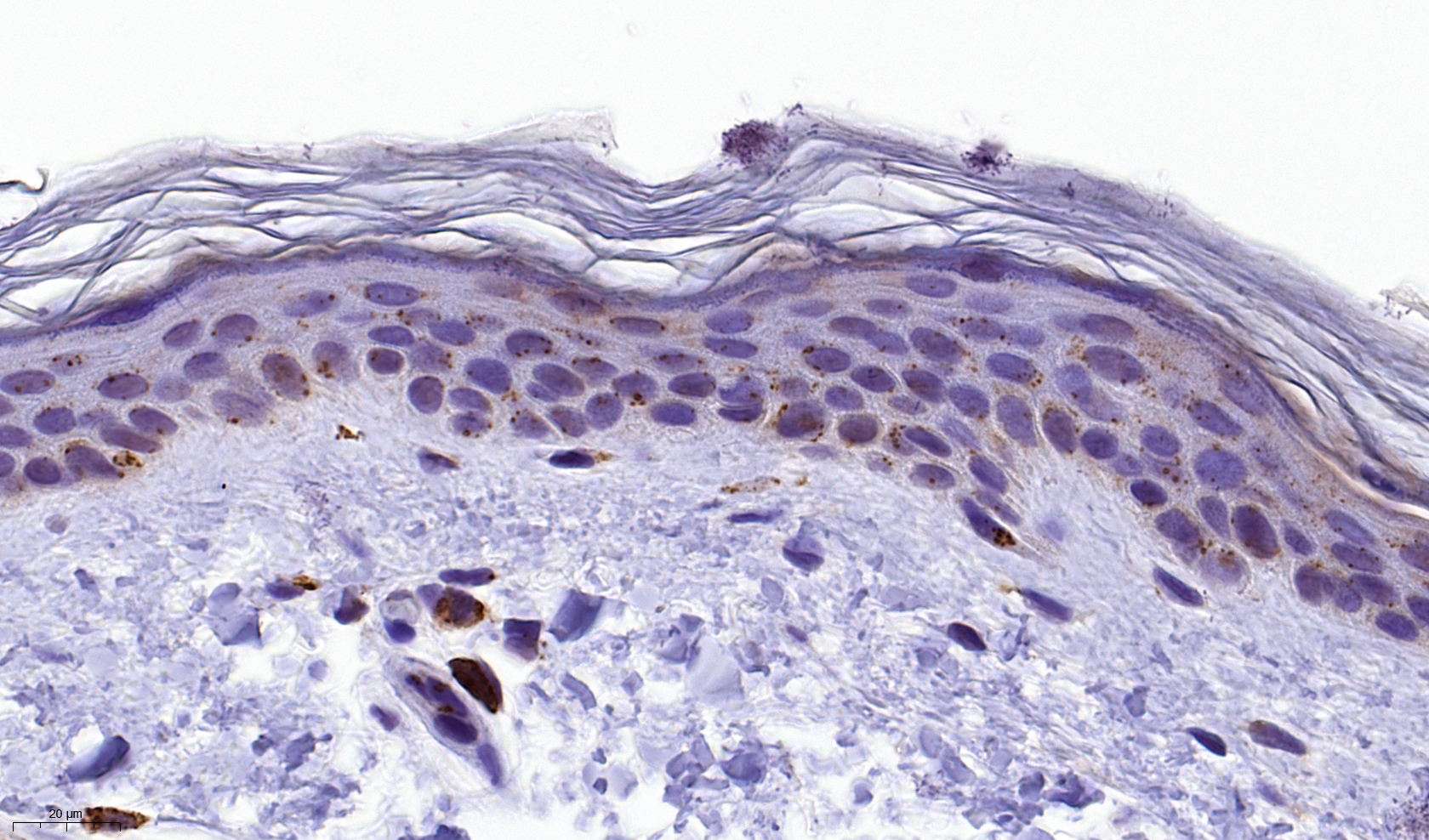

Supplement: Supplementary file 3 — Source Data for Appendix [file EMMM-15-e16758-s005.zip › Figure S8/norm_Haut_ILEI_63.0x.tif]

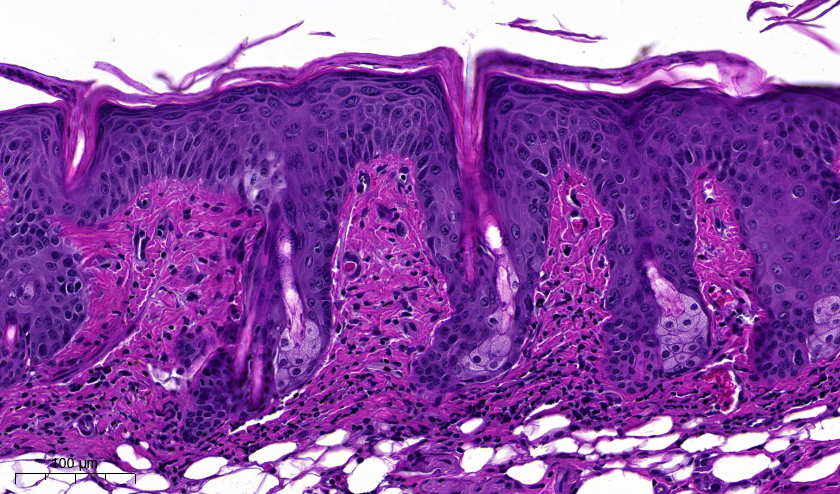

Supplement: Supplementary file 11 — Source Data for Figure 7 [file EMMM-15-e16758-s004.zip › Figure 7/7I/KTG-179_12.0x OE_TPA_DMSO.tiff]

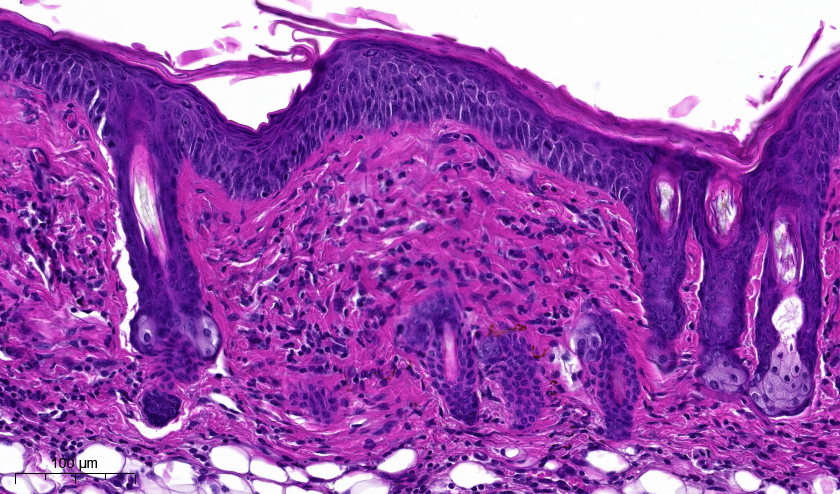

Supplement: Supplementary file 11 — Source Data for Figure 7 [file EMMM-15-e16758-s004.zip › Figure 7/7I/KTG_229_HE_12.0x ctrl_TPA_DMSO.tiff]

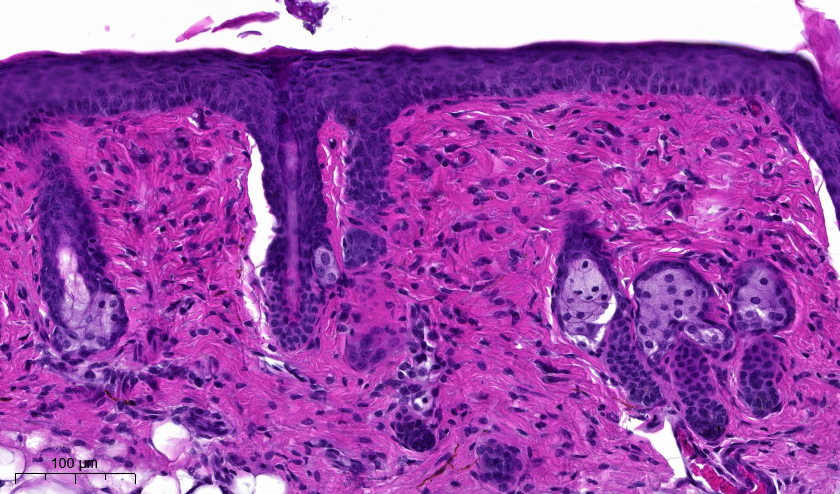

Supplement: Supplementary file 11 — Source Data for Figure 7 [file EMMM-15-e16758-s004.zip › Figure 7/7I/KTG_238_HE_12.0x ctrl_TPA_UK.tiff]

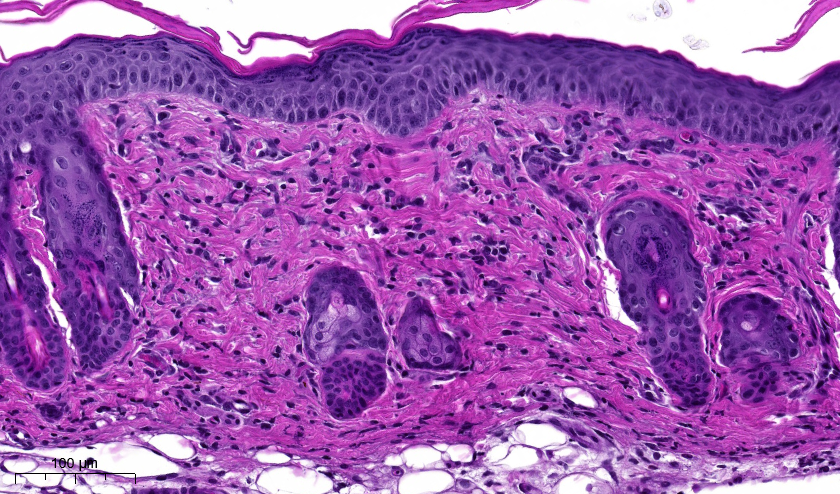

Supplement: Supplementary file 11 — Source Data for Figure 7 [file EMMM-15-e16758-s004.zip › Figure 7/7I/KTG_240_HE_12.0x OE_TPA_UK.tiff]

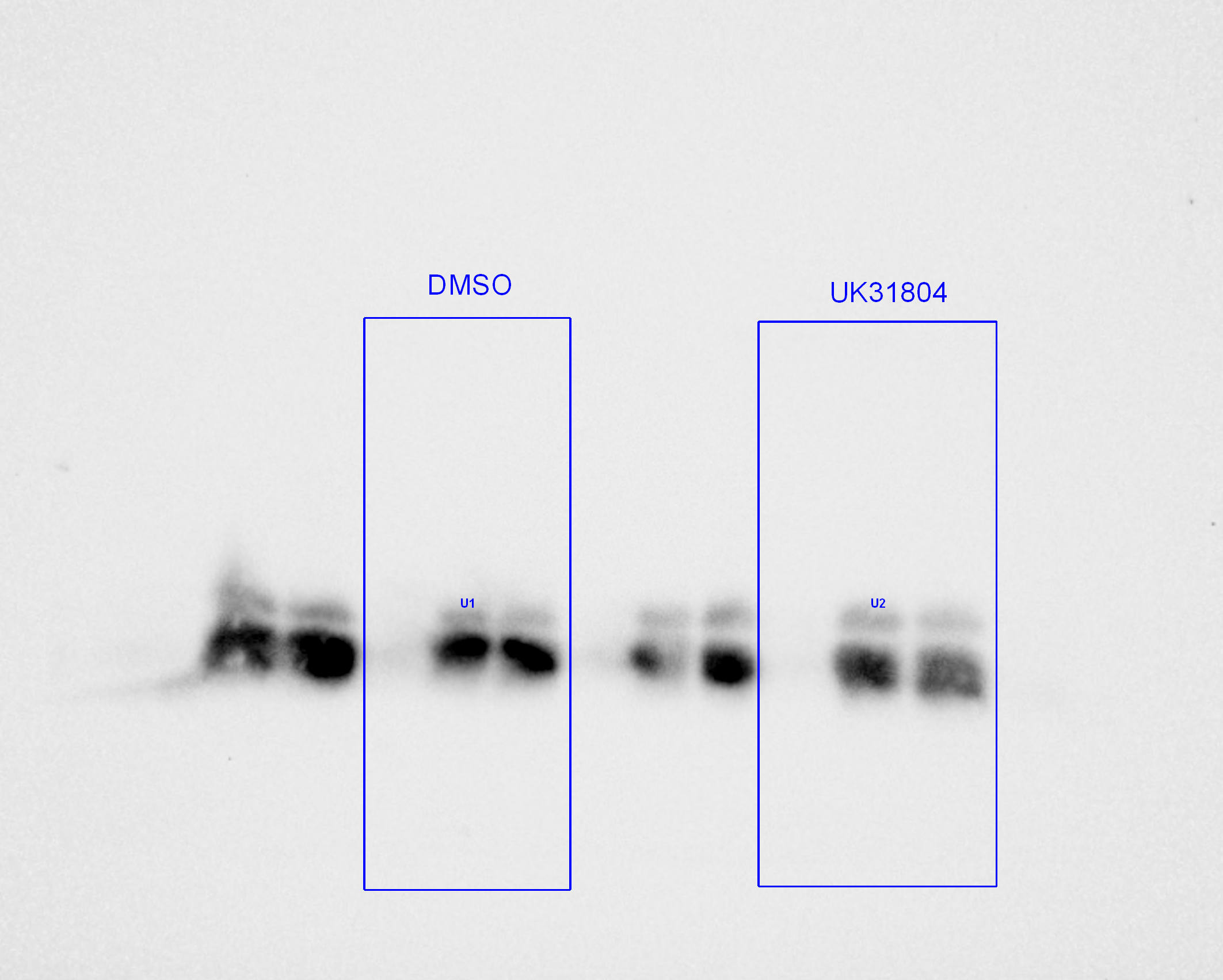

Supplement: Supplementary file 11 — Source Data for Figure 7 [file EMMM-15-e16758-s004.zip › Figure 7/7L/uncropped_ILEI CM DMSO+UK.tif]
