# Supplementary material for: FAM3C/ILEI protein is elevated in psoriatic lesions and triggers psoriasiform hyperproliferation in mice
Source: EMBO Mol Med. 2023 May 25;15(7):e16758. doi: 10.15252/emmm.202216758 (PMC10331587; doi:10.15252/emmm.202216758)
Supplement: Supplementary file 12 [file EMMM-15-e16758-s011.pdf]

EMBO Molecular Medicine

FAM3C/ILEI protein is elevated in psoriatic lesions and triggers psoriasiform hyperproliferation in mice

Barizah Malik, Iva Vokic, Thomas Mohr, Marle Poppelaars, Martin Holcmann, Philipp Novoszel, Gerald Timelthaler, Thomas Lendl, Dana Krauss, Ulrich Elling, Michael Mildner, Josef Penninger, Peter Petzelbauer, Maria Sibilia, and Agnes Csiszar
DOI: 10.15252/emmm.202216758

Corresponding author(s): Agnes Csiszar (agnes.csiszar@meduniwien.ac.at)

Review Timeline:

Submission Date:	17th Aug 22
Editorial Decision:	13th Sep 22
Revision Received:	28th Mar 23
Editorial Decision:	11th Apr 23
Revision Received:	6th May 23
Accepted:	8th May 23

Editor: Zeljko Durdevic

Transaction Report:

(Note: With the exception of the correction of typographical or spelling errors that could be a source of ambiguity, letters and reports are not edited. Depending on transfer agreements, referee reports obtained elsewhere may or may not be included in this compilation. Referee reports are anonymous unless the Referee chooses to sign their reports.)

13th Sep 2022

Dear Dr. Csiszar,

Thank you for the submission of your manuscript to EMBO Molecular Medicine. We have now received feedback from the two reviewers who agreed to evaluate your manuscript. While the referee #2 is overall supportive of the study and raises minor critique, referee #1 recognizes potential interest of the study but also raises serious concerns that should be addressed in a major revision.

Further consideration of a revision that addresses reviewers' concerns in full will entail a second round of review. EMBO Molecular Medicine encourages a single round of revision only and therefore, acceptance or rejection of the manuscript will depend on the completeness of your responses included in the next, final version of the manuscript. For this reason, and to save you from any frustrations in the end, I would strongly advise against returning an incomplete revision.

We would welcome the submission of a revised version within three months for further consideration. Please let us know if you require longer to complete the revision.

I look forward to receiving your revised manuscript.

Yours sincerely,

Zeljko Durdevic

Zeljko Durdevic
Editor
EMBO Molecular Medicine

*****

When submitting your revised manuscript, please carefully review the instructions that follow below. We perform an initial quality control of all revised manuscripts before re-review; failure to include requested items will delay the evaluation of your revision.

We require:

1) A .docx formatted version of the manuscript text (including legends for main figures, EV figures and tables). Please make sure that the changes are highlighted to be clearly visible.

2) Individual production quality figure files as .eps, .tif, .jpg (one file per figure). For guidance, download the 'Figure Guide PDF': (https://www.embopress.org/page/journal/17574684/authorguide#figureformat).

3) A .docx formatted letter INCLUDING the reviewers' reports and your detailed point-by-point responses to their comments. As part of the EMBO Press transparent editorial process, the point-by-point response is part of the Review Process File (RPF), which will be published alongside your paper.

4) A complete author checklist, which you can download from our author guidelines (https://www.embopress.org/page/journal/17574684/authorguide#submissionofrevisions). Please insert information in the checklist that is also reflected in the manuscript. The completed author checklist will also be part of the RPF.

5) Please note that all corresponding authors are required to supply an ORCID ID for their name upon submission of a revised manuscript.

6) It is mandatory to include a 'Data Availability' section after the Materials and Methods. Before submitting your revision, primary datasets produced in this study need to be deposited in an appropriate public database, and the accession numbers and

database listed under 'Data Availability'. Please remember to provide a reviewer password if the datasets are not yet public (see https://www.embopress.org/page/journal/17574684/authorguide#dataavailability).

In case you have no data that requires deposition in a public database, please state so in this section. Note that the Data Availability Section is restricted to new primary data that are part of this study.

7) For data quantification: please specify the name of the statistical test used to generate error bars and P values, the number (n) of independent experiments (specify technical or biological replicates) underlying each data point and the test used to calculate p-values in each figure legend. The figure legends should contain a basic description of n, P and the test applied. Graphs must include a description of the bars and the error bars (s.d., s.e.m.). See also 'Figure Legend' guidelines: https://www.embopress.org/page/journal/17574684/authorguide#figureformat

8) We would also encourage you to include the source data for figure panels that show essential data. Numerical data should be provided as individual .xls or .csv files (including a tab describing the data). For blots or microscopy, uncropped images should be submitted (using a zip archive if multiple images need to be supplied for one panel). Additional information on source data and instruction on how to label the files are available at

.

9) Our journal encourages inclusion of *data citations in the reference list* to directly cite datasets that were re-used and obtained from public databases. Data citations in the article text are distinct from normal bibliographical citations and should directly link to the database records from which the data can be accessed. In the main text, data citations are formatted as follows: "Data ref: Smith et al, 2001" or "Data ref: NCBI Sequence Read Archive PRJNA342805, 2017". In the Reference list, data citations must be labeled with "[DATASET]". A data reference must provide the database name, accession number/identifiers and a resolvable link to the landing page from which the data can be accessed at the end of the reference. Further instructions are available at .

10) We replaced Supplementary Information with Expanded View (EV) Figures and Tables that are collapsible/expandable online. A maximum of 5 EV Figures can be typeset. EV Figures should be cited as 'Figure EV1, Figure EV2" etc... in the text and their respective legends should be included in the main text after the legends of regular figures.

- For the figures that you do NOT wish to display as Expanded View figures, they should be bundled together with their legends in a single PDF file called *Appendix*, which should start with a short Table of Content. Appendix figures should be referred to in the main text as: "Appendix Figure S1, Appendix Figure S2" etc.

- Additional Tables/Datasets should be labeled and referred to as Table EV1, Dataset EV1, etc. Legends have to be provided in a separate tab in case of .xls files. Alternatively, the legend can be supplied as a separate text file (README) and zipped together with the Table/Dataset file.

See detailed instructions here:

.

11) The paper explained: EMBO Molecular Medicine articles are accompanied by a summary of the articles to emphasize the major findings in the paper and their medical implications for the non-specialist reader. Please provide a draft summary of your article highlighting

- the medical issue you are addressing,

- the results obtained and

- their clinical impact.

This may be edited to ensure that readers understand the significance and context of the research. Please refer to any of our published articles for an example.

12) For more information: There is space at the end of each article to list relevant web links for further consultation by our readers. Could you identify some relevant ones and provide such information as well? Some examples are patient associations, relevant databases, OMIM/proteins/genes links, author's websites, etc...

13) Author contributions: the contribution of every author must be detailed in a separate section (before the acknowledgments).

14) A Conflict of Interest statement should be provided in the main text.

15) Every published paper now includes a 'Synopsis' to further enhance discoverability. Synopses are displayed on the journal webpage and are freely accessible to all readers. They include a short stand first (maximum of 300 characters, including space) as well as 2-5 one-sentences bullet points that summarizes the paper. Please write the bullet points to summarize the key NEW findings. They should be designed to be complementary to the abstract - i.e. not repeat the same text. We encourage inclusion of key acronyms and quantitative information (maximum of 30 words / bullet point). Please use the passive voice. Please attach these in a separate file or send them by email, we will incorporate them accordingly.

Please also suggest a striking image or visual abstract to illustrate your article as a PNG file 550 px wide x 300-600 px high.

EMBO Molecular Medicine has a "scooping protection" policy, whereby similar findings that are published by others during review or revision are not a criterion for rejection. Should you decide to submit a revised version, I do ask that you get in touch after three months if you have not completed it, to update us on the status.

***** Reviewer's comments *****

Referee #1 (Remarks for Author):

Malik et al. demonstrated that increased protein expression of ILEI through uPA activation was linked to psoriatic pathogenesis in epidermis autonomous manner, and claimed that targeting of ILEI or uPA was relevant to novel therapeutic strategy for psoriasis. The manuscript is well written and reveals a novel ILEI signature as one of the drivers in psoriasis, however, there are many concerns to be solved.

Major comments
1. Fig.1; They need to check out the ILEI expression in other chronic skin diseases with epidermal hyperplasia, like chronic dermatitis or prurigo, or those with neutrophilic dermatitis such as subcorneal pustulosis, to explore if this is the case with psoriasis alone.

2. Given that psoriatic epidermis showed increased ILEI protein levels but not ILEI mRNA, what do the authors consider the most upstream intrinsic signal of ILEI over-expression at protein level in psoriatic epidermis would be? uPA? If so, what would be the mechanism contributing to upregulation of uPA in psoriasis?

3. They should corroborate if elevation of uPA expression occurs in psoriatic epidermis by immunohistochemistry.

4. Given that ILEI protein levels correlated with psoriatic disease severity (Fig. 1D), how about the correlation between uPA levels (protein or mRNA) and psoriatic severity?

5. They should examine whether ILEI and uPA protein levels in patients with psoriasis could be attenuated after treatment.

6. In K5-ILEIind mice treated with TPA, they should investigate the skin levels of IL-17A, IL-17C, IL-36, IL-22, IL-23 in comparison with control mice.

7. Did ILEI-overexpression affect KC proliferation? How about Ki67 staining of them under low or high Ca conditions in Fig.3F?

8. Fig. 4: In addition to neutrophils, they should examine other important cells in psoriasis in ILEI-overexpressing mice, such as T17 cells (Th17 and Tgd-17), plasmacytoid dendritic cells in comparison with controls.

9. K5-ILEIind(ILEI-overexpressing) KCs need TPA stimulation to recapitulate psoriatic phenotype either in in vivo or in vitro. They should examine if any involvement of PKC signal in collaboration with the ILEI-Akt/Erk-PS727-Stat3 pathway.

10. What do they consider is the biological relevance of TPA in the context of psoriasis pathogenesis? Since a previous study revealed that TPA stimulation resulted in transactivation of EGFR signaling in KCs(CANCER RESEARCH 64, 2382-2389, 2004), it would be helpful to investigate whether EGFR ligands such as HB-EGF and amphiregulin was involved in ILEI-overexpressing KCs to exert the psoriatic phenotype when treated with TPA stimulation (Fig.5F).

11. Please examine any effect of EGFR inhibitor on TPA-treated K5-ILEIind KCs in Fig. 5F.

12. The results of Western blotting in Fig.5D is unclear, so this reviewer does not agree what they claim. It looked that pS727-Stat3 was dependent on ILEI and TPA; pY705 was not affected by any stumuli; pErk was increased mainly by TPA but partially by ILEI; pAkt was downed by TPA.

13. They should also use human primary KCs to reproduce the results of Fig. 4F to I, Fig.5D, E and G.

14. In Fig. 4F-I, they should also examine mRNAs for IL-17c, IL-36 and EGFR ligands like HB-EGF.

15. Attenuation of psoriatic phenotype in K5.ILEI-KO looks marginal based on epidermal thickness (Fig. 6C). Were there changes in skin infiltrates of other cells than neutrophils in KO mice? How about T17 cells?

16. Please reproduce the results in Figure F by use of human primary keratinocytes.

Minor comments
1. The results in Fig.6F and H are redundant, so please combine them.
2. Figure 7: Please quantify the ILEI protein with ELISA assay.
3. Figure 8D: the legands, normal and psoriasis seem opposite.
4. The title "FAM3/ILEI is upregulated" is misleading, since its mRNA levels were unchanged in psoriasis and mouse models as they mentioned.
5. How about the serum ILEI(dimeric form) levels in psoriatic patients? If it is the case, would they be correlated with PASI score?
6. The paragraph starting from line 321to322 seems to be overstated. They cannot exclude any effect of ILEI targeting on immune cells until they examine in detail. In Fig S6, they only checked the effect of ILEI deletion in hematopoietic lineage on any change in the cell population and TPA response in skin.

Referee #2 (Remarks for Author):

Malik et al here examine how FAM3C/ILEI promotes psoriasiform skin inflammation. The study is comprehensive and mechanistic, involving human and mouse studies as well as in vivo and cell culture systems. Complimentary knock-in and knock-out mice are used as well as a chemical inhibitor targeting a potential human drug candidate identified through the investigators RNA-seq analyses. The study is well executed and presented.

The keratinocytes were sorted based on Sca1 expression. Sca1 has been used to identify several different mouse genes, including Casp3, Ly6a and Atxn1. In the interest of avoiding confusion and increasing reproducibility it may be best to use only gene nomenclatures officially approved and used across databases. Also, what is the justification for using this to tell IFE from HF keratinocytes, and how are CD45 negative HF keratinocytes distinct from other CD45 negative cells? Could binding of the anti-Sca1 antibody potentially activate the cells in a way that bias the results? Please explain and revise accordingly.

Point-by-point response to the Reviewers:

Referee #1 (Remarks for Author):

Malik et al. demonstrated that increased protein expression of ILEI through uPA activation was linked to psoriatic pathogenesis in epidermis autonomous manner, and claimed that targeting of ILEI or uPA was relevant to novel therapeutic strategy for psoriasis. The manuscript is well written and reveals a novel ILEI signature as one of the drivers in psoriasis, however, there are many concerns to be solved.

Major comments

1. Fig.1; They need to check out the ILEI expression in other chronic skin diseases with epidermal hyperplasia, like chronic dermatitis or prurigo, or those with neutrophilic dermatitis such as subcorneal pustulosis, to explore if this is the case with psoriasis alone.

The Reviewer is raising an interesting aspect being relevant for understanding how general or how psoriasis-specific our observations are. We have already included in the original submission in Fig.8 the evaluation of the identified ILEI signature in atopic dermatitis (AD) patients, which showed substantial power to stratify AD from normal skin, albeit only moderately compared to psoriasis. Interestingly, the signature was also able to distinguish AD from psoriasis, indicating, that genes of the signature have different weighting in the two diseases and thus, ILEI-linked pathways might have distinctive regulations in the two diseases, which will be certainly exciting to look at in follow-up studies. We included now these thoughts into the **Discussion** (rows 653-660).

To set a more solid basis for the justification of such future studies, we took the advice of the Reviewer and performed ILEI IHC on a small set (n=4) of AD patients and could demonstrate elevated ILEI protein expression in this disease. These data are included now as **Appendix Figure S13** in the revised manuscript. Unfortunately, we did not have access to patient skin biopsies for the additional diseases the Reviewer suggested. Nevertheless, we were able to extend the panel of this explorative analysis with two additional other skin diseases linked to chronic inflammation, Lupus erythematosus (n=2) and Mycosis fungoides (n=4). These also showed variable, but elevated ILEI expression and these data are also part of **Appendix Figure S13** in the revised manuscript. These preliminary data indicate a potential broad relevance of ILEI in chronic inflammatory and hyperplastic skin diseases, which needs intensive and focused research in the future, as we shortly formulate in the **Discussion** of the revised version (rows 661-663).

2. Given that psoriatic epidermis showed increased ILEI protein levels but not ILEI mRNA, what do the authors consider the most upstream intrinsic signal of ILEI over-expression at protein level in psoriatic epidermis would be? uPA? If so, what would be the mechanism contributing to upregulation of uPA in psoriasis?

ILEI has a strong translational control via TGFb signaling (Chaudhury et al., 2010; Waerner et al., 2006) and its secretion is regulated by uPA-uPAR signaling (Csiszar et al., 2014). Plasminogen (Plg) and Kallkrein also play a role indirectly in ILEI secretion, as Plg is needed

for the proteolytic activation of uPA, and kallikrein is able to cleave and activate Plg (Sotiropoulou et al., 2009). Plg plays in addition a role in the proteolytic maturation of ILEI, and other serin proteases, e.g. neutrophil elastase, can also cleave it (Csiszar et al., 2014). It has been described that keratinocyte-specific TGFb overexpression (K5.TGFb) leads to psoriasiform skin inflammation (Han et al., 2010), and that both uPA and plasmin, the activated form of Plg are upregulated in psoriasis (Li et al., 2011; Rubina et al., 2017). To follow the Reviewer's question, we analyzed the expression of these factors in three different settings in the revised MS: **1.** We show that TGFb, uPA and uPAR have elevated expression in two independent psoriasis transcriptomics data sets (**Figure EV1E,F**), in accordance with earlier reports. **2.** We included a heatmap on the expression of these factors in the three analyzed conditions of our RNAseq data on total skin of the K5-ILEI^{ind} mouse model after TPA and acetone treatments in **Figure EV4A**. These data on TPA vs. Acetone treated control skin showed also a clear trend in the upregulation of all these factors, for several of them ILEI overexpression providing an additional trigger on elevated expression, uPA appearing significantly upregulated, as also presented in the originally submitted MS. **3.** To delineate primary cell-intrinsic ILEI-dependent events in skin epidermis after TPA treatment, we performed an additional RNAseq experiment on freshly sorted keratinocytes 24 hours after TPA treatment of K5-ILEI^{ind} and ILEI^{ind} mice for the revision. A heatmap in **Figure EV4E** shows the expression level of above regulatory factors in ILEI overexpressing vs. control keratinocytes in their in vivo context and further confirms the cell-intrinsic significant regulation of uPA expression in keratinocytes by ILEI.

In addition, we included a paragraph in the **Discussion** (rows 559-581) about the known role of TGFb and other regulators of ILEI secretion in psoriasis as a potential link to elevated ILEI expression.

The question, what would regulate uPA in psoriasis, goes beyond the scope of our study. We describe in our mouse model that ILEI itself upregulates uPA expression, by this generating a feed-forward loop on both, ILEI secretion and further increase of uPA expression. This can be a partial answer. Our uPA IHC on psoriatic lesions (see Point 3 for details), showed at the one hand that (i) uPA shows very similar localization to ILEI in both normal and psoriatic epidermis, indicating a tight co-regulation of the two factors, but also made obvious at the other hand that (ii) there are ample and more abundant other sources of uPA beside keratinocytes in the skin, including infiltrating immune cells and Langerhans cells, making this question requesting a very broad level of investigation in the future.

3. They should corroborate if elevation of uPA expression occurs in psoriatic epidermis by immunohistochemistry.

+

4. Given that ILEI protein levels correlated with psoriatic disease severity (Fig. 1D), how about the correlation between uPA levels (protein or mRNA) and psoriatic severity?

It has been demonstrated via IHC and IF studies by others that uPA (and also Plasmin, its activator) is increased in psoriatic lesions at protein level (Li et al., 2011; Rubina et al., 2017). Nevertheless, we do find the Reviewer's request very relevant to re-analyze and extend these data with the question if this upregulation correlates with disease severity. For this, we performed uPA IHC on the same panel of healthy and psoriatic skins as described for ILEI,

presented in **Figure EV5A** in the revised MS. While similarly to ILEI, uPA expression was restricted to basal epidermis in normal skin, in psoriatic lesions it was expressed throughout the thickened epidermis in a perinuclear pattern reminiscent of trans-Golgi and secretory vesicles. The much higher abundance in many other cell types and skin compartments prohibited, however, the quantification of uPA protein levels in keratinocytes on IHC. To overcome this, we utilized available psoriasis transcriptomics data sets and correlated uPA mRNA levels to disease severity. Unfortunately, there were no available data cohorts with annotated severity scores. Thus, we took and Keratin 6 and 16 (K6 and K16) expressions, described as close molecular correlates/markers of psoriasis severity and performed correlation analysis (**Figure EV5B,C**). While K6 or K16 in combination with uPA separated normal from psoriasis very efficiently and thus, showed strong positive correlation with uPA in their expression in both analyzed cohorts, in psoriasis patients alone, the correlation power of uPA to the two "severity" markers was very different in the two analyzed cohorts. Thus, we utilize matched samples from repeated biopsies on the same patients, to reduce the effect of disease/dataset heterogeneity, linking the outcome and conclusion of this part to Point 5 of the Reviewer (please see there follow-up details)

5. They should examine whether ILEI and uPA protein levels in patients with psoriasis could be attenuated after treatment.

While the Reviewer addresses an exciting and clinically highly relevant question, this goes beyond the scope and resources of the current study. We do not have access to tissue material from psoriasis patients underwent some therapy. To be able to look at protein levels, this would be a prospective study requiring a new ethical permission and new sampling resulting in a minimum time of one year needed to finish this request. Unfortunately, proteomics databases, such as ProteomeXchange, do not contain studies comparing normal and disease tissue of psoriasis patients. The strong translational regulation of ILEI and its non-altered mRNA expression in the disease prohibited to utilize transcriptomics data for ILEI changes after therapy. Nevertheless, we had microarray data available on a patient cohort with anti-IL17R (Brodalumab) therapy, and we analyzed uPA mRNA levels in this data set. Results are shown in **Figure EV5E,F** in the revised MS. These data show that at high-dose therapy initial high uPA levels turned back to non-lesional levels after treatment. This was linked to severity reduction based on the correlation plots we generated for KRT16 and PLAU expression on this patient cohort. From this we concluded that (therapy-induced) severity changes were accompanied by concomitant changes in uPA expression.

6. In K5-ILEIind mice treated with TPA, they should investigate the skin levels of IL-17A, IL-17C, IL-36, IL-22, IL-23 in comparison with control mice.

We agree with the Reviewer, these are very important regulatory cytokines in psoriasis. Though we did not observe an ILEI-linked regulation of them, we missed to discuss them specifically. In the revised version, we dedicated a heatmap on the expression of these genes extracted from our RNAseq data set (**Figure EV4C**). These data show that each of these cytokines were upregulated by TPA, and ILEI did not have any further significant effect on them. Genes missing from the heatmap were not detectable in the transcriptomic profiles. Furthermore,

IL17A protein levels analyzed by ELISA on skin protein extracts (Supplemental Figure S4I in the original MS, S6H in the revised version) confirmed the findings on IL17A at mRNA level.

7. Did ILEI-overexpression affect KC proliferation? How about Ki67 staining of them under low or high Ca conditions in Fig.3F?

To address the reviewers request, we established *ex vivo* settings of primary keratinocytes of the *K5-ILEI*^{ind} model for proliferation analysis as well. The suggested conditions of the reviewer were not completely feasible to perform. The *ex vivo* TPA trigger was very efficient to study TPA-driven differences in differentiation, but treatment rather impaired proliferation at all conditions, making it difficult to investigate differences. To overcome this, we performed *ex vivo* proliferation analysis on control and ILEI overexpressing keratinocytes maintained under TPA treatment with or without doxycycline supplementation up to 72 hours after isolation from TPA treated skin of *K5-ILEI*^{ind} and *ILEI*^{ind} mice kept on normal or doxycycline diet. We observed higher proliferation index in ILEI overexpressing keratinocytes. These data are included in **Appendix Figure S2A,B**. At longer cultivation, however, differences faded away preventing us to analyze differential behavior upon *ex vivo* differentiation. Our simplest explanation on these observations is that inflammation induced epidermal hyperproliferation is largely triggered by paracrine feed-back mechanisms deriving from the recruited inflammatory immune cells and the dermal stroma, and different capacity of keratinocytes on the induction of such feed-back regulations (i) would remain abortive at pure *ex vivo* TPA treatment conditions and (ii) might be overwritten soon after isolation by the *ex vivo* culture conditions that consist of a rich cocktail of growth factors needed for proliferation maintenance.

8. Fig. 4: In addition to neutrophils, they should examine other important cells in psoriasis in ILEI-overexpressing mice, such as T17 cells (Th17 and Tgd-17), plasmacytoid dendritic cells in comparison with controls.

We followed the Reviewer's request and performed flow cytometry analysis on TPA/acetone treated back skin of ILEI overexpressing and control mice to cover immune cell populations that could not be analyzed by IHC.

The main IL17 producing cell population in acute murine inflammatory skin are gdT cells (Cai et al., 2011), therefore we focused on the analysis of these cells. We did not observe any ILEI-dependent differences (**Appendix Figure S4G** in the revised MS), which was in accordance with not having seen an ILEI-linked regulation of IL17A cytokine levels in the skin (IL17A ELISA; Supplemental Figure S4I in the original MS, S6H in the revised version).

We also analyzed dendritic cell (DC) populations in these skin samples. Conventional DCs (cDC1 and cDC2) did not show ILEI-dependent regulation, which was in line with our above observations, as cDC2 cells via IL23 stimulus are the main promoters of Th17 and gdT-17 cell differentiation and IL17A production (Wohn et al., 2013). In contrast, plasmacytoid DCs (pDC) were increased at ILEI overexpressing conditions. This is an interesting observation, but as the molecular role of this population is still unclear in psoriasiform mouse models (Glitzner et al., 2014), the meaning of this will need future investigations. We included these data in **Appendix Figure S3C-E** in the revised MS and provided a statement in the **Discussion** (row 544) that ILEI does not seem to regulate Th17 response in the mouse model.

9. K5-ILEIind(ILEI-overexpressing) KCs need TPA stimulation to recapitulate psoriatic phenotype either in in vivo or in vitro. They should examine if any involvement of PKC signal in collaboration with the ILEI-Akt/Erk-PS727-Stat3 pathway.

This is an interesting suggestion of the Reviewer. It is known that PKC activation regulates the phosphorylation of each, Akt, Erk and STAT3. At the one hand, PKC reduces Akt activation indirectly by increasing the activity of PP2A phosphatase. At the other hand, PKC directly activates Erk and STAT3, and being a S/T kinase, phosphorylation occurs on the Ser727 residue of STAT3. All these actions were recapitulated in our signaling Western blots upon TPA treatment. However, while TPA/PKC showed opposing effects on Akt vs. Erk and STAT3 S727 phosphorylation, we observed increased phosphorylation of all three signaling molecules upon ILEI treatment, making it rather unlikely that ILEI had its effect by directly cooperating with PKC signaling. It is also true, however, that PKC represents a big family with up to 10 different isozymes, and PKC effects on Akt, Erk and STAT3 have been assigned to different isozymes. By this, we formally cannot exclude the possibility that ILEI might selectively cooperate with some PKC isoforms, but these analysis would go beyond the scope of current study.

10. What do they consider is the biological relevance of TPA in the context of psoriasis pathogenesis? Since a previous study revealed that TPA stimulation resulted in transactivation of EGFR signaling in KCs(CANCER RESEARCH 64, 2382-2389, 2004), it would be helpful to investigate whether EGFR ligands such as HB-EGF and amphiregulin was involved in ILEI-overexpressing KCs to exert the psoriatic phenotype when treated with TPA stimulation (Fig.5F).

The relevance of TPA in psoriasis pathogenesis is its ability to induce epidermal hyperplasia driven by local inflammation at treatment site. Molecularly, its effect is diverse, with many shared processes described in psoriasis, including autocrine re-wiring effects in KCs, neutrophil and Th17 activation, reciprocal regulation of epidermal cells by immune and stromal cells. STAT3 activation is considered as one of the initial orchestrating events for this.

As the Reviewer cites an earlier seminal study, EGFR signaling plays an important role in STAT3 activation in the skin upon TPA treatment. This prompted us to follow the Reviewer's constructive suggestion and investigated the expression of EGFR ligands in three different settings in the revised MS: **1.** we analyzed their expression in freshly sorted keratinocytes with IFE and HF enrichment of TPA-treated ILEI-overexpressing skin (**Appendix Figure S4J**). While some of them showed TPA-driven upregulation, none of them increased expression in an ILEI-dependent manner. **2.** We included a heatmap on the expression of these factors in the three analyzed conditions of our RNAseq data on total skin of the K5-ILEI^{ind} mouse model after TPA and acetone treatments in **Figure EV4B**. These data on TPA vs. Acetone treated control skin confirmed the TPA-driven regulation of all these ligands, but ILEI overexpression had no additional effect on any of them. **3.** We included a heatmap on the expression of these ligands from the RNAseq experiment on freshly sorted keratinocytes 24 hours after TPA treatment (**Figure EV4F).** These data also did not show strict ILEI-dependent increase of EGF ligands, the only event close to significance was the downregulation of Egf in ILEI-overexpressing KCs. From these three set of experiments we concluded that ILEI overexpression did not lead to elevated expression of EGFR ligands, and thus, the most likely role of EGFR signaling might

be restricted to its known contribution to TPA-driven effects, which as we observed, were, nevertheless, a pre-requisite of an ILEI effect.

11. Please examine any effect of EGFR inhibitor on TPA-treated K5-ILEIind KCs in Fig. 5F.

A very interesting question. EGFR signaling has previously been described to play a role in TPA-induced skin inflammation. However, given the minimal effects of ILEI overexpression on EGFR ligand expression and due to time limitations we did not examine the effect of EGFR inhibition. Nevertheless, we think of it as a possible question to be dissected in more detail in future studies.

12. The results of Western blotting in Fig.5D is unclear, so this reviewer does not agree what they claim. It looked that pS727-Stat3 was dependent on ILEI and TPA; pY705 was not affected by any stumuli; pErk was increased mainly by TPA but partially by ILEI; pAkt was downed by TPA.

We fully agree with the observations of the Reviewer, and had to notice that we did a very poor description of these blots. We thank to the Reviewer pointing out this and helping in improving clear communication of our results. Originally, we focused only onto the ILEI-relevant effects, although they appear as very subtle and late events compared to the robust TPA effects, which are the eye-catchers on these blots.

In the original submission, all that the Reviewer stated we described with the one superficial sentence: "Phosphorylation changes of the initial 30 minutes did not show ILEI, but TPA dependence.", and directly continued with the detailed description of the ILEI-specific changes we observed at 4 hours after treatment start.

We changed the text substantially in the revised version as following: "TPA stimuli had robust effect on STAT3 serine phosphorylation, Erk activation and downregulation of Akt phosphorylation, high base-line levels of tyrosine-phosphorylated STAT3 were not affected. Importantly, co-treatment with ILEI did not show any specific effects within the first hour after TPA stimuli. (Fig.5D,E)." (rows 279-282). Only then, we draw the attention on the changes at 4 hours and describe the observed ILEI-specific changes (rows 282-288).

In addition, to emphasize that all differences were late events, we added the following sentence at the end of this paragraph: "The late effect of ILEI-specific changes in signaling activities might be indicative for a regulatory feed-back loop on these signaling molecules by ILEI rather than a direct activation by ILEI signaling." (rows 289-291). We hope the Reviewer agrees with these formulations.

13. They should also use human primary KCs to reproduce the results of Fig. 4F to I, Fig.5D, E and G.

We agree with the Reviewer, recapitulating our findings on human primary keratinocytes is of additive value on the human relevance of our results. We followed the Reviewer's request and established cooperation with the laboratory of Michael Mildner at the Dermatology Department of our University for human KC studies.

As the most robust ILEI target gene in the mouse KC setting, we used TNFa expression as read-out for initial tests and treatment optimization. To focus on primary ILEI effects, analysis was performed after 8 hours of stimuli, the shortest time point that was analyzed in murine KC settings. First, we confirmed the dose dependent effect of human recombinant ILEI on TNFa expression after TPA stimulus on primary KCs of one healthy donor, by this also determining the optimal dose of the recombinant protein. Next, we analyzed the TPA and ILEI-dependent changes in the expression of TNFa, IL1a and CXCL1 in primary KCs of three independent donors. We could recapitulate our findings in mouse KCs for each of these genes in most donors, only donor 1 failed to upregulate IL1a in an ILEI dependent manner. Of note, differences in the amplitude of TPA and/or ILEI responsiveness of the individual donors were very high, reflecting the individual heterogeneity of human primary materials and preventing us to pool these data at this case number for statistical analysis. Still, the analysis of three independent donors further strengthen the significance of our findings. These data were included in **Appendix Figure S7A,B** in the revised MS.

In our attempts to conduct signaling experiments in primary human keratinocytes, we faced challenges in achieving synchronized culture conditions and very high baseline signaling activities. This was particularly challenging due to the delayed and subtle nature of ILEI effects observed in mouse keratinocyte cultures, which were likely due to secondary feedback regulatory events impacting the signaling activities under investigation. The observed differences between mouse and human keratinocytes may be attributed to the differences in culture and isolation conditions. To be more precise, mouse cells were cultured on culture vessels coated with collagen and fibronectin, while human cells were cultured on uncoated vessels. Since it is well-established that the interaction between keratinocytes and matrix proteins can affect cell signaling, this difference between the mouse and human cell cultures could be of great significance. Moreover, mouse cells were stimulated the day after isolation, whereas human keratinocytes were isolated and grown for up to 10 days in the first passage, after which they were frozen and then thawed for the experiments. The increased population doublings of human keratinocytes may also be an important factor that affects their differentiation state and subsequently, their signaling activities. Due to the inherent dissimilarities between mouse and human keratinocytes, it is inevitable to encounter variations in the procedures utilized. These variations are acceptable and cannot be disregarded. Despite the challenges in capturing the transient signaling events leading to these effects, we observed a clear ILEI responsiveness in the expression of target genes in human keratinocyte cultures, which was highly appreciated.

14. In Fig. 4F-I, they should also examine mRNAs for IL-17c, IL-36 and EGFR ligands like HB-EGF.

The Reviewer’s comment was well taken. Thus, we systemically analyzed the expression of all relevant EGFR ligands, IL17C and IL36 family members in control and ILEI overexpressing keratinocytes. To be closest to physiological conditions, we started the analysis on freshly isolated KCs of TPA-treated skin (the material used in Fig. 4E). None of these factors showed ILEI-dependent upregulation in these samples, data included in **Appendix Figure S4J** in the revised MS.

15. Attenuation of psoriatic phenotype in K5.ILEI-KO looks marginal based on epidermal thickness (Fig. 6C). Were there changes in skin infiltrates of other cells than neutrophils in KO mice? How about T17 cells?

Neutrophil infiltrates were the major effect in ILEI overexpressing conditions, and ILEI deletion recapitulated these findings reversely. As other cell types, the analysis of which is presented in Supplemental Figure 6, showed no differences in ILEI overexpressing skin, similarly we did not observe upregulation in IL17A and IL23 expression in ILEI overexpressing context, we focused only onto the positive results and did not re-investigate all these populations at loss-of-function conditions. The simplest explanation of the "marginal" effect we see in the fact that TPA induces the expression of several cytokines and growth factors that can act on the upregulation of neutrophil recruiting factors. Our overexpression data indicate that ILEI did not regulate these other factors, thus they might not act downstream of ILEI. The moderate effect of ILEI deletion might indicate that these factors also might not act through ILEI, but probably parallel in a partially redundant fashion. Alternatively, but not mutually exclusively, as our ILEI IHC data clearly indicate elevated expression of the protein also in many other cells in the inflamed skin, e.g. dermal stromal fibroblasts, diverse recruited immune cells, including neutrophils, this ILEI source might have functionally compensated the lack of ILEI in keratinocytes. Future studies with advanced mouse genetics and upon the availability of specific ILEI inhibitors will be needed to resolve this question.

16. Please reproduce the results in Figure7 F by use of human primary keratinocytes.

The Reviewer's request was well taken, findings on ILEI-dependent uPA and uPAR expression were analyzed in hKCs using the experimental design described in details in Point 13. uPAR showed only TPA-dependent regulation. Upregulation of uPA expression was further significantly elevated by ILEI co-treatment in two of the three donors, third showing very high upregulation after TPA treatment alone, that might have desensitized for additional ILEI effects. These results are shown in **Appendix Figure S7D** in the revised MS.

Minor comments

1. The results in Fig.6F and H are redundant, so please combine them.

We thank the reviewer recognizing this opportunity for simplification. We **combined** the two panels **as Figure 6F** in the revised version of the MS.

2. Figure 7: Please quantify the ILEI protein with ELISA assay.

The Reviewer formulated our dreams to have purchasable ELISA kits for the quantification of murine ILEI. Unfortunately, this is currently not the case. There are a few reports on the use of ILEI ELISA for the quantification of the human protein, but also these reports did not provide information on the manufacturer. There is a single report on ELISA-based mouse ILEI quantification, and this scientific report is basically about the development of an ILEI sandwich ELISA (Nakano et al., 2021) indicating that those authors also missed purchasable validated resources. We gave a trial and tested one human ILEI ELISA kit with the hope for murine cross-reactivity, as we previously validated the capturing antibody of the kit for cross-reaction with murine ILEI, but the assay did not work for the murine protein. Thus, **as of current state,**

Western blotting stays the only option. We hope that increasing visibility and interest on ILEI research, e.g. through studies like ours, will also facilitate the commercial development of resources required for such studies.

3. Figure 8D: the legands, normal and psoriasis seem opposite.

We thank the reviewer pointing out this inconsistency. The algorithm that generated this plot included a directionality aspect at color coding, but its logics, just as for the Reviewer, also did not find general agreement among tested colleagues. Thus, we followed the reviewer's suggestion and **simplified color coding** by marking both, normal and psoriasis "side" with the same color and including an arrow from normal to psoriasis to know in which direction up and down-regulations are to interpret. Most of our "test persons" found this helpful, we hope the Reviewer also shares this opinion.

4. The title "FAM3/ILEI is upregulated" is misleading, since its mRNA levels were unchanged in psoriasis and mouse models as they mentioned.

We thank to the Reviewer on the constructive criticism on the title, this prompted us to make more thoughts on a correct formulation. In the revised version, we changed the title from "FAM3C/ILEI is upregulated in psoriatic patients…" to "**FAM3C/ILEI protein is elevated in psoriatic lesions…"** also correcting that our analysis was restricted to lesional histological samples and did not include analysis on systemic ILEI levels of patients.

5. How about the serum ILEI(dimeric form) levels in psoriaticpatients? If it is the case, would they be correlated with PASI score?

This is indeed an extremely striking question, if (elevated) ILEI levels could be detected in the serum of psoriatic patients. Unfortunately, **we do not have access to such patient material**. PASI score includes among its scoring parameters the total area of psoriatic lesions as well as epidermal thickness, both correlating with the number of lesional keratinocytes as well as with the overall abundance of recruited inflammatory immune cells, the two most important sources of ILEI protein in psoriatic lesions based on our findings by IHC. Hence, we can only speculate that if ILEI would be detectable in the serum of psoriatic patients, we could expect that it would correlate with disease severity/PASI score.

This will be a very interesting question of future studies with high clinical impact.

6. The paragraph starting from line 321to322 seems to be overstated. They cannot exclude any effect of ILEI targeting on immune cells until they examine in detail. In Fig S6, they only checked the effect of ILEI deletion in hematopoietic lineage on any change in the cell population and TPA response in skin.

We agree with the Reviewer, we analyzed the effect of hematopoietic ILEI deletion only at homeostatic conditions and upon topical administration of the inflammatory agent TPA. The immune system faces ample other potential challenges, the ILEI relevance of which will need detailed examination. To reflect better these limitations of our study, we **toned down** the conclusion from "Thus, loss of ILEI expression in immune cells does not induce an apparent inflammatory phenotype and therefore makes ILEI a potentially feasible therapeutic candidate for psoriasis." **to**: "Thus, ILEI loss in immune cells **was well tolerated both at homeostatic**

conditions and in a first test of a local inflammatory trigger. These preliminary data make ILEI a potentially feasible therapeutic candidate for psoriasis treatment."

Referee #2 (Remarks for Author):

Malik et al here examine how FAM3C/ILEI promotes psoriasiform skin inflammation. The study is comprehensive and mechanistic, involving human and mouse studies as well as in vivo and cell culture systems. Complimentary knock-in and knock-out mice are used as well as a chemical inhibitor targeting a potential human drug candidate identified through the investigators RNA-seq analyses. The study is well executed and presented.

The keratinocytes were sorted based on Sca1 expression. Sca1 has been used to identify several different mouse genes, including Casp3, Ly6a and Atxn1. In the interest of avoiding confusion and increasing reproducibility it may be best to use only gene nomenclatures officially approved and used across databases.

We thank the Reviewer pointing out this inconsistency. The official name of the gene we meant here is indeed Ly6a. To avoid confusion, we have **changed "Sca1" to "Ly6a/Sca1"** in the revised version of the MS; first providing the official name and then, separated by a slash, the name frequently used in the skin field.

Also, what is the justification for using this to tell IFE from HF keratinocytes, and how are CD45 negative HF keratinocytes distinct from other CD45 negative cells?

Thanks the reviewer for raising this point. To briefly explain our justification: only the epidermal sheet was taken for the FACS single cell suspension after being separated from the dermis by tryptic digestion. Hence, CD45 negative cells in this compartment should consist only epidermal cells. The marker Ly6a/Sca1 was taken to separate then IFE cells (Ly6a+) from HF cells (Ly6a-), see also *(Sakamoto et al., 2022).* Based on this recent publication we could calculate that with our gating we have to expect 9% HF-derived cells in the IFE population, as cells of the infundibulum (IFD) that forms the apical connection of the HF to the IFE are Ly6a+, but classified as a HF subcompartment. Similarly, our "HF" population is expected to contain potentially up to 5% "non-HF" cells of undefined origin and misses IFD. To reflect the described 9-14% inpurities of the sorted populations and avoid an oversimplified use of these terms , we have cited the Nagao paper in the materials and methods part as state-of-the-art marker sets for KC subpopulation sorting and **tuned down the text writing "enriched for IFE and HF" instead of "IFE and HF"** (first appearance rows 238-239).

Could binding of the anti-Sca1 antibody potentially activate the cells in a way that bias the results? Please explain and revise accordingly.

We agree with the Reviewer, such considerations have to be kept in mind in all type of FACS based cellular enrichment. Ly6a/Sca1 is a surface marker broadly used in skin biology for the identification and sorting of epidermal subpopulations (Joost et al., 2016; Sakamoto et al., 2022). To our best knowledge, **there is no study and observation reported about a potential activation of epidermal cells upon the binding of this antibody.**

Here, cells were sorted at 4°C, by this any potential alterations in cellular behavior were kept at absolute minimum. In addition, as control and ILEI overexpressing cohorts were propagated identically, both would had been equally affected. Finally, our observations were generally valid for both, the Sca1+ IFE and the Sca1- HF epidermal cells and we do not claim for any specific effect of ILEI overexpression in the IFE or HF compartment. Thus, even if Sca1 binding would alter IFE cells in any way, our similar observations on Sca1- HF cells highly support that the ILEI-linked effects would not be a consequence of such an alteration.

Reference List:

Cai, Y., X. Shen, C. Ding, C. Qi, K. Li, X. Li, V.R. Jala, H.G. Zhang, T. Wang, J. Zheng, and J. Yan. 2011. Pivotal role of dermal IL-17-producing gammadelta T cells in skin inflammation. *Immunity* 35:596-610.

Chaudhury, A., G.S. Hussey, P.S. Ray, G. Jin, P.L. Fox, and P.H. Howe. 2010. TGF-beta-mediated phosphorylation of hnRNP E1 induces EMT via transcript-selective translational induction of Dab2 and ILEI. *Nat Cell Biol* 12:286-293.

Csiszar, A., B. Kutay, S. Wirth, U. Schmidt, S. Macho-Maschler, M. Schreiber, M. Alacakaptan, G.F. Vogel, K. Aumayr, and L.A. Huber. 2014. Interleukin-like epithelial-to-mesenchymal transition inducer activity is controlled by proteolytic processing and plasminogen–urokinase plasminogen activator receptor system–regulated secretion during breast cancer progression. *Breast Cancer Research* 16:1-18.

Glitzner, E., A. Korosec, P.M. Brunner, B. Drobits, N. Amberg, H.B. Schonthaler, T. Kopp, E.F. Wagner, G. Stingl, M. Holcmann, and M. Sibilia. 2014. Specific roles for dendritic cell subsets during initiation and progression of psoriasis. *EMBO molecular medicine* 6:1312-1327.

Han, G., C.A. Williams, K. Salter, P.J. Garl, A.G. Li, and X.J. Wang. 2010. A role for TGFbeta signaling in the pathogenesis of psoriasis. *The Journal of investigative dermatology* 130:371-377.

Joost, S., A. Zeisel, T. Jacob, X. Sun, G. La Manno, P. Lonnerberg, S. Linnarsson, and M. Kasper. 2016. Single-Cell Transcriptomics Reveals that Differentiation and Spatial Signatures Shape Epidermal and Hair Follicle Heterogeneity. *Cell systems* 3:221-237 e229.

Li, Q., F. Ke, W. Zhang, X. Shen, Q. Xu, H. Wang, X.Z. Yu, Q. Leng, and H. Wang. 2011. Plasmin plays an essential role in amplification of psoriasiform skin inflammation in mice. *PloS one* 6:e16483.

Nakano, M., Y. Mitsuishi, L. Liu, N. Watanabe, E. Hibino, S. Hata, T. Saito, T.C. Saido, S. Murayama, K. Kasuga, T. Ikeuchi, T. Suzuki, and M. Nishimura. 2021. Extracellular Release of ILEI/FAM3C and Amyloid-beta Is Associated with the Activation of Distinct Synapse Subpopulations. *Journal of Alzheimer's disease : JAD* 80:159-174.

Rubina, K., V.Y. Sysoeva, E. Zagorujko, Z. Tsokolaeva, M. Kurdina, Y.V. Parfyonova, and V. Tkachuk. 2017. Increased expression of uPA, uPAR, and PAI-1 in psoriatic skin and in basal cell carcinomas. *Archives of dermatological research* 309:433-442.

Sakamoto, K., S. Goel, A. Funakoshi, T. Honda, and K. Nagao. 2022. Flow cytometry analysis of the subpopulations of mouse keratinocytes and skin immune cells. *STAR protocols* 3:101052.

Sotiropoulou, G., G. Pampalakis, and E.P. Diamandis. 2009. Functional roles of human kallikrein-related peptidases. *The Journal of biological chemistry* 284:32989-32994.

Waerner, T., M. Alacakaptan, I. Tamir, R. Oberauer, A. Gal, T. Brabletz, M. Schreiber, M. Jechlinger, and H. Beug. 2006. ILEI: a cytokine essential for EMT, tumor formation, and late events in metastasis in epithelial cells. *Cancer cell* 10:227-239.

Wohn, C., J.L. Ober-Blobaum, S. Haak, S. Pantelyushin, C. Cheong, S.P. Zahner, S. Onderwater, M. Kant, H. Weighardt, B. Holzmann, B. Reizis, B. Becher, E.P. Prens, and B.E. Clausen. 2013. Langerin(neg) conventional dendritic cells produce IL-23 to drive psoriatic plaque formation in mice. *Proceedings of the National Academy of Sciences of the United States of America* 110:10723-10728.

11th Apr 2023

Dear Dr. Csiszar,

Thank you for the submission of your revised manuscript to EMBO Molecular Medicine. I am pleased to inform you that we will be able to accept your manuscript pending the following final amendments:

1) In the main manuscript file, please do the following:
- Correct/answer the track changes suggested by our data editors by working from the attached document.
- Reduce keywords to max 5.
- Remove "Abbreviations".
- Remove "Study explains".
- Remove "Summary of Appendix".
- In M&M, please include statement that in addition to the principles set out in the WMA Declaration of Helsinki the experiments also conformed the Department of Health and Human Services Belmont Report.
- Please rename "Competing Interest" to "Disclosure Statement & Competing Interests". We updated our journal's competing interests policy in January 2022 and request authors to consider both actual and perceived competing interests. Please review the policy https://www.embopress.org/competing-interests and update your competing interests if necessary.
- Author contributions: Please remove it from the manuscript and specify author contributions in our submission system. CRediT has replaced the traditional author contributions section because it offers a systematic machine-readable author contributions format that allows for more effective research assessment. You are encouraged to use the free text boxes beneath each contributing author's name to add specific details on the author's contribution. More information is available in our guide to authors:
https://www.embopress.org/page/journal/17574684/authorguide#authorshipguidelines
- Please remove information about deposited source data (add this information to the blue box at the end of the page of Source data checklist) and leave only information about RNA sequencing. In addition to the accession number please provide URL of all deposited datasets. Please be aware that all datasets should be made freely available upon acceptance, without restriction. Use the following format to report the accession number of your data:

The datasets produced in this study are available in the following databases:
[data type]: [full name of the resource] [accession number/identifier] ([doi or URL or identifiers.org/DATABASE:ACCESSION])

Please check "Author Guidelines" for more information.
https://www.embopress.org/page/journal/17574684/authorguide#availabilityofpublishedmaterial
2) Appendix: Please add table of content with page numbers on the first page. Also, move all M&M to the main manuscript file.
3) The Paper Explained: Please add a summary of the study to the main manuscript file structured as followed: PROBLEM - the medical issue you are addressing, RESULTS - the results obtained, and IMPACT - their clinical impact. Please refer to any of our published primary research articles for an example. Please check "Author Guidelines" for more information.
https://www.embopress.org/page/journal/17574684/authorguide#researcharticleguide
4) Synopsis: Every published paper now includes a 'Synopsis' to further enhance discoverability. Synopses are displayed on the journal webpage and are freely accessible to all readers. They include separate synopsis image and synopsis text.
- Synopsis image: Please provide a striking image or visual abstract as a high-resolution jpeg file 550 px-wide x (250-400)-px high to illustrate your article.
- Synopsis text: Please provide a short standfirst (maximum of 300 characters, including space) as well as 2-5 one sentence bullet points that summarise the paper as a .doc file. Please write the bullet points to summarise the key NEW findings. They should be designed to be complementary to the abstract - i.e. not repeat the same text. We encourage inclusion of key acronyms and quantitative information (maximum of 30 words / bullet point). Please use the passive voice.
- Please check your synopsis text and image before submission with your revised manuscript. Please be aware that in the proof stage minor corrections only are allowed (e.g., typos).
5) For more information: This space should be used to list relevant web links for further consultation by our readers. Could you identify some relevant ones and provide such information as well? Some examples are patient associations, relevant databases, OMIM/proteins/genes links, author's websites, etc...
6) Source data: Please make sure that all requested source data are either submitted to our system or uploaded to EBI repository. Currently source data for Figure 6H and Appendix Figures S1B and S1C are missing.
7) As part of the EMBO Publications transparent editorial process initiative (see our Editorial at http://embomolmed.embopress.org/content/2/9/329), EMBO Molecular Medicine will publish online a Review Process File (RPF) to accompany accepted manuscripts. This file will be published in conjunction with your paper and will include the anonymous referee reports, your point-by-point response and all pertinent correspondence relating to the manuscript. Let us know whether you agree with the publication of the RPF and as here, if you want to remove or not any figures from it prior to publication. Please note that the Authors checklist will be published at the end of the RPF.
8) Please provide a point-by-point response letter to my comments (as Word file).

I look forward to reading a new revised version of your manuscript as soon as possible.

Yours sincerely,

Zeljko Durdevic

Zeljko Durdevic
Editor
EMBO Molecular Medicine

*** Instructions to submit your revised manuscript ***

*** PLEASE NOTE *** As part of the EMBO Publications transparent editorial process initiative (see our Editorial at https://www.embopress.org/doi/pdf/10.1002/emmm.201000094), EMBO Molecular Medicine will publish online a Review Process File to accompany accepted manuscripts.

In the event of acceptance, this file will be published in conjunction with your paper and will include the anonymous referee reports, your point-by-point response and all pertinent correspondence relating to the manuscript. If you do NOT want this file to be published, please inform the editorial office at contact@embomolmed.org.

When submitting your revised manuscript, please include:

1) a .docx formatted version of the manuscript text (including Figure legends and tables)

2) Separate figure files*

3) supplemental information as Expanded View and/or Appendix. Please carefully check the authors guidelines for formatting Expanded view and Appendix figures and tables at https://www.embopress.org/page/journal/17574684/authorguide#expandedview

4) a letter INCLUDING the reviewer's reports and your detailed responses to their comments (as Word file).

5) The paper explained: EMBO Molecular Medicine articles are accompanied by a summary of the articles to emphasize the major findings in the paper and their medical implications for the non-specialist reader. Please provide a draft summary of your article highlighting
- the medical issue you are addressing,
- the results obtained and
- their clinical impact.
This may be edited to ensure that readers understand the significance and context of the research.
Please refer to any of our published articles for an example.

6) For more information: There is space at the end of each article to list relevant web links for further consultation by our readers. Could you identify some relevant ones and provide such information as well? Some examples are patient associations, relevant databases, OMIM/proteins/genes links, author's websites, etc...

7) Author contributions: the contribution of every author must be detailed in a separate section.

8) EMBO Molecular Medicine now requires a complete author checklist (https://www.embopress.org/page/journal/17574684/authorguide) to be submitted with all revised manuscripts. Please use the checklist as guideline for the sort of information we need WITHIN the manuscript. The checklist should only be filled with page numbers were the information can be found. This is particularly important for animal reporting, antibody dilutions (missing) and exact values and n that should be indicted instead of a range.

9) Every published paper now includes a 'Synopsis' to further enhance discoverability. Synopses are displayed on the journal webpage and are freely accessible to all readers. They include a short stand first (maximum of 300 characters, including space) as well as 2-5 one sentence bullet points that summarise the paper. Please write the bullet points to summarise the key NEW findings. They should be designed to be complementary to the abstract - i.e. not repeat the same text. We encourage inclusion of key acronyms and quantitative information (maximum of 30 words / bullet point). Please use the passive voice. Please attach these in a separate file or send them by email, we will incorporate them accordingly.

You are also welcome to suggest a striking image or visual abstract to illustrate your article. If you do please provide a jpeg file 550 px-wide x 400-px high.

10) A Conflict of Interest statement should be provided in the main text

11) Please note that we now mandate that all corresponding authors list an ORCID digital identifier. This takes <90 seconds to complete. We encourage all authors to supply an ORCID identifier, which will be linked to their name for unambiguous name identification.

Currently, our records indicate that the ORCID for your account is 0000-0001-7911-3427.

Please click the link below to modify this ORCID:
Link Not Available

12) The system will prompt you to fill in your funding and payment information. This will allow Wiley to send you a quote for the article processing charge (APC) in case of acceptance. This quote takes into account any reduction or fee waivers that you may be eligible for. Authors do not need to pay any fees before their manuscript is accepted and transferred to our publisher.

*Additional important information regarding Figures

Each figure should be given in a separate file and should have the following resolution:
Graphs 800-1,200 DPI
Photos 400-800 DPI
Colour (only CMYK) 300-400 DPI"

Figures are not edited by the production team. All lettering should be the same size and style; figure panels should be indicated by capital letters (A, B, C etc). Gridlines are not allowed except for log plots. Figures should be numbered in the order of their appearance in the text with Arabic numerals. Each Figure must have a separate legend and a caption is needed for each panel.

*Additional important information regarding figures and illustrations can be found at https://bit.ly/EMBOPressFigurePreparationGuideline. See also figure legend preparation guidelines: https://www.embopress.org/page/journal/17574684/authorguide#figureformat

The system will prompt you to fill in your funding and payment information. This will allow Wiley to send you a quote for the article processing charge (APC) in case of acceptance. This quote takes into account any reduction or fee waivers that you may be eligible for. Authors do not need to pay any fees before their manuscript is accepted and transferred to our publisher.

***** Reviewer's comments *****

Referee #2 (Remarks for Author):

Suitable for publication.

27th Apr 2023

Dear Dr. Durdevic,

With the following point-by-point response to Your decision letter from the 11th of April 2023 we are confirming the fulfillment of all requested amendments. We hope our answers and actions left no open issues remaining from your side that would prevent the final acceptance of our manuscript.

Sincerely,

Agnes Csiszar

“Thank you for the submission of your revised manuscript to EMBO Molecular Medicine. I am pleased to inform you that we will be able to accept your manuscript pending the following final amendments:

1) In the main manuscript file, please do the following:

- Correct/answer the track changes suggested by our data editors by working from the attached document.

We took over all corrections and included all requested explanations. For easy re-identification of requests and our action on them, we inserted the original comments of the data editors at the corresponding positions and provided the information with tracked changes in the revised manuscript.

- Reduce keywords to max 5.

We reduced to number of keywords to 5.

- Remove "Abbreviations".

List of abbreviations was removed.

- Remove "Study explains".

Done.

- Remove "Summary of Appendix".

Done.

- In M&M, please include statement that in addition to the principles set out in the WMA Declaration of Helsinki the experiments also conformed the Department of Health and Human Services Belmont Report.

Requested statement was included into the paragraph of “Human material” in the M&M chapter as following: “Experiments conformed the Department of Health and Human Services Belmont Report.” and marked with tracked changes.

- Please rename "Competing Interest" to "Disclosure Statement & Competing Interests". We updated our journal's competing interests policy in January 2022 and request authors to consider both actual and perceived competing interests. Please review the policy https://www.embopress.org/competing-interests and update your competing interests if necessary.

“Competing Interests” were renamed to “Disclosure Statement & Competing Interests”.

- Author contributions: Please remove it from the manuscript and specify author contributions in our

submission system. CRediT has replaced the traditional author contributions section because it offers a systematic machine-readable author contributions format that allows for more effective research assessment. You are encouraged to use the free text boxes beneath each contributing author's name to add specific details on the author's contribution. More information is available in our guide to authors:
https://www.embopress.org/page/journal/17574684/authorguide#authorshipguidelines
Author contributions were removed from the MS and information was added at the specific site of the online submission form.

- Please remove information about deposited source data (add this information to the blue box at the end of the page of Source data checklist) and leave only information about RNA sequencing. In addition to the accession number please provide URL of all deposited datasets. Please be aware that all datasets should be made freely available upon acceptance, without restriction. Use the following format to report the accession number of your data:
The datasets produced in this study are available in the following databases: [data type]: [full name of the resource] [accession number/identifier] ([doi or URL or identifiers.org/DATABASE:ACCESSION])
Please check "Author Guidelines" for more information.
https://www.embopress.org/page/journal/17574684/authorguide#availabilityofpublishedmaterial
In the Data availability chapter of the MS we included only information on the RNAseq following exactly the requested format. Information about the deposited source data of all other kinds we added into the blue box of the Source data checklist, as requested.

2) Appendix: Please add table of content with page numbers on the first page. Also, move all M&M to the main manuscript file.
Table of content with page numbers was added on the first page of the Appendix, all M&M was moved back to the main manuscript file.

*3) The Paper Explained: Please add a summary of the study **to the main manuscript file** structured as followed: PROBLEM - the medical issue you are addressing, RESULTS - the results obtained, and IMPACT - their clinical impact. Please refer to any of our published primary research articles for an example. Please check "Author Guidelines" for more information.*
https://www.embopress.org/page/journal/17574684/authorguide#researcharticleguide
"The Paper Explained" summary was added to the main manuscript file, inserted directly after the "Abstract" in the requested structure including PROBLEM, RESULTS and IMPACT.

4) Synopsis: Every published paper now includes a 'Synopsis' to further enhance discoverability. Synopses are displayed on the journal webpage and are freely accessible to all readers. They include separate synopsis image and synopsis text.
- Synopsis image: Please provide a striking image or visual abstract as a high-resolution jpeg file 550 px-wide x (250-400)-px high to illustrate your article.
High-resolution jpg file was uploaded for "Synopsis image".

- Synopsis text: Please provide a short standfirst (maximum of 300 characters, including space) as well as 2-5 one sentence bullet points that summarise the paper as a .doc file. Please write the bullet points to summarise the key NEW findings. They should be designed to be complementary to the

abstract - i.e. not repeat the same text. We encourage inclusion of key acronyms and quantitative information (maximum of 30 words / bullet point). Please use the passive voice.
"Synopsis text" conform to the listed criteria was uploaded onto the Submission portal.

- Please check your synopsis text and image before submission with your revised manuscript. Please be aware that in the proof stage minor corrections only are allowed (e.g., typos).
Both text and graphics were checked and "test read" by several individuals before upload to minimize the risk of inconsistencies.

5) For more information: This space should be used to list relevant web links for further consultation by our readers. Could you identify some relevant ones and provide such information as well? Some examples are patient associations, relevant databases, OMIM/proteins/genes links, author's websites, etc...
We did not identify any relevant web links that would merit particular attention to list as "For more information".

6) Source data: Please make sure that all requested source data are either submitted to our system or uploaded to EBI repository. Currently source data for ***Figure 6H and Appendix Figures S1B and S1C*** *are missing.*
We have to apologize, though all listed missing data were in, they were "hidden" due to an incomplete update on the Figure labeling and numbering in the Source Data File after revision. E.g. data on Figure 6H were still in a folder named 6I (the old annotation) and Appendix Figures S1B and S1C were former panels of Figure S3. We extended now the Source Data File deposited at Biostudies (URL link and details were added into the blue box of the Source Data File author check list), including the files corresponding to the listed missing once. Every Figure labeling there corresponds now to the labeling of the latest revised version of the manuscript. We hope this will allow to clearly identify the corresponding Source Data of the listed Figure panels.

7) As part of the EMBO Publications transparent editorial process initiative (see our Editorial at http://embomolmed.embopress.org/content/2/9/329), EMBO Molecular Medicine will publish online a Review Process File (RPF) to accompany accepted manuscripts. This file will be published in conjunction with your paper and will include the anonymous referee reports, your point-by-point response and all pertinent correspondence relating to the manuscript. Let us know whether you agree with the publication of the RPF and as here, if you want to remove or not any figures from it prior to publication. Please note that the Authors checklist will be published at the end of the RPF.
I agree that a RPF file will be published without restrictions from my side.

8) Please provide a point-by-point response letter to my comments (as Word file).
It is done and uploaded onto the submission portal.
I look forward to reading a new revised version of your manuscript as soon as possible.
Yours sincerely,
Zeljko Durdevic

8th May 2023

Dear Dr. Csiszar,

We are pleased to inform you that your manuscript is accepted for publication and is now being sent to our publisher to be included in the next available issue of EMBO Molecular Medicine.

Please read below for additional IMPORTANT information regarding your article, its publication and the production process.

Congratulations on your interesting work,

Zeljko Durdevic

Zeljko Durdevic
Editor
EMBO Molecular Medicine

Follow us on Twitter @EmboMolMed
Sign up for eTOCs at embopress.org/alertsfeeds

*** *** *** IMPORTANT INFORMATION *** *** ***

SPEED OF PUBLICATION
The journal aims for rapid publication of papers, using using the advance online publication "Early View" to expedite the process: A properly copy-edited and formatted version will be published as "Early View" after the proofs have been corrected. Please help the Editors and publisher avoid delays by providing e-mail address(es), telephone and fax numbers at which author(s) can be contacted.

Should you be planning a Press Release on your article, please get in contact with embomolmed@wiley.com as early as possible, in order to coordinate publication and release dates.

LICENSE AND PAYMENT:

All articles published in EMBO Molecular Medicine are fully open access: immediately and freely available to read, download and share.

EMBO Molecular Medicine charges an article processing charge (APC) to cover the publication costs. You, as the corresponding author for this manuscript, should have already received a quote with the article processing fee separately. Please let us know in case this quote has not been received.

Once your article is at Wiley for editorial production you will receive an email from Wiley's Author Services system, which will ask you to log in and will present you with the publication license form for completion. Within the same system the publication fee can be paid by credit card, an invoice, pro forma invoice or purchase order can be requested.

Payment of the publication charge and the signed Open Access Agreement form must be received before the article can be published online.

PROOFS

You will receive the proofs by e-mail approximately 2 weeks after all relevant files have been sent o our Production Office. Please return them within 48 hours and if there should be any problems, please contact the production office at embopressproduction@wiley.com.

Please inform us if there is likely to be any difficulty in reaching you at the above address at that time. Failure to meet our

deadlines may result in a delay of publication.

All further communications concerning your paper proofs should quote reference number EMM-2022-16758-V3 and be directed to the production office at embopressproduction@wiley.com.

Thank you,

Zeljko Durdevic
Editor
EMBO Molecular Medicine

EMBO Press Author Checklist

Corresponding Author Name: Agnes Csiszar
Journal Submitted to: EMBO Molecular Medicine
Manuscript Number: EMM-2022-16758

USEFUL LINKS FOR COMPLETING THIS FORM
The EMBO Journal - Author Guidelines
EMBO Reports - Author Guidelines
Molecular Systems Biology - Author Guidelines
EMBO Molecular Medicine - Author Guidelines

Reporting Checklist for Life Science Articles (updated January
This checklist is adapted from Materials Design Analysis Reporting (MDAR) Checklist for Authors. MDAR establishes a minimum set of requirements in transparent reporting in the life sciences (see Statement of Task: 10.31222/osf.io/9sm4x). Please follow the journal's guidelines in preparing your
Please note that a copy of this checklist will be published alongside your article.

Abridged guidelines for figures
1. Data
The data shown in figures should satisfy the following conditions:
- the data were obtained and processed according to the field's best practice and are presented to reflect the results of the experiments in an accurate and unbiased manner.
- ideally, figure panels should include only measurements that are directly comparable to each other and obtained with the same assay.
- plots include clearly labeled error bars for independent experiments and sample sizes. Unless justified, error bars should not be shown for technical
- if n<5, the individual data points from each experiment should be plotted. Any statistical test employed should be justified.
- Source Data should be included to report the data underlying figures according to the guidelines set out in the authorship guidelines on Data

2. Captions
Each figure caption should contain the following information, for each panel where they are relevant:
- a specification of the experimental system investigated (eg cell line, species name).
- the assay(s) and method(s) used to carry out the reported observations and measurements.
- an explicit mention of the biological and chemical entity(ies) that are being measured.
- an explicit mention of the biological and chemical entity(ies) that are altered/varied/perturbed in a controlled manner.
- the exact sample size (n) for each experimental group/condition, given as a number, not a range;
- a description of the sample collection allowing the reader to understand whether the samples represent technical or biological replicates (including how many animals, litters, cultures, etc.).
- a statement of how many times the experiment shown was independently replicated in the laboratory.
- definitions of statistical methods and measures:
  - common tests, such as t-test (please specify whether paired vs. unpaired), simple χ2 tests, Wilcoxon and Mann-Whitney tests, can be unambiguously identified by name only, but more complex techniques should be described in the methods section;
  - are tests one-sided or two-sided?
  - are there adjustments for multiple comparisons?
  - exact statistical test results, e.g., P values = x but not P values < x;
  - definition of 'center values' as median or average;
  - definition of error bars as s.d. or s.e.m.

Please complete ALL of the questions below.
Select "Not Applicable" only when the requested information is not relevant for your study.

Materials

Newly Created Materials	Information included in the manuscript?	In which section is the information available? (Reagents and Tools Table, Materials and Methods, Figures, Data Availability Section)
New materials and reagents need to be available; do any restrictions apply?	Not Applicable	

Antibodies	Information included in the manuscript?	In which section is the information available? (Reagents and Tools Table, Materials and Methods, Figures, Data Availability Section)
For **antibodies** provide the following information:<br>- Commercial antibodies: RRID (if possible) or supplier name, catalogue number and or/clone number<br>- Non-commercial: RRID or citation	Yes	Materials and Methods (p25, p28, p30 and p31) referring to Appendix Table S2 for details on dilution, campany and catalogue number.

DNA and RNA sequences	Information included in the manuscript?	In which section is the information available? (Reagents and Tools Table, Materials and Methods, Figures, Data Availability Section)
**Short novel DNA or RNA including primers, probes**: provide the sequences.	Yes	Materials and Methods (p27 and p32) referring to Appendix Table S2 for details on sequences

Cell materials	Information included in the manuscript?	In which section is the information available? (Reagents and Tools Table, Materials and Methods, Figures, Data Availability Section)
**Cell lines:** Provide species information, strain. Provide accession number in repository **OR** supplier name, catalog number, clone number, and/**OR** RRID.	Not Applicable	
**Primary cultures:** Provide species, strain, sex of origin, genetic modification status.	Yes	Materials and Methods (p25-26 and p28-29)
Report if the cell lines were recently **authenticated** (e.g., by STR profiling) and tested for mycoplasma contamination.	Not Applicable	

Experimental animals	Information included in the manuscript?	In which section is the information available? (Reagents and Tools Table, Materials and Methods, Figures, Data Availability Section)
**Laboratory animals or Model organisms:** Provide species, strain, sex, age, genetic modification status. Provide accession number in repository **OR** supplier name, catalog number, clone number, **OR** RRID.	Yes	Materials and Methods (p26-27)
**Animal observed in or captured from the field:** Provide species, sex, and age where possible.	Not Applicable	
Please detail **housing and husbandry conditions**.	Yes	Materials and Methods (p26-27)

Plants and microbes	Information included in the manuscript?	In which section is the information available? (Reagents and Tools Table, Materials and Methods, Figures, Data Availability Section)
**Plants:** provide species and strain, ecotype and cultivar where relevant, unique accession number if available, and source (including location for collected wild specimens).	Not Applicable	
**Microbes:** provide species and strain, unique accession number if available, and source.	Not Applicable	

Human research participants	Information included in the manuscript?	In which section is the information available? (Reagents and Tools Table, Materials and Methods, Figures, Data Availability Section)
If collected and within the bounds of privacy constraints report on age, sex and gender or ethnicity for all study participants.	Not Applicable	

Core facilities	Information included in the manuscript?	In which section is the information available? (Reagents and Tools Table, Materials and Methods, Figures, Data Availability Section)
If your work benefited from core facilities, was their service mentioned in the acknowledgments section?	Yes	Acknowledgement (p34)

Design

Study protocol	Information included in the manuscript?	In which section is the information available? (Reagents and Tools Table, Materials and Methods, Figures, Data Availability Section)

If study protocol has been **pre-registered, provide DOI in the manuscript**. For clinical trials, provide the trial registration number **OR** cite DOI.	Not Applicable	
Report the **clinical trial registration number** (at ClinicalTrials.gov or equivalent), where applicable.	Not Applicable	

Laboratory protocol	Information included in the manuscript?	In which section is the information available? (Reagents and Tools Table, Materials and Methods, Figures, Data Availability Section)
Provide DOI OR other citation details if **external detailed step-by-step protocols** are available.	Not Applicable	

Experimental study design and statistics	Information included in the manuscript?	In which section is the information available? (Reagents and Tools Table, Materials and Methods, Figures, Data Availability Section)
Include a statement about **sample size** estimate even if no statistical methods were used.	Yes	Materials and Methods (p34)
Were any steps taken to minimize the effects of subjective bias when allocating animals/samples to treatment (e.g. **randomization procedure**)? If yes, have they been described?	Yes	Materials and Methods (p27)
Include a statement about **blinding** even if no blinding was done.	Yes	Materials and Methods (p27)
Describe **inclusion/exclusion criteria** if samples or animals were excluded from the analysis. Were the criteria pre-established?<br><br>If sample or data points were omitted from analysis, report if this was due to attrition or intentional exclusion and provide justification.	Yes	Materials and Methods (p34)
For every figure, are **statistical tests** justified as appropriate? Do the data meet the assumptions of the tests (e.g., normal distribution)? Describe any methods used to assess it. Is there an estimate of variation within each group of data? Is the variance similar between the groups that are being statistically compared?	Yes	Materials and Methods (p34)

Sample definition and in-laboratory replication	Information included in the manuscript?	In which section is the information available? (Reagents and Tools Table, Materials and Methods, Figures, Data Availability Section)
In the figure legends: state number of times the experiment was **replicated** in laboratory.	Yes	Figure legends (p45-53)
In the figure legends: define whether data describe **technical or biological replicates**.	Yes	Figure legends (p45-53)

Ethics

Ethics	Information included in the manuscript?	In which section is the information available? (Reagents and Tools Table, Materials and Methods, Figures, Data Availability Section)
Studies involving **human participants**: State details of **authority granting ethics approval** (IRB or equivalent committee(s), provide reference number for approval.	Yes	Materials and Methods (p25)
Studies involving **human participants**: Include a statement confirming that **informed consent** was obtained from all subjects and that the experiments conformed to the principles set out in the WMA Declaration of Helsinki and the Department of Health and Human Services Belmont Report.	Yes	Materials and Methods (p25)
Studies involving **human participants:** For publication of **patient photos**, include a statement confirming that consent to publish was obtained.	Not Applicable	
Studies involving experimental **animals**: State details of **authority granting ethics approval** (IRB or equivalent committee(s), provide reference number for approval. Include a statement of compliance with ethical regulations.	Yes	Materials and Methods (p26)
Studies involving **specimen and field samples:** State if relevant **permits** obtained, provide details of authority approving study; if none were required, explain why.	Not Applicable	

Dual Use Research of Concern (DURC)	Information included in the manuscript?	In which section is the information available? (Reagents and Tools Table, Materials and Methods, Figures, Data Availability Section)
Could your study fall under dual use research restrictions? Please check biosecurity documents and list of **select agents and toxins** (CDC): https://www.selectagents.gov/sat/list.htm	Not Applicable	
If you used a select agent, is the security level of the lab appropriate and reported in the manuscript?	Not Applicable	
If a study is subject to dual use research of concern regulations, is the name of the **authority granting approval and reference number** for the regulatory approval provided in the manuscript?	Not Applicable	

Reporting

The MDAR framework recommends adoption of discipline-specific guidelines, established and endorsed through community initiatives. Journals have their own policy about requiring specific guidelines and recommendations to complement MDAR.

Adherence to community standards	Information included in the manuscript?	In which section is the information available? (Reagents and Tools Table, Materials and Methods, Figures, Data Availability Section)
State if relevant guidelines or checklists (e.g., **ICMJE, MIBBI, ARRIVE, PRISMA**) have been followed or provided.	Not Applicable	
For **tumor marker prognostic studies,** we recommend that you follow the **REMARK** reporting guidelines (see link list at top right). See author guidelines, under 'Reporting Guidelines'. Please confirm you have followed these guidelines.	Not Applicable	
For **phase II and III randomized controlled trials**, please refer to the **CONSORT** flow diagram (see link list at top right) and submit the CONSORT checklist (see link list at top right) with your submission. See author guidelines, under 'Reporting Guidelines'. Please confirm you have submitted this list.	Not Applicable	

Data Availability

Data availability	Information included in the manuscript?	In which section is the information available? (Reagents and Tools Table, Materials and Methods, Figures, Data Availability Section)
Have **primary datasets** been deposited according to the journal's guidelines (see 'Data Deposition' section) and the respective accession numbers provided in the Data Availability Section?	Yes	Data Availibility Section (p34)
Were **human clinical and genomic datasets** deposited in a public access-controlled repository in accordance to ethical obligations to the patients and to the applicable consent agreement?	Not Applicable	
Are **computational models** that are central and integral to a study available without restrictions in a machine-readable form? Were the relevant accession numbers or links provided?	Not Applicable	
If publicly available data were reused, provide the respective **data citations in the reference list**.	Yes	reference list (p36-44)
